# Supplementary material for: Phylogenomics and metabolic engineering reveal a conserved gene cluster in Solanaceae plants for withanolide biosynthesis
Source: Nat Commun. 2025 Jul 10;16:6367. doi: 10.1038/s41467-025-61686-1 (PMC12246201; doi:10.1038/s41467-025-61686-1)
Supplement: Supplementary file 1 — Supplementary Information [file 41467_2025_61686_MOESM1_ESM.pdf]

Phylogenomics and metabolic engineering reveal a conserved gene cluster in Solanaceae plants for withanolide biosynthesis

Hakim, Choudhary, Malhotra, Peng, Bültemeier *et al.*

Supplementary Method 1. RNA extraction

RNA from *W. somnifera* root was extracted with the RNA plant and fungi kit (Macherey & Nagel). Homogenization of sample was performed using mortar, pestle and liquid nitrogen. On-column DNA digestion was performed according to the manufacturer's instructions. The extracted RNA quality and quantity was determined using Nanodrop measurement and agarose gel. Paired-end RNA-seq (2 x 150 nt) was conducted using Illumina NovaSeq 6000 (BMKGene).

Supplementary Method 2. Identification of transposable elements

Transposable elements (TEs) were annotated using Extensive *de*-novo TE Annotator[1] (EDTA) v2.2.1 with the following parameters: --genome --cds --anno 1 --sensitive 1 --evaluate 1. EDTA uses various tools like HelitronScanner[2], LTR_FINDER[3,4], LTR_retriever[5], LTRharvest[6], TIR-Learner[7], Generic Repeat Finder[8] and TEsorter[9] to identify and classify novel TEs. Tandem repeats in the genome sequence were identified using Tandem Repeats Finder[10] (TRF) v4.10.0 using parameters 'Match=2, Mismatch=5, Delta=7, PM=80, PI=10, Minscore=5 and MaxPeriod=2000'.

Supplementary Method 3. Synthetic procedures

To determine the C22 stereochemistry of (22*R*)-ergosta-5,24-diene-3β,22-diol (**10**), two pairs of structurally similar C22 epimers **S7-(22*S*/*R*)** and **S9-(22*S*/*R*)** that differ from **10** only in the oxidation of C26 were synthesised. Moreover, (22*R*)-ergosta-5,24-diene-1α,3β,22,26-tetrol (**17**) was accessed by chemical reduction of withanoside V aglycone (**16**). The full synthetic routes are shown in Supplementary Fig. 67.

All reactions sensitive to moisture and/or air were carried out under an atmosphere of argon, using heat gun-dried glassware, and anhydrous solvents. Anhydrous dichloromethane, toluene, tetrahydrofuran and diethyl ether were taken from a M. Braun GmbH MB SPS-800 solvent purification system and were stored over 4 Å molecular sieves. Petroleum ether and tetrahydrofuran were purified by distillation. Stigmasterol was acquired from Thermo Scientific in >90% (GC) purity. Hoveyda Grubbs Catalyst® M721 was provided by Umicore. All other solvents (HPLC quality) and commercially available reagents were used without further purification unless otherwise stated.

Concentration under reduced pressure was performed by rotary evaporation at 40 °C and appropriate pressure, followed by exposure to vacuum (10^{-3} mbar) at 25 °C.

Heating experiments were performed in an oil bath.

Thin layer chromatography: All reactions were monitored using pre-coated TLC sheets ALUGRAM® Xtra SIL G/UV_{254} (0.2 mm, silica gel, F_{254}, aluminium-backed, MACHEREY-NAGEL) with detection by UV light (λ = 254 nm) and/or by staining with an aqueous solution of cerium sulphate and phosphomolybdic acid and heat.

Flash column chromatography: silica gel (0.04-0.063 mm, 240-400 mesh) obtained from MACHEREY-NAGEL. The applied petroleum ether fraction had a bp of 40-60 °C. The eluent is given as volume ratios (v/v).

Automated flash column chromatography: Combiflash NextGen 300+ used with RediSep® Silver columns by Teledyne ISCO or FlashPure Select columns by Büchi. The eluent is given as volume rations (v/v).

NMR experiments were recorded in $CDCl_3$ or methanol-d_4 purchased from deutero GmbH. The following NMR spectrometers were used: Bruker Avance I 400 MHz, Bruker Avance III HD 400 MHz, Bruker Avance III 400 MHz with Prodigy BBFO probe head, Bruker Avance III HD 500 MHz with TCI cryo probe head, Bruker Avance Neo 600 MHz with DUL cryo probe head, Bruker Avance IIIHD 600 MHz with Prodigy TCI probe head. Chemical shifts δ are reported in parts per million (ppm) using residual undeuterated solvent ($CDCl_3$: δ_H = 7.26 ppm, δ_C = 77.16 ppm; methanol-d_4: δ_H = 3.31 ppm, δ_C = 49.00 ppm) as an internal reference at 298 K. The given multiplicities are phenomenological; thus, the actual appearance of the signals is stated and not the theoretically expected one. The following abbreviations are used to designate multiplicities: s = singlet, d = doublet, t = triplet, q = quartet, quint = quintet, or combinations of these acronyms. Broad signals will be denoted by addition of the letter "b" e.g., broad singlet is written as "bs". In case no multiplicity could be identified, the chemical shift range of the signal is given (m = multiplet). Peak integrals are given as multiples of protons *Y*H, with *Y* being the number of protons belonging to the given signal. NMR spectra were processed using MestreNova version 15.0.1. NMR spectra of synthetic compounds are shown in Supplementary Fig. 68-99.

Infrared spectroscopy: Infrared (IR) spectra were measured on SHIMADZU FT-IR Affinity-1S spectrometer. Wavenumbers $\tilde{\nu}$ are given in cm^{-1} and intensities are as follows: s = strong, m = medium, w = weak.

High-resolution mass spectra (HRMS) HR-ESI-MS analysis was performed using either a Waters QTof Premier coupled with a Waters Acquity HPLC incl. a TUV detector or on a Waters LCT Premier mass spectrometer coupled with a Waters Alliance 2695 HPLC. The sample was dissolved in a suitable solvent (approx. 1 mg/mL) and diluted in methanol (1:100). An aliquot of 5 µL was injected in constant flow of methanol and data were recorded in positive or negative ion mode. On the LCT Premier mass spectrometer no HPLC column was installed.

HR-EI-MS analyses were carried out on a Waters Micromass GCT Premier (70 eV) with direct sample inlet.

Optical rotations were measured on a Krüss optronic P3000 or a Perkin-Elmer 241 polarimeter at wavelength of λ_{max} = 589 nm (sodium D-line) using 100 mm cells. The used solvent is specified for each substance and concentration (g/100 mL) is indicated.

Melting points (m. p.) were measured with a MPA100 melting point apparatus by Stanford Research Systems.

Stigmasteryl mesylate (S1)

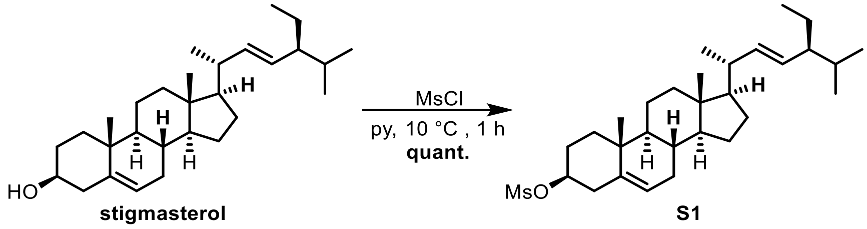

To a stirred solution of stigmasterol (4.50 g, 10.9 mmol, 1.0 equiv.) in pyridine (100 mL) was added methanesulfonyl chloride (4.22 mL, 54.5 mmol, 5.0 equiv.) dropwise at 10 °C. After stirring at 10 °C for 1 h, the reaction mixture was poured into a mixture of ice and water (200 mL) under stirring. The precipitate was filtered off, washed with water (3× 100 mL), and dried under vacuum to give mesylate **S1** (5.35 g, 10.9 mmol, quant.) as a colourless powder, which was used in the next step without further purification.

TLC R_f = 0.44 (PE/EtOAc 7:3, CAM).

m. p. 124–127 °C.

^{1}H-NMR (400 MHz, $CDCl_3$): δ [ppm] = 5.43–5.40 (m, 1H), 5.15 (dd, *J* = 15.2, 8.6 Hz, 1H), 5.02 (dd, *J* = 15.2, 8.6 Hz, 1H), 4.52 (dddd, *J* = 11.0, 11.0, 5.8, 4.7 Hz, 1H), 3.00 (s, 3H), 2.58–2.44 (m, 2H), 2.08–1.94 (m, 4H), 1.90 (dt, *J* = 13.4, 3.6 Hz, 1H), 1.85–1.65 (m, 2H), 1.60–1.36 (m, 8H), 1.32–1.05 (m, 6H), 1.04–1.00 (m, 7H contains 1.03 (d, *J* = 6.5 Hz, 3H), 1.02 (s, 3H)), 0.98–0.89 (m, 1H), 0.84 (d, *J* = 6.4 Hz, 3H), 0.81 (d, *J* = 7.5 Hz, 3H), 0.79 (d, *J* = 7.0 Hz, 3H), 0.70 (s, 3H).

^{13}C-NMR (101 MHz, $CDCl_3$): δ [ppm] = 138.8, 138.4, 129.5, 124.0, 82.2, 56.9, 56.1, 51.4, 50.1, 42.3, 40.6, 39.7, 39.3, 38.9, 37.1, 36.5, 32.02, 32.01, 31.9, 29.1, 29.0, 25.6, 24.5, 21.4, 21.2, 21.2, 19.3, 19.1, 12.4, 12.2.

opt. act. $[\alpha]_D^{24}$ = –43.9 (c=1.20, $CHCl_3$).

HRMS (ESI): *m/z* $[M+Na]^+$ calcd. for $[C_{30}H_{50}O_3NaS]^+$: 513.3373; found: 513.3358.

IR ṽ [cm^{-1}] = 2953 (m), 2932 (m), 2868 (w), 1317 (s), 1172 (s), 964 (m), 939 (s), 925 (s), 879 (m), 871 (s), 561 (m), 529 (s).

All characterization data was consistent with the data reported by Peracaula and co-workers[13].

***i*-Stigmasteryl methyl ether (S2)**

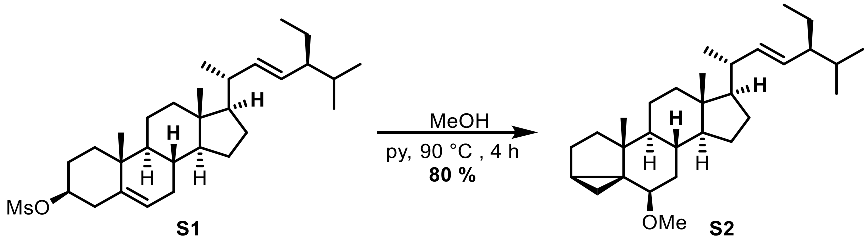

S2 was synthesized according to the procedure published by Bräse and co-workers[14].

Mesylate **S1** (9.60 g, 19.6 mmol, 1.0 equiv.) was suspended in pyridine (32 mL) and methanol (160 mL). After stirring at 90 °C for 4 h, the solvents were removed under reduced pressure and diethyl ether (100 mL) was added. Remaining solids were separated by filtration and washed with diethyl ether (3x 150 mL). The solution was concentrated under reduced pressure, the resulting oil was adsorbed on silica gel and purified by automated flash column chromatography (silica gel, PE/EtOAc 100:0 to 90:10) yielding *i*-steroid **S2**

(7.20 g, 16.9 mmol, 86%) and the isomeric *i*-steroid (1.00 g, 2.34 mmol, 12%) as clear, sticky resins which solidified in the freezer.

TLC R_f = 0.55 (PE/EtOAc 9:1, CAM).
^{1}H-NMR (400 MHz, $CDCl_3$): δ [ppm] = 5.15 (dd, J = 15.2, 8.6 Hz, 1H), 5.01 (dd, J = 15.2, 8.6 Hz, 1H), 3.32 (s, 3H), 2.77 (t, J = 2.9 Hz, 1H), 2.10–2.01 (m, 1H), 1.97 (dt, J = 12.5, 3.4 Hz, 1H), 1.89 (dt, J = 13.5, 3.1 Hz, 1H), 1.81–1.64 (m, 3H), 1.60–1.47 (m, 5H), 1.46–1.36 (m, 3H), 1.30–1.00 (m, 13H, contains 1.02 (s, 3H), 1.01 (d, J = 7.0 Hz, 6H)), 0.91–0.78 (m, 12H, contains 0.85 (d, J=6.4 Hz, 3H)), 0.73 (s, 3H), 0.65 (dd, J = 5.0, 3.8 Hz, 1H), 0.43 (dd, J = 8.0, 5.1 Hz, 1H).
^{13}C-NMR (101 MHz, $CDCl_3$): δ [ppm] = 138.5, 129.4, 82.6, 56.8, 56.7, 56.3, 51.4, 48.2, 43.6, 42.8, 40.7, 40.4, 35.4, 35.2, 33.5, 32.0, 30.6, 29.2, 25.6, 25.1, 24.4, 22.9, 21.6, 21.4, 21.2, 19.4, 19.2, 13.2, 12.6, 12.4.
opt. act. $[\alpha]_D^{23}$ = +32.2 (c=1.20, $CHCl_3$).
HRMS (EI): *m/z* $[M]^{+\bullet}$ calcd. for $[C_{30}H_{50}O]^{+\bullet}$: 426.3856; found: 426.3848.
IR ṽ [cm^{-1}] = 2957 (m), 2945 (s), 2928 (s), 2895 (m), 2864 (s), 1454 (m), 1368 (w), 1089 (s), 1080 (s), 1015 (w), 968 (m), 615 (w).

All characterisation data was consistent with the data reported by Bräse and co-workers[14].

(20*S*)-6*β*-Methoxy-3*α*,5-cyclopregnancarbaldehyde (S3)

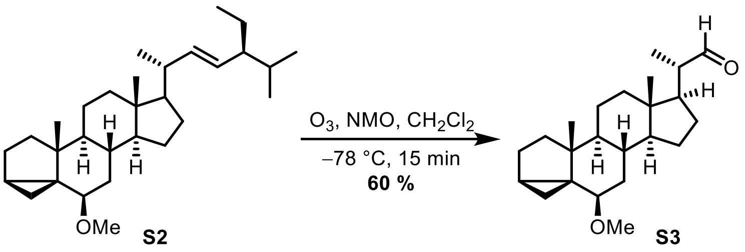

S3 was synthesized according to the procedure published by Dussault and co-workers[15].

To a stirred solution of *i*-stigmasteryl methyl ether (**S2**, 2.09 g, 4.90 mmol, 1.0 equiv.) in CH_2Cl_2 (50 mL) was added *N*-methyl morpholine *N*-oxide (1.72 g, 14.7 mmol, 3.0 equiv.). At –78 °C ozone was bubbled slowly through the stirred solution and the reaction progress was checked by TLC (reaction time 10–15 min). Longer reaction times resulted in oxidation to the carboxylic acid. The reaction mixture was then sparged with O_2 for 2-3 min and allowed to warm to ambient temperature, dried over $MgSO_4$ and concentrated under reduced pressure. The resulting oil was adsorbed on silica gel and purified by automated flash column chromatography (silica gel, PE/EtOAc 100:0 to 90:10) yielding aldehyde **S3** as a colourless oil (1.01 g, 2.93 mmol, 60%) which solidified in the freezer.

TLC R_f = 0.30 (PE/EtOAc 9:1, CAM).
^{1}H-NMR (400 MHz, $CDCl_3$): δ [ppm] = 9.57 (d, J = 3.2 Hz, 1H), 3.31 (s, 3H), 2.77 (t, J = 2.8 Hz, 1H), 2.36 (dqd, J = 10.0, 6.8, 3.2 Hz, 1H), 1.97–1.81 (m, 3H), 1.81–1.61 (m, 3H), 1.56–1.31 (m, 6H), 1.27–1.14 (m, 2H), 1.11 (d, J = 6.8 Hz, 3H), 1.10–1.04 (m, 2H), 1.02 (s, 3H), 0.93–0.78 (m, 3H), 0.76 (s, 3H), 0.68–0.61 (m, 1H), 0.43 (dd, J = 8.0, 5.1 Hz, 1H).
^{13}C-NMR (101 MHz, $CDCl_3$): δ [ppm] = 205.4, 82.4, 56.7, 55.9, 51.3, 49.7, 48.2, 43.5, 43.5, 40.1, 35.3, 35.2, 33.5, 30.6, 27.3, 25.1, 24.7, 22.8, 21.6, 19.4, 13.6, 13.2, 12.7.
opt. act. $[\alpha]_D^{23}$ = +31.5 (c=1.08, $CHCl_3$).
HRMS (EI): *m/z* $[M]^{+\bullet}$ calcd. for $[C_{23}H_{36}O_2]^{+\bullet}$: 344.2710; found: 344.2716.
IR ṽ [cm^{-1}] = 2947 (s), 2933 (s), 2913 (m), 2868 (m), 2845 (w), 1721 (s), 1454 (m), 1445 (w), 1382 (w), 1091 (s), 1018 (m), 999 (w), 970 (w), 943 (w), 613 (w).

All characterisation data was consistent with the data reported by Bräse and co-workers[14].

(22*S*)-24-nor-6*β*-Methoxy-3*α*,5-cyclocholest-25-en-22-ol (S4-(22*S*))

(22*R*)-24-nor-6*β*-Methoxy-3*α*,5-cyclocholest-25-en-22-ol (S4-(22*R*))

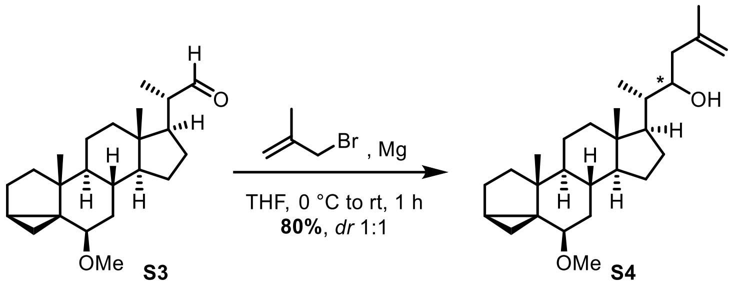

S4 was synthesized according to the procedure published by Nemoto and co-workers[16].

To a stirred suspension of **S3** (1.41 g, 4.10 mmol, 1.0 equiv.) and Mg powder (499 mg, 20.5 mmol, 5.0 equiv.) in THF (4 mL) was added 3-bromo-2-methylprop-1-ene (1.65 mL, 16.4 mmol, 4.0 equiv.) at 0 °C. After stirring at this temperature for 1 h, the reaction mixture was diluted with CH_2Cl_2 (10 mL) and NH_4Cl (sat., 10 mL) was added. The aqueous layer was extracted with CH_2Cl_2 (3× 20 mL) and the combined organic phases were dried over $MgSO_4$ and concentrated under reduced pressure. The residue was adsorbed on silica gel and the diastereomers were separated by automated flash column chromatography (silica gel, PE/EtOAc 100:0 to 90:10) to give alcohols **S4** (1.31 g, 3.28 mmol, 80%, *dr* 1:1) as clear oils.

S4-(22*S*):

TLC R_f = 0.41 (PE/EtOAc 8:2, CAM).

^{1}H-NMR (400 MHz, $CDCl_3$): δ [ppm] = 4.82 (dt, J = 2.9, 1.4 Hz, 1H), 4.78–4.73 (m, 1H), 3.81 (ddd, J = 9.2, 4.4, 1.6 Hz, 1H), 3.31 (s, 3H), 2.75 (t, J = 2.9 Hz, 1H), 2.24 (dd, J = 13.6, 9.1 Hz, 1H), 2.06–1.81 (m, 4H), 1.78–1.57 (m, 6H, contains 1.73 (s, 3H)), 1.54–1.44 (m, 3H), 1.43–1.26 (m, 5H), 1.24–1.01 (m, 7H, contains 1.01 (s, 3H)), 0.91 (d, J = 6.6 Hz, 3H), 0.89–0.76 (m, 3H), 0.71 (s, 3H), 0.66–0.60 (m, 1H), 0.41 (dd, J = 8.1, 5.1 Hz, 1H).

^{13}C-NMR (101 MHz, $CDCl_3$): δ [ppm] = 143.3, 113.1, 82.5, 70.3, 56.7, 56.5, 53.0, 48.1, 44.2, 43.5, 42.8, 40.38, 40.36, 35.3, 35.2, 33.5, 30.7, 27.9, 25.1, 24.2, 22.9, 22.6, 21.6, 19.4, 13.2, 12.2, 11.9.

opt. act. $[\alpha]_D^{23}$ = +36.3 (c=0.91, $CHCl_3$).

HRMS (ESI): *m/z* $[M+Na]^+$ calcd. for $[C_{27}H_{44}O_2Na]^+$: 423.3234; found: 423.3226.

S4-(22*R*):

TLC R_f = 0.36 (PE/EtOAc 8:2, CAM).

^{1}H-NMR (400 MHz, $CDCl_3$): δ [ppm] = 4.87–489 (m, 1H), 4.83–4.78 (m, 1H), 3.77 (ddd, J = 10.8, 3.5, 2.1 Hz, 1H), 3.32 (s, 3H), 2.76 (t, J = 2.9 Hz, 1H), 2.11–2.04 (m, 1H), 2.02–1.93 (m, 2H), 1.89 (dt, J = 13.7, 3.2 Hz, 1H), 1.83 (td, J = 7.0, 3.4 Hz, 1H), 1.79–1.68 (m, 6H, contains 1.76 (s, 3H)), 1.67–1.58 (m, 2H), 1.55–1.46 (m, 2H), 1.46–1.34 (m, 3H), 1.26–0.99 (m, 8H, contains 1.02 (s, 3H)), 0.93 (d, J = 6.7 Hz, 3H), 0.91–0.79 (m, 3H), 0.75 (s, 3H), 0.68–0.61 (m, 1H), 0.43 (dd, J = 8.0, 5.1 Hz, 1H).

^{13}C-NMR (101 MHz, $CDCl_3$): δ [ppm] = 143.6, 113.5, 82.5, 69.8, 56.7, 56.3, 53.4, 48.2, 43.5, 43.3, 40.7, 40.4, 38.9, 35.4, 35.2, 33.5, 30.7, 27.6, 25.1, 24.4, 22.9, 22.3, 21.6, 19.4, 13.2, 12.5, 12.4.

opt. act. $[\alpha]_D^{23}$ = +44.7 (c=0.94, $CHCl_3$).

HRMS (ESI): *m/z* $[M+Na]^+$ calcd. for $[C_{27}H_{44}O_2Na]^+$: 423.3234; found: 423.3232.
IR ṽ $[cm^{-1}]$ = 2934 (s), 2866 (m), 1456 (m), 1443 (w), 1373 (m), 1097 (s), 1082 (s), 1057 (s), 1016 (m), 999 (w), 968 (w), 891 (m), 883 (m), 613 (w), 534 (w).

(22*S*)-24-nor-6*β*-Methoxy-3*α*,5-cyclocholest-25-ene-22-methacrylate (S5-(22*S*))

(22*R*)-24-nor-6*β*-Methoxy-3*α*,5-cyclocholest-25-ene-22-methacrylate (S5-(22*R*))

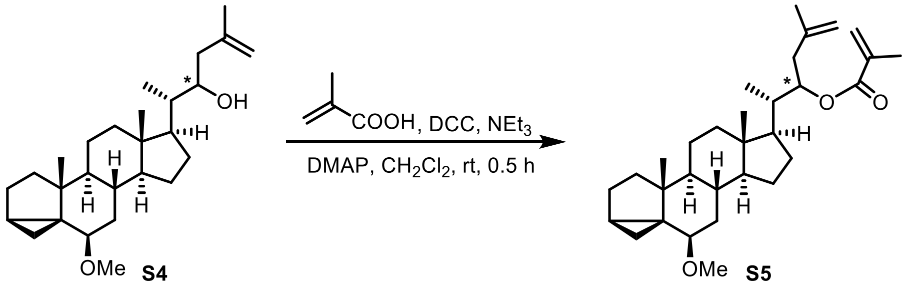

To a stirred solution of alcohol **S4-(22*S*)** (792 mg, 1.98 mmol, 1.0 equiv.) in CH_2Cl_2 (20 mL) were added methacrylic acid (0.84 mL, 9.89 mmol, 5.0 equiv.), *N,N′*-dicyclohexyl carbodiimide (2.04 g, 9.89 mmol, 5.0 equiv.), triethylamine (1.38 mL, 9.89 mmol, 5.0 equiv.) and *N,N*-dimethylpyridin-4-amine (24.2 mg, 0.20 mmol, 0.1 equiv.). After stirring for 30 min, the mixture was diluted with CH_2Cl_2 (20 mL), washed with HCl (aq., 10% *v*/*v*, 30 mL) and $NaHCO_3$ (sat. aq., 30 mL) and then was dried over $MgSO_4$. The solvent was removed under reduced pressure, the residue was adsorbed on silica gel and automated flash column chromatography (silica gel, PE/EtOAc 100:0 to 75:25) gave ester **S5-(22*S*)** (885 mg, 1.89 mmol, 96%) as a white amorphous solid.

S5-(22*S*):

TLC R_f = 0.56 (PE/EtOAc 8:2, UV, CAM).
^{1}H-NMR (400 MHz, $CDCl_3$): *δ* [ppm] = 6.10–6.05 (m, 1H), 5.52–5.48 (m, 1H), 5.21 (td, *J* = 7.1, 1.4 Hz, 1H), 4.79–4.73 (m, 1H), 4.73–4.68 (m, 1H), 3.31 (s, 3H), 2.76 (t, *J* = 2.9 Hz, 1H), 2.37 (dd, *J* = 13.5, 7.3 Hz, 1H), 2.19 (dd, *J* = 13.5, 6.8 Hz, 1H), 2.03–1.92 (m, 5H, contains 1.93 (s, 3H)), 1.88 (dt, *J* = 13.5, 3.1 Hz, 1H), 1.77 (s, 3H), 1.75–1.67 (m, 2H), 1.66–1.55 (m, 2H), 1.50 (td, *J* = 12.1, 7.7 Hz, 2H), 1.43–1.09 (m, 5H), 1.09–0.98 (m, 8H, contains 1.05 (d, *J*=6.9 Hz, 1H), 1.01 (s, 3H)), 0.92–0.75 (m, 3H), 0.72 (s, 3H), 0.67–0.60 (m, 1H), 0.42 (dd, *J* = 8.0, 5.1 Hz, 1H).
^{13}C-NMR (101 MHz, $CDCl_3$): δ [ppm] = 167.1, 142.0, 137.0, 125.1, 113.5, 82.5, 74.2, 56.7, 56.5, 53.0, 48.1, 43.5, 42.9, 40.9, 40.4, 39.0, 35.4, 35.1, 33.5, 30.7, 28.4, 25.1, 24.3, 22.9, 22.4, 21.6, 19.4, 18.6, 13.2, 12.9, 12.2.
opt. act. $[\alpha]_D^{23}$ = +21.9 (c=1.11, $CHCl_3$).
HRMS (ESI): *m/z* $[M+Na]^+$ calcd. for $[C_{31}H_{48}O_3Na]^+$: 491.3496; found: 491.3501.
IR ṽ $[cm^{-1}]$ = 2951 (w), 2928 (w), 2909 (w), 2870 (w), 1709 (s), 1447 (w), 1315 (m), 1292 (m), 1159 (s), 1098 (s), 1084 (m), 1059 (w), 1015 (w), 970 (w), 941 (m), 887 (m), 810 (w).

S5-(22*R*): starting with 755 mg (1.88 mmol) of **S5-(22*R*)** resulted in a clear oil (714 mg, 1.52 mmol, 81%).
TLC R_f = 0.56 (PE/EtOAc 8:2, UV, CAM).
^{1}H-NMR (400 MHz, $CDCl_3$): *δ* [ppm] = 6.08 (s, 1H), 5.50 (q, *J* = 1.7 Hz, 1H), 5.16 (dt, *J* = 10.9, 2.2 Hz, 1H), 4.73 (s, 1H), 4.69 (s, 1H), 3.32 (d, *J* = 1.3 Hz, 3H), 2.77 (d, *J* = 2.9 Hz, 1H), 2.24 (dd, *J* = 14.4, 11.0 Hz, 1H), 2.12 (d, *J* = 14.3 Hz, 1H), 1.99–1.80 (m, 7H, contains 1.92 (s, 3H))), 1.80–1.69 (m, 5H, contains 1.72 (s, 3H)), 1.68–1.57 (m, 2H), 1.56–1.45 (m, 2H), 1.45–1.35 (m, 2H), 1.27–1.11 (m, 3H), 1.10–1.03 (m, 2H), 1.01 (s, 3H), 0.96 (d, *J* = 6.7 Hz, 3H), 0.92–0.77 (m, 3H), 0.73 (s, 3H), 0.64 (t, *J* = 4.4 Hz, 1H), 0.43 (dd, *J* = 8.1, 5.1 Hz, 1H).

^{13}C-NMR (101 MHz, $CDCl_3$): δ [ppm] = 167.0, 142.5, 137.0, 125.0, 112.7, 82.5, 74.7, 56.7, 56.3, 53.4, 48.2, 43.5, 43.3, 40.4, 39.3, 36.0, 35.4, 35.2, 33.5, 30.6, 27.3, 25.1, 24.4, 22.9, 22.5, 21.6, 19.4, 18.5, 13.2, 13.2, 12.5.
opt. act. $[\alpha]_D^{23}$ = +40.8 (c=0.98, $CHCl_3$).
HRMS (ESI): *m/z* $[M+Na]^+$ calcd. for $[C_{31}H_{48}O_3Na]^+$: 491.3496; found: 491.3501.

(22*S*)-6*β*-Methoxy-3*α*,5-cycloergosta-24,25-diene-26,22-lactone (S6-(22*S*))

(22*R*)-6*β*-Methoxy-3*α*,5-cycloergosta-24,25-diene-26,22-lactone (S6-(22*R*))

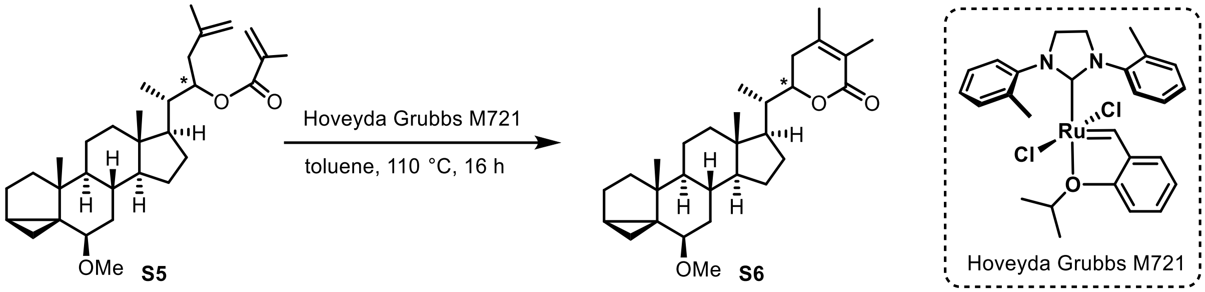

S6 was synthesized according to the procedure published by Nemoto and co-workers[16].

A solution of ester **S5-(22*S*)** (694 mg, 1.48 mmol, 1.0 equiv.) and catalyst M721 (84.6 mg, 0.15 mmol, 0.1 equiv.) in toluene (148 mL) was stirred for 16 h at 110 °C. The solvent was removed under reduced pressure and automated flash column chromatography (silica gel, PE/EtOAc 100:0 to 65:35) gave lactone **S6-(22*S*)** (105 mg, 0.24 mmol, 16%, 52% brsm) as colourless crystalline solid.

S6-(22*S*):

TLC R_f = 0.31 (PE/EtOAc 8:2, UV, CAM).
m. p. 128–130 °C.
^{1}H-NMR (400 MHz, $CDCl_3$): δ [ppm] = 4.40 (dd, *J* = 12.4, 3.1 Hz, 1H), 3.31 (d, *J* = 1.3 Hz, 3H), 2.76 (d, *J* = 2.9 Hz, 1H), 2.63 (t, *J* = 15.4 Hz, 1H), 1.97–1.81 (m, 9H, contains 1.92 (s, 3H), 1.87 (s, 3H)), 1.80–1.58 (m, 5H), 1.55–1.46 (m, 3H), 1.44–1.34 (m, 2H), 1.28–1.06 (m, 4H), 1.04–1.00 (m, 6H, contains 1.02 (d, *J* = 9.8 Hz, 3H), 1.01 (s, 3H)), 0.94–0.77 (m, 4H), 0.71 (s, 3H), 0.64 (t, *J* = 4.5 Hz, 1H), 0.42 (dd, *J* = 8.1, 5.1 Hz, 1H).
^{13}C-NMR (101 MHz, $CDCl_3$): δ [ppm] = 167.3, 149.6, 122.0, 82.5, 78.4, 56.7, 56.3, 51.5, 48.0, 43.5, 42.8, 40.1, 39.6, 35.4, 35.1, 34.1, 33.5, 30.7, 27.9, 25.1, 24.2, 22.9, 21.6, 20.5, 19.4, 13.3, 13.2, 12.7, 12.3.
opt. act. $[\alpha]_D^{23}$ = +16.5 (c=1.15, $CHCl_3$).
HRMS (ESI): *m/z* $[M+Na]^+$ calcd. for $[C_{29}H_{44}O_3Na]^+$: 463.3183; found: 463.3170.

The absolute stereoconfiguration of **S6-(22*S*)** was determined by X-ray crystallography. A suitable crystal 0.40×0.29×0.04 mm^3 was selected and mounted on an 18 mm CryoLoop (20 micron, 0.2 - 0.3 mm, Hampton Research) on an XtaLAB AFC12 (RINC): Kappa single diffractometer. The crystal was kept at a steady temperature during data collection. The structure was solved with the ShelXT 2018/2 [17] structure solution program using the Intrinsic Phasing solution method and by using Olex2 [18] as the graphical interface. The model was refined with version 2019/3 of ShelXL 2019/3 [19] using Least Squares minimisation. X-ray crystallographic data is shown in Supplementary Table 18.

S6-(22*R*): starting with 675 mg (1.44 mmol) of **S5-(22*R*)** resulted in a yellowish amorphous solid (107 mg, 0.24 mmol, 17%, 54% brsm).

TLC R_f = 0.30 (PE/EtOAc 8:2, UV, CAM).

^{1}H-NMR (400 MHz, $CDCl_3$): δ [ppm] = 4.37 (dt, *J* = 13.4, 3.5 Hz, 1H), 3.31 (d, *J* = 1.1 Hz, 3H), 2.76 (t, *J* = 3.0 Hz, 1H), 2.43 (t, *J* = 15.5 Hz, 1H), 2.04–1.83 (m, 10H, contains 1.93 (s, 3H), 1.87 (s, 3H)), 1.80–1.57 (m, 4H), 1.56–1.45 (m, 2H), 1.44–1.31 (m, 3H), 1.27–0.97 (m, 11H, contains 1.02 (s, 3H), 1.00 (s, 3H)), 0.93–0.76 (m, 3H), 0.74 (s, 3H), 0.65 (t, *J* = 4.5 Hz, 1H), 0.43 (dd, *J* = 8.1, 5.1 Hz, 1H).

^{13}C-NMR (101 MHz, $CDCl_3$): δ [ppm] = 167.2, 149.1, 122.1, 82.4, 78.5, 56.7, 56.3, 52.4, 48.2, 43.5, 43.3, 40.3, 39.0, 35.4, 35.2, 33.5, 30.7, 29.7, 27.6, 25.1, 24.4, 22.9, 21.6, 20.7, 19.4, 13.6, 13.2, 12.6, 12.2.

opt. act. $[\alpha]_D^{23}$ = +107.1 (c=1.13, $CHCl_3$).

HRMS (ESI): *m/z* $[M+Na]^+$ calcd. for $[C_{29}H_{44}O_3Na]^+$: 463.3183; found: 463.3184.

IR ṽ [cm^{-1}] = 2930 (m), 2864 (w), 1705 (s), 1395 (m), 1379 (m), 1315 (w), 1184 (m), 1146 (w), 1125 (s), 1098 (s), 1082 (m), 1016 (m), 995 (w), 970 (w), 762 (m).

(22*S*)- 3*β*-Hydroxyergosta-5,24-diene-26,22-lactone (S7-(22*S*))

(22*R*)- 3*β*-Hydroxyergosta-5,24-diene-26,22-lactone (S7-(22*R*))

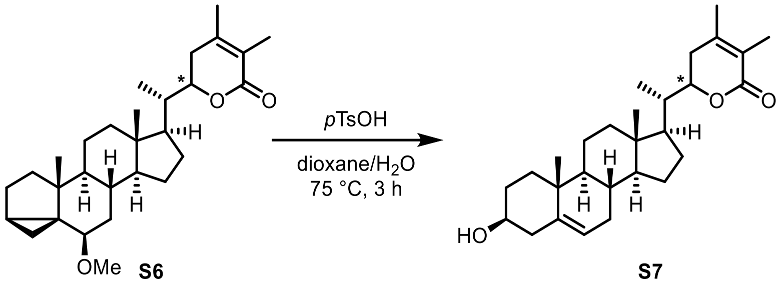

S7 was synthesized according to the procedure published by Kang and co-workers[20].

Unsaturated lactone **S6-(22*S*)** (19.5 mg, 44.3 μmol, 1.0 equiv.) was dissolved in 1,4dioxane (0.75 mL) and *p*TsOH (aq., 0.04 M, 0.15 mL) was added. After stirring at 75 °C for 3 h, the mixture was diluted with CH_2Cl_2 (5 mL) and H_2O (2 mL). The aqueous layer was extracted with CH_2Cl_2 (3× 5 mL), the combined organic phases were dried over $MgSO_4$ and concentrated under reduced pressure. The residue was adsorbed on silica gel and column chromatography (silica gel, PE/EtOAc 50:50) gave alcohol **S7** (17.5 mg, 41.0 μmol, 93%) as colourless solid.

S7-(22*S*):

TLC R_f = 0.20 (PE/EtOAc 6:4, UV, CAM).

m. p. 173–176 °C.

^{1}H-NMR (600 MHz, MeOD): δ [ppm] = 5.35 (dt, *J* = 4.7, 2.0 Hz, 1H), 4.45 (ddd, *J* = 13.3, 3.6, 1.3 Hz, 1H), 3.44–3.36 (m, 1H), 2.71–2.63 (m, 1H), 2.28–2.17 (m, 2H), 2.08–1.76 (m, 12H, contains 1.98 (s, 3H), 1.86 (s, 3H)), 1.70–1.44 (m, 8H), 1.43–1.34 (m, 1H), 1.26 (td, *J* = 12.7, 4.3 Hz, 1H), 1.19–1.07 (m, 3H), 1.05 (d, *J* = 6.3 Hz, 3H), 1.03 (s, 3H), 0.97 (ddd, *J* = 12.2, 10.8, 5.0 Hz, 1H), 0.75 (s, 3H).

^{13}C-NMR (151 MHz, MeOD): δ [ppm] = 169.5, 153.3, 142.2, 122.4, 121.9, 80.2, 72.4, 58.0, 53.1, 51.7, 43.4, 43.0, 41.1, 40.5, 38.5, 37.7, 34.6, 33.3, 33.0, 32.3, 28.5, 25.2, 22.2, 20.3, 19.9, 13.7, 12.5, 12.1.
opt. act. $[\alpha]_D^{23}$ = –43.9 (c=1.20, $CHCl_3$).
HRMS (ESI): *m/z* $[M+Na]^+$ calcd. for $[C_{28}H_{42}O_3Na]^+$: 449.3026; found: 449.3032.
IR ṽ [cm^{-1}] = 2930 (m), 1694 (s), 1383 (w), 1146 (s), 1146 (s), 1092 (m), 1059 (s), 1022 (m), 1011 (m), 799 (m), 762 (w), 511 (w), 405 (w).

S7-(22*R*): starting with 28.3 mg (64.2 µmol) of **S6-(22*R*)** resulted in a colourless solid (26.8 mg, 62.8 µmol, 98%).
TLC R_f = 0.20 (PE/EtOAc 6:4, UV, CAM).
m. p. 200–203 °C.
^{1}H-NMR (600 MHz, MeOD): *δ* [ppm] = 5.35 (dt, *J* = 5.7, 1.9 Hz, 1H), 4.41 (ddd, *J* = 13.4, 3.6, 3.6 Hz, 1H), 3.43–3.35 (m, 1H), 2.52–2.43 (m, 1H), 2.28–2.17 (m, 2H), 2.11 (dd, *J* = 18.0, 3.5 Hz, 1H), 2.06 (dt, *J* = 12.7, 3.5 Hz, 1H), 2.03–1.91 (m, 4H, contains 1.99 (s, 3H)), 1.91–1.86 (m, 1H), 1.86–1.83 (m, 3H), 1.82–1.74 (m, 2H), 1.70–1.63 (m, 1H), 1.62–1.45 (m, 5H), 1.44–1.36 (m, 1H), 1.28–1.13 (m, 3H), 1.12–1.05 (m, 2H), 1.05–1.03 (m, 7H, contains 1.04 (s, 3H), 1.03 (d, *J* = 6.6 Hz, 3H)), 0.97 (ddd, *J* = 12.0, 10.6, 5.1 Hz, 1H), 0.78 (s, 3H).
^{13}C-NMR (151 MHz, MeOD): δ [ppm] = 169.5, 152.7, 142.3, 122.3, 122.2, 80.2, 72.4, 57.7, 53.2, 51.7, 43.9, 43.0, 41.0, 40.4, 38.6, 37.7, 33.3, 33.0, 32.3, 30.4, 28.3, 25.4, 22.2, 20.4, 19.9, 13.8, 12.4, 12.1.
opt. act. $[\alpha]_D^{23}$ = +41.2 (c=0.98, $CHCl_3$).
HRMS (ESI): *m/z* $[M+Na]^+$ calcd. for $[C_{28}H_{42}O_3Na]^+$: 449.3026; found: 449.3015.

(22*S*)- 6*β*-Methoxy-3*α*,5-cycloergost-24-ene-22,26-diol (S8-(22*S*))

(22*R*)- 6*β*-Methoxy-3*α*,5-cycloergost-24-ene-22,26-diol (S8-(22*R*))

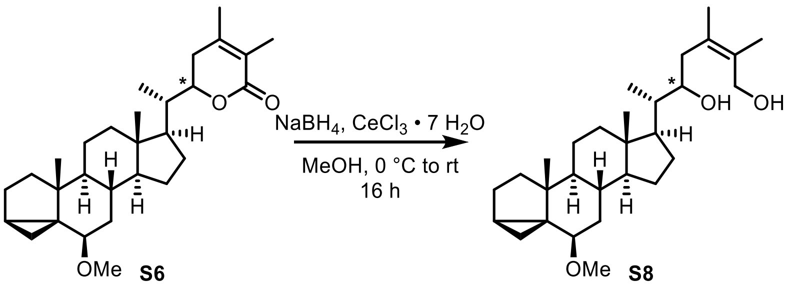

To a solution of unsaturated lactone **S6-(22*S*)** (36.0 mg, 82 µmol, 1.0 equiv.) in MeOH (0.16 mL) was added cerium trichloride heptahydrate (60.9 mg, 163 µmol, 2.0 equiv.) at 0 °C. After 1 h sodium borohydride (6.2 mg, 163 µmol, 2.0 equiv.) was added. After 15 min the ice bath was removed and the solution was allowed to reach ambient temperature while being stirred for 16 h before adding EtOAc (5 mL) and hydrogen chloride (aq., 1 M, 5 mL). The aqueous layer was extracted with EtOAc (3× 10 mL), the combined organic phases were dried over Na_2SO_4 and concentrated under reduced pressure. The residue was adsorbed on silica gel and column chromatography (silica gel, PE/EtOAc 50:50) gave diol **S8-(22*S*)** (27.2 mg, 61.3 µmol, 75%) as colourless crystalline solid.

S8-(22*S*):

TLC R_f = 0.24 (PE/EtOAc 7:3, CAM).

m. p. 103–107 °C.

^{1}H-NMR (400 MHz, $CDCl_3$): δ [ppm] = 4.32 (d, J = 11.3 Hz, 1H), 3.73 (m, 2H), 3.32 (s, 3H), 2.80–2.69 (m, 2H), 2.56 (bs, 1H), 1.98 (dt, J = 12.5, 3.4 Hz, 1H), 1.94–1.83 (m, 2H), 1.78–1.66 (m, 9H, contains 1.80 (s, 3H), 1.70 (s, 3H)), 1.66–1.59 (m, 1H), 1.57–1.47 (m, 2H), 1.45–1.29 (m, 5H), 1.28–1.00 (m, 7H, contains 1.02 (s, 3H)), 0.98–0.93 (m, 3H), 0.92–0.77 (m, 3H), 0.73 (s, 3H), 0.64 (dd, J = 5.1, 3.8 Hz, 1H), 0.43 (dd, J = 8.0, 5.1 Hz, 1H).

^{13}C-NMR (101 MHz, $CDCl_3$): δ [ppm] = 131.5, 131.0, 82.5, 70.6, 63.6, 56.7, 56.5, 53.2, 48.1, 43.5, 42.9, 42.1, 40.4, 40.3, 35.3, 35.2, 33.5, 30.7, 27.9, 25.1, 24.3, 22.9, 21.6, 19.4, 19.2, 18.6, 13.2, 12.3, 12.3.

opt. act. $[\alpha]_D^{23}$ = +34.0 (c=0.99, $CHCl_3$).

HRMS (ESI): *m/z* $[M+Na]^+$ calcd. for $[C_{29}H_{48}O_3Na]^+$: 467.3496; found: 467.3509.

IR ṽ [cm^{-1}] = 2936 (m), 2905 (w), 2864 (m), 1456 (w), 1381 (w), 1098 (s), 1086 (m), 1011 (m), 1001 (s), 970 (w).

S8-(22*R*): starting with 40.0 mg (91 μmol) of **S6-(22*R*)** resulted in a white crystalline solid (29.6 mg, 66.5 μmol, 73%).

TLC R_f = 0.18 (PE/EtOAc 7:3, CAM).

m. p. 200–202 °C.

^{1}H-NMR (400 MHz, $CDCl_3$): δ [ppm] = 4.35 (d, J = 11.2 Hz, 1H), 3.76 (m, 2H), 3.34 (s, 3H), 2.79 (t, J = 2.9 Hz, 1H), 2.52 (dd, J = 13.3, 10.9 Hz, 1H), 2.02 (dt, J = 12.6, 3.4 Hz, 1H), 1.92 (dt, J = 13.4, 3.1 Hz, 1H), 1.86–1.70 (m, 12H, contains 1.83 (s, 3H), 1.72 (s, 3H)), 1.70–1.61 (m, 1H), 1.55 (t, J = 6.3 Hz, 1H), 1.52 (t, J = 6.7 Hz, 1H), 1.48–1.35 (m, 3H), 1.28–1.07 (m, 5H), 1.04 (s, 3H), 1.00 (d, J = 6.7 Hz, 3H), 0.94–0.81 (m, 3H), 0.77 (s, 3H), 0.67 (dd, J = 5.1, 3.8 Hz, 1H), 0.45 (dd, J = 8.0, 5.1 Hz, 1H).

^{13}C-NMR (101 MHz, $CDCl_3$): δ [ppm] = 131.9, 130.7, 82.5, 70.1, 63.6, 56.7, 56.3, 53.4, 48.2, 43.5, 43.3, 42.5, 40.4, 35.4, 35.2, 34.4, 33.5, 30.7, 27.7, 25.1, 24.5, 22.9, 21.6, 19.4, 19.0, 18.7, 13.2, 12.4, 12.4.

opt. act. $[\alpha]_D^{23}$ = (c=, $CHCl_3$).

HRMS (ESI): *m/z* $[M+Na]^+$ calcd. for $[C_{29}H_{48}O_3Na]^+$: 467.3496; found: 467.3493.

(22*S*)-Ergosta-5,24-diene-3β,22,26-triol (S9-(22*S*))

(22*R*)-Ergosta-5,24-diene-3β,22,26-triol (S9-(22*R*))

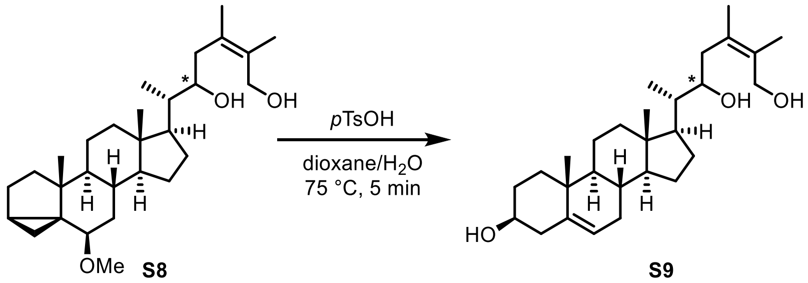

S9 was synthesized according to a modified procedure published by Kang and co-workers[20].

Unsaturated diol **S8-(22*S*)** (14.8 mg, 33.3 μmol, 1.0 equiv.) was dissolved in 1,4-dioxane (0.45 mL) and 0.15 mL of a *p*TsOH stock solution (aq., 0.03 M, 0.15 equiv.) was added. After stirring at 75 °C for 5 min (longer reaction time resulted in decomposition), the mixture was diluted with EtOAc (5 mL) and H_2O (2 mL). The aqueous layer was extracted with EtOAc (3× 5 mL), the combined organic phases were dried

over Na_2SO_4 and concentrated under reduced pressure. The residue was adsorbed on silica gel and column chromatography (silica gel, PE/EtOAc 50:50 to 30:70) gave triol **S9-(22*S*)** (7.8 mg, 33.3 µmol, 54%) as colourless solid.

S9-(22*S*):

TLC R_f = 0.21 (PE/EtOAc 1:1, CAM).
m. p. 164–167 °C.
^{1}H-NMR (600 MHz, MeOD): δ [ppm] = 5.34 (dd, *J* = 4.6, 2.4 Hz, 1H), 4.16 (d, *J* = 11.5 Hz, 1H), 3.93 (d, *J* = 11.5 Hz, 1H), 3.74 (ddd, *J* = 8.8, 4.6, 1.4 Hz, 1H), 3.43–3.33 (m, 1H), 2.53 (dd, *J* = 13.5, 8.7 Hz, 1H), 2.27–2.17 (m, 2H), 2.07–1.91 (m, 4H), 1.87 (dt, *J* = 13.3, 3.5 Hz, 1H), 1.83–1.75 (m, 4H, contains 1.76 (s, 3H)), 1.72 (s, 3H), 1.69–1.59 (m, 1H), 1.59–1.45 (m, 6H), 1.43–1.36 (m, 1H), 1.36–1.28 (m, 3H), 1.28–1.19 (m, 1H), 1.18–1.05 (m, 3H), 1.03 (s, 3H), 0.99–0.87 (m, 5H, contains 0.97 (d, *J* = 6.7 Hz, 3H)), 0.73 (s, 3H).
^{13}C-NMR (151 MHz, MeOD): δ [ppm] = 142.2, 131.6, 131.3, 122.4, 72.4, 72.2, 63.6, 58.2, 54.1, 51.7, 43.4, 43.0, 42.4, 41.2, 40.9, 38.6, 37.7, 33.3, 33.0, 32.3, 28.9, 25.3, 22.2, 19.9, 19.4, 17.6, 12.7, 12.2.
opt. act. $[\alpha]_D^{23}$ = –30.7 (c=0.25, MeOH).
HRMS (ESI): *m/z* $[M+Na]^+$ calcd. for $[C_{28}H_{46}O_3Na]^+$: 453.3339; found: 453.3326.
IR ṽ [cm^{-1}] = 2963 (m), 2926 (s), 2905 (m), 2879 (m), 2862 (m), 2851 (w), 1038 (s), 1022 (s), 1003 (s), 982 (s), 957 (m), 849 (w), 528 (m), 486 (w), 459 (w).

S9-(22*R*): starting with (8.5 mg, 19 mmol) of **S8-(22*R*)** resulted in a colourless solid (5.6 mg, 13 mmol, 68%).
TLC R_f = 0.20 (PE/EtOAc 1:1, CAM).
m. p. 135–140 °C.
^{1}H-NMR (600 MHz, MeOD): δ [ppm] = 5.35 (dt, *J* = 5.5, 1.9 Hz, 1H), 4.19 (d, *J* = 11.4 Hz, 1H), 3.85 (d, *J* = 11.5 Hz, 1H), 3.72 (ddd, *J* = 10.8, 3.3, 1.9 Hz, 1H), 3.44–3.35 (m, 1H), 2.44 (dd, *J* = 13.7, 10.8 Hz, 1H), 2.28–2.17 (m, 2H), 2.06 (dt, *J* = 12.6, 3.5 Hz, 1H), 2.02–1.96 (m, 1H), 1.92–1.83 (m, 3H), 1.82–1.74 (m, 4H, contains 1.78 (s, 3H)), 1.72 (s, 3H), 1.70–1.63 (m, 1H), 1.60–1.39 (m, 7H), 1.27–1.05 (m, 5H), 1.03 (s, 3H), 0.99 (d, *J* = 6.8 Hz, 3H), 0.98–0.85 (m, 1H), 0.76 (s, 3H).
^{13}C-NMR (151 MHz, MeOD): δ [ppm] = 142.3, 132.0, 131.6, 122.4, 72.4, 71.3, 63.7, 57.8, 54.5, 51.7, 43.8, 43.7, 43.0, 41.1, 38.6, 37.7, 35.0, 33.3, 33.0, 32.3, 28.7, 25.5, 22.2, 19.9, 19.1, 18.2, 12.8, 12.3.
opt. act. $[\alpha]_D^{23}$ = –9.6 (c=0.52, MeOH).
HRMS (ESI): *m/z* $[M+Na]^+$ calcd. for $[C_{28}H_{46}O_3Na]^+$: 453.3339; found: 453.3327.

(22*R*)-Ergosta-5,24-diene-1α,3β,22,26-tetrol (17))

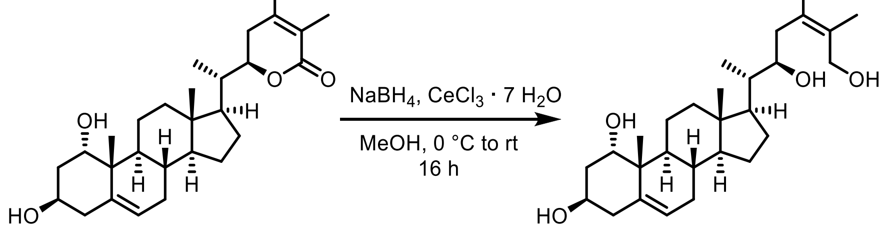

withanoside V aglycone (16) **17**

To a solution of withanoside V aglycone **(16)** (4.1 mg, 9.3 µmol, 1.0 equiv.) in MeOH (0.19 mL) was added cerium trichloride heptahydrate (10.2 mg, 27.4 µmol, 3.0 equiv.) at 0 °C. After 1 h sodium borohydride (2.1 mg, 56 µmol, 6.0 equiv.) was added. After 15 min the ice bath was removed and the solution was allowed to reach ambient temperature while being stirred for 16 h before adding EtOAc (5 mL) and hydrogen chloride (aq., 1 M, 5 mL). The aqueous layer was extracted with EtOAc (3× 10 mL), the combined organic phases were dried over Na_2SO_4 and concentrated under reduced pressure. The residue was adsorbed on silica gel and column chromatography (silica gel, EtOAc) gave tetrol **17** (2.0 mg, 4.5 µmol, 48%) and withanoside V aglycone **(16)** (2.1 mg, 4.7 µmol, 51%) as colourless crystalline solids.

17:

TLC R_f = 0.25 (EtOAc, CAM).

^{1}H-NMR (500 MHz, MeOD): *δ* [ppm] = 5.52–5.47 (m, 1H), 4.19 (d, *J* = 11.4 Hz, 1H), 3.94–3.84 (m, 4H, contains 3.86 (d, *J* = 11.6 Hz, 1H)f), 3.80 (t, *J* = 2.9 Hz, 1H), 3.72 (ddd, *J* = 10.8, 3.3, 1.9 Hz, 1H), 2.44 (dd, *J* = 13.7, 10.8 Hz, 1H), 2.33–2.20 (m, 2H), 2.08–1.82 (m, 6H), 1.78 (s, 3H), 1.76–1.64 (m, 6H, contains 1.72 (s, 3H)), 1.63–1.37 (m, 6H), 1.27–1.06 (m, 5H), 1.02 (s, 3H), 0.99 (d, *J* = 6.8 Hz, 3H), 0.76 (s, H).

^{13}C-NMR (126 MHz, MeOD): δ [ppm] = 139.4, 132.0, 131.6, 125.2, 73.6, 71.3, 67.0, 63.7, 57.8, 54.5, 43.9, 43.7, 42.7, 42.6, 42.5, 41.0, 39.2, 35.0, 33.3, 32.9, 28.7, 25.6, 21.3, 20.1, 19.1, 18.2, 12.8, 12.3..

HRMS (ESI): *m/z* $[M+Na]^+$ calcd. for $[C_{28}H_{46}O_4Na]^+$: 469.3288; found: 469.3293.

Supplementary Method 4. Virus-induced gene silencing in *N. benthamiana*

Virus-induced gene silencing (VIGS) in *N. benthamiana* was performed following a previously published protocol[11]. Target gene fragments of *NbDWF1* and *NbPDS* (500-600 bp) were designed using the SGN VIGS Tool[12]. Fragments were amplified from *N. benthamiana* leaf cDNA (primers in Supplementary Data 4) and cloned into pTRV2[11] by restriction digestion with EcoRI and XhoI. The resulting plasmid was transformed into *E. coli* Top10 cells, isolated and its sequence confirmed by Sanger sequencing. To enable *Agrobacterium*-mediated gene transfer into *N. benthamiana*, either pTRV1, pTRV2 or pTRV2-gene of interest (GOI) were transformed into *Agrobacterium tumefaciens* GV3101 by electroporation.

Agrobacterium strains for VIGS were grown and harvested as described in section "Transient expression in *Nicotiana benthamiana*". All strains were diluted in infiltration buffer to an OD_{600} of 1.0 and strains containing pTRV1 and pTRV2/pTRV2-GOI were mixed in a 1:1 ratio. The mixture was then infiltrated into the leaves of three-week-old *N. benthamiana* plants. These were maintained in a phytochamber for two further weeks until symptoms of photobleaching were clearly visible in *PDS*-silenced control plants.
After these two weeks post inoculation, leaves that had newly emerged were infiltrated with *Agrobacterium tumefaciens* GV3101 containing the plasmid pHREAC-*Pper24ISO* for transient expression. After five days of transient expression in *DWF1*-silenced plants, leaves were harvested and analysed by GC-MS as described. Representative results are shown in Supplementary Fig. 12.

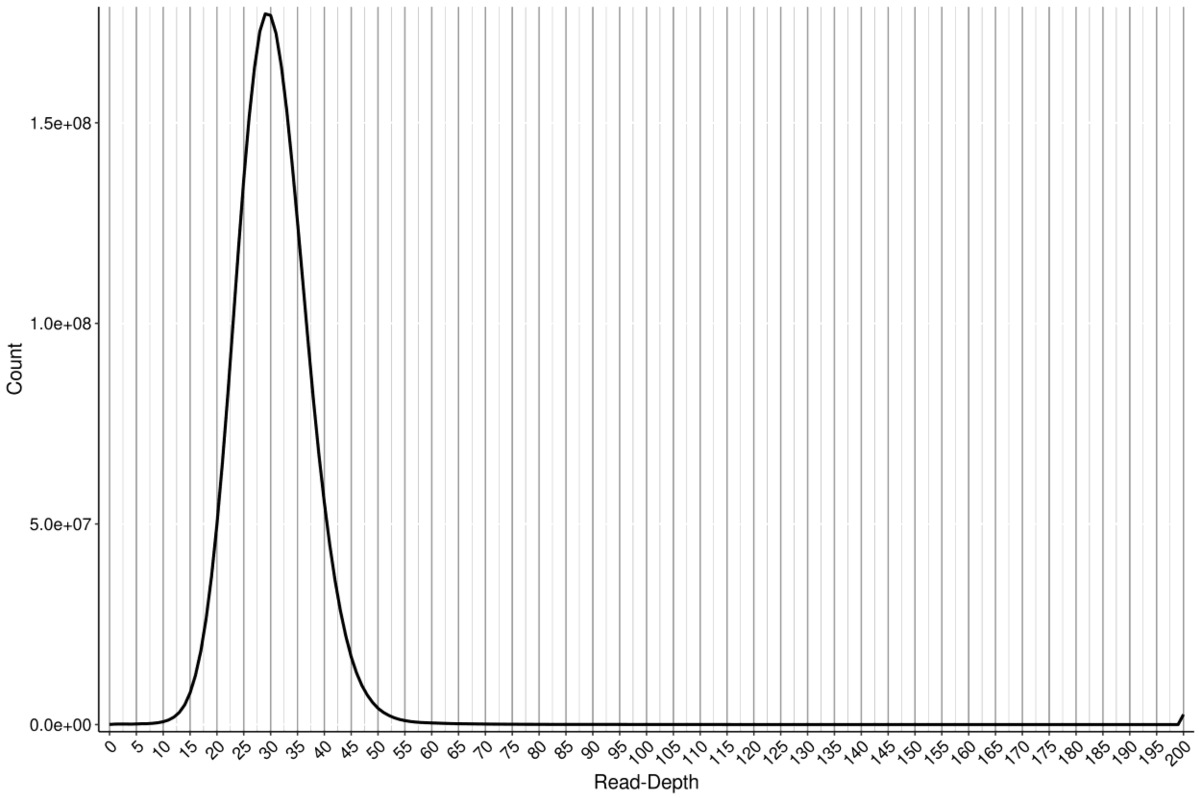

Supplementary Fig. 1. *W. somnifera* read coverage depth histogram with a unimodal distribution suggesting a diploid genome with low heterozygosity.

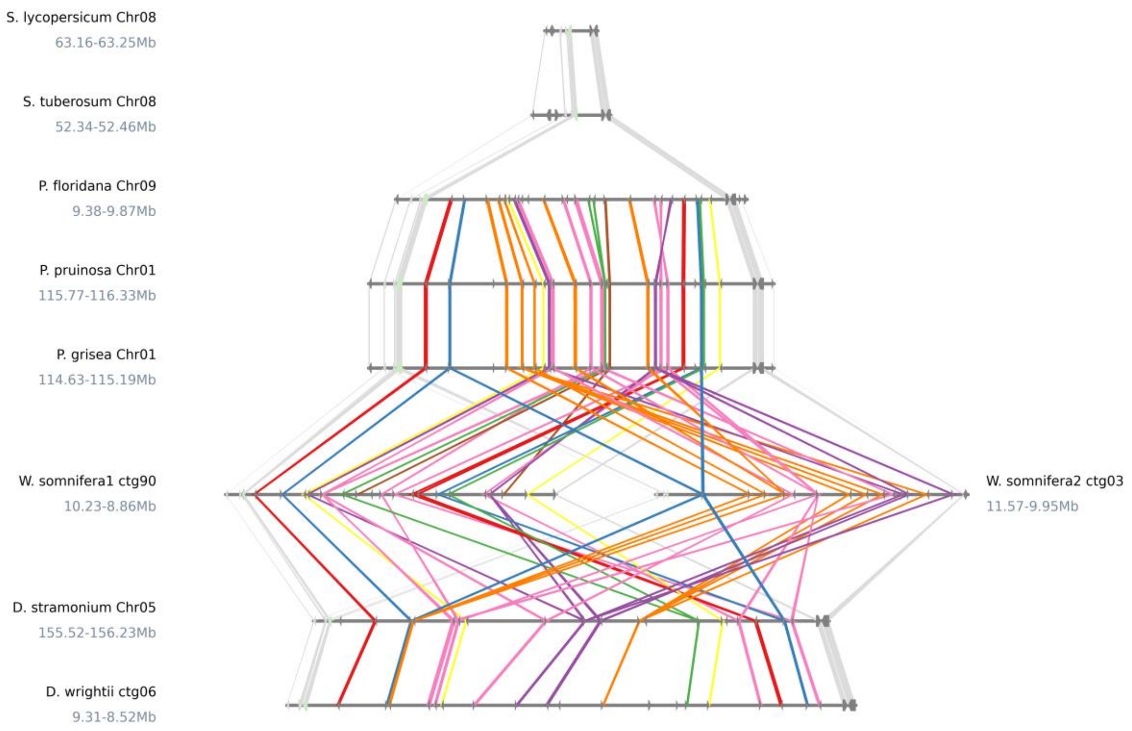

Supplementary Fig. 2. Syntenic biosynthetic gene clusters containing *24ISO* in withanolide-producing Solanaceae plants with their locations and sizes. The contig/chromosome numbers where the gene clusters are located are next to the species names while the specific genomic regions are described below the species names.

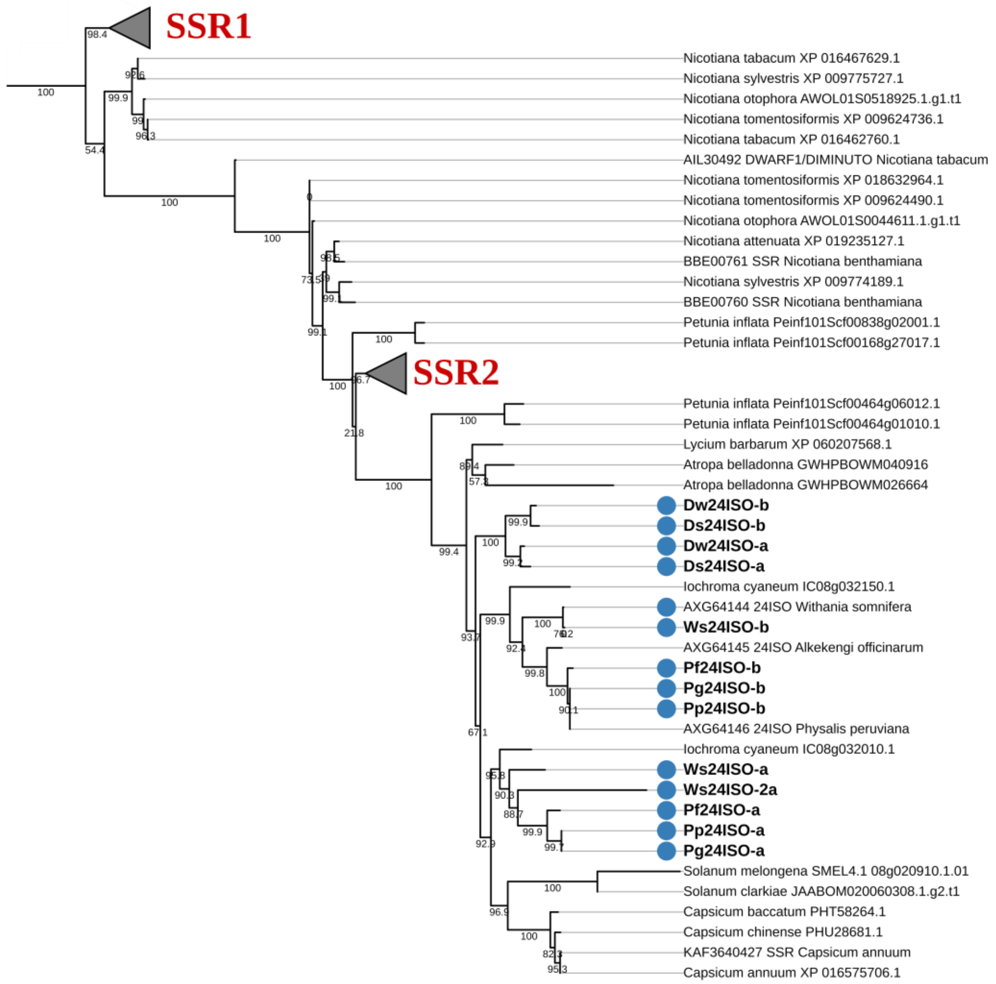

Supplementary Fig. 3. Codon-based phylogenetic tree of Δ^{24} isomerase genes (*24ISO*) in withanolide gene clusters. Sequences from known withanolide-producing Solanaceae species which are part of the gene cluster are marked by blue dots. Sequences identified in this study are highlighted in bold; a suffix (-a/-b etc.) was added to label homologues sequentially according to their position within the gene clusters (see Supplementary Data 1 for details). Closely related SSR1 and SSR2 sequences are used as outgroup. Numbers above the nodes are the bootstrap values of maximum likelihood (ML) analysis based on 1000 replicates. Ds: *Datura stramonium*, Dw: *Datura wrightii*, Ws: *Withania somnifera*, Pg: *Physalis grisea*, Pp: *Physalis pruinosa*, and Pf: *Physalis floridana*.

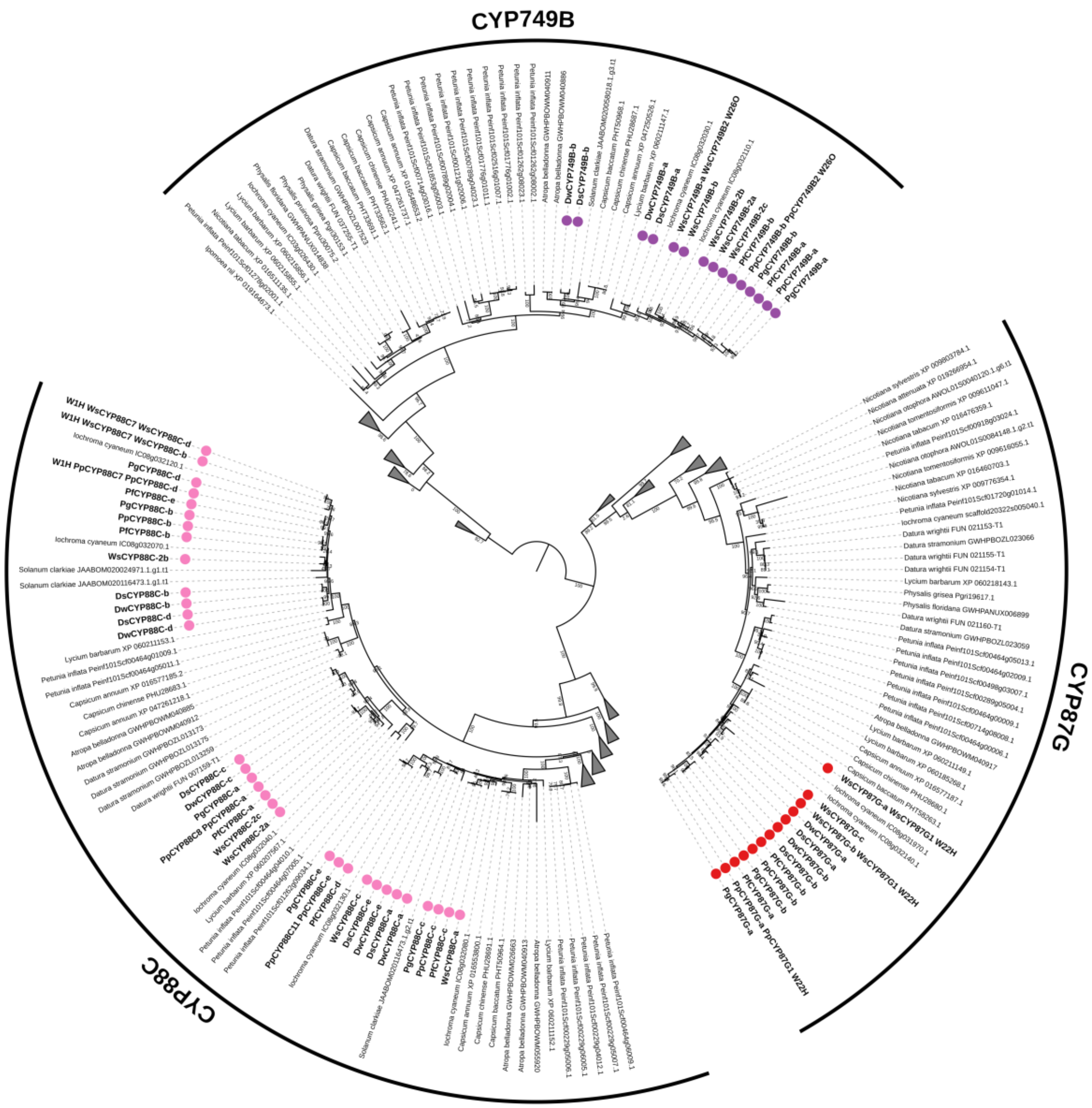

Supplementary Fig. 4. Codon-based phylogenetic tree of cytochrome P450 monooxygenase genes (*CYP*) in withanolide gene clusters. Sequences from known withanolide-producing Solanaceae species which are part of the gene cluster are marked by purple, red, and pink dots corresponding to CYP749B, CYP87G, and CYP88C sequences, respectively. Sequences identified in this study are highlighted in bold; a suffix (-a/-b etc.) was added to label homologues sequentially according to their position within the gene clusters (see Supplementary Data 1 for details). Sequences experimentally characterised in this work (CYP87G1, CYP88C7, CYP749B2) are additionally annotated with official CYP nomenclature and functional names. Numbers above the nodes are the bootstrap values of maximum likelihood (ML) analysis based on 1000 replicates. Ds: *Datura stramonium*, Dw: *Datura wrightii*, Ws: *Withania somnifera*, Pg: *Physalis grisea*, Pp: *Physalis pruinosa*, and Pf: *Physalis floridana*.

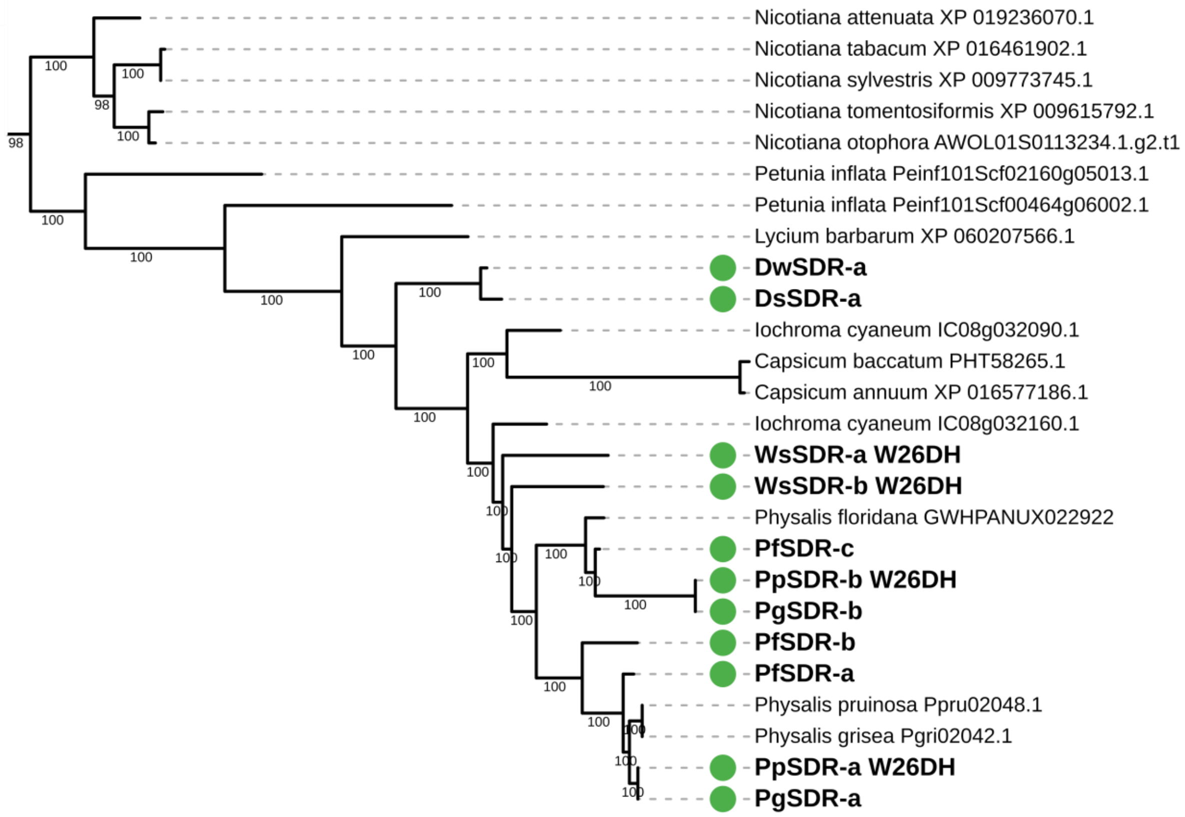

Supplementary Fig. 5. Codon-based phylogenetic tree of short-chain dehydrogenase genes (*SDR*) in withanolide gene clusters. Sequences from known withanolide-producing Solanaceae species which are part of the gene cluster are marked by green dots. Sequences identified in this study are highlighted in bold; a suffix (-a/-b etc.) was added to label homologues sequentially according to their position within the gene clusters (see Supplementary Data 1 for details). Sequences experimentally characterised in this work are additionally annotated with functional names. Numbers above the nodes are the bootstrap values of maximum likelihood (ML) analysis based on 1000 replicates. Ds: *Datura stramonium*, Dw: *Datura wrightii*, Ws: *Withania somnifera*, Pg: *Physalis grisea*, Pp: *Physalis pruinosa*, and Pf: *Physalis floridana*.

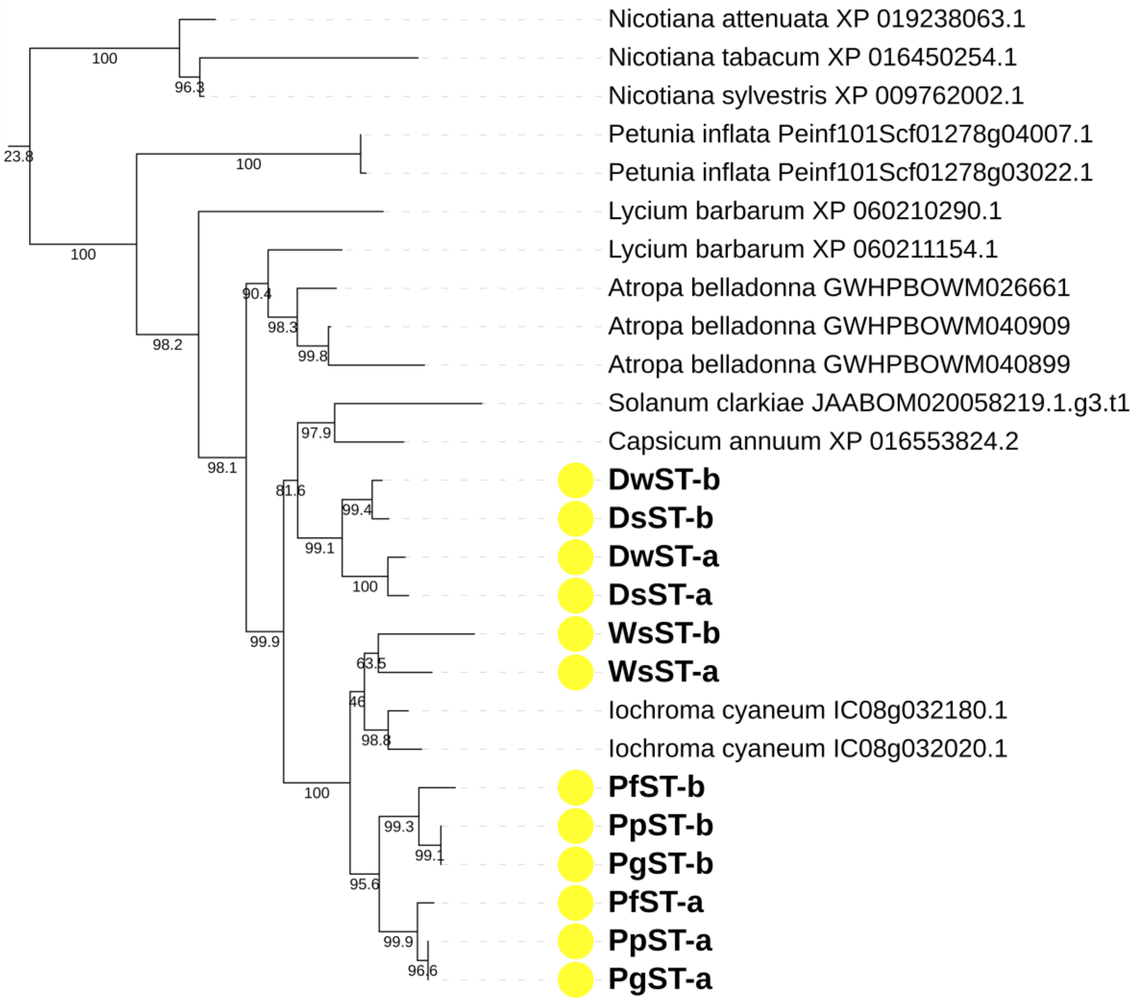

Supplementary Fig. 6. Codon-based phylogenetic tree of sulfotransferase genes (*ST*) in withanolide gene clusters. Sequences from known withanolide-producing Solanaceae species which are part of the gene cluster are marked by yellow dots. Sequences identified in this study are highlighted in bold; a suffix (-a/-b etc.) was added to label homologues sequentially according to their position within the gene clusters (see Supplementary Data 1 for details). Numbers above the nodes are the bootstrap values of maximum likelihood (ML) analysis based on 1000 replicates. Ds: *Datura stramonium*, Dw: *Datura wrightii*, Ws: *Withania somnifera*, Pg: *Physalis grisea*, Pp: *Physalis pruinosa*, and Pf: *Physalis floridana*.

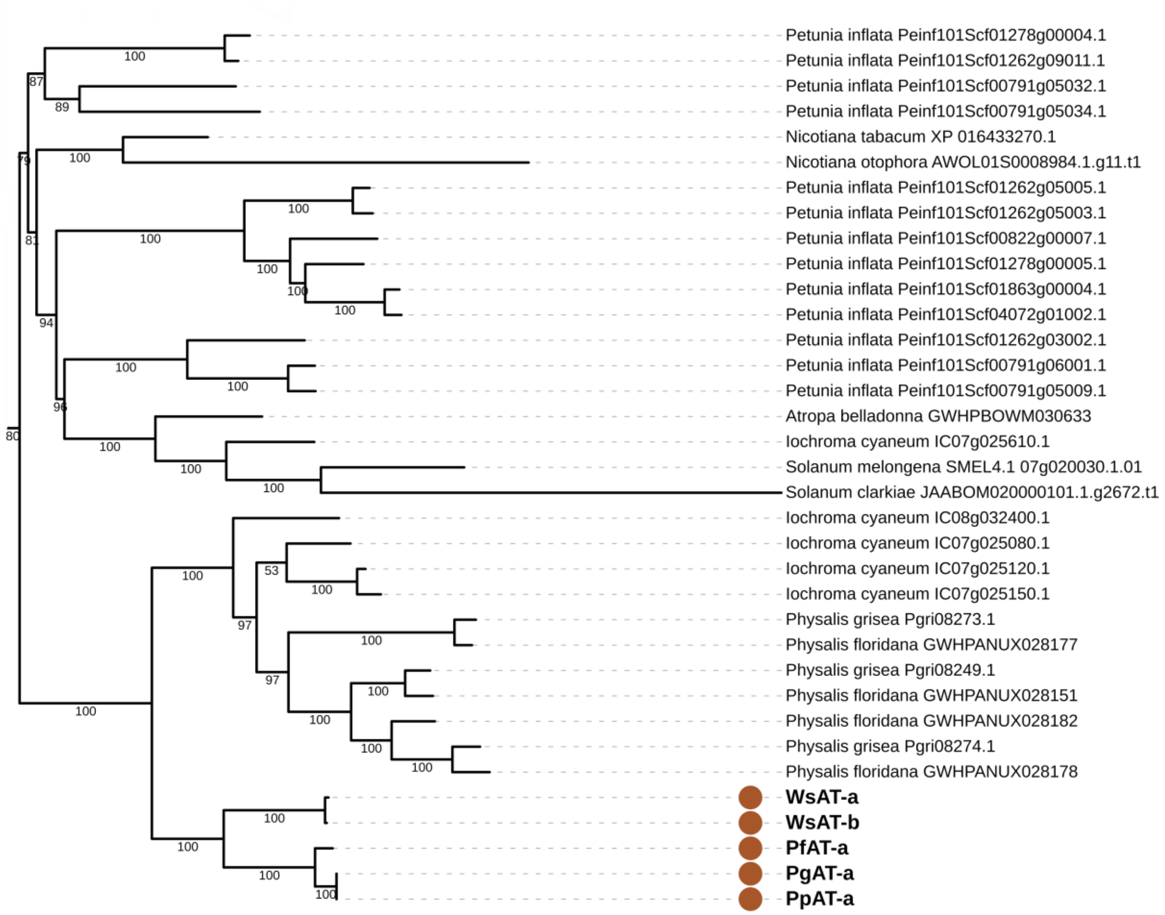

Supplementary Fig. 7. Codon-based phylogenetic tree of acyltransferase genes (*AT*) in withanolide gene clusters. Sequences from known withanolide-producing Solanaceae species which are part of the gene cluster are marked by brown dots. Sequences identified in this study are highlighted in bold; a suffix (-a/-b etc.) was added to label homologues sequentially according to their position within the gene clusters (see Supplementary Data 1 for details). Numbers above the nodes are the bootstrap values of maximum likelihood (ML) analysis based on 1000 replicates. Ds: *Datura stramonium*, Dw: *Datura wrightii*, Ws: *Withania somnifera*, Pg: *Physalis grisea*, Pp: *Physalis pruinosa*, and Pf: *Physalis floridana*.

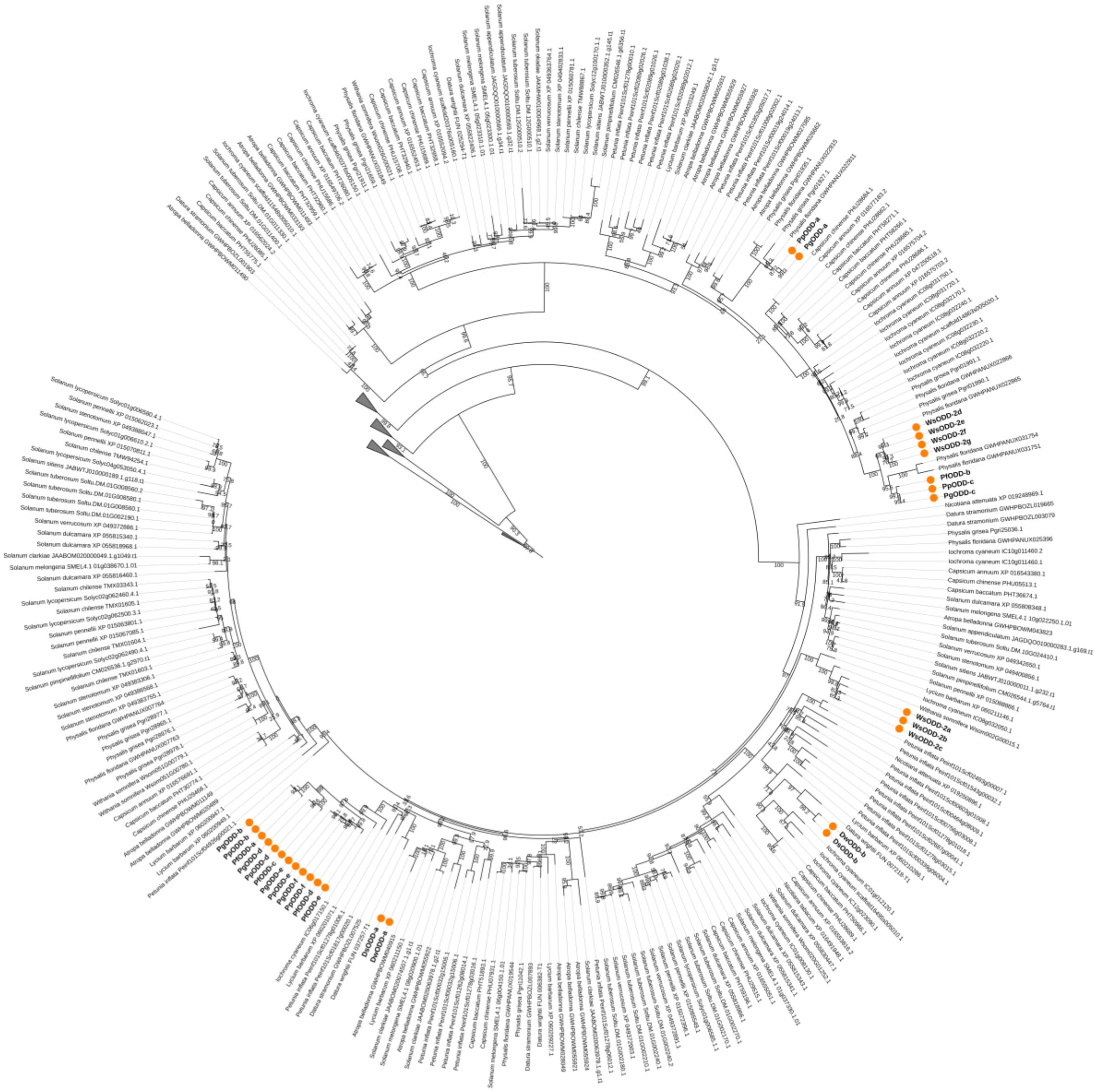

Supplementary Fig. 8. Codon-based phylogenetic tree of 2-oxoglutarate-dependent dioxygenase genes (*ODD*) in withanolide gene clusters. Sequences from known withanolide-producing Solanaceae species which are part of the gene cluster are marked by orange dots. Sequences identified in this study are highlighted in bold. Numbers above the nodes are the bootstrap values of maximum likelihood (ML) analysis based on 1000 replicates. Ds: *Datura stramonium*, Dw: *Datura wrightii*, Ws: *Withania somnifera*, Pg: *Physalis grisea*, Pp: *Physalis pruinosa*, and Pf: *Physalis floridana*.

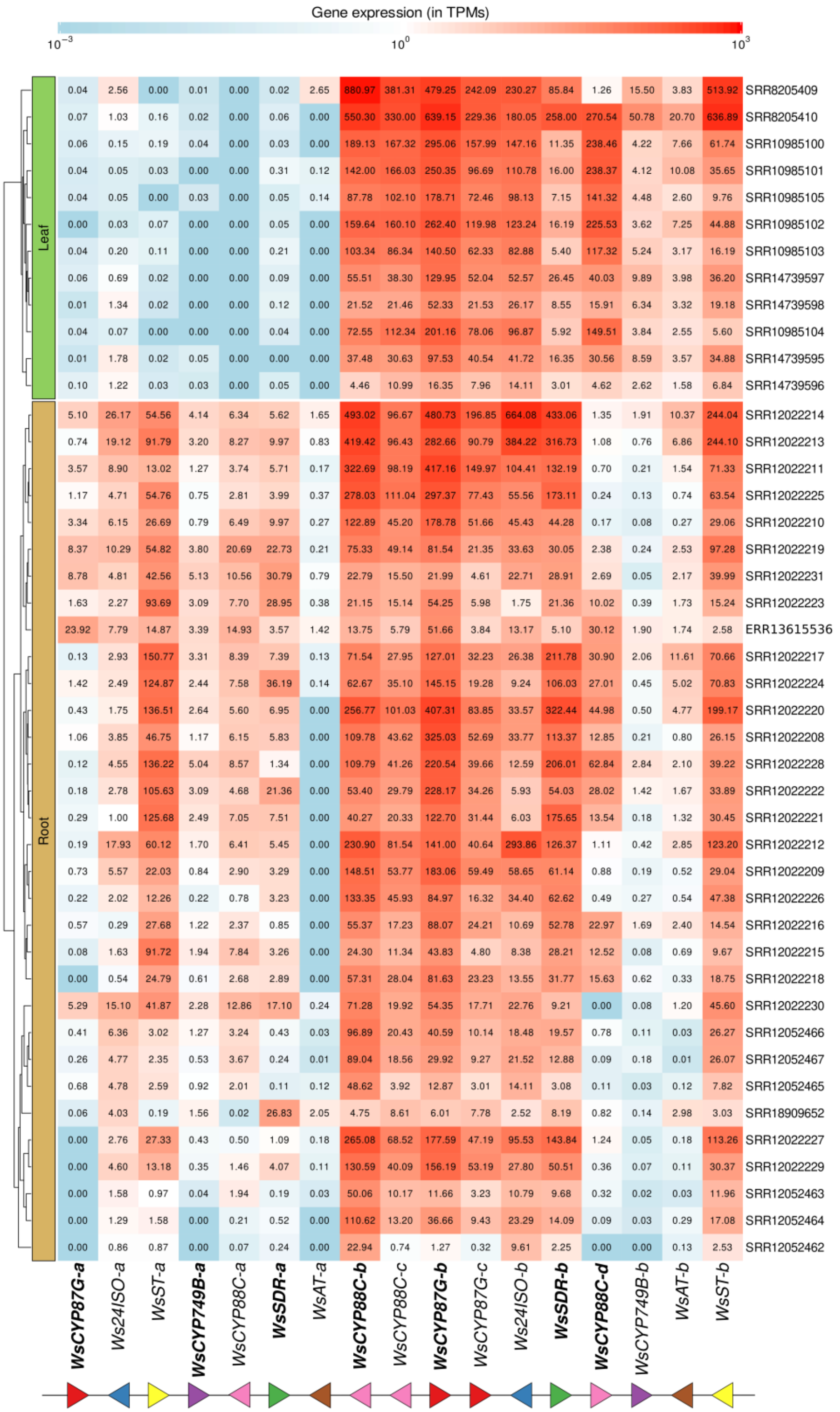

Supplementary Fig. 9. Genes within the withanolide gene cluster 1 of *W. somnifera* are co-expressed but fall into two distinct expression pattern groups. Genes shown in bold (CYP87G1 = W22H; CYP88C7 = W1H; CYP749B2 = W26O; SDR = W26DH) were functionally characterized in this work.

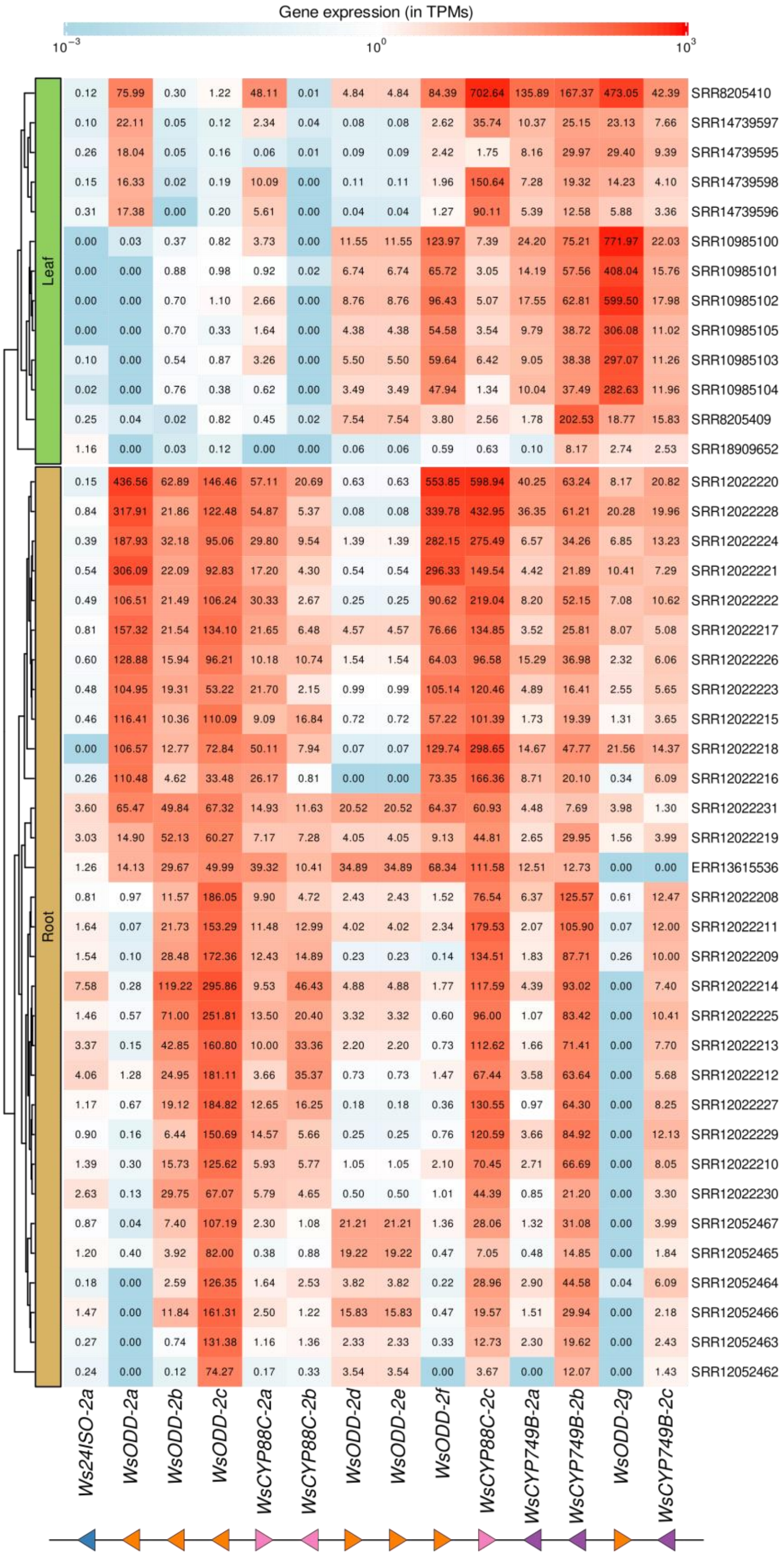

Supplementary Fig. 10. Expression patterns of genes from the second *24ISO*-containing gene cluster ("*W. somnifera* 2") in the genome of *W. somnifera*.

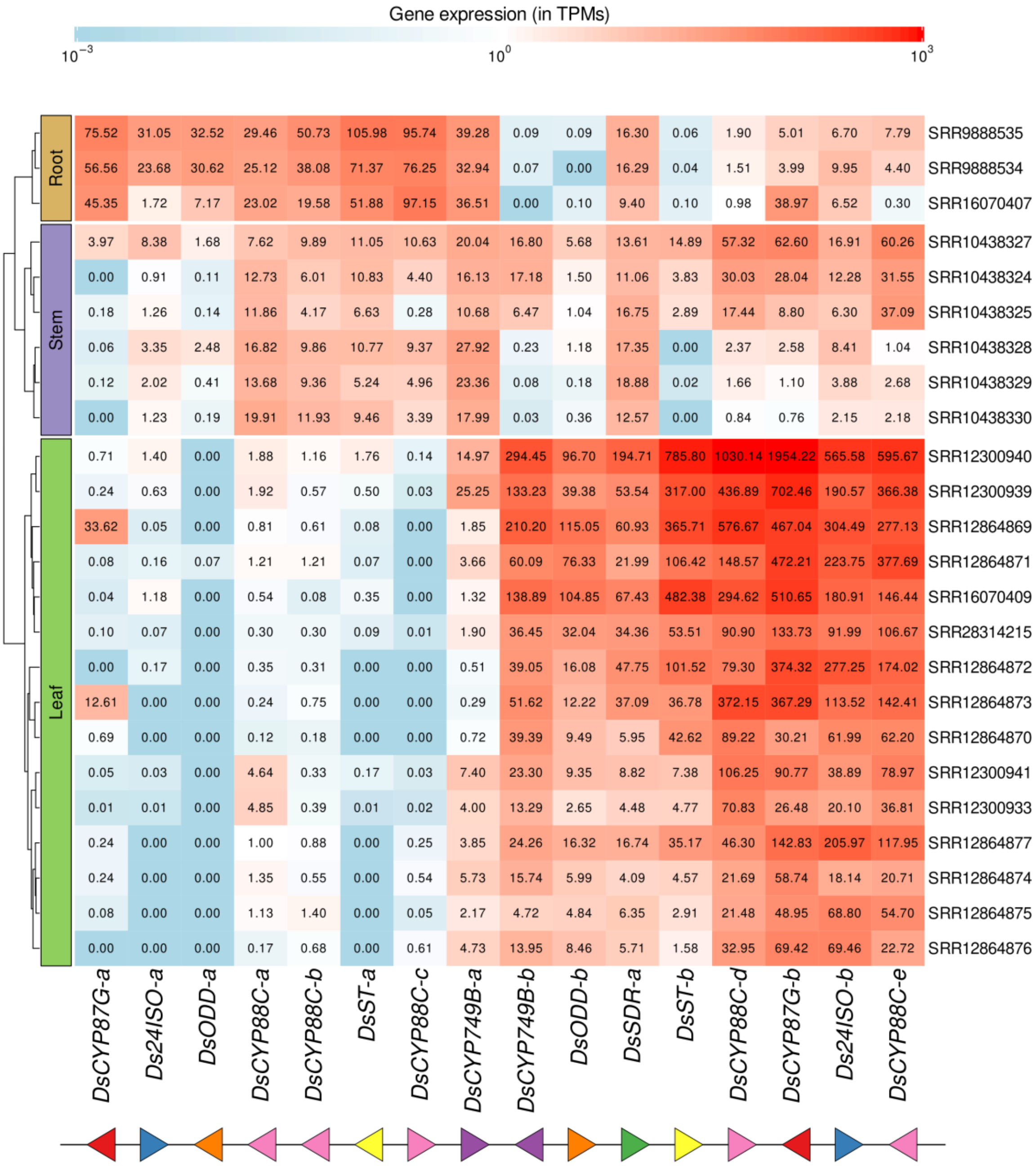

Supplementary Fig. 11. Genes within the withanolide gene cluster of *D. stramonium* are co-expressed but fall into two distinct expression pattern groups.

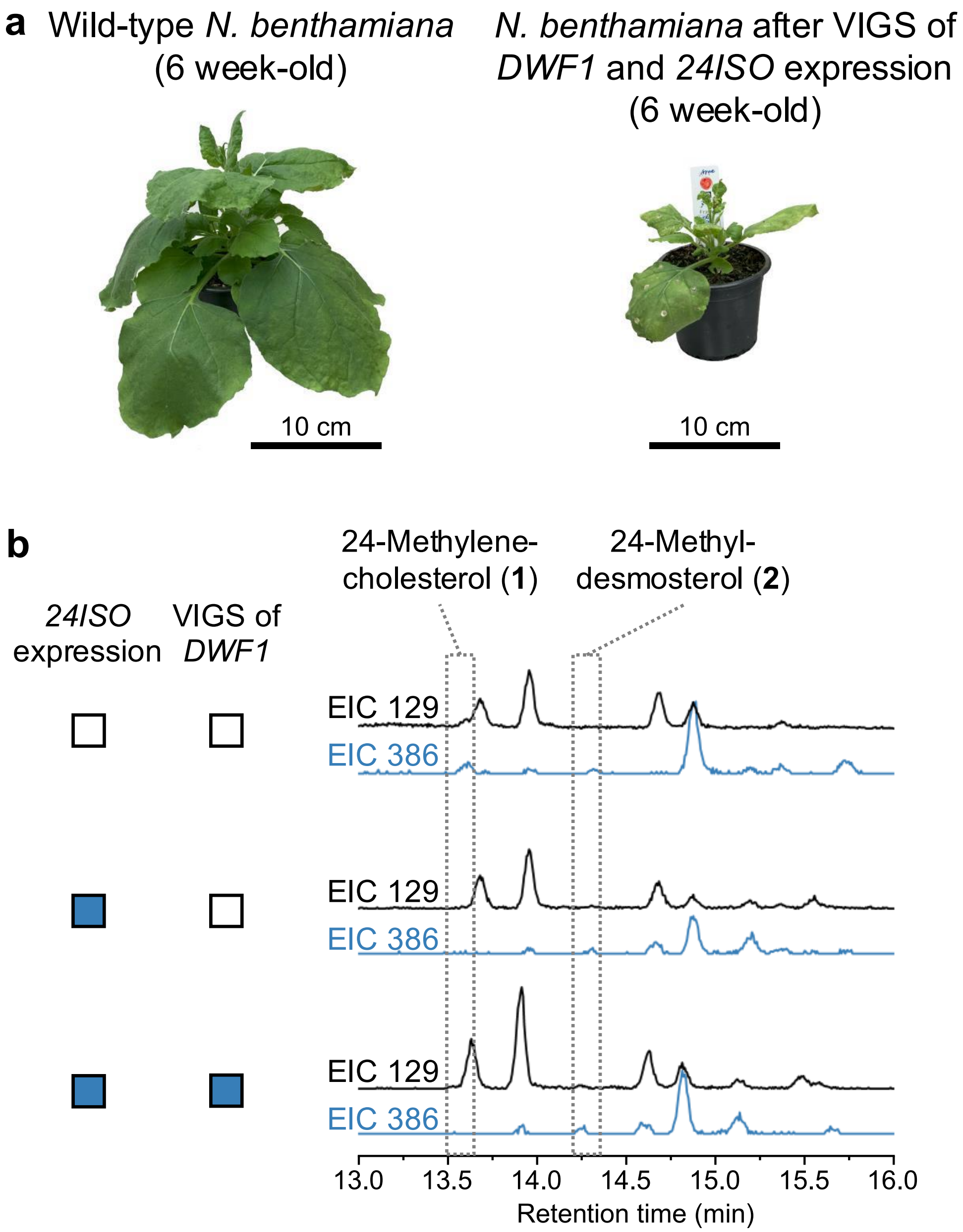

Supplementary Fig. 12. Combining virus-induced gene silencing of *DWF1* with transient expression of *24ISO* leads to production of trace amounts of 24-methyldesmosterol (2) but comes with a severe dwarf phenotype. a Comparison of a typical *N. benthamiana* wild-type plant and a representative plant after virus-induced gene silencing of *DWF1* and transient expression of *24ISO*. **b** Extracted ion chromatograms (EIC) from GC-MS analysis of different combinations of *24ISO* expression and *DWF1* silencing. *m*/*z* 129 is a general A ring fragment of typical steroids, whereas *m*/*z* 386 is specific for 24-methylenecholesterol (**1**) and 24-methyldesmosterol (**2**) (Supplementary Fig. 14-16 and Supplementary Data 2).

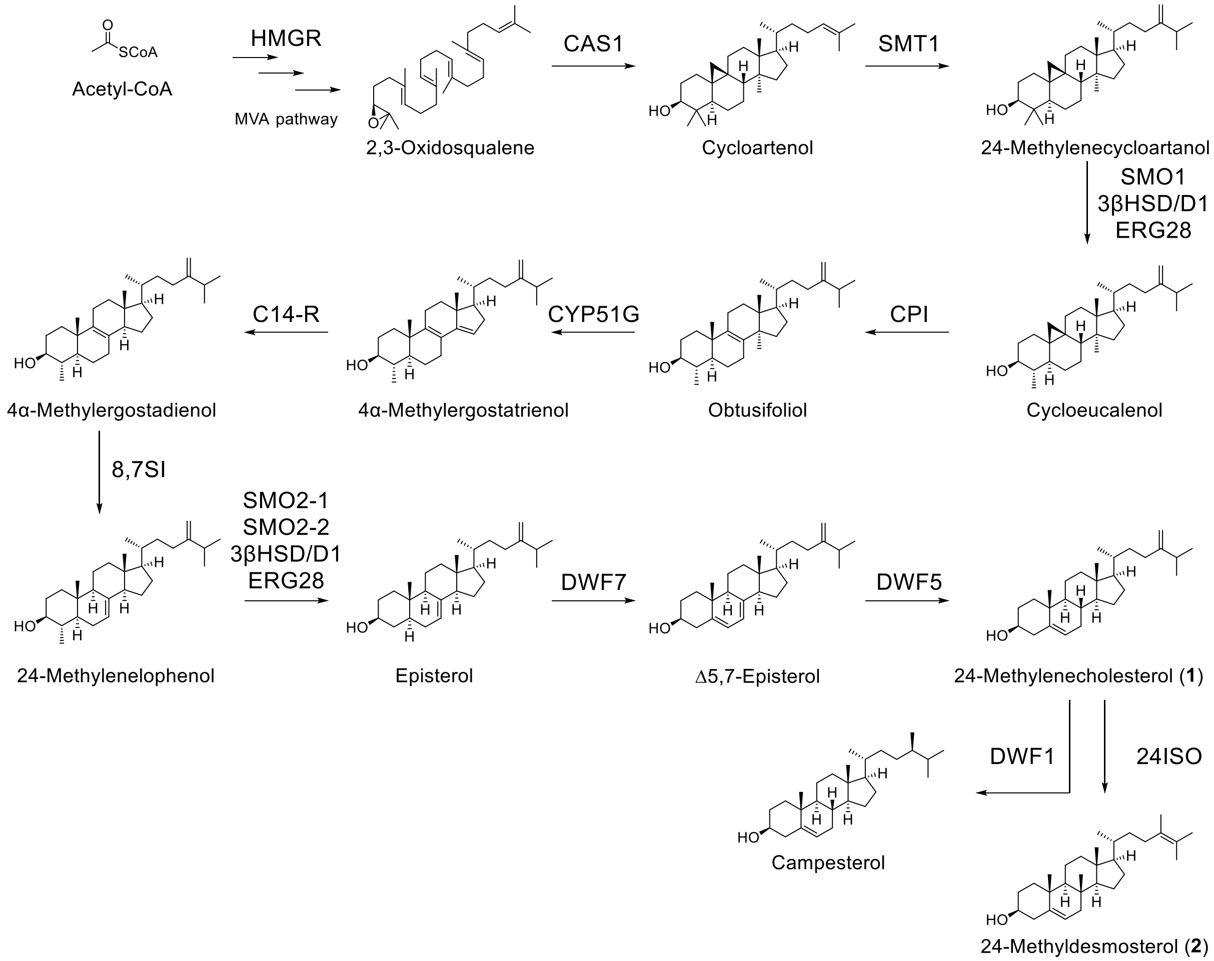

Supplementary Fig. 13. Full phytosterol pathway transiently overproduced in this work.

HMGR: 3-hydroxy-3-methyl-glutaryl-coenzyme A reductase; **CAS1:** cycloartenol synthase 1; **SMT1:** sterol C-24 methyltransferase; **SMO1 and SMO2-1/2:** sterol C-4 methyl oxidase 1 and 2-1/2-2; **3βHSD/D1:** 3β-hydroxysteroid dehydrogenase/C-4 decarboxylase 1; **ERG28:** ergosterol biosynthetic protein 28; **CPI:** cyclopropylsterol isomerase; **CYP51G:** sterol C-14 demethylase; **C14-R:** sterol C-14 reductase; **8,7SI:** sterol 8,7 isomerase; **DWF7 (DWARF7/STE1):** Δ7 sterol C-5 desaturase; **DWF5 (DWARF5 / 7RED):** sterol Δ7 reductase; **DWF1 (DWARF1/DIM):** sterol C-24 reductase; **24ISO:** sterol Δ24 isomerase.

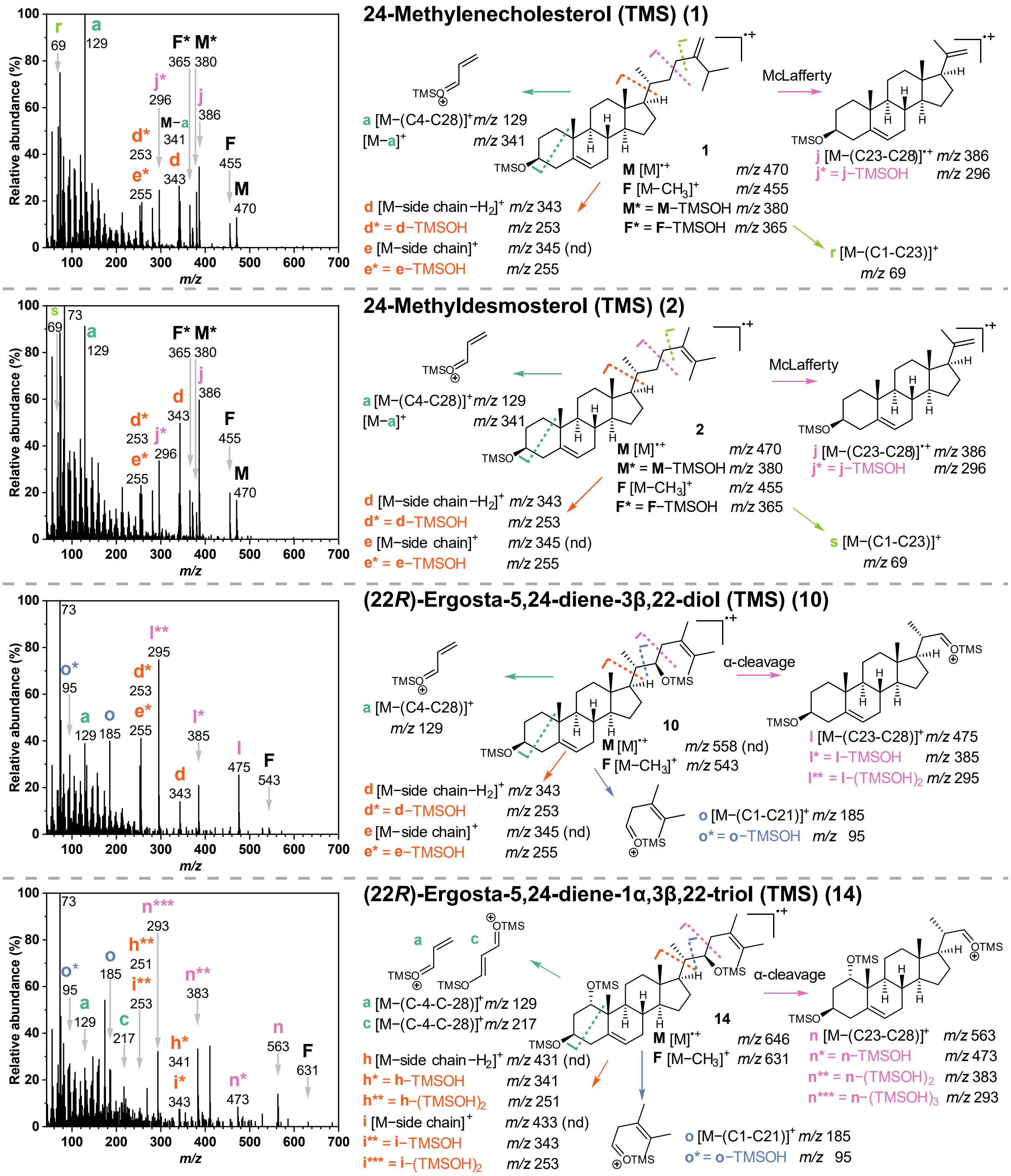

Supplementary Fig. 14. Electron impact mass spectra and proposed fragmentation routes of main withanolide pathway intermediates from this work. See also Supplementary Data 2.

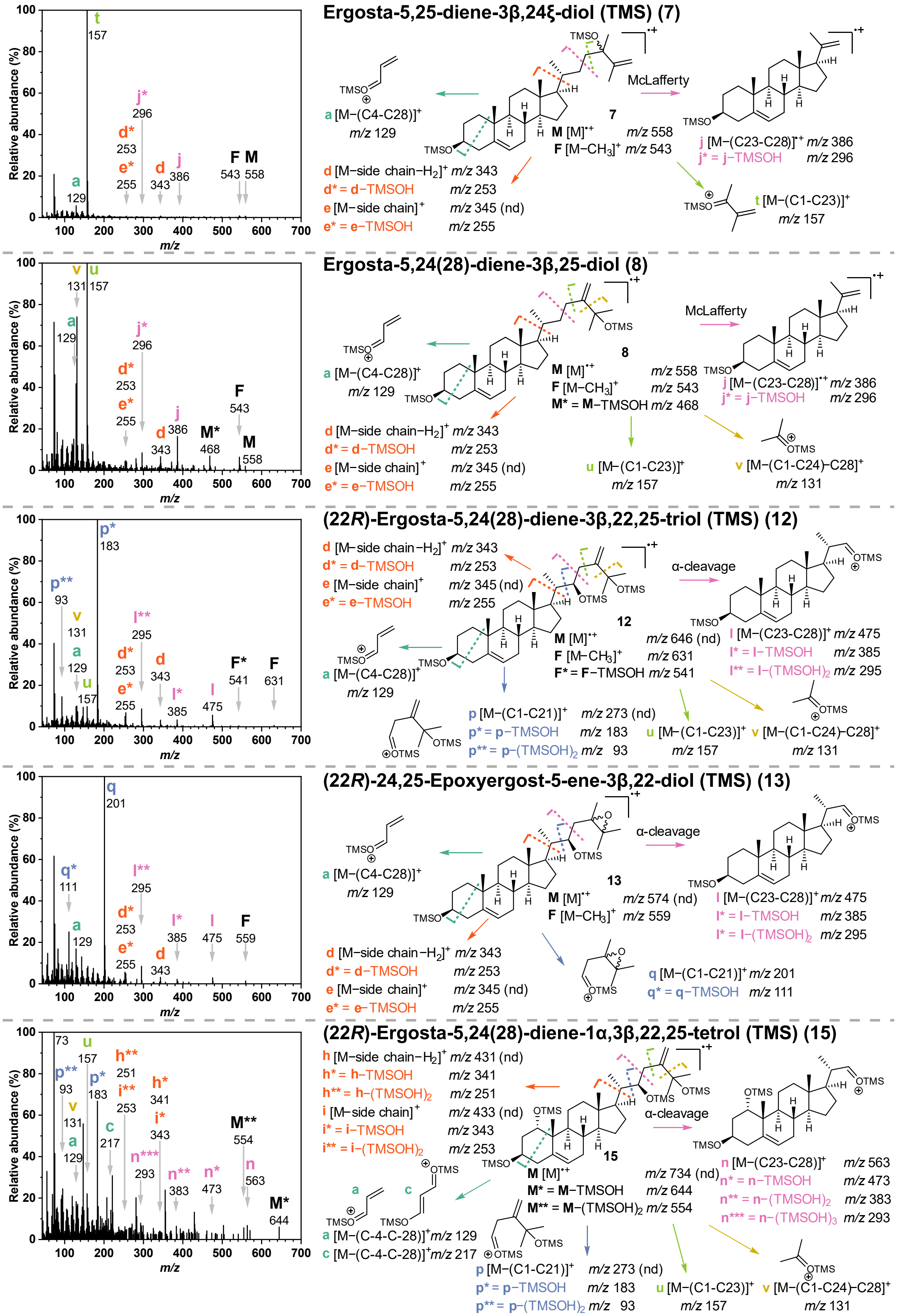

Supplementary Fig. 15. Electron impact mass spectra and proposed fragmentation routes of *N. benthamiana* shunt products from this work. See also Supplementary Data 2.

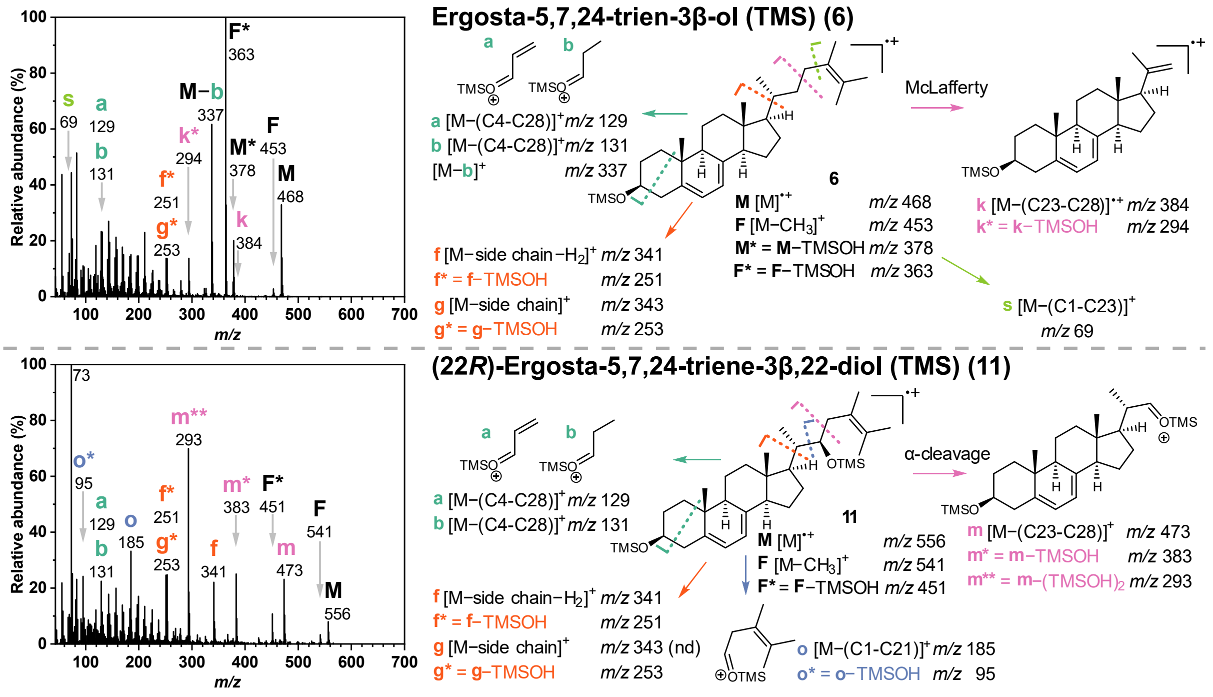

Supplementary Fig. 16. Electron impact mass spectra and proposed fragmentation routes of *S. cerevisiae* shunt products from this work. See also Supplementary Data 2.

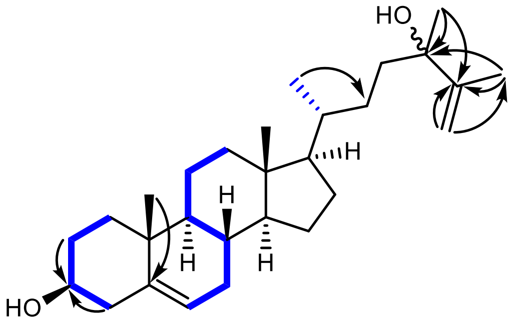

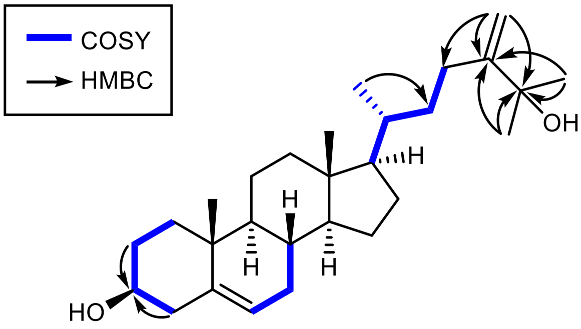

Ergosta-5,25-diene-3β,24ξ-diol
(physalindicanol A) (**7**)

Ergosta-5,24(28)-diene-3β,25-diol
(physalindicanol B) (**8**)

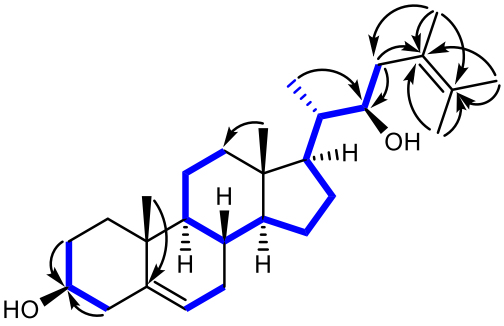

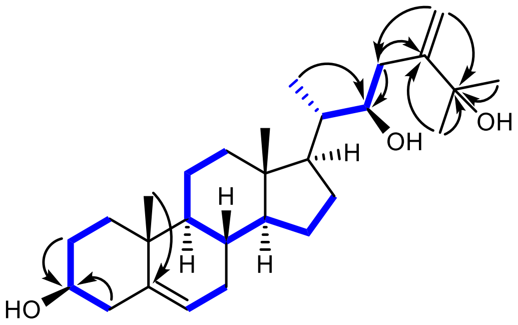

(22*R*)-Ergosta-5,24-diene-3β,22-diol (**10**)

(22*R*)-Ergosta-5,24(28)-diene-3β,22,25-triol
(phyministerol A) (**12**)

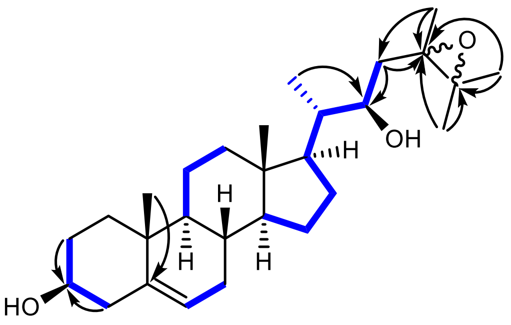

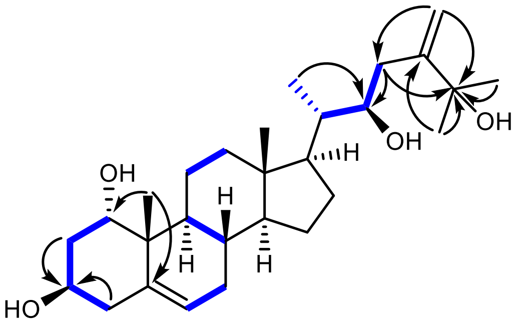

(22*R*)-24,25-Epoxyergost-5-ene-3β,22-diol (**13**)

(22*R*)-Ergosta-5,24(28)-diene-1α,3β,22,25-tetrol (**15**)

Supplementary Fig. 17. Key NMR correlations (COSY and HMBC) for the structure elucidation of isolated compounds.

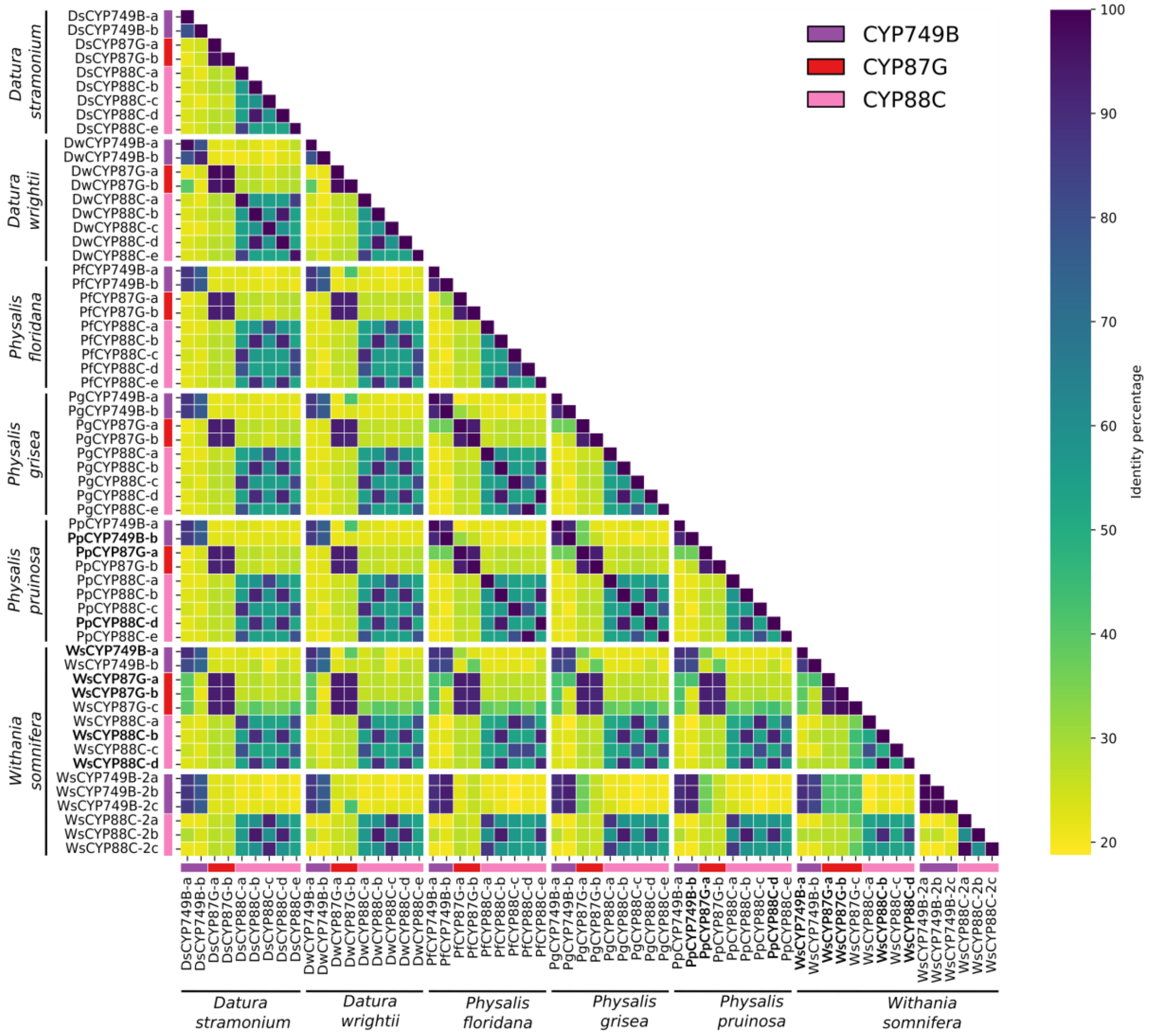

Supplementary Fig. 18. Protein sequence identity matrix of the CYPs identified in the withanolide gene clusters.

Gene sequences shown in bold (CYP87G1 = W22H; CYP88C7 = W1H; CYP749B2 = W26O) were functionally characterized in this work. Source data are provided as a Source Data file.

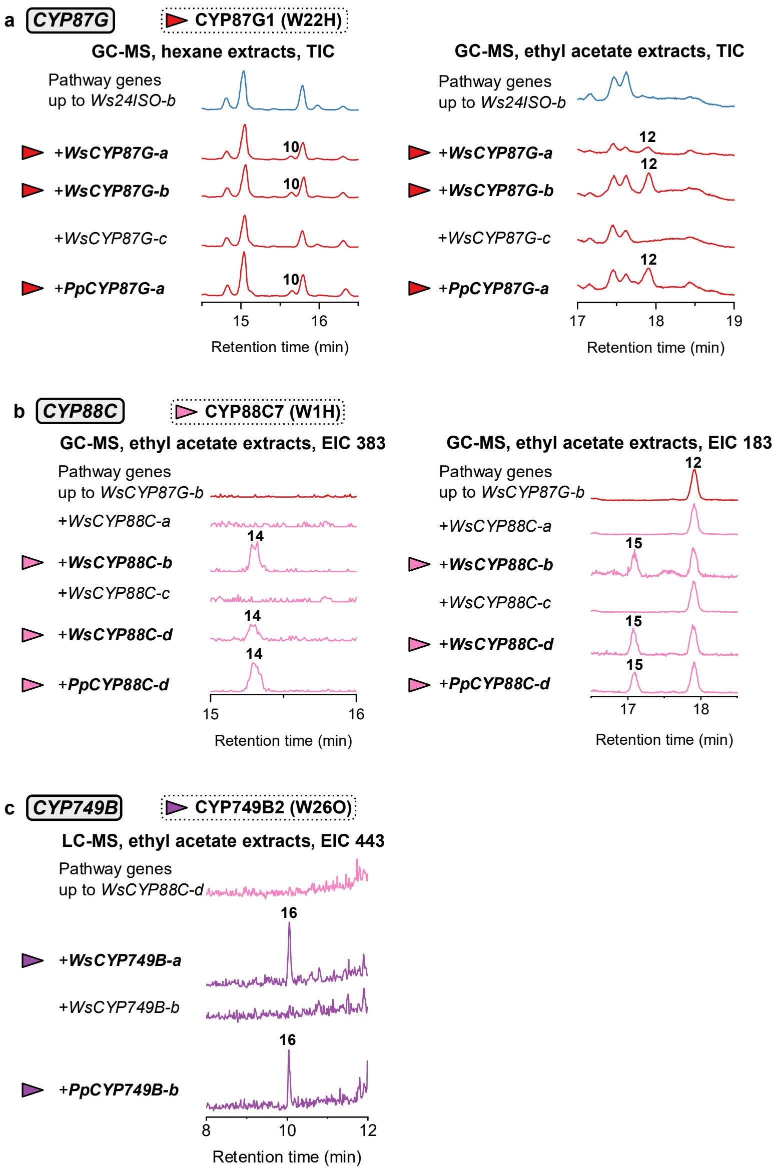

Supplementary Fig. 19. Conserved function of CYPs encoded by withanolide gene clusters within and across species demonstrated by co-expression in *N. benthamiana*.

CYP homologues with conserved function as CYP87G1 (W22H), CYP88C7 (W1H), or CYP749B2 (W26O) are highlighted with coloured triangles and in bold. **a** The two CYP87G homologues a and b from *W. somnifera* produce the same product (22*R*)-ergosta-5,24-diene-3β,22-diol (**10**) and shunt product (22*R*)-ergosta-5,24(28)-diene-3β,22,25-triol (**12**) as the one tested homologue a from *P. pruinosa*. **b** The two CYP88C homologues b and d from *W. somnifera*, which belong to the CYP88C7 orthogroup, produce the same product (22*R*)-ergosta-5,24-diene-1α,3β,22-triol (**14**) and shunt product (22*R*)-ergosta-5,24(28)-diene-1α,3β,22,25-tetrol (**15**) as the one tested homologue d from *P. pruinosa*. Notably, *W. somnifera* CYP88C homologues a and c which belong to a different orthogroup did not show activity under these conditions. **c** *Ws*CYP749B-a and *Pp*CYP749B-b produced the same product withanoside V aglycone (**16**), whereas no activity was observed for *Ws*CYP749B-b, the other CYP749B homologue encoded in *W. somnifera* gene cluster 1.

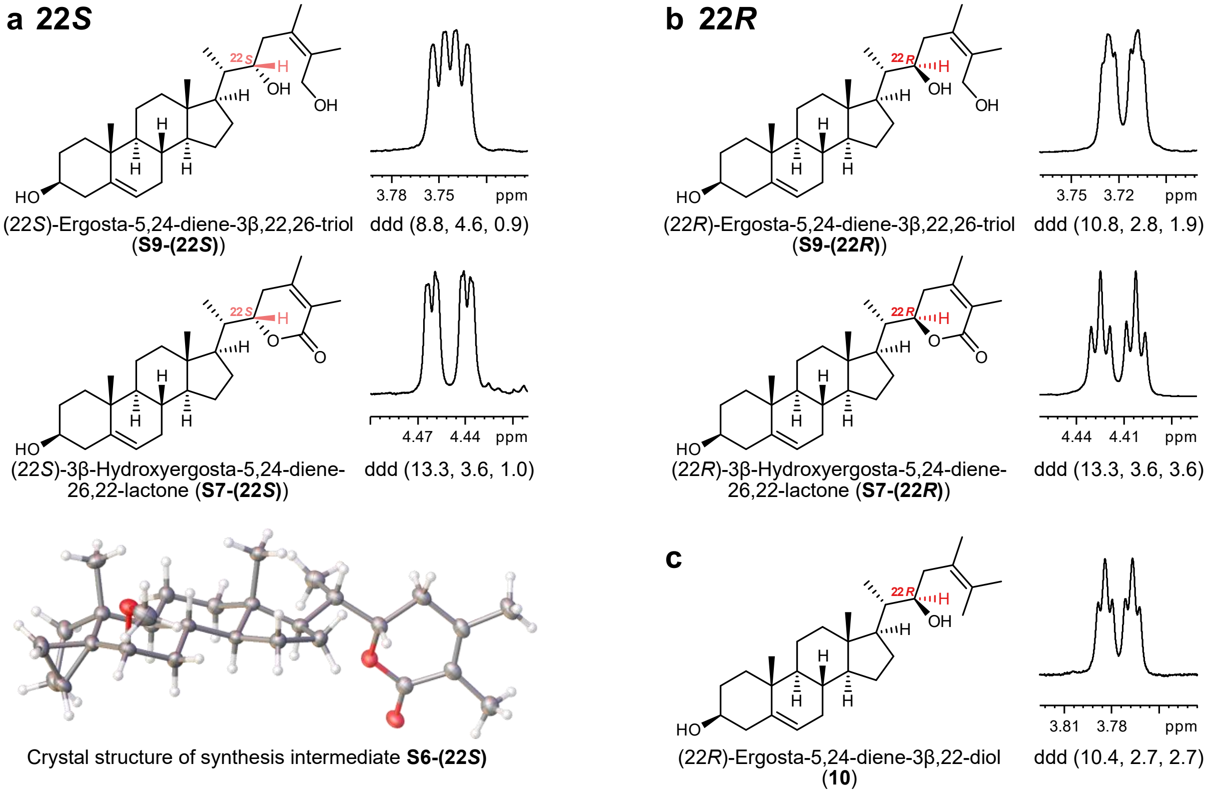

Supplementary Fig. 20. Determination of C-22 stereochemistry of (22*R*)-ergosta-5,24-diene-3β,22-diol (10) by comparison with H-22 multiplets of synthetic diastereomer pairs S7-(22*S*/*R*) and S9-(22*S*/*R*) (MeOD, 600 MHz, 298 K).

a H-22 multiplets and coupling constants of synthetic 22*S* compounds **S9-(22*S*)** and **S7-(22*S*)**. The 22*S* stereochemistry was confirmed by X-ray analysis of synthesis intermediate **S6-(22*S*)** (see also Supplementary Table 18).

b H-22 multiplets and coupling constants of synthetic 22*R* compounds **S9-(22*R*)** and **S7-(22*R*)**.

c H-22 multiplet and coupling constants of (22*R*)-ergosta-5,24-diene-3β,22-diol (**10**). The occurrence of two approximately equal minor coupling constants indicates a 22*R* configuration.

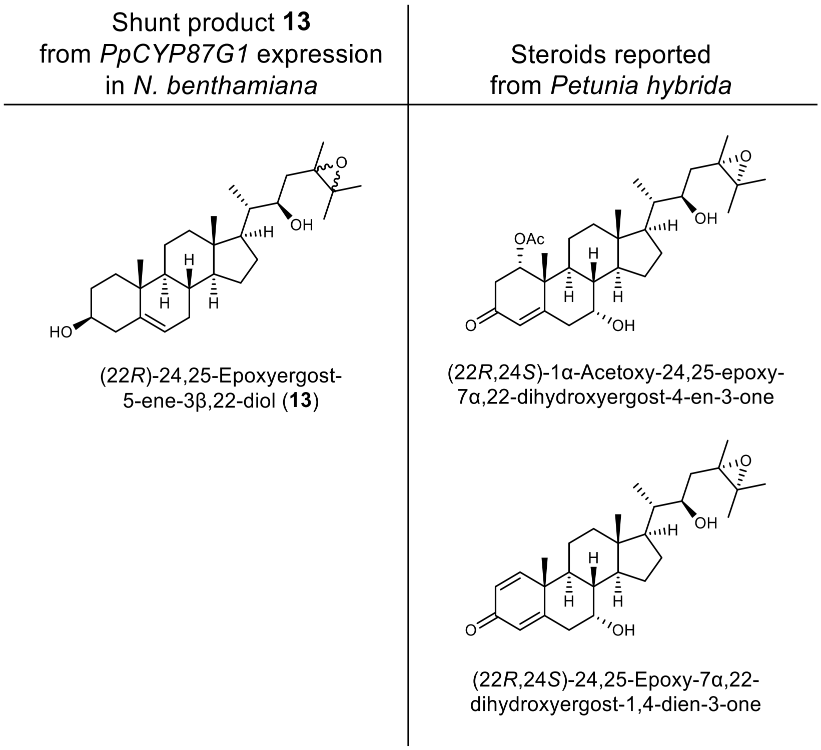

Supplementary Fig. 21. Structure comparison of shunt product 13 with steroids isolated from *Petunia hybrida* (Solanaceae)[21].

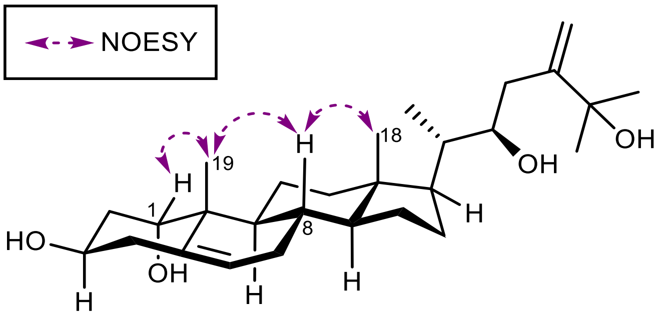

Supplementary Fig. 22. Key NOESY correlations in support of 1α configuration of (22*R*)-ergosta-5,24(28)-diene-1α,3β,22,25-tetrol (15).

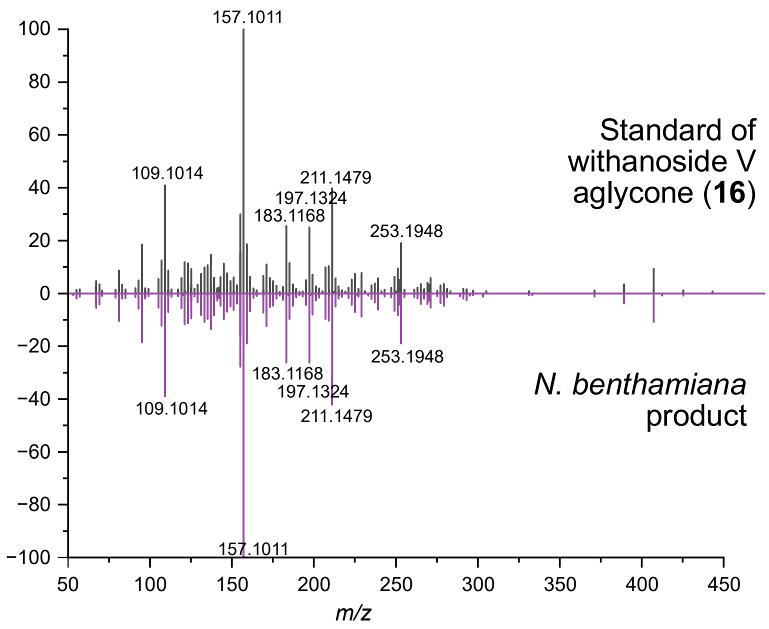

Supplementary Fig. 23. Mirror plot comparing high resolution MS/MS spectra (35 eV) of authentic withanoside V aglycone (16) and the product from heterologous expression of withanolide pathway genes in *N. benthamiana*.

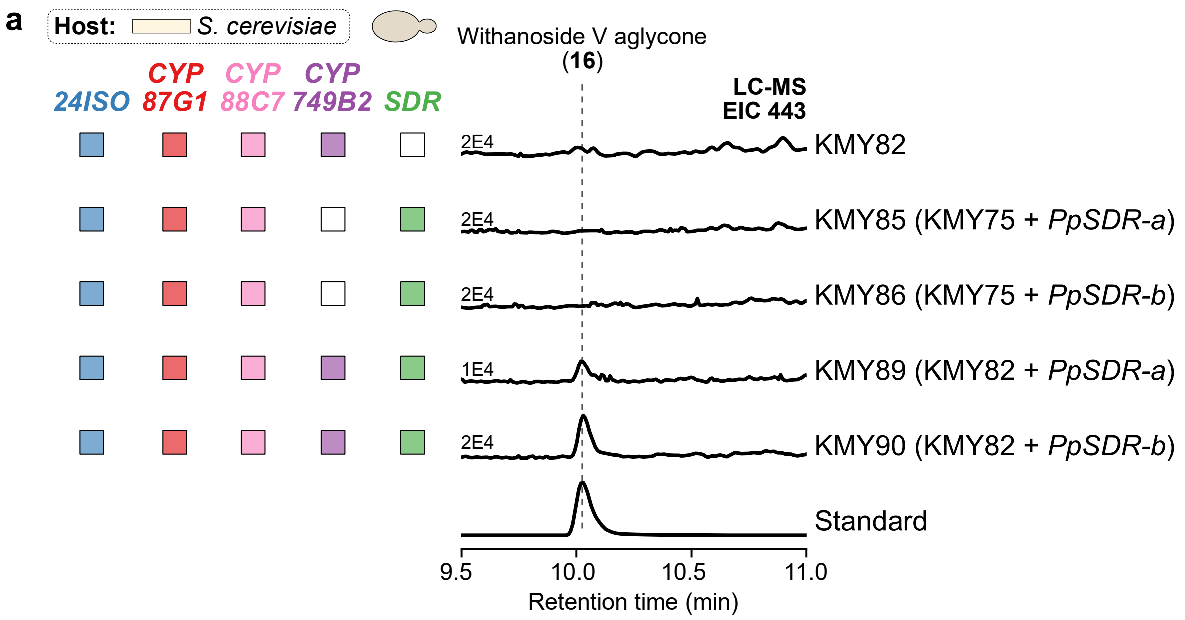

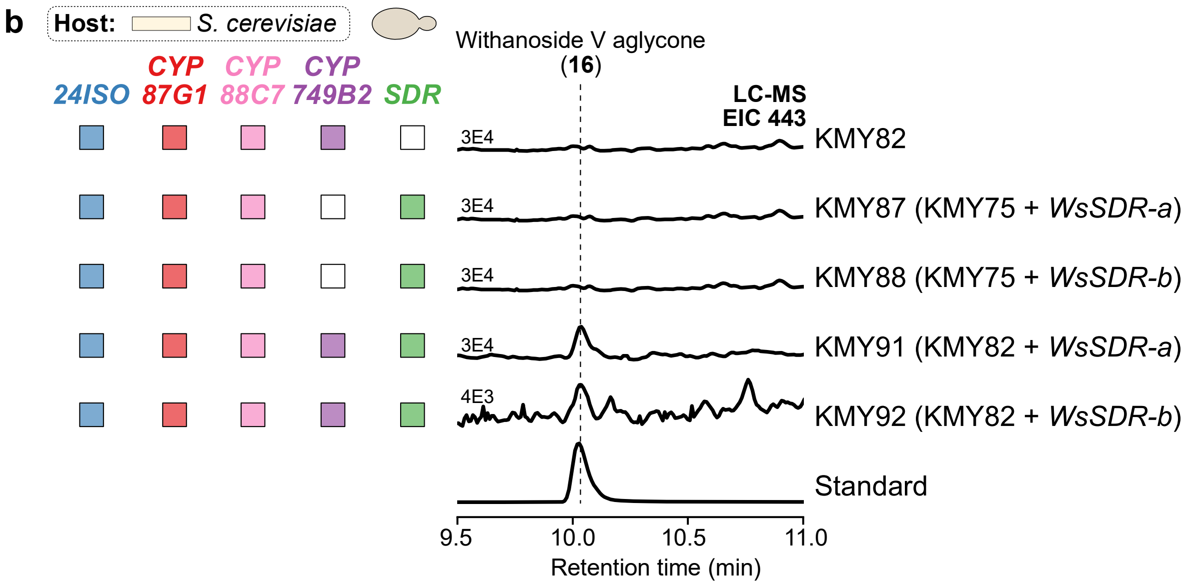

Supplementary Fig. 24. Formation of withanoside V aglycone (16) in yeast strains including *SDR*.

Both *SDR* homologues *a*/*b* from *P. pruinosa* (a) and from *W. somnifera* (b) enabled lactone formation when combined with *24ISO* and the three CYP genes *CYP87G1*, *CYP88C7*, and *CYP749B2*.

Budding_yeast icon by umasstr https://github.com/umasstr is licensed under CC0 https://creativecommons.org/publicdomain/zero/1.0/ and was used without modifications.

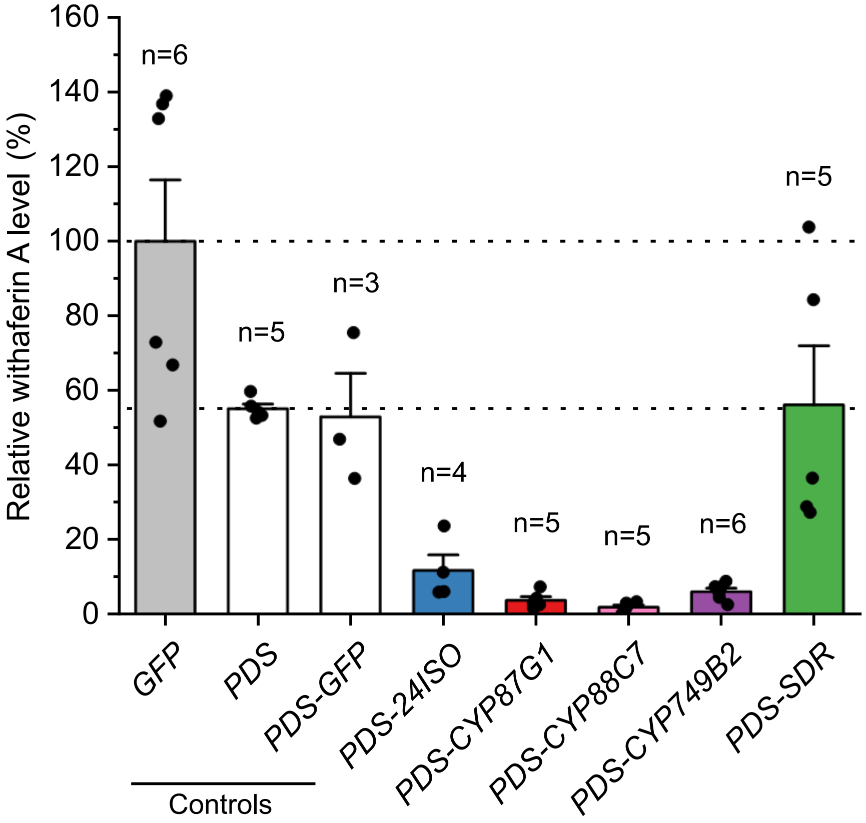

Supplementary Fig. 25. Effect of virus-induced gene silencing in *W. somnifera* on withaferin A levels including additional controls.

As silencing of the visual marker gene *PDS* alone leads to a decrease in withaferin A levels compared to a nonspecific *GFP* silencing control, all gene silencing experiments shown in Fig. 8 and Supplementary Fig. 26 (*PDS* plus target gene) were evaluated against a *PDS-GFP* co-silencing control.

For each sample group, six plants were infiltrated; the sample size shown above the bars refers to the number of plants that showed a photobleaching phenotype from *PDS* silencing (except for the *GFP* only control). Only leaf parts with photobleaching were considered as successfully silenced and analysed (except for the *GFP* only control).

Similar results were observed in two independent experimental batches.

Source data are provided as a Source Data file.

a

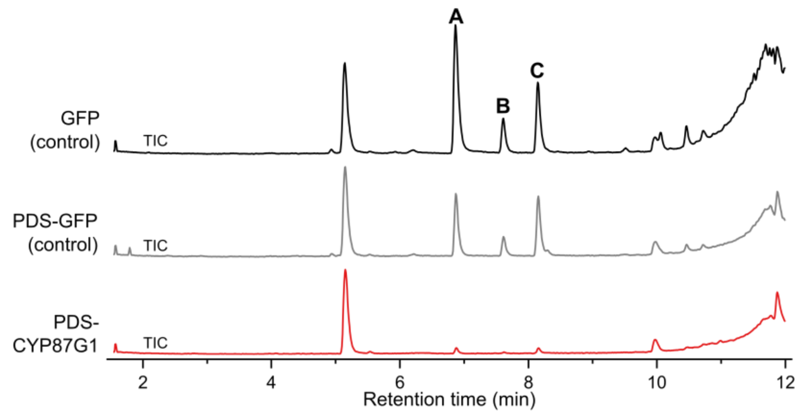

Compound	Retention time (min)	Molecular formula	Calc. mass	Mass	ppm	Compound
A	6.9	$C_{28}H_{39}O_6^+$ [M+H]$^+$	471.2741	471.2708	-7.0	Withaferin A
B	7.6	$C_{28}H_{39}O_6^+$ [M+H]$^+$	471.2741	471.2748	1.5	Unidentified withanolide
C	8.2	$C_{28}H_{41}O_6^+$ [M+H]$^+$	473.2898	473.2910	2.5	Unidentified withanolide

b

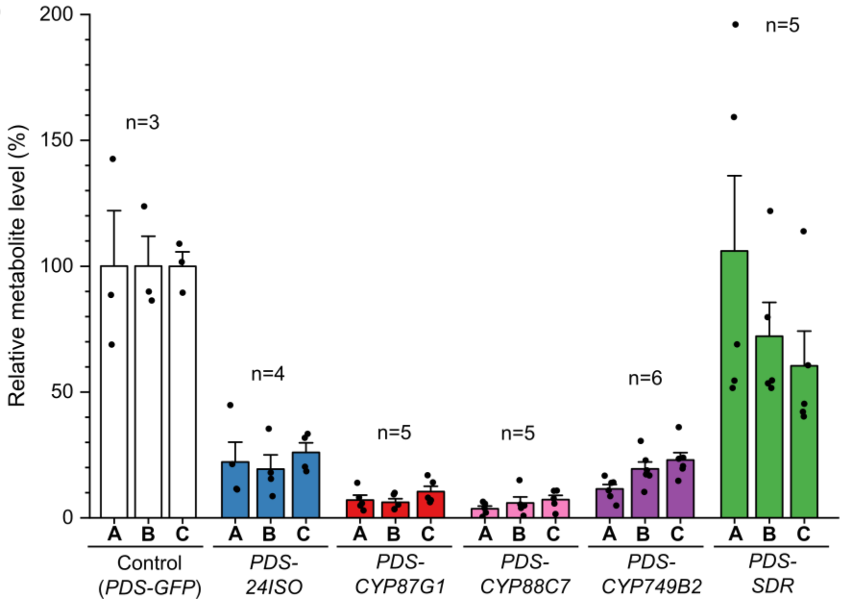

Supplementary Fig. 26. Effect of virus-induced gene silencing in *W. somnifera* on levels of withaferin A (compound A) as well as two other putative withanolides (compounds B/C).

a Representative LC-MS chromatograms (TIC) and mass spectrometry data of VIGS samples. Besides withaferin A (compound **A**), two other compounds were classified as putative withanolides based on their similar retention times and high-resolution masses and were also affected by VIGS of withanolide biosynthetic genes. Withaferin A was identified based on an authentic reference compound. **b** Relative quantification of metabolite levels of **A**, **B**, and **C** in silenced plants. For each compound, the mean peak area from the *PDS-GFP* control normalised by the internal standard peak area and sample dry weight was set to 100%. The bar plot shows mean ± SEM and individual data points. For each sample group, six plants were infiltrated; the sample size shown above the bars refers to the number of plants that showed a photobleaching phenotype from *PDS* silencing. Only leaf parts with photobleaching were considered as successfully silenced and analysed. Similar results were observed in two independent experimental batches. Source data are provided as a Source Data file.

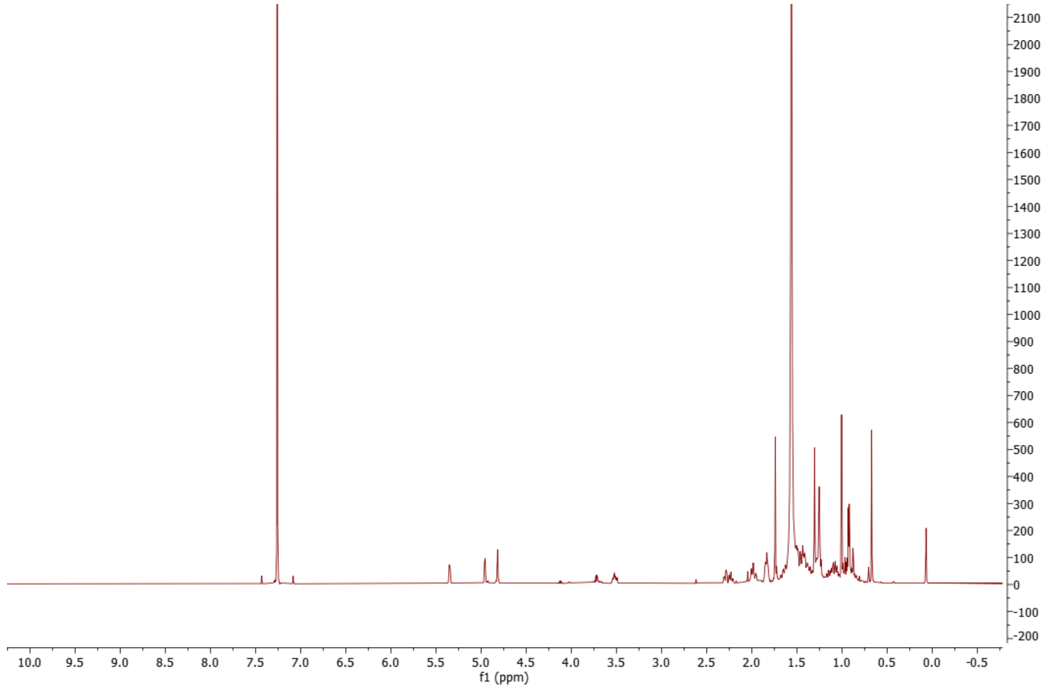

Supplementary Fig. 27. ^{1}H **spectrum of ergosta-5,25-diene-3β,24ξ-diol (physalindicanol A) (7) (CDCl$_3$, 298 K, 600 MHz).**

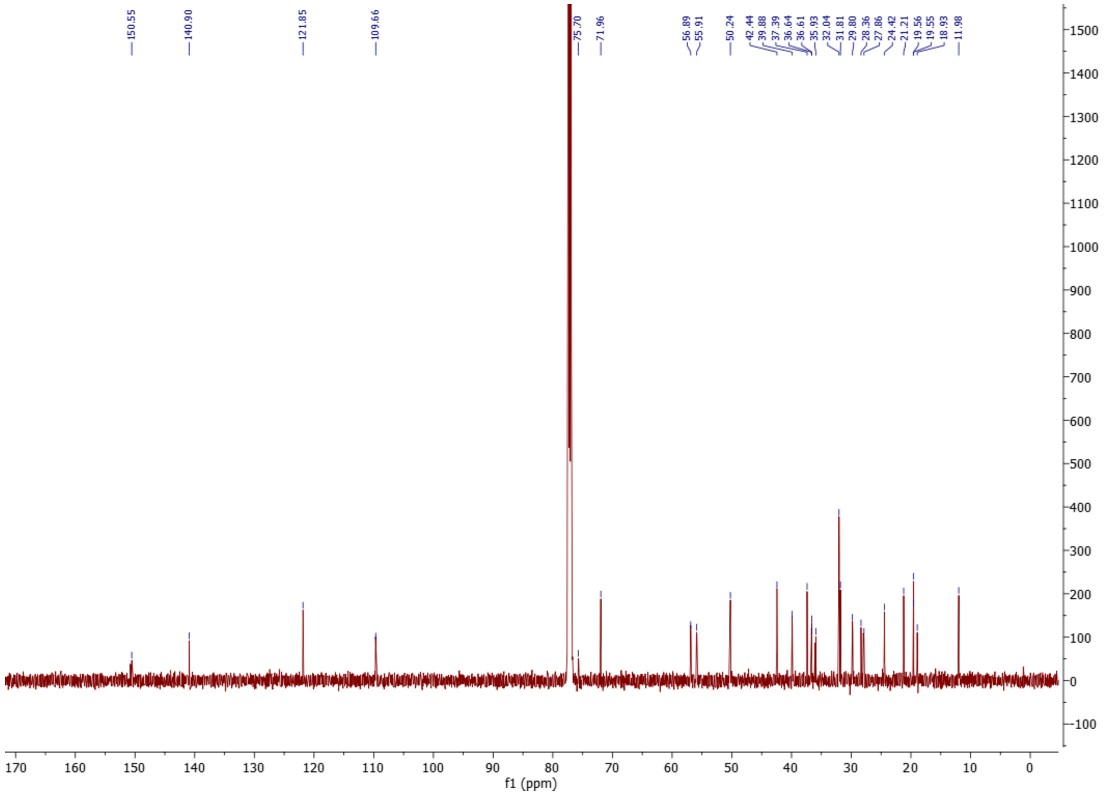

Supplementary Fig. 28. ^{13}C **spectrum of ergosta-5,25-diene-3β,24ξ-diol (physalindicanol A) (7) (CDCl$_3$, 298 K, 151 MHz).**

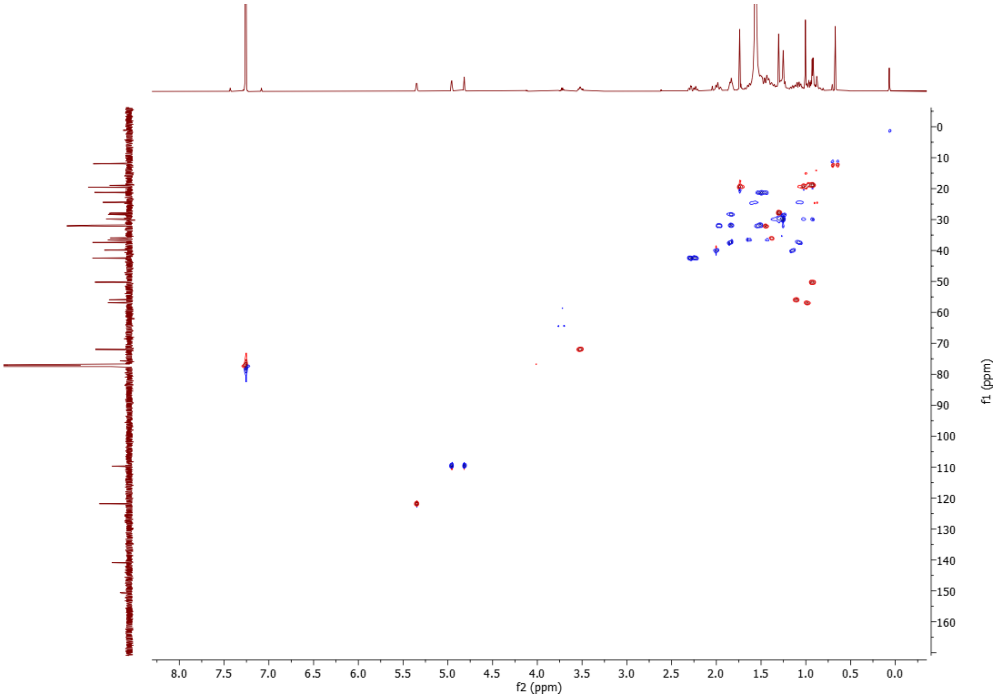

Supplementary Fig. 29. HSQC spectrum of ergosta-5,25-diene-3β,24ξ-diol (physalindicanol A) (7) ($CDCl_3$, 298 K, 600 MHz).

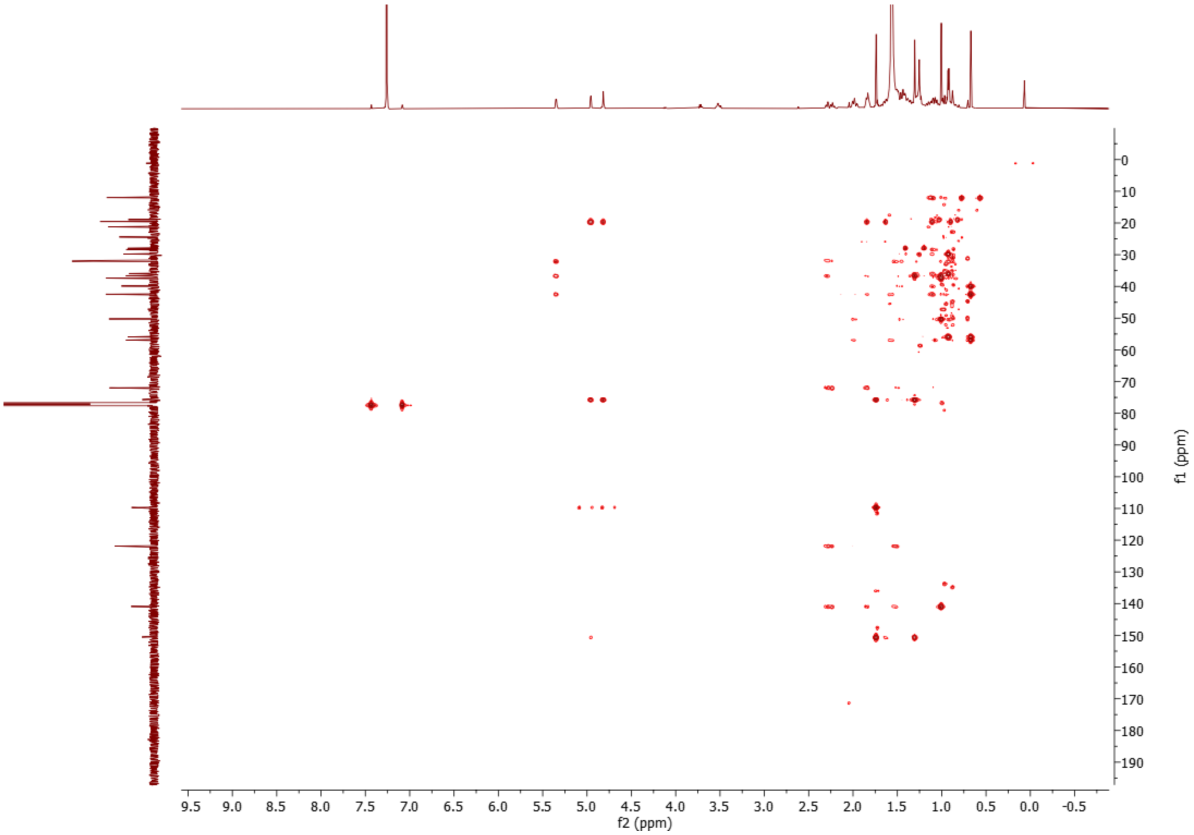

Supplementary Fig. 30. HMBC spectrum of ergosta-5,25-diene-3β,24ξ-diol (physalindicanol A) (7) ($CDCl_3$, 298 K, 600 MHz).

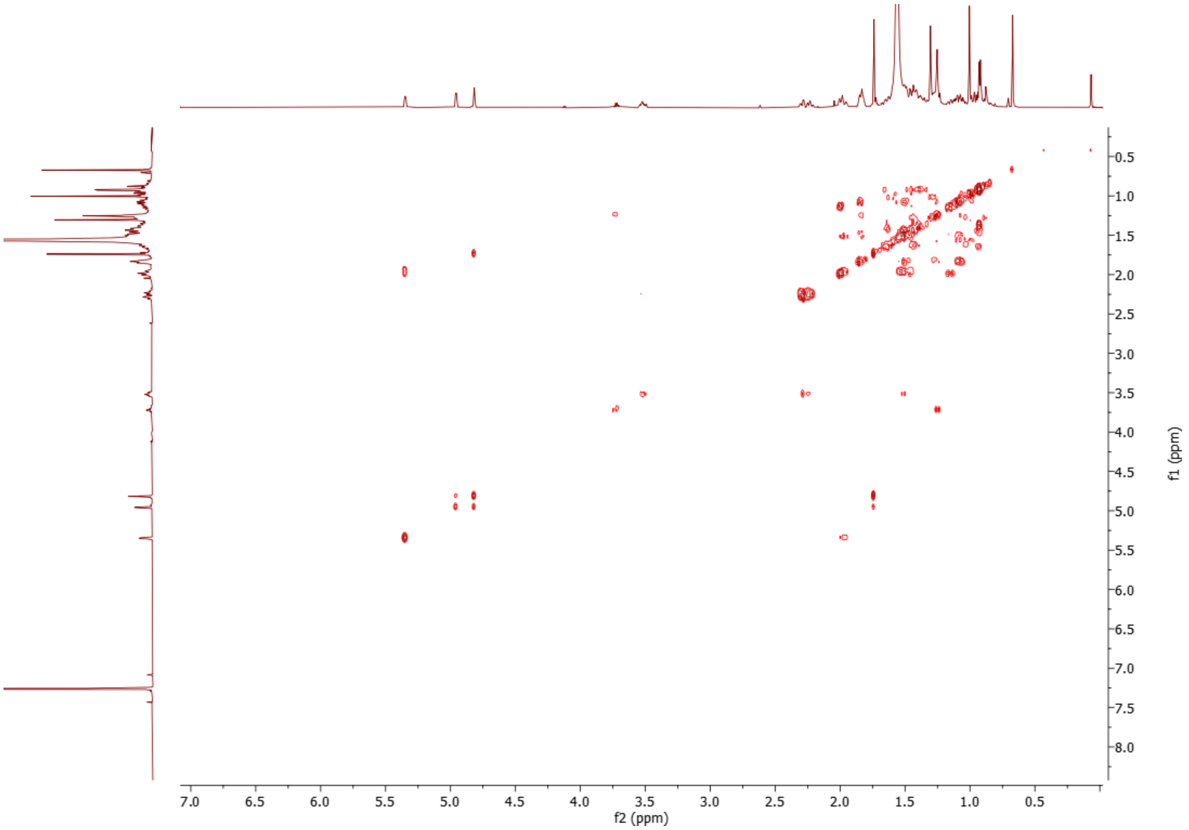

Supplementary Fig. 31. COSY spectrum of ergosta-5,25-diene-3β,24ξ-diol (physalindicanol A) (7) ($CDCl_3$, 298 K, 600 MHz).

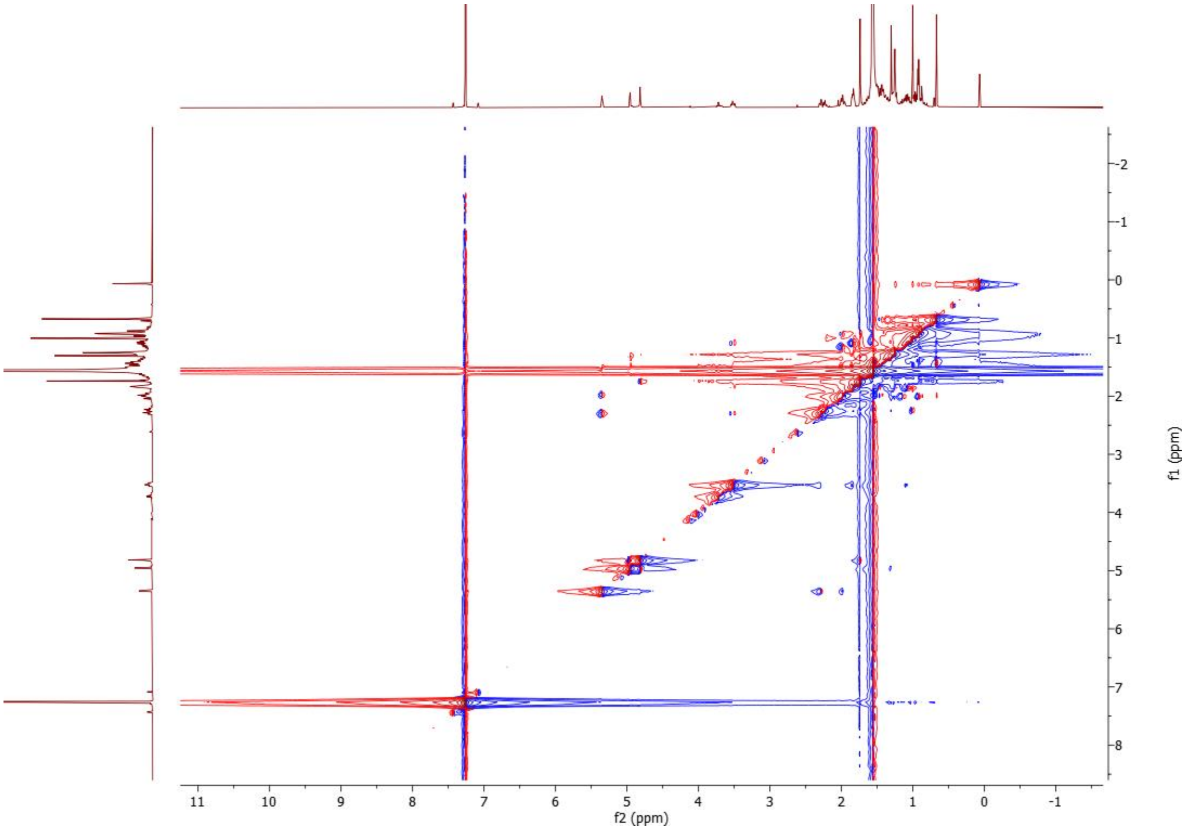

Supplementary Fig. 32. NOESY spectrum of ergosta-5,25-diene-3β,24ξ-diol (physalindicanol A) (7) ($CDCl_3$, 298 K, 600 MHz).

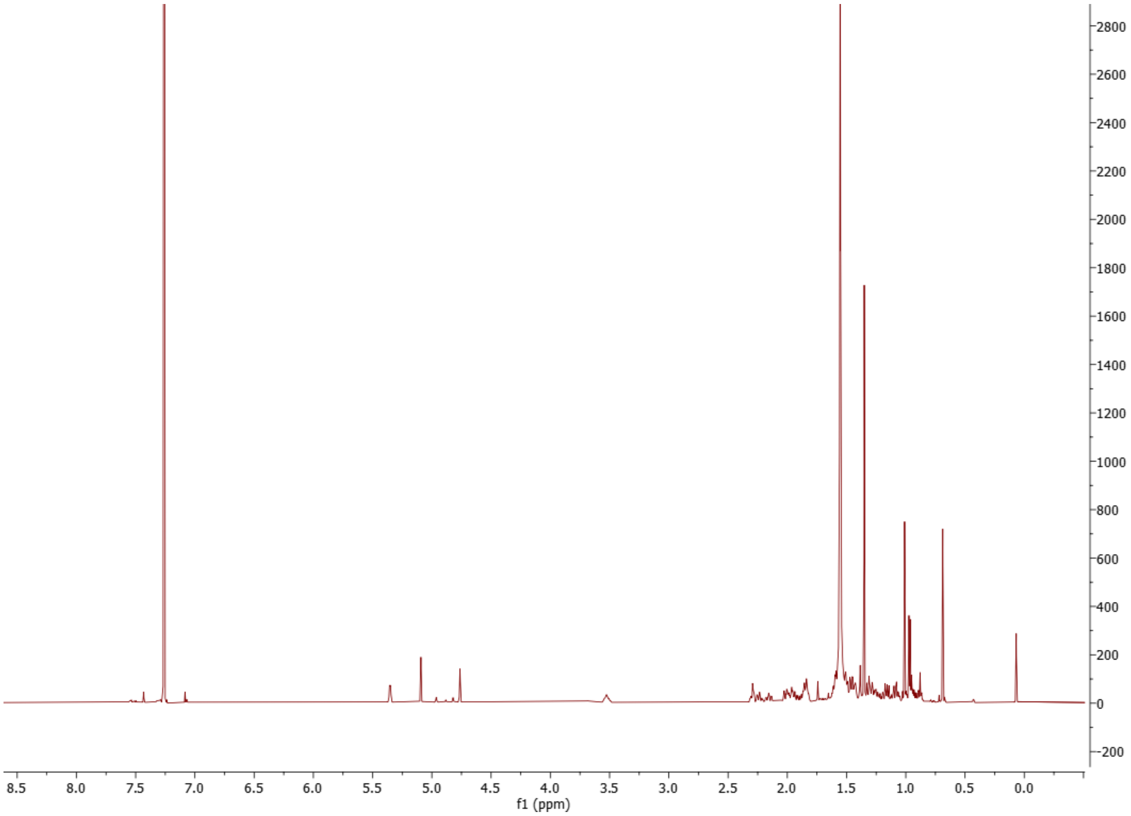

Supplementary Fig. 33. ^{1}H spectrum of ergosta-5,24(28)-diene-3β,25-diol (8) (physalindicanol B) ($CDCl_3$, 298 K, 600 MHz).

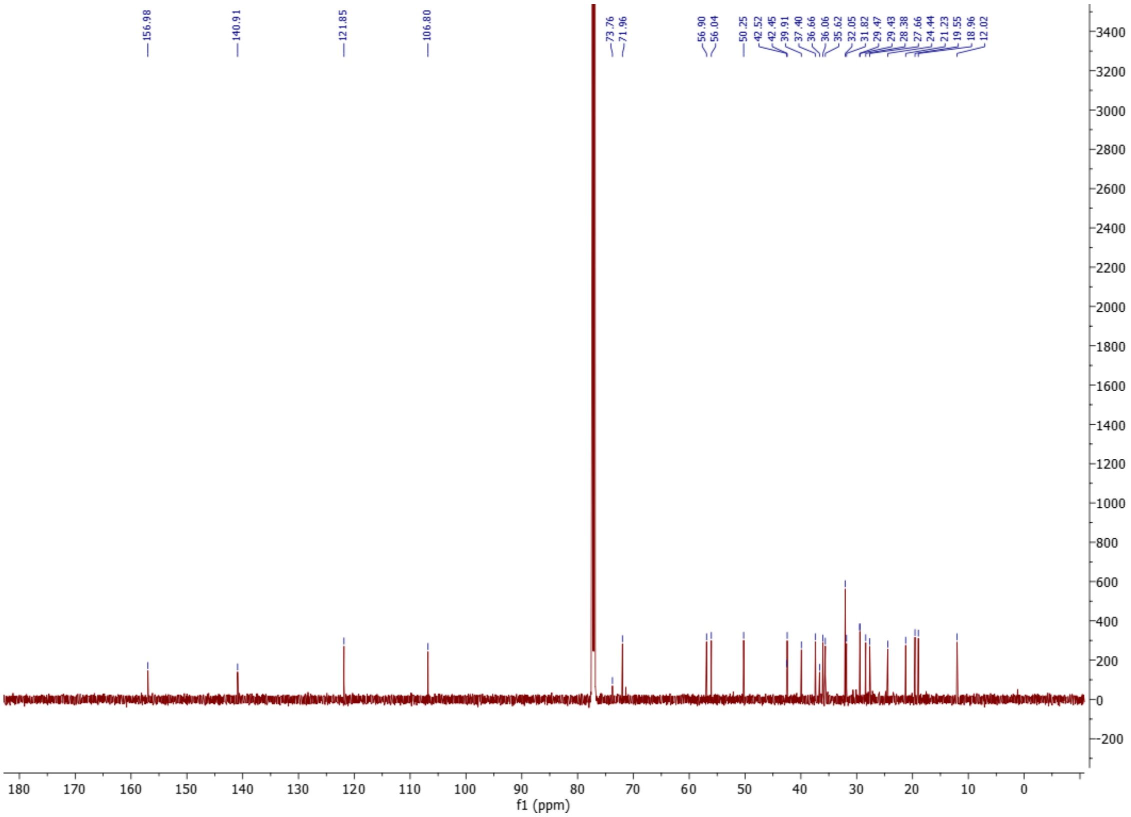

Supplementary Fig. 34. ^{13}C spectrum of ergosta-5,24(28)-diene-3β,25-diol (8) (physalindicanol B) ($CDCl_3$, 298 K, 151 MHz).

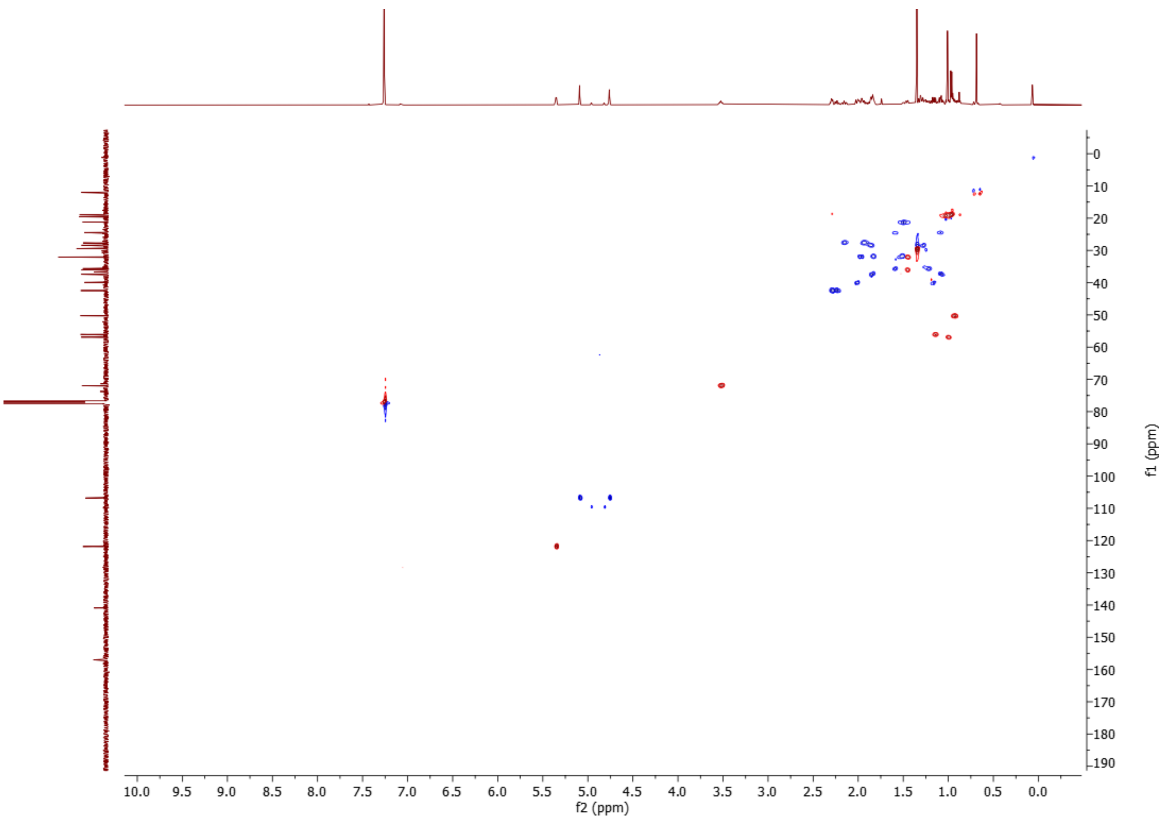

Supplementary Fig. 35. HSQC spectrum of ergosta-5,24(28)-diene-3β,25-diol (8) (physalindicanol B) ($CDCl_3$, 298 K, 600 MHz).

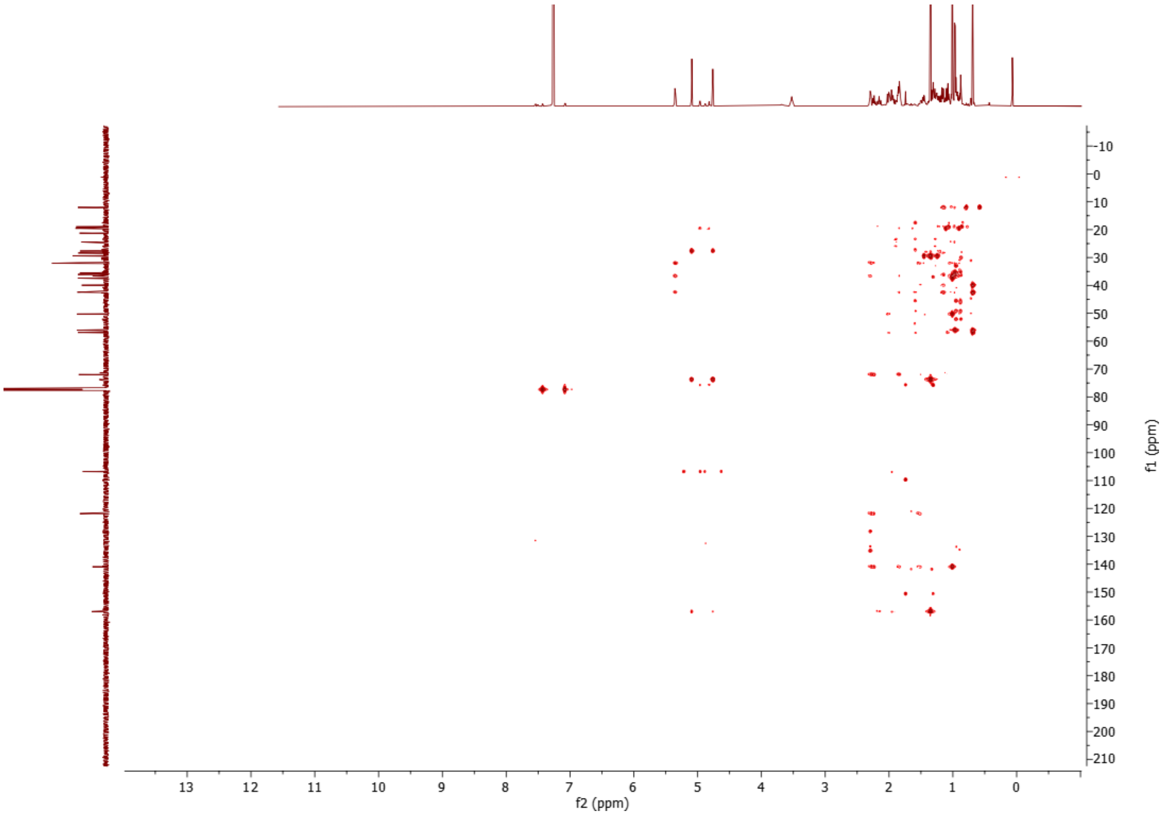

Supplementary Fig. 36. HMBC spectrum of ergosta-5,24(28)-diene-3β,25-diol (8) (physalindicanol B) ($CDCl_3$, 298 K, 600 MHz).

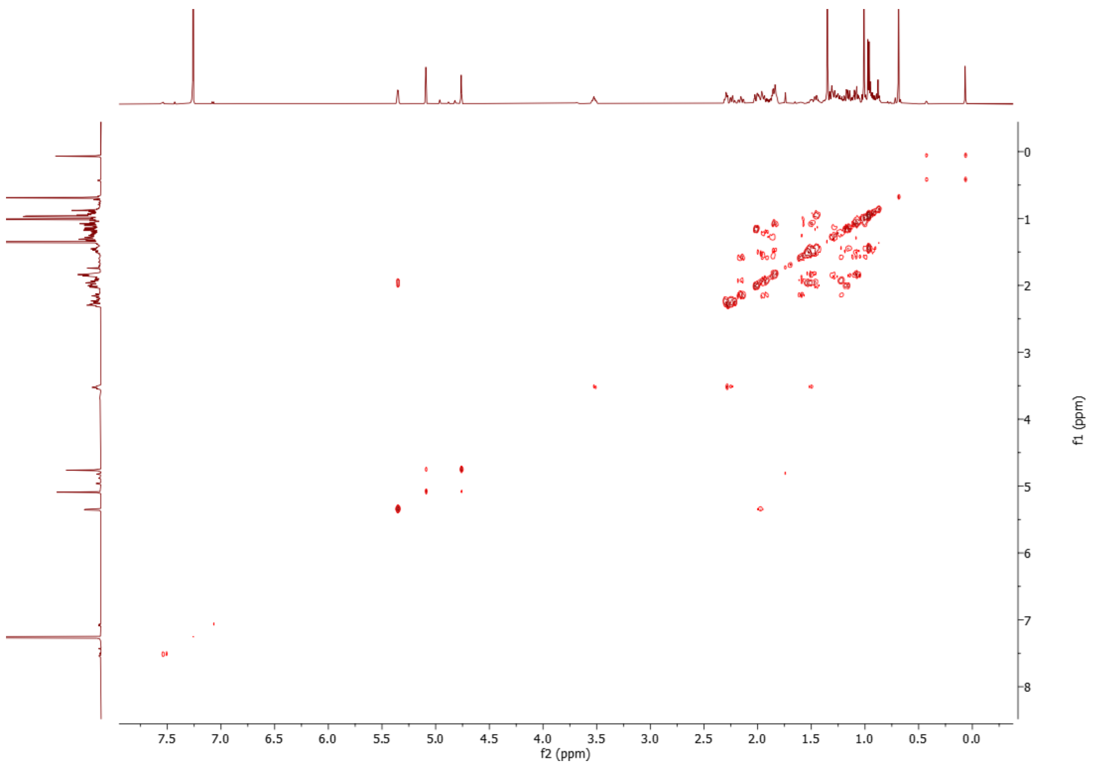

Supplementary Fig. 37. COSY spectrum of ergosta-5,24(28)-diene-3β,25-diol (8) (physalindicanol B) ($CDCl_3$, 298 K, 600 MHz).

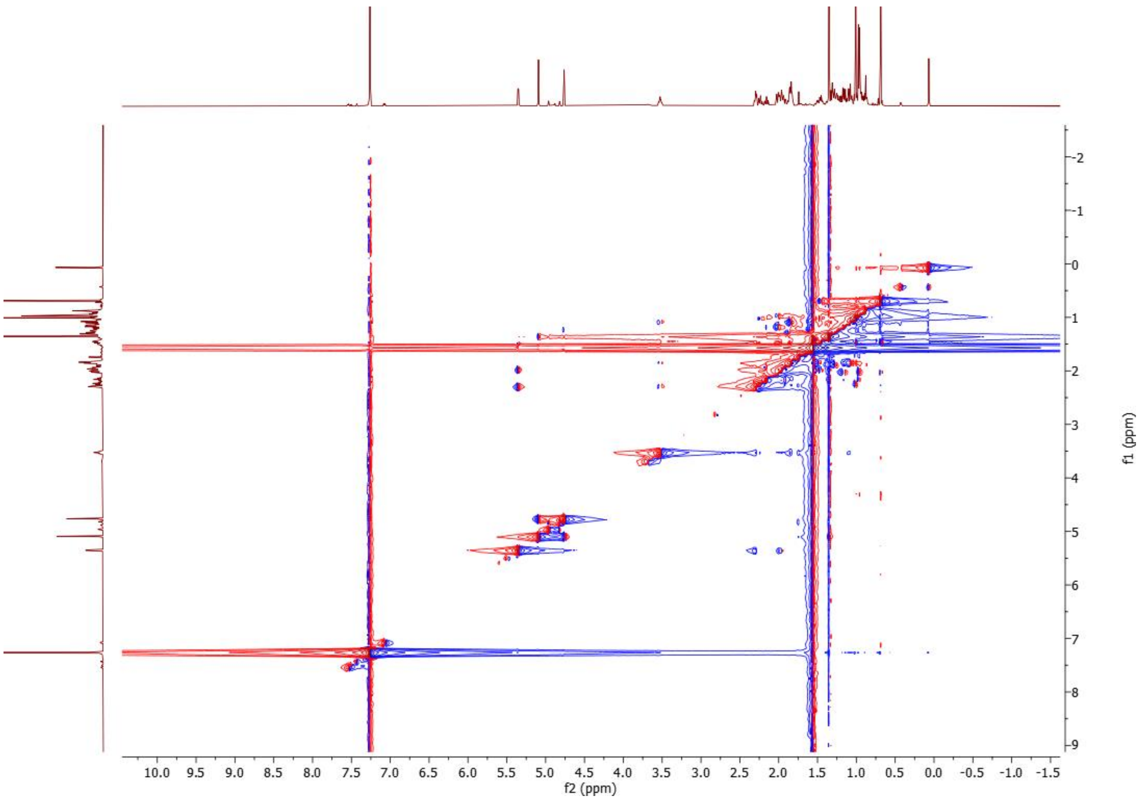

Supplementary Fig. 38. NOESY spectrum of ergosta-5,24(28)-diene-3β,25-diol (8) (physalindicanol B) ($CDCl_3$, 298 K, 600 MHz).

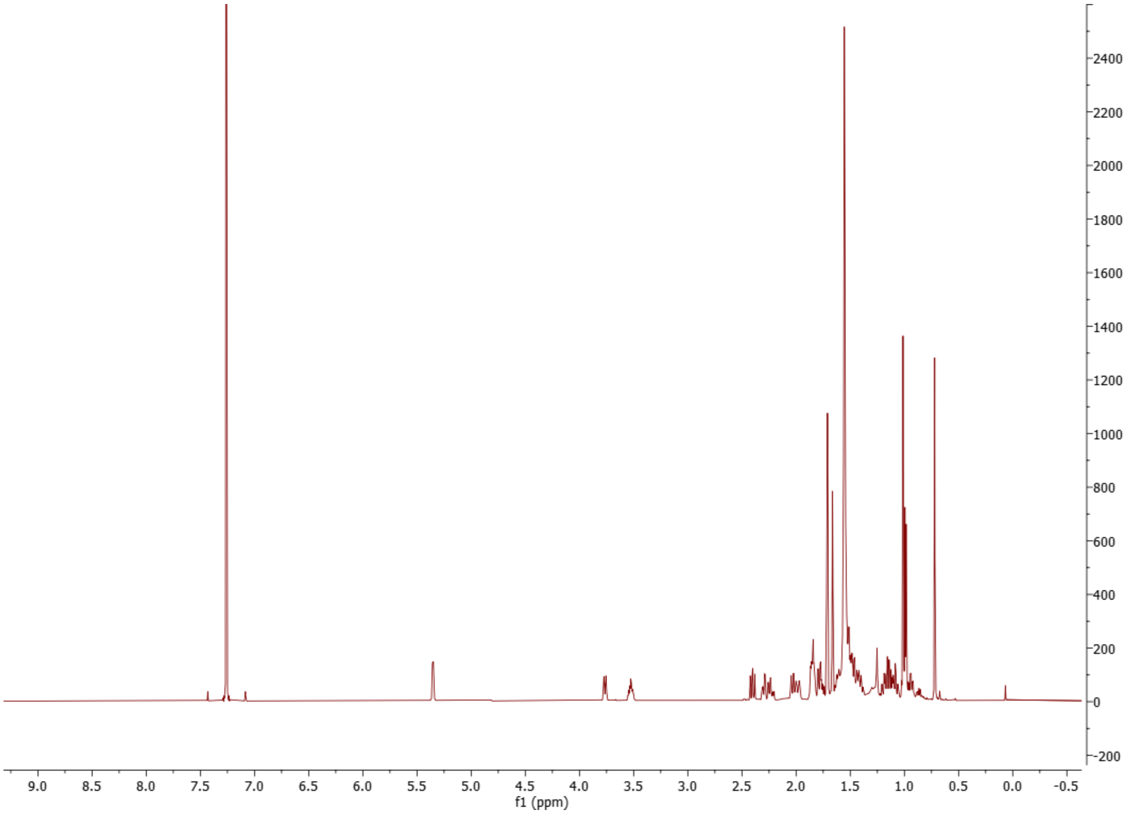

Supplementary Fig. 39. ^{1}H spectrum of (22*R*)-ergosta-5,24-diene-3β,22-diol (10) ($CDCl_3$, 298 K, 600 MHz).

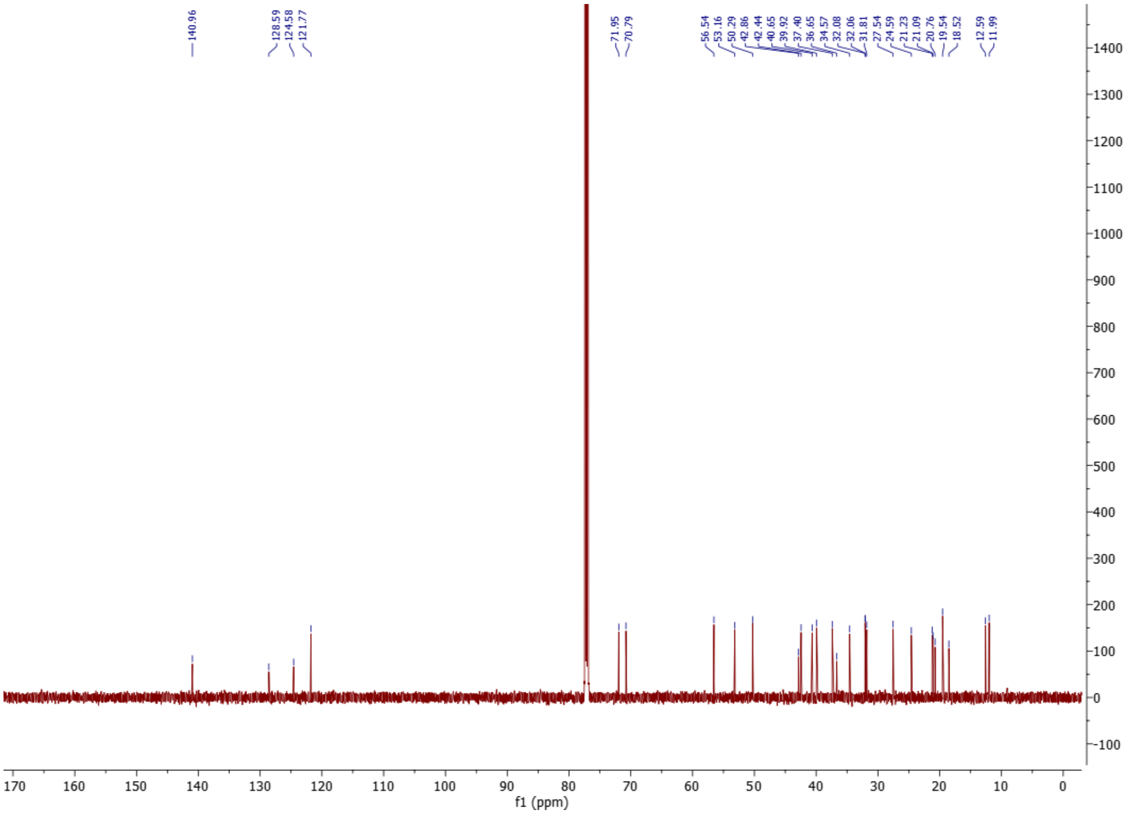

Supplementary Fig. 40. ^{13}C spectrum of (22*R*)-ergosta-5,24-diene-3β,22-diol (10) ($CDCl_3$, 298 K, 151 MHz).

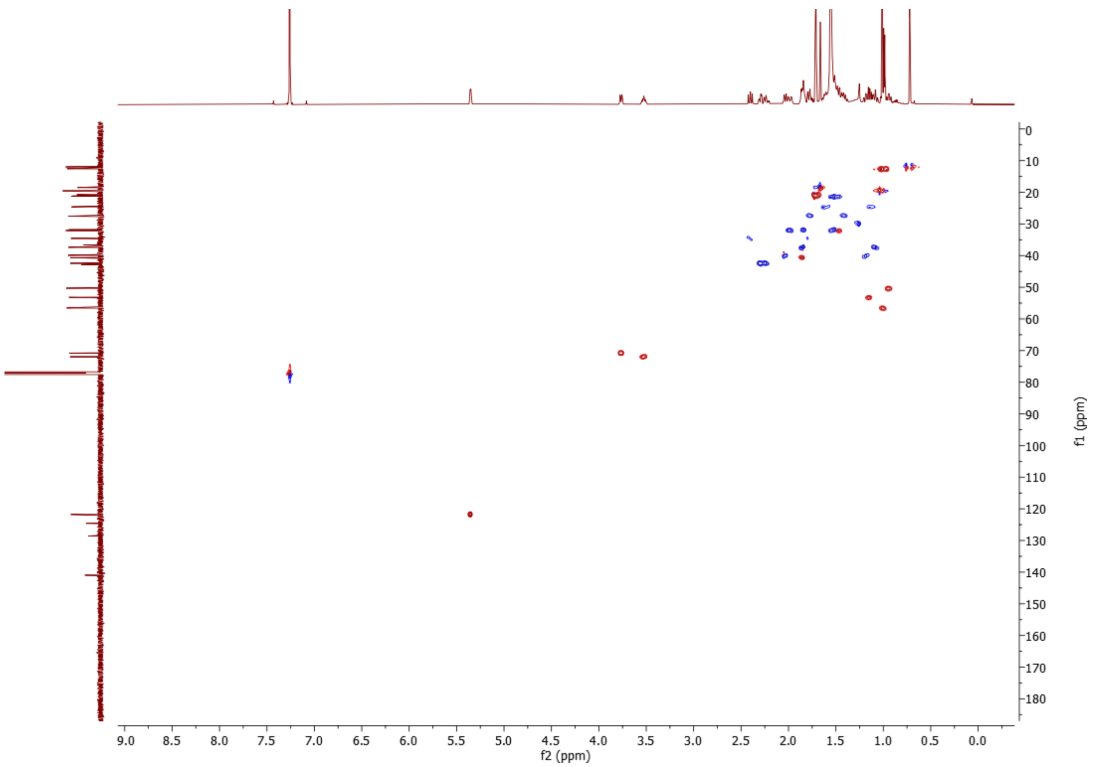

Supplementary Fig. 41. HSQC spectrum of (22*R*)-ergosta-5,24-diene-3β,22-diol (10) ($CDCl_3$, 298 K, 600 MHz).

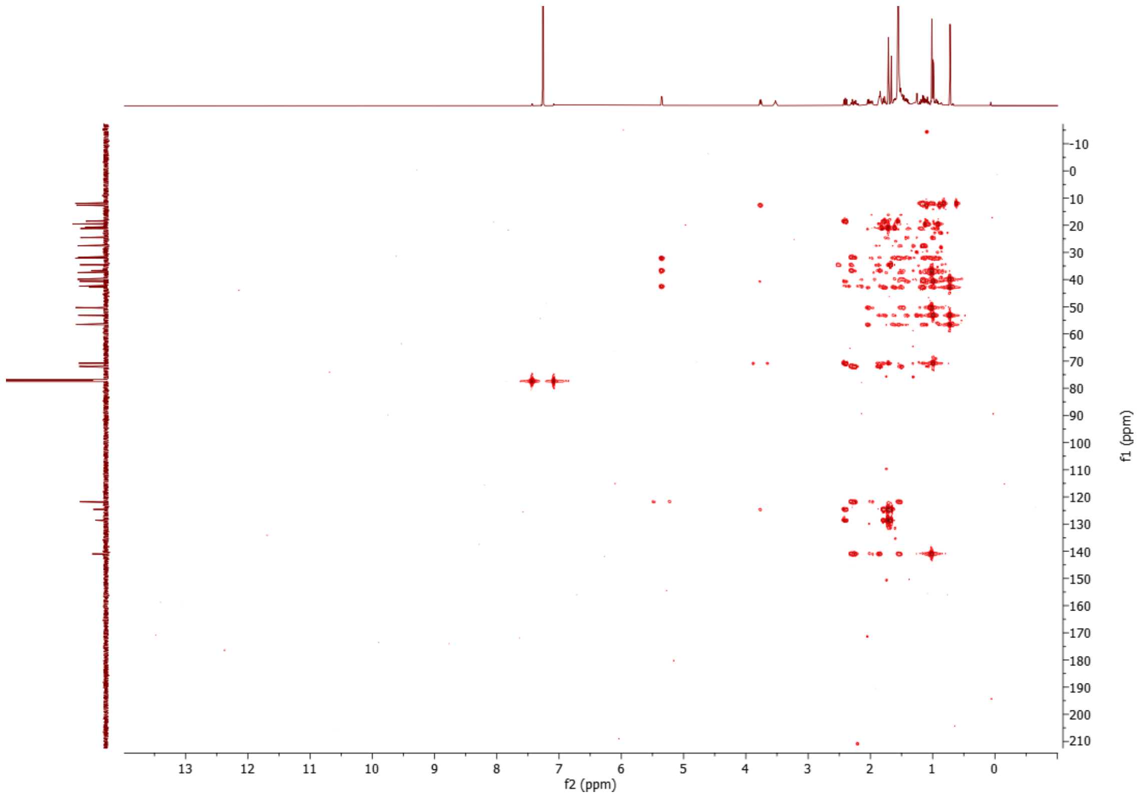

Supplementary Fig. 42. HMBC spectrum of (22*R*)-ergosta-5,24-diene-3β,22-diol (10) ($CDCl_3$, 298 K, 600 MHz).

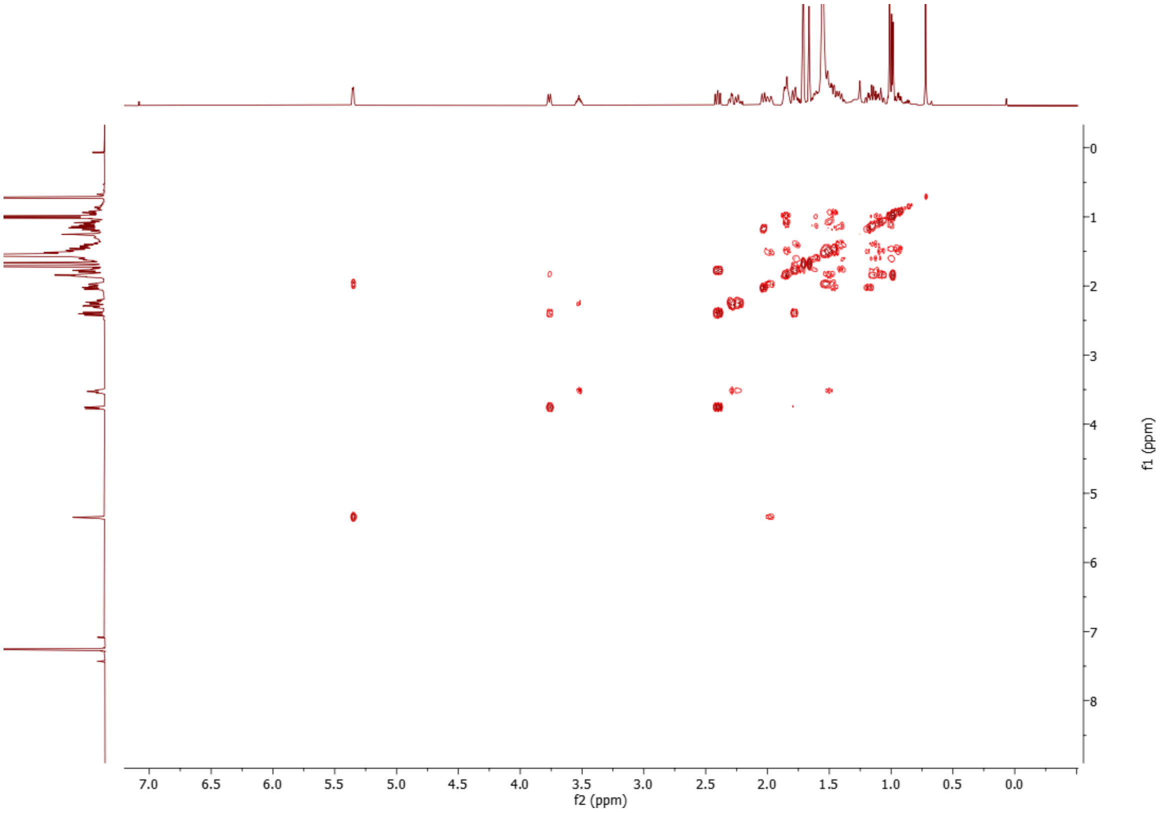

Supplementary Fig. 43. COSY spectrum of (22*R*)-ergosta-5,24-diene-3β,22-diol (10) ($CDCl_3$, 298 K, 600 MHz).

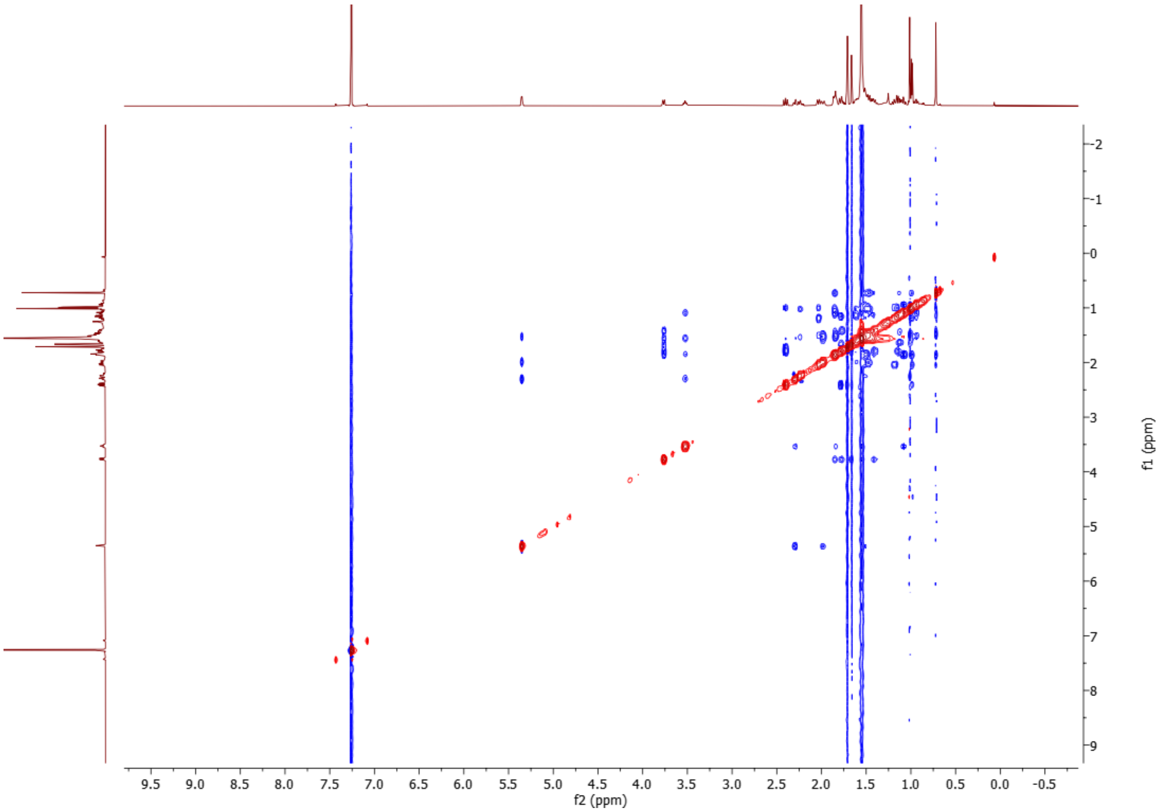

Supplementary Fig. 44. NOESY spectrum of (22*R*)-ergosta-5,24-diene-3β,22-diol (10) ($CDCl_3$, 298 K, 600 MHz).

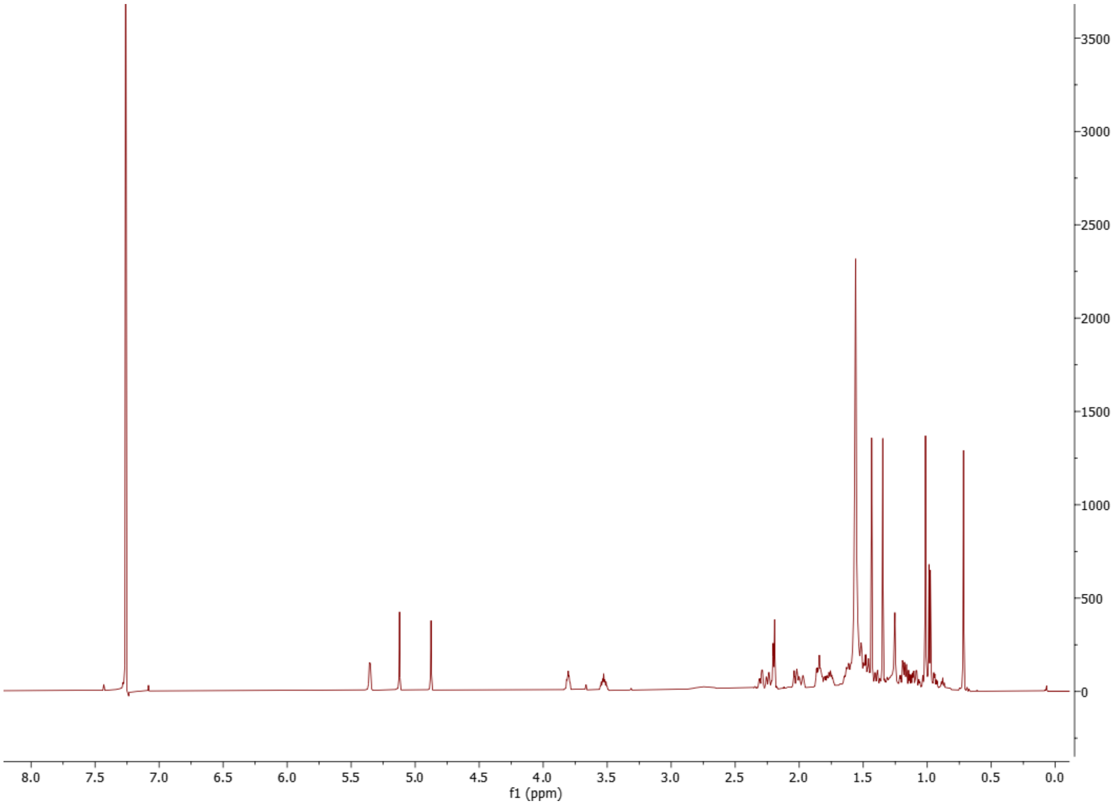

Supplementary Fig. 45. ¹H spectrum of (22*R*)-ergosta-5,24(28)-diene-3β,22,25-triol (phyministerol A) (12) ($CDCl_3$, 298 K, 600 MHz).

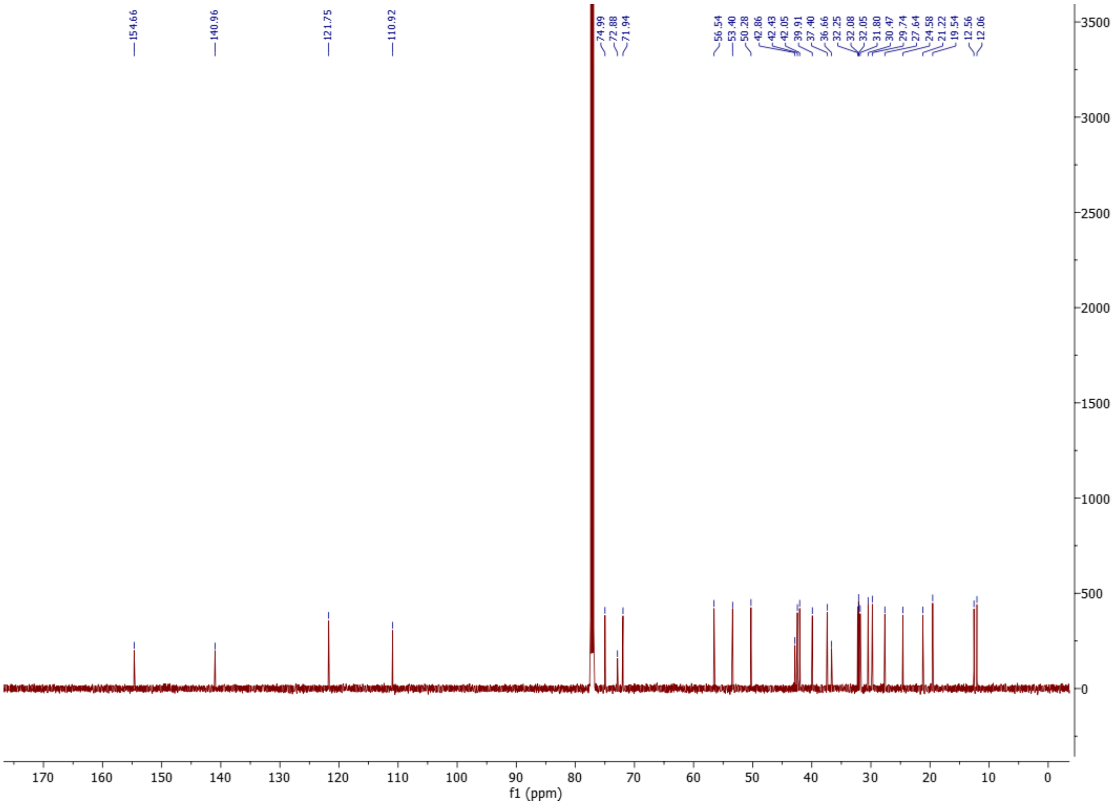

Supplementary Fig. 46. ¹³C spectrum of (22*R*)-ergosta-5,24(28)-diene-3β,22,25-triol (phyministerol A) (12) ($CDCl_3$, 298 K, 151 MHz).

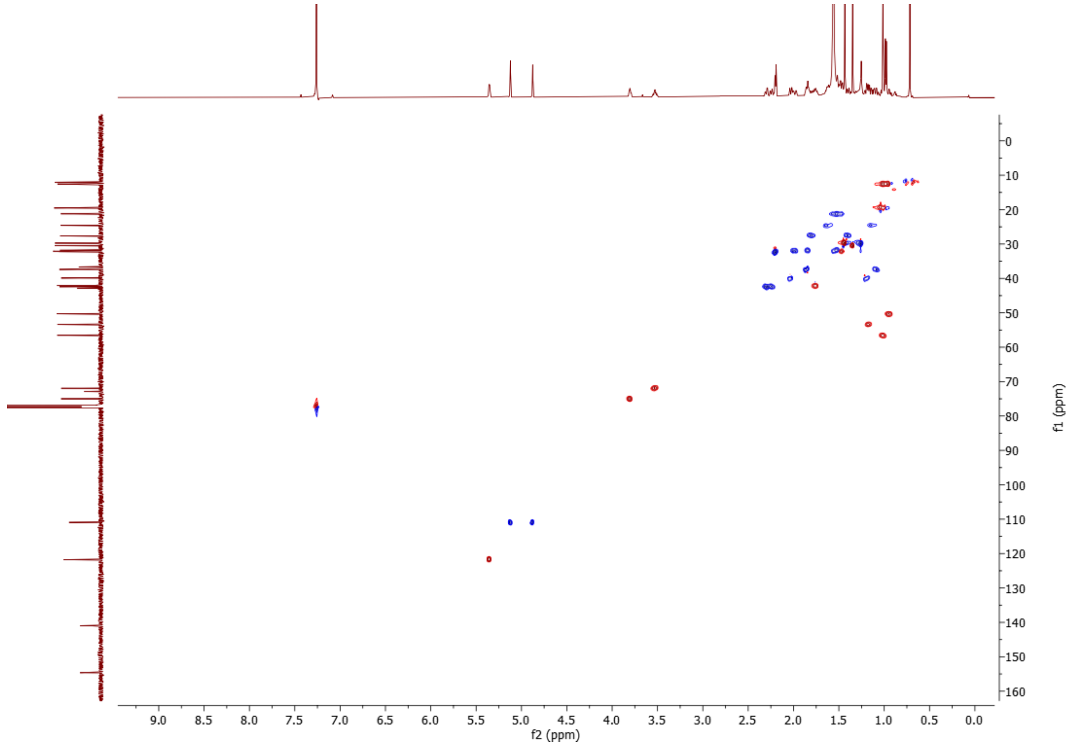

Supplementary Fig. 47. HSQC spectrum of (22*R*)-ergosta-5,24(28)-diene-3β,22,25-triol (phyministerol A) (12) ($CDCl_3$, 298 K, 600 MHz).

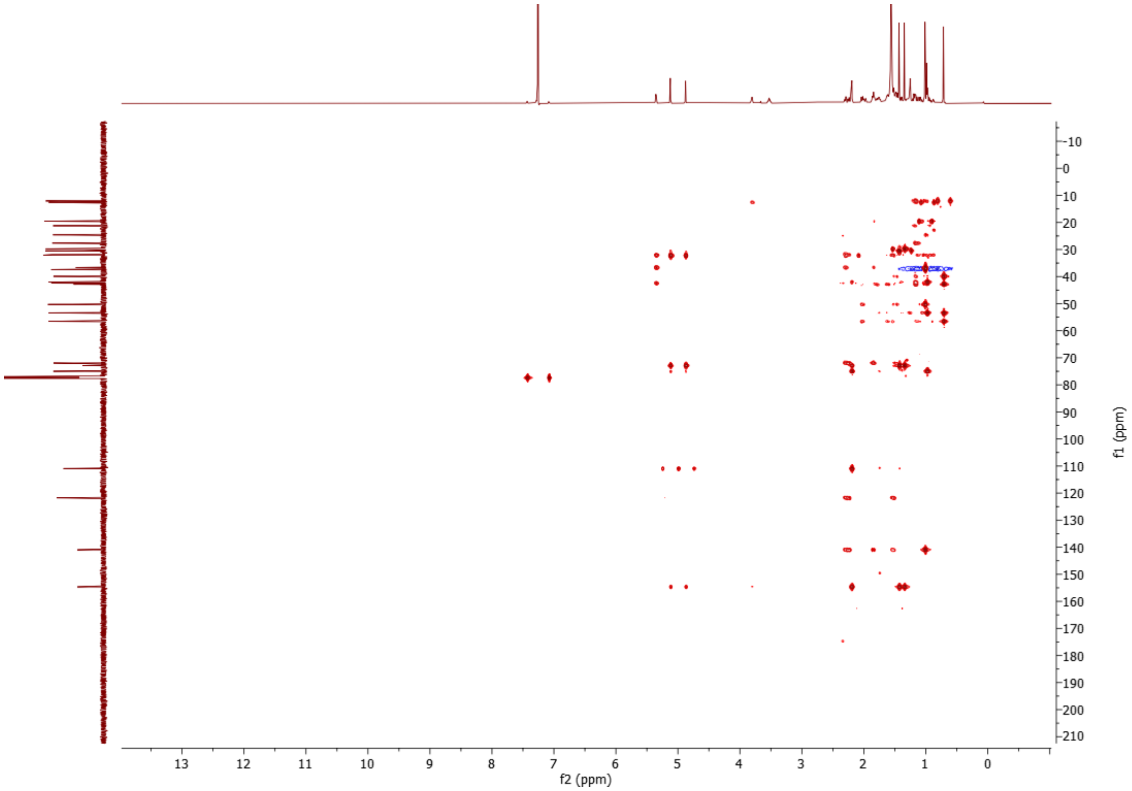

Supplementary Fig. 48. HMBC spectrum of (22*R*)-ergosta-5,24(28)-diene-3β,22,25-triol (phyministerol A) (12) ($CDCl_3$, 298 K, 600 MHz).

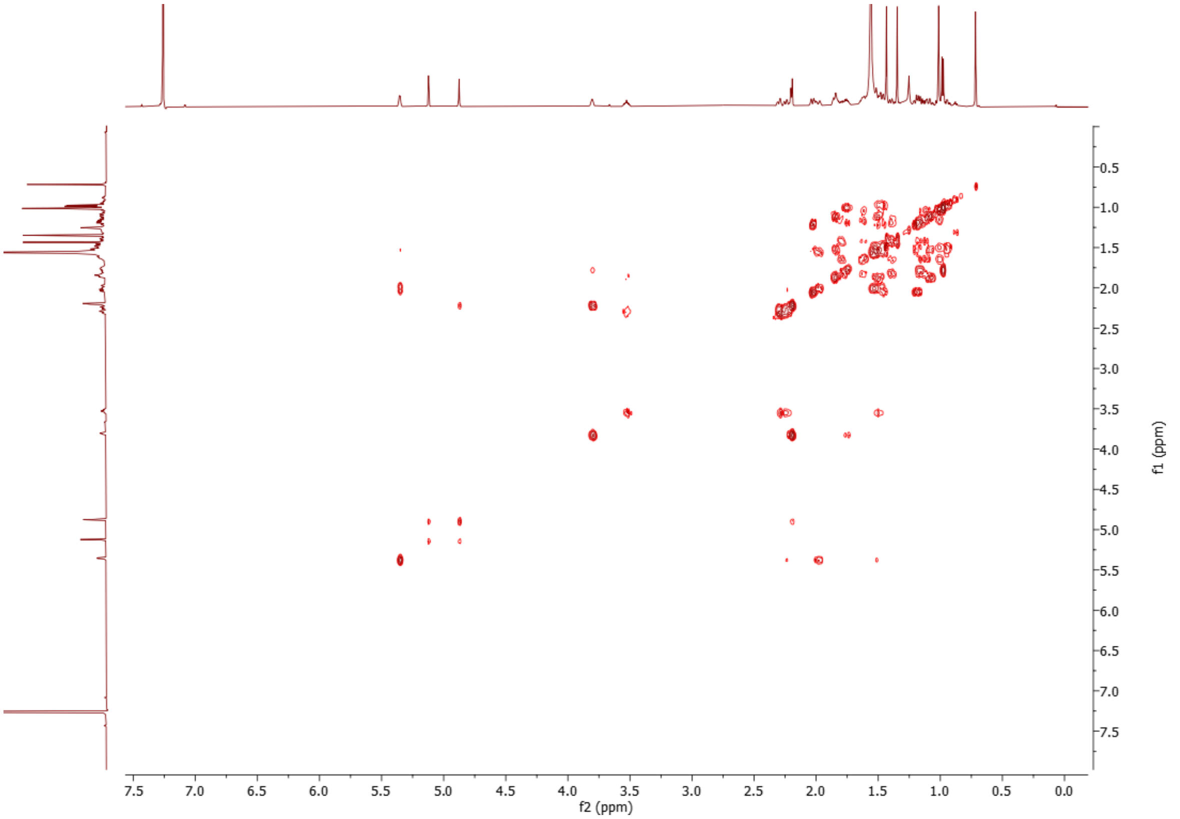

Supplementary Fig. 49. COSY spectrum of (22*R*)-ergosta-5,24(28)-diene-3β,22,25-triol (phyministerol A) (12) ($CDCl_3$, 298 K, 600 MHz).

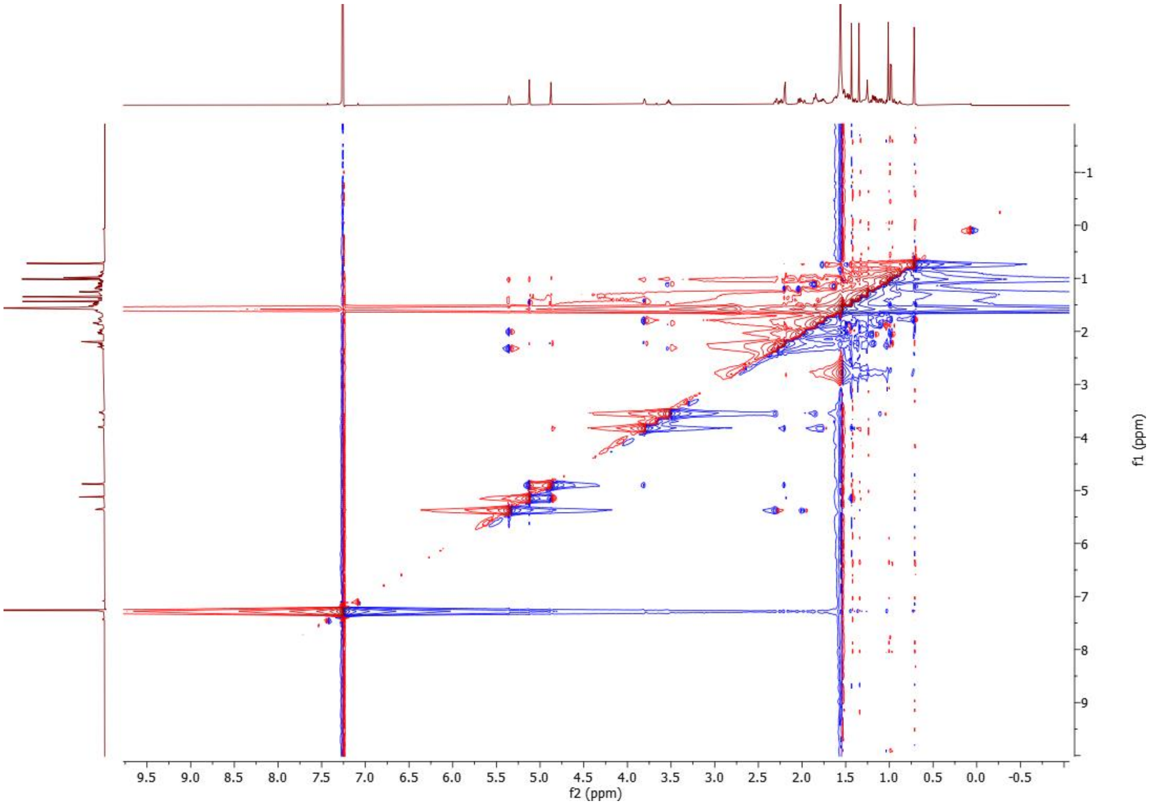

Supplementary Fig. 50. NOESY spectrum of (22*R*)-ergosta-5,24(28)-diene-3β,22,25-triol (phyministerol A) (12) ($CDCl_3$, 298 K, 600 MHz).

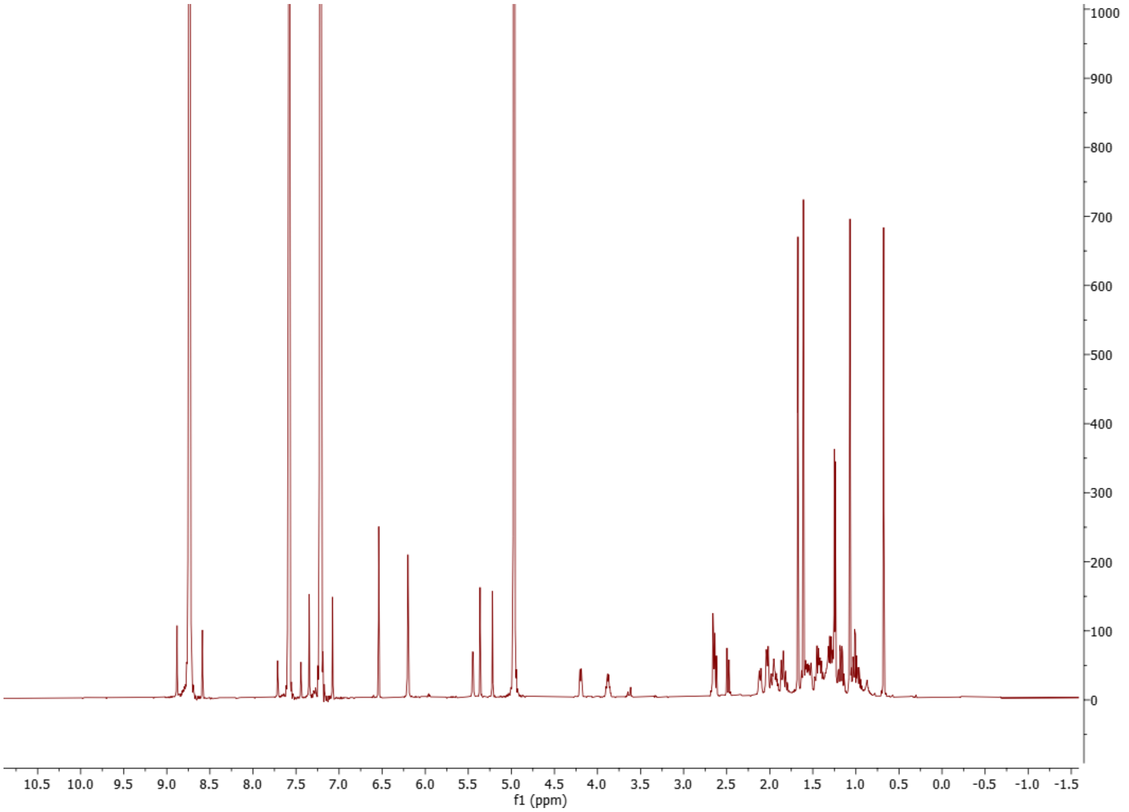

Supplementary Fig. 51. ^{1}**H spectrum of (22*R*)-ergosta-5,24(28)-diene-3β,22,25-triol (phyministerol A) (12) (600 MHz,** C_5D_5N**, 298 K).**

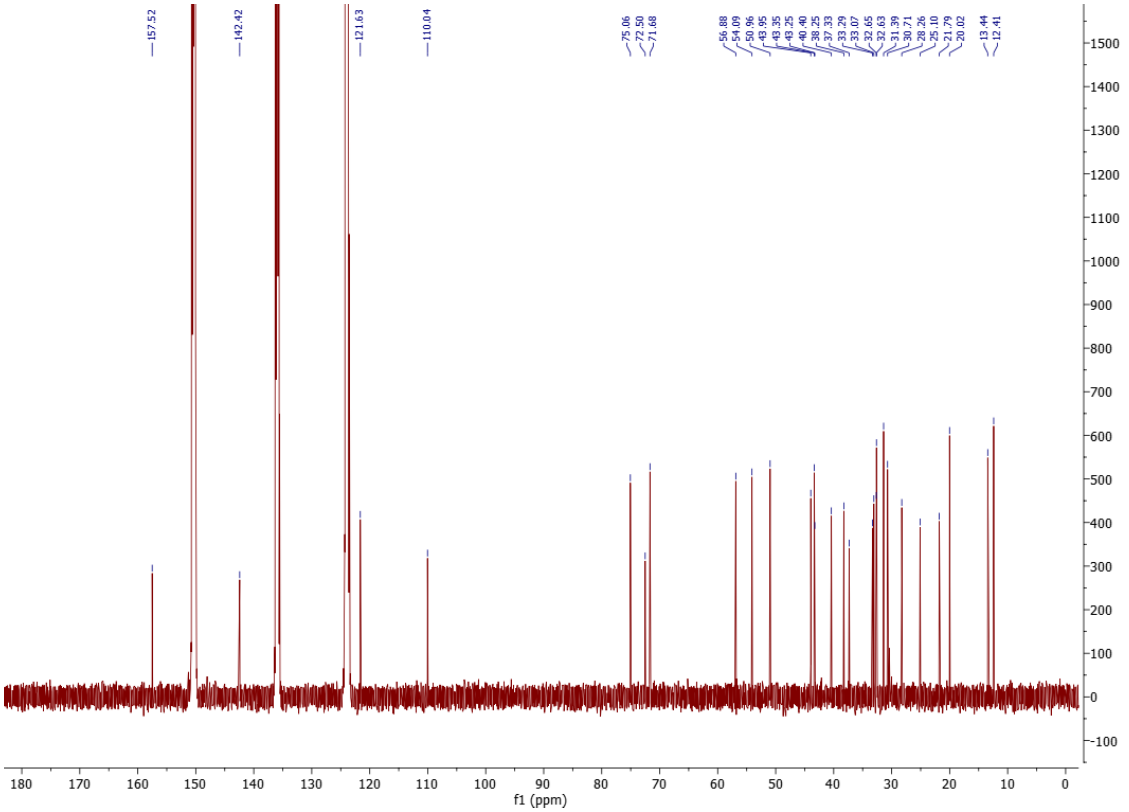

Supplementary Fig. 52. ^{13}**C spectrum of (22*R*)-ergosta-5,24(28)-diene-3β,22,25-triol (phyministerol A) (12) (151 MHz,** C_5D_5N**, 298 K).**

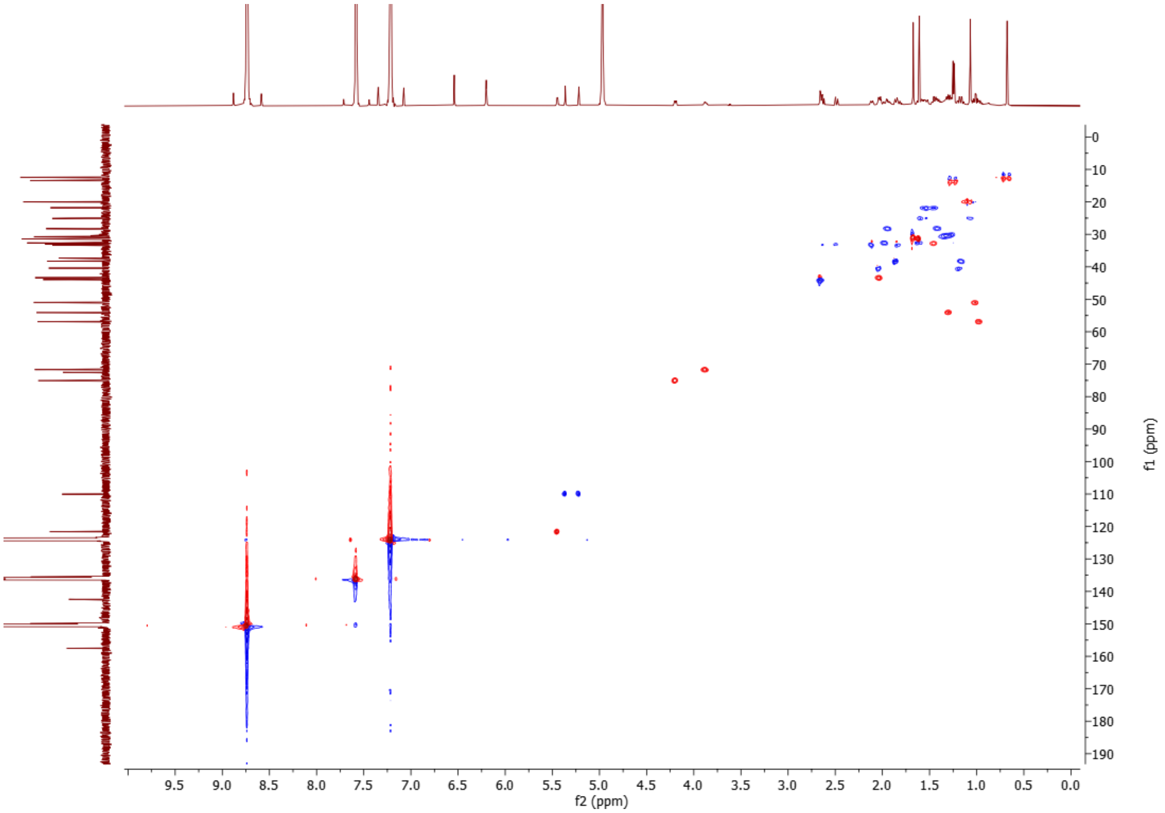

Supplementary Fig. 53. HSQC spectrum of (22*R*)-ergosta-5,24(28)-diene-3β,22,25-triol (phyministerol A) (12) (600 MHz, C_5D_5N, 298 K).

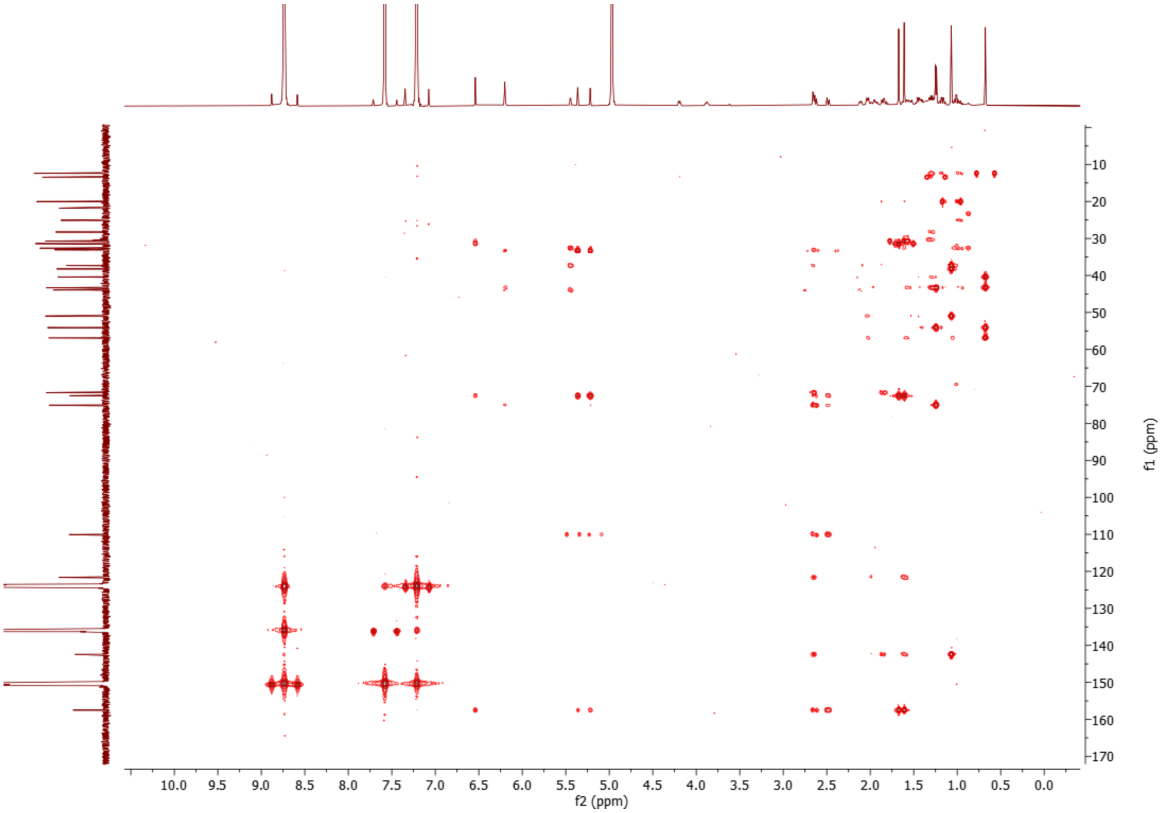

Supplementary Fig. 54. HMBC spectrum of (22*R*)-ergosta-5,24(28)-diene-3β,22,25-triol (phyministerol A) (12) (600 MHz, C_5D_5N, 298 K).

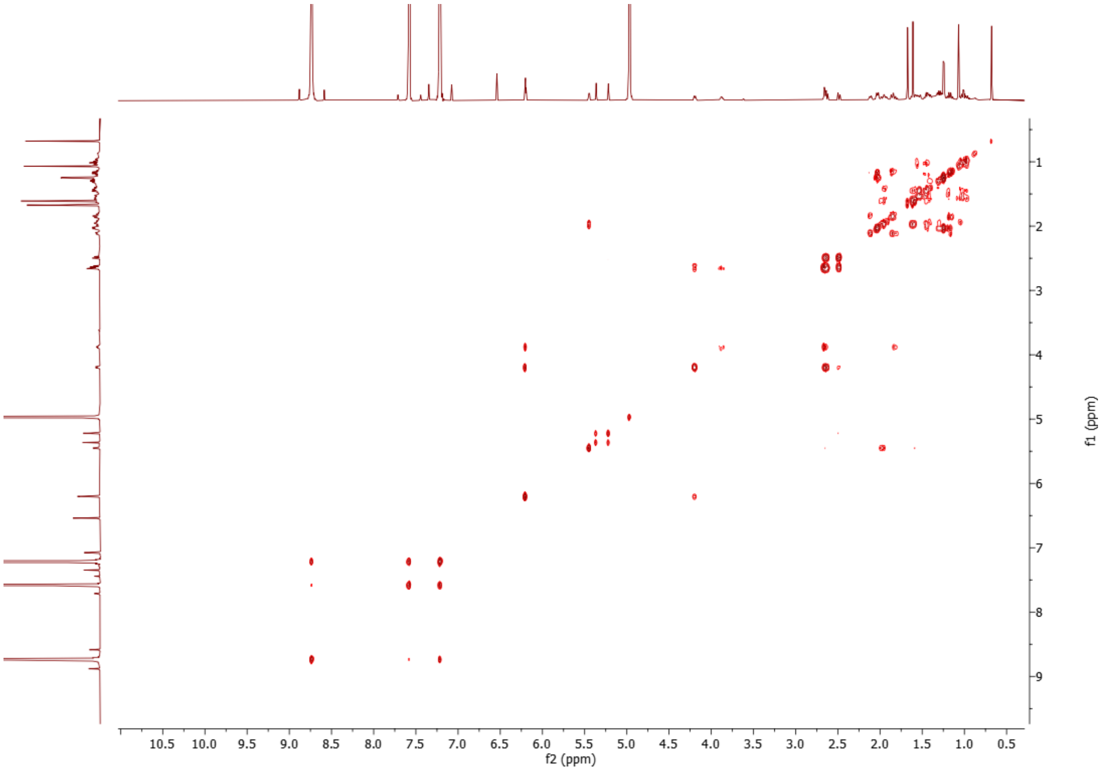

Supplementary Fig. 55. COSY spectrum of (22*R*)-ergosta-5,24(28)-diene-3β,22,25-triol (phyministerol A) (12) (600 MHz, C_5D_5N, 298 K).

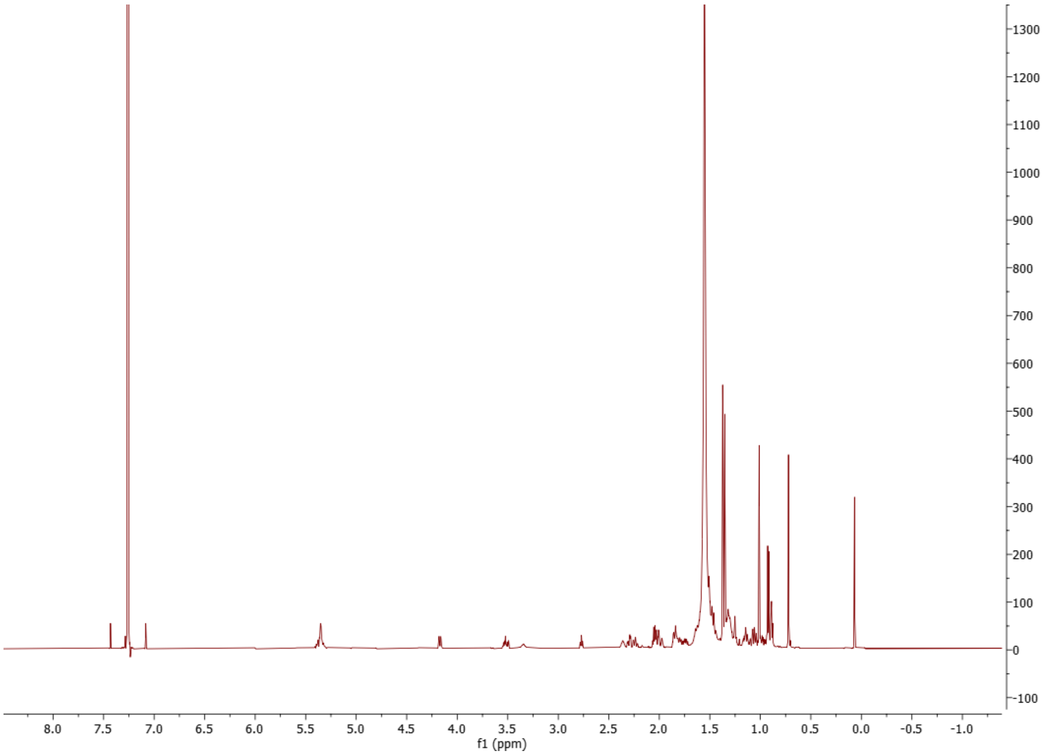

Supplementary Fig. 56. ^{1}H spectrum of (22*R*)-24,25-epoxy-ergost-5-ene-3β,22-diol (13) ($CDCl_3$, 298 K, 600 MHz).

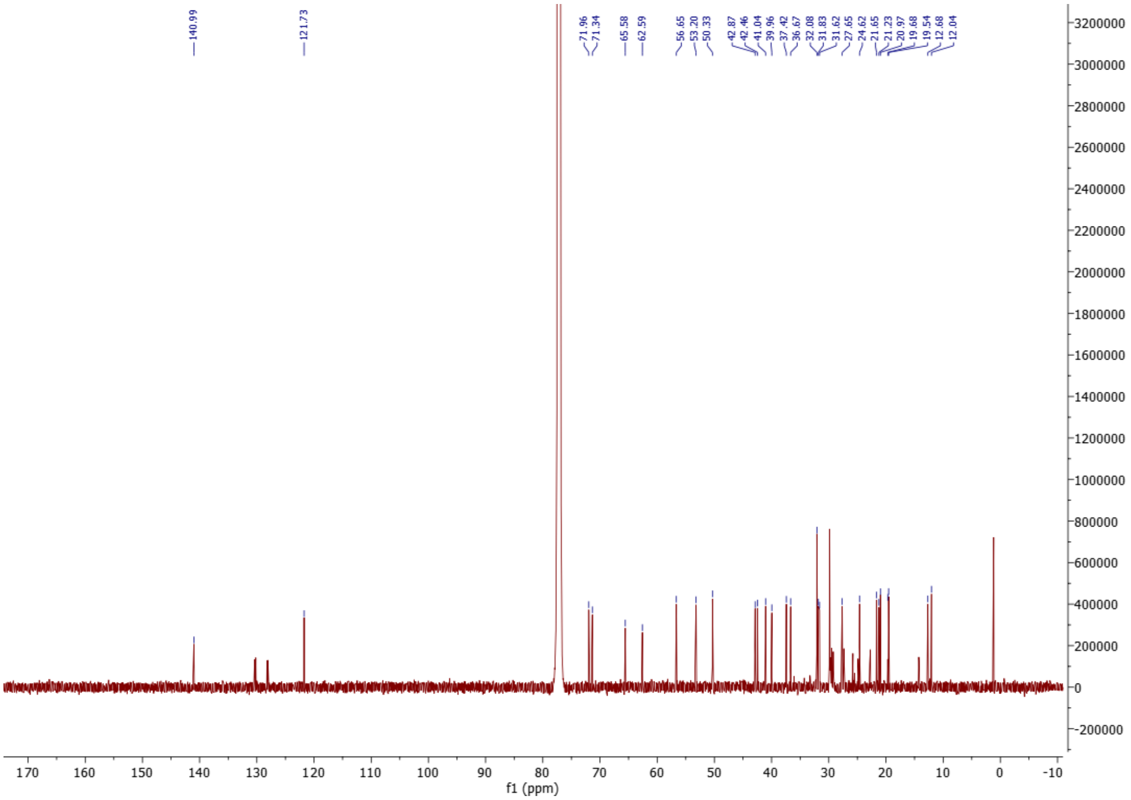

Supplementary Fig. 57. ^{13}C **spectrum of (22*R*)-24,25-epoxy-ergost-5-ene-3β,22-diol (13) ($CDCl_3$, 298 K, 151 MHz).**

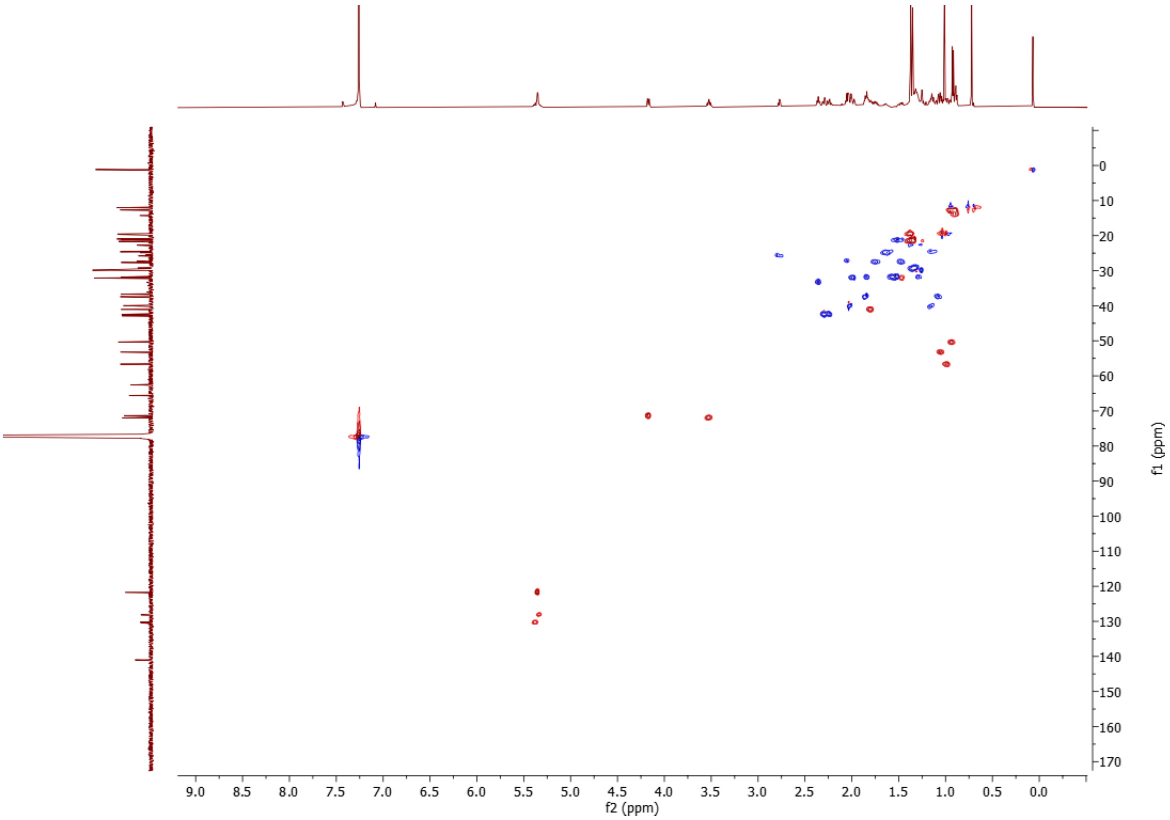

Supplementary Fig. 58. HSQC spectrum of (22*R*)-24,25-epoxy-ergost-5-ene-3β,22-diol (13) ($CDCl_3$, 298 K, 600 MHz).

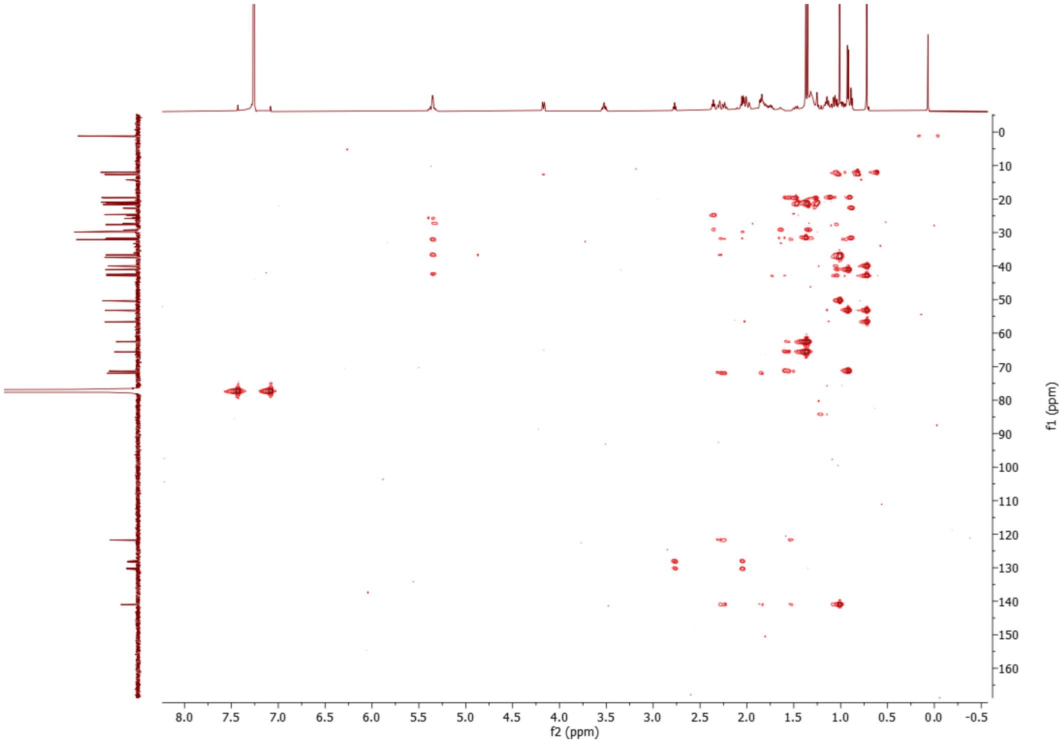

Supplementary Fig. 59. HMBC spectrum of (22*R*)-24,25-epoxy-ergost-5-ene-3β,22-diol (13) ($CDCl_3$, 298 K, 600 MHz).

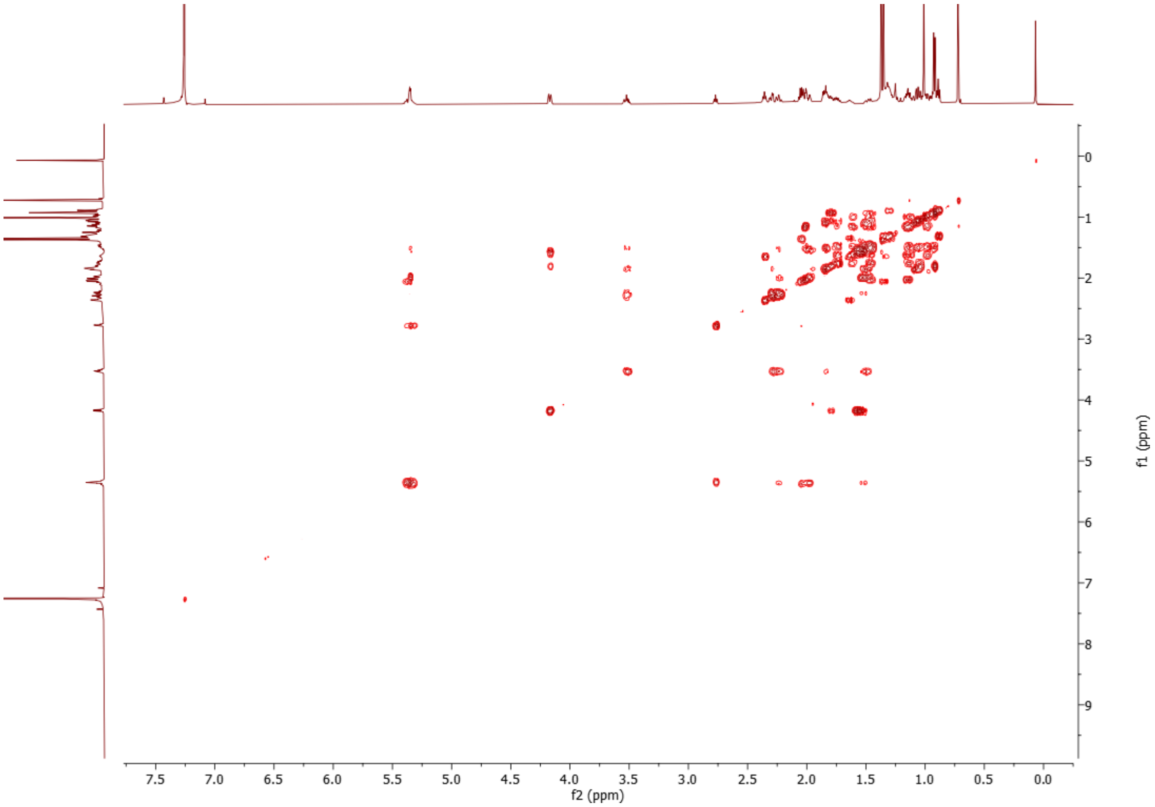

Supplementary Fig. 60. COSY spectrum of (22*R*)-24,25-epoxy-ergost-5-ene-3β,22-diol (13) ($CDCl_3$, 298 K, 600 MHz).

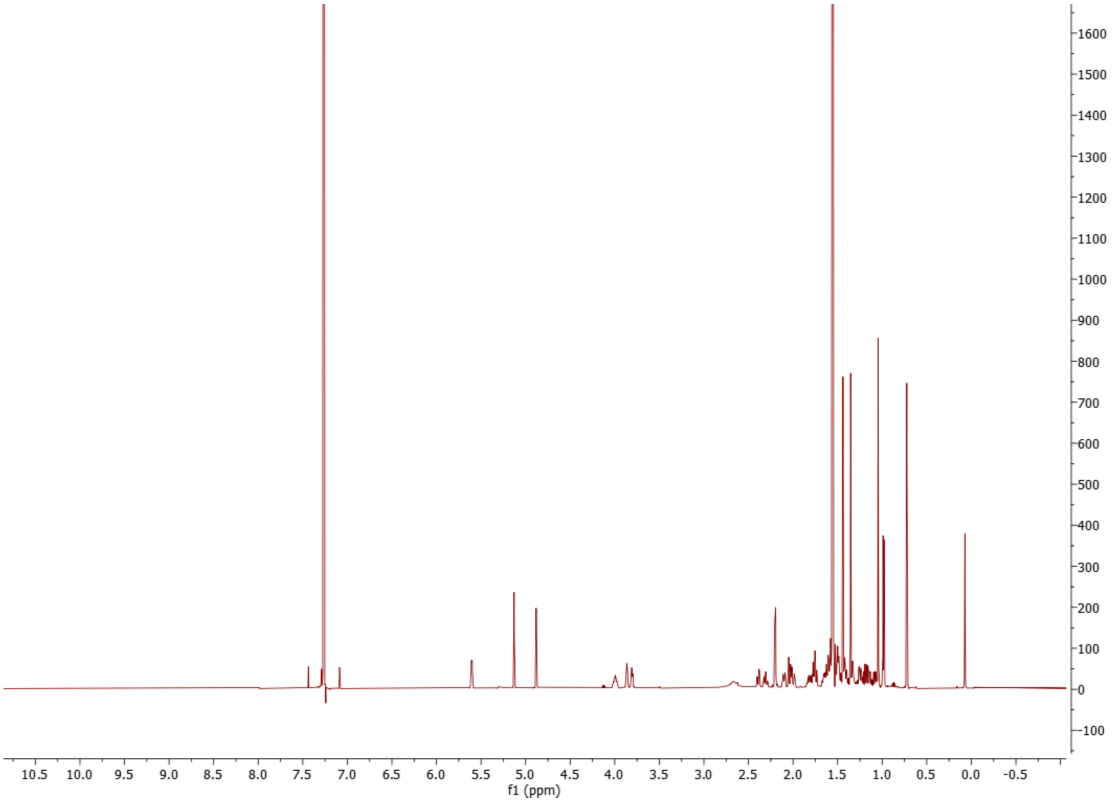

Supplementary Fig. 61. **^{1}H spectrum of (22*R*)-ergosta-5,24(28)-diene-1α,3β,22,25-tetraol (15) ($CDCl_3$, 298 K, 600 MHz).**

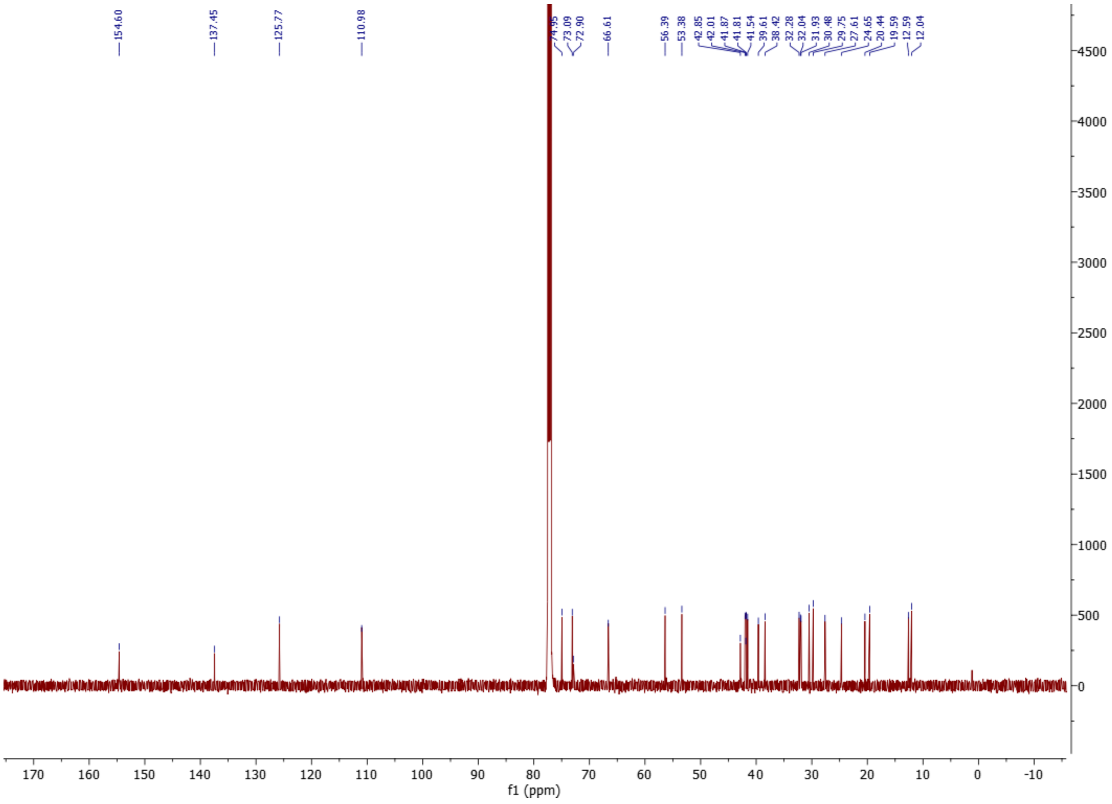

Supplementary Fig. 62. **^{13}C spectrum of (22*R*)-ergosta-5,24(28)-diene-1α,3β,22,25-tetraol (15) ($CDCl_3$, 298 K, 151 MHz).**

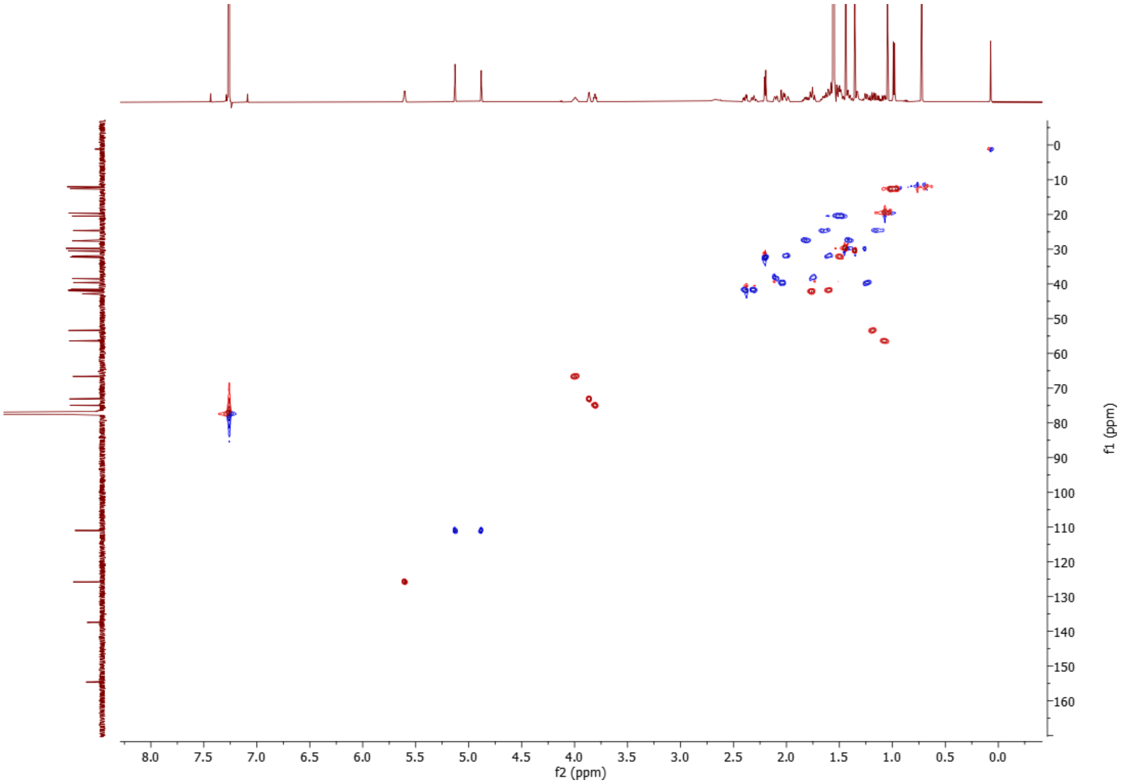

Supplementary Fig. 63. HSQC spectrum of (22*R*)-ergosta-5,24(28)-diene-1α,3β,22,25-tetraol (15) ($CDCl_3$, 298 K, 600 MHz).

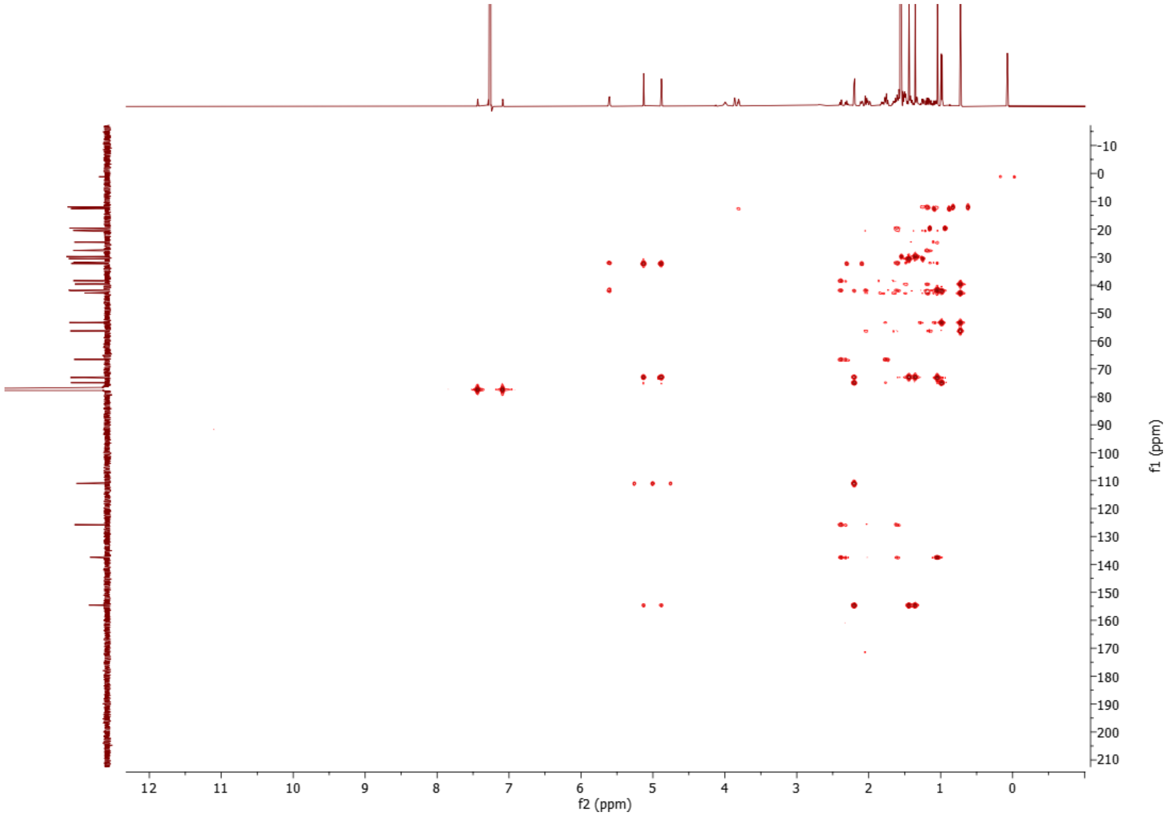

Supplementary Fig. 64. HMBC spectrum of (22*R*)-ergosta-5,24(28)-diene-1α,3β,22,25-tetraol (15) ($CDCl_3$, 298 K, 600 MHz).

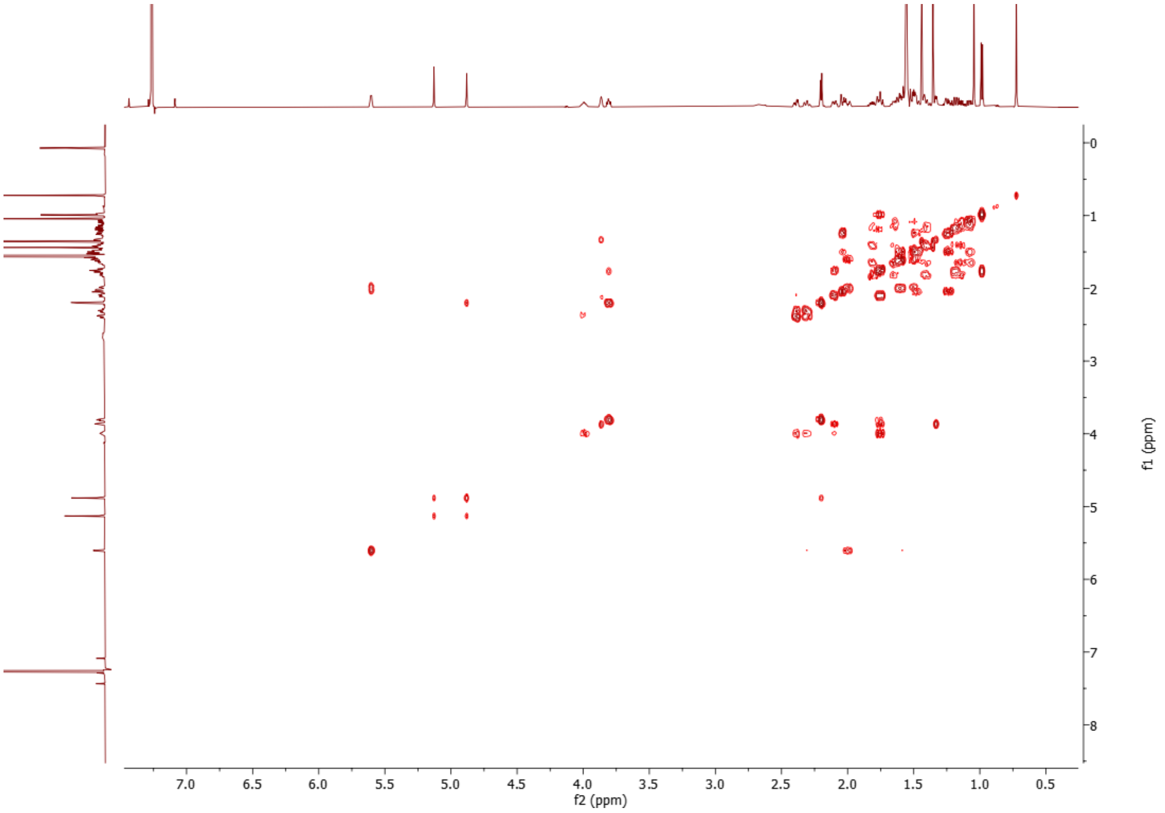

Supplementary Fig. 65. COSY spectrum of (22*R*)-ergosta-5,24(28)-diene-1α,3β,22,25-tetraol (15) ($CDCl_3$, 298 K, 600 MHz).

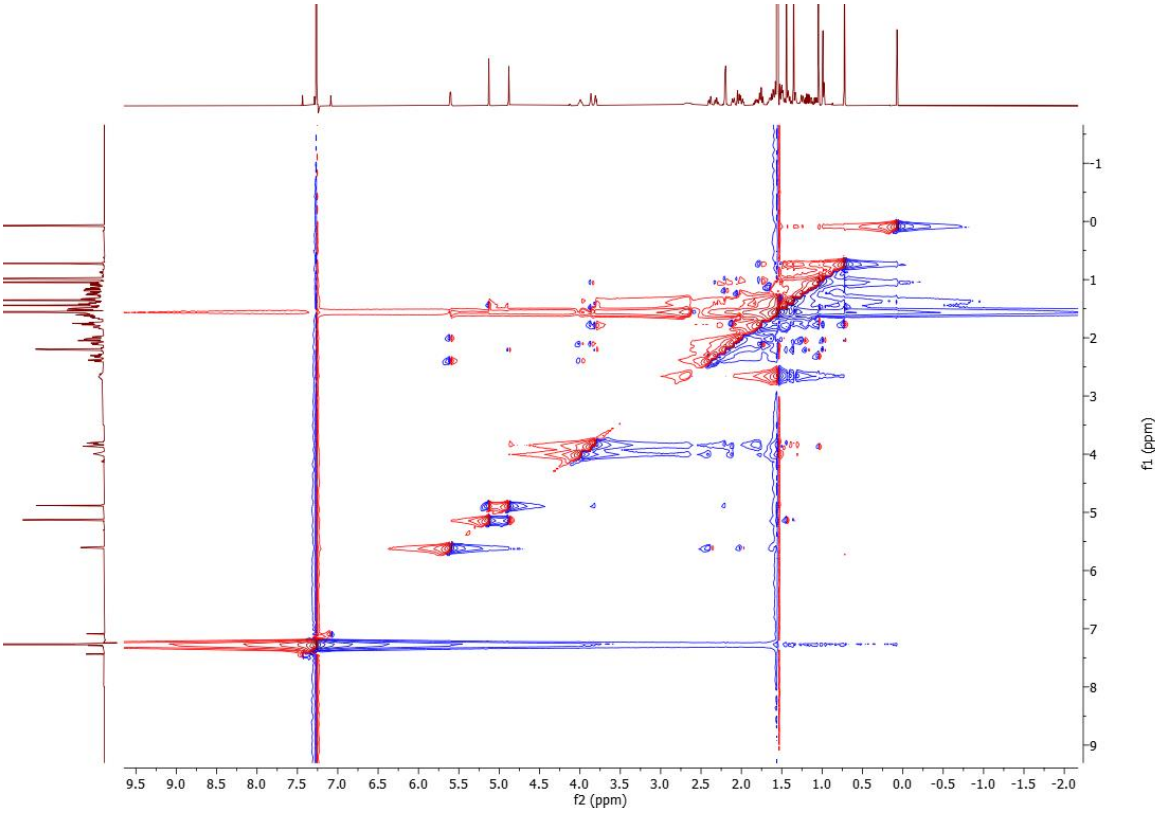

Supplementary Fig. 66. NOESY spectrum of (22*R*)-ergosta-5,24(28)-diene-1α,3β,22,25-tetraol (15) ($CDCl_3$, 298 K, 600 MHz).

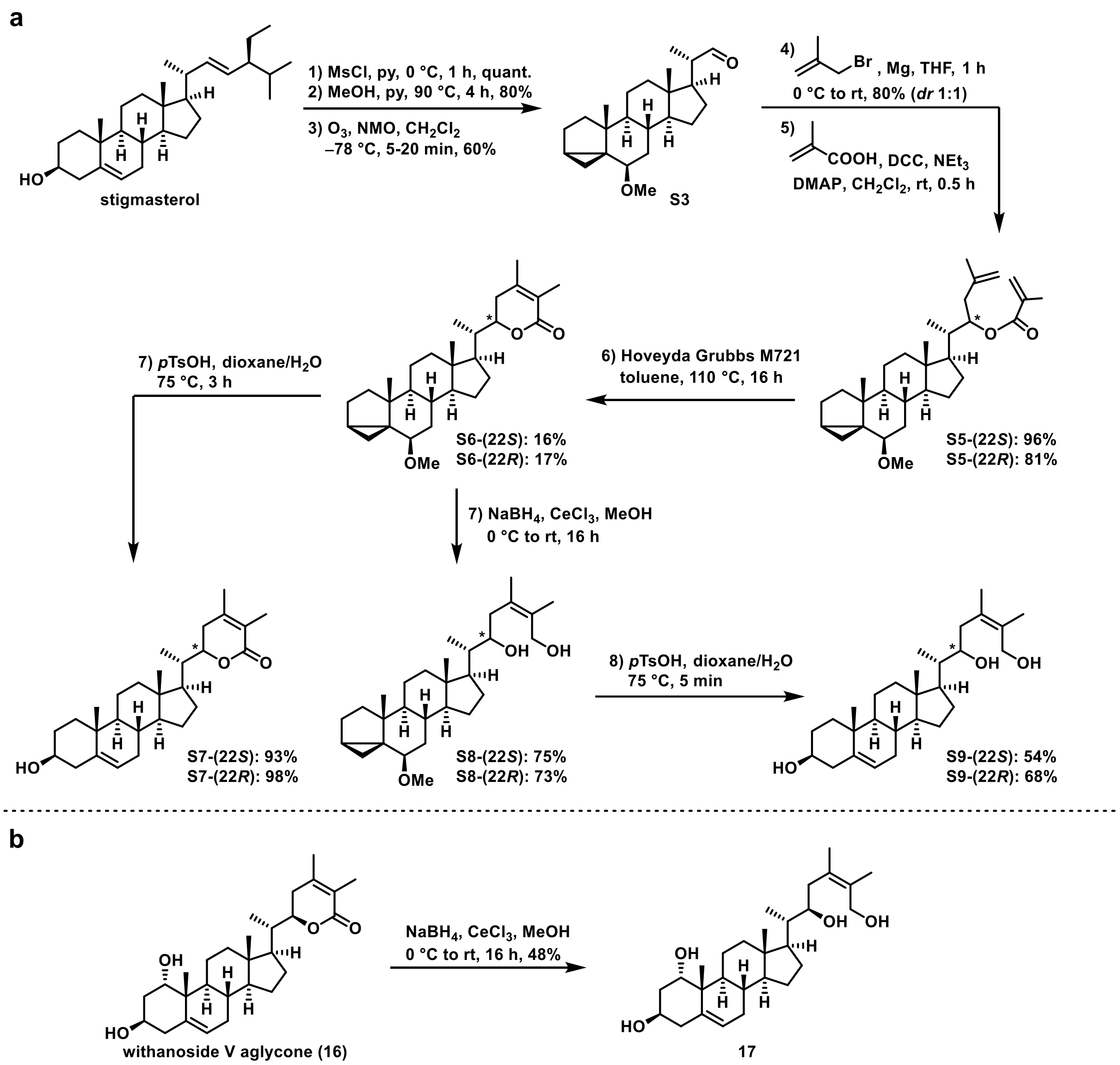

Supplementary Fig. 67. Synthetic routes used in this work.

a Synthesis of C22 diastereomer pairs **S7-(22*S*/*R*)** and **S9-(22*S*/*R*)** used to determine the C22 stereochemistry of (22*R*)-ergosta-5,24-diene-3β,22-diol (**10**) (Supplementary Fig. 20).

b Semisynthesis of (22*R*)-ergosta-5,24-diene-1α,3β,22,26-tetrol (**17**) by chemical reduction of withanoside V aglycone (**16**).

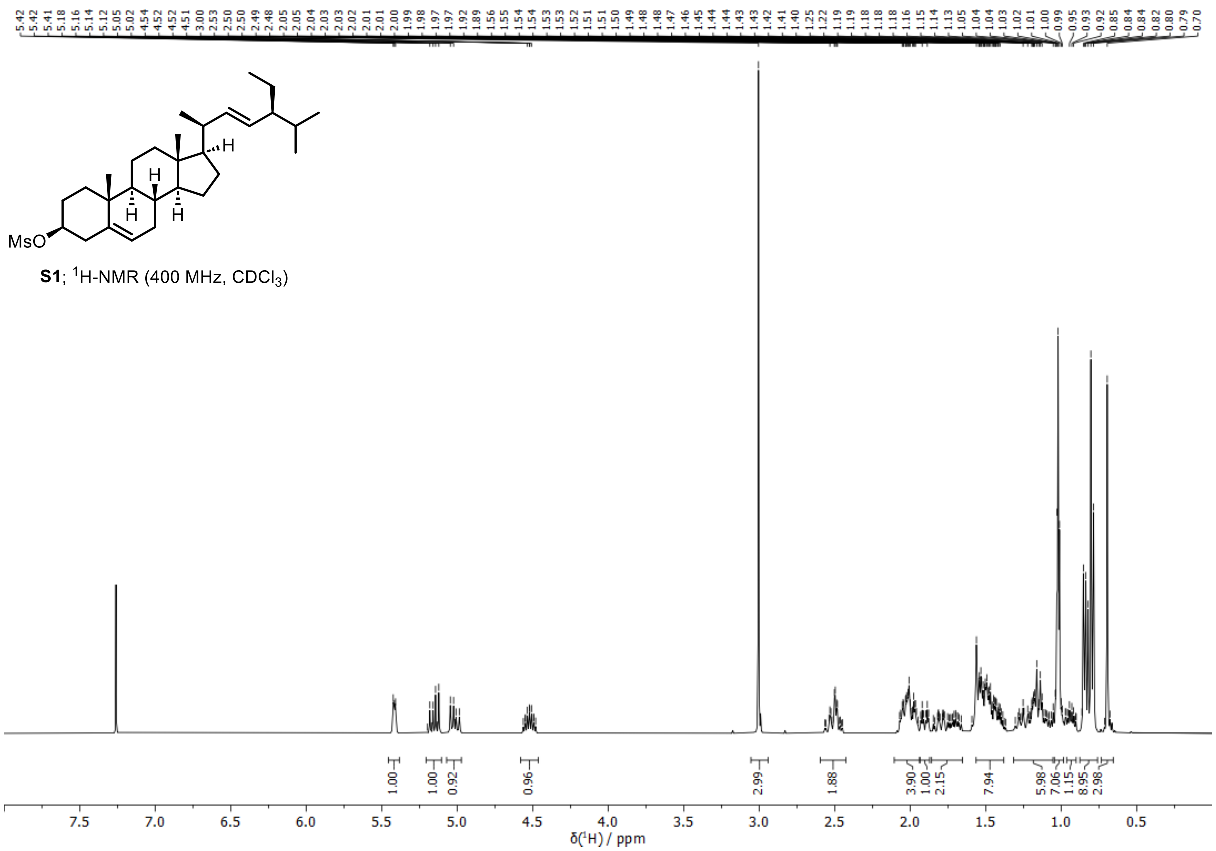

Supplementary Fig. 68. ^{1}H spectrum of stigmasteryl mesylate (S1) (400 MHz, $CDCl_3$, 298 K).

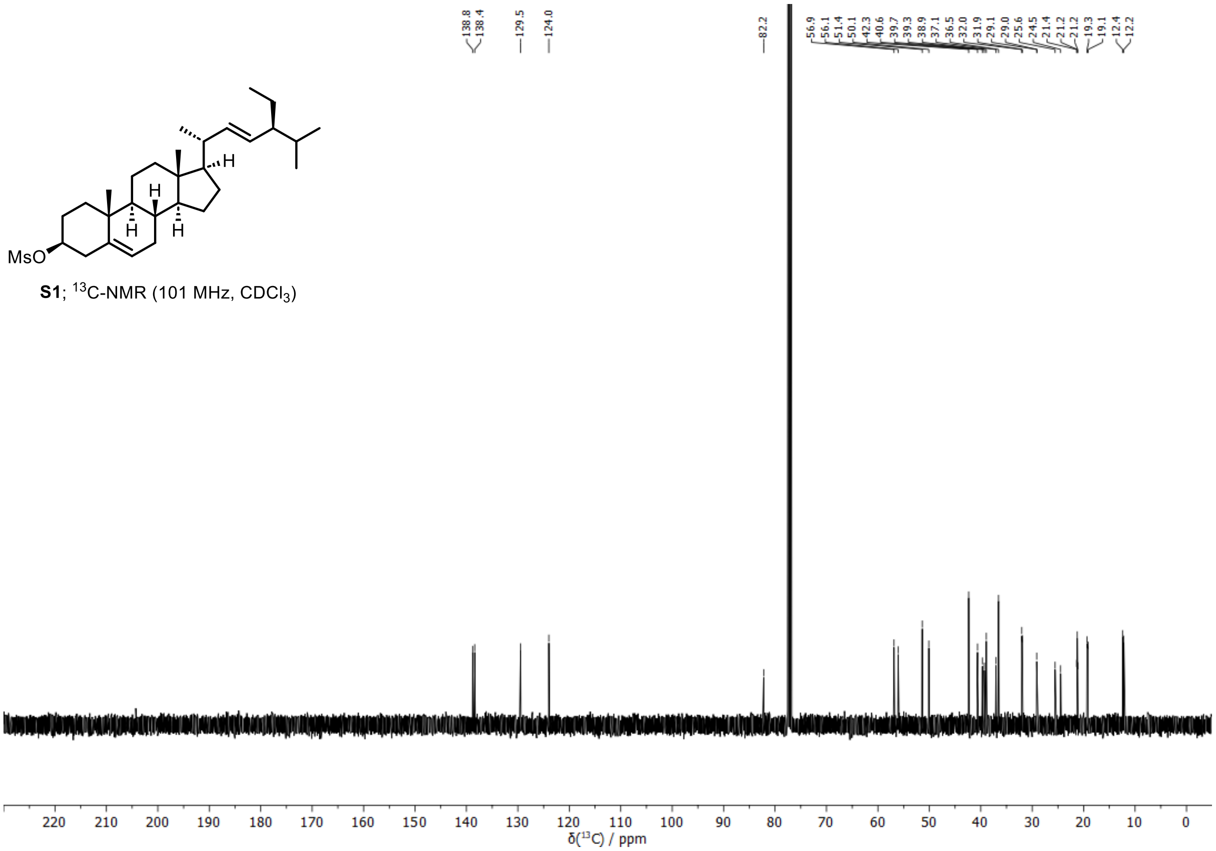

Supplementary Fig. 69. ^{13}C spectrum of stigmasteryl mesylate (S1) (101 MHz, $CDCl_3$, 298 K).

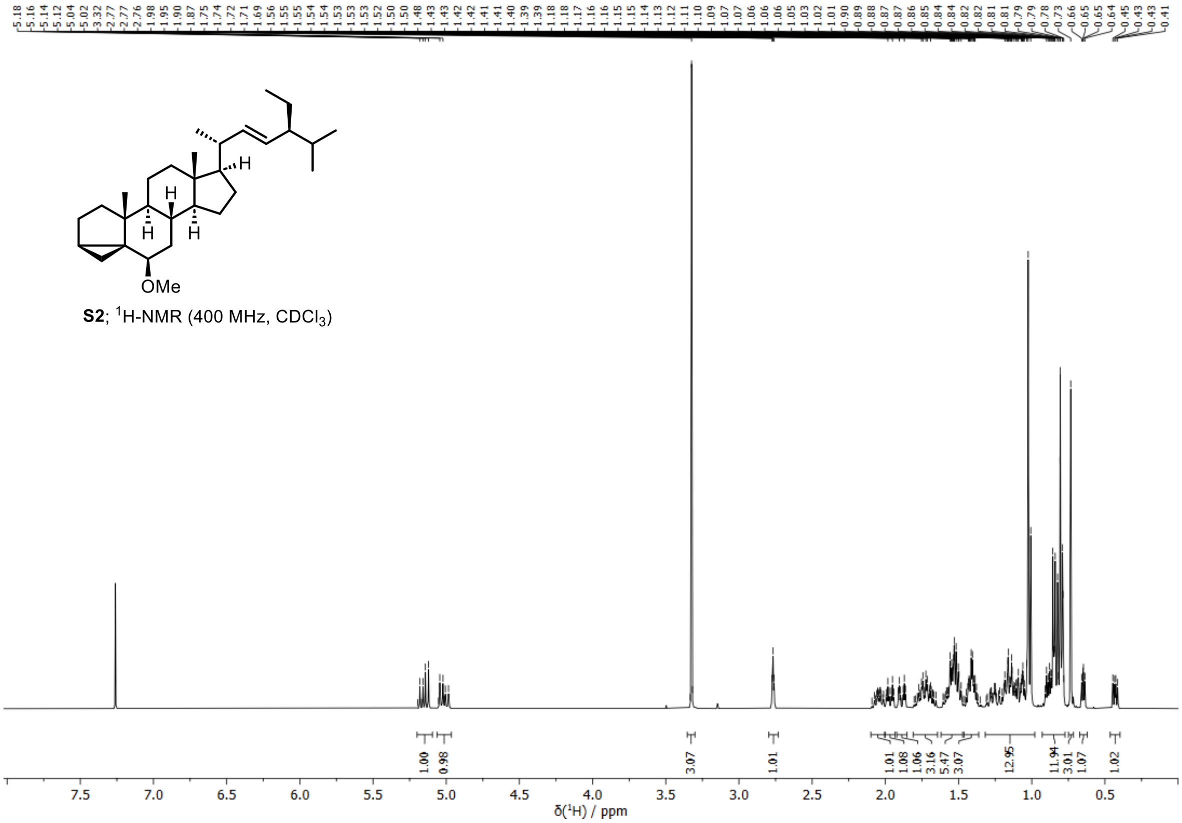

Supplementary Fig. 70. **^{1}H spectrum of *i*-stigmasteryl methyl ether (S2) (400 MHz, $CDCl_3$, 298 K).**

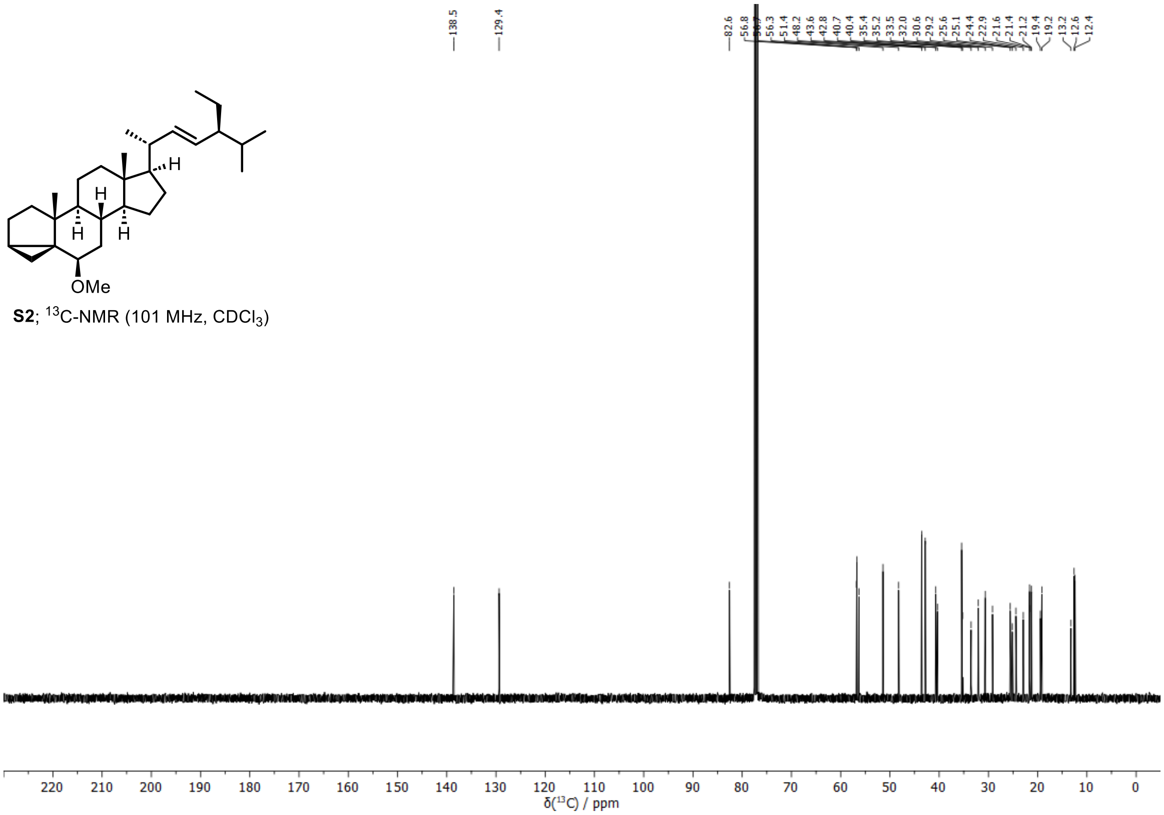

Supplementary Fig. 71. **^{13}C spectrum of *i*-stigmasteryl methyl ether (S2) (101 MHz, $CDCl_3$, 298 K).**

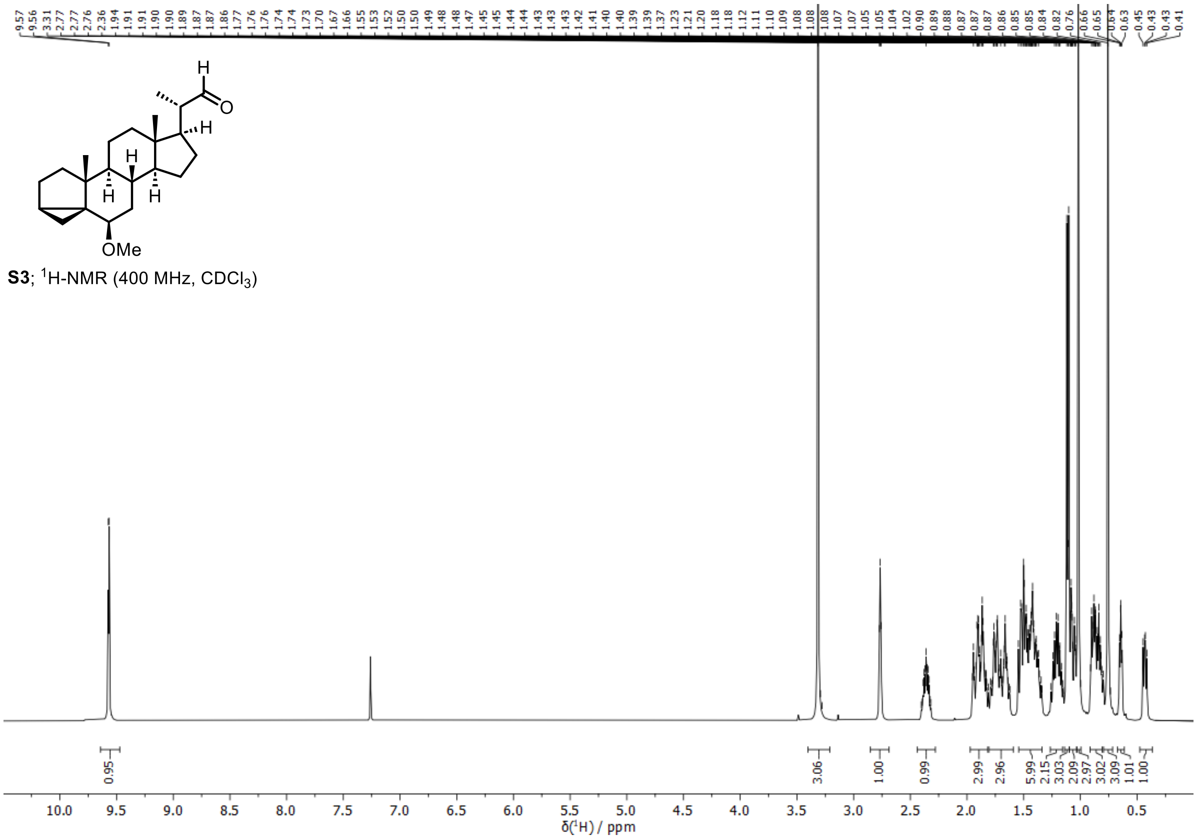

Supplementary Fig. 72. ^{1}H spectrum of (20*S*)-6*β*-methoxy-3*α*,5-cyclopregnancarbaldehyde (S3) (400 MHz, $CDCl_3$, 298 K).

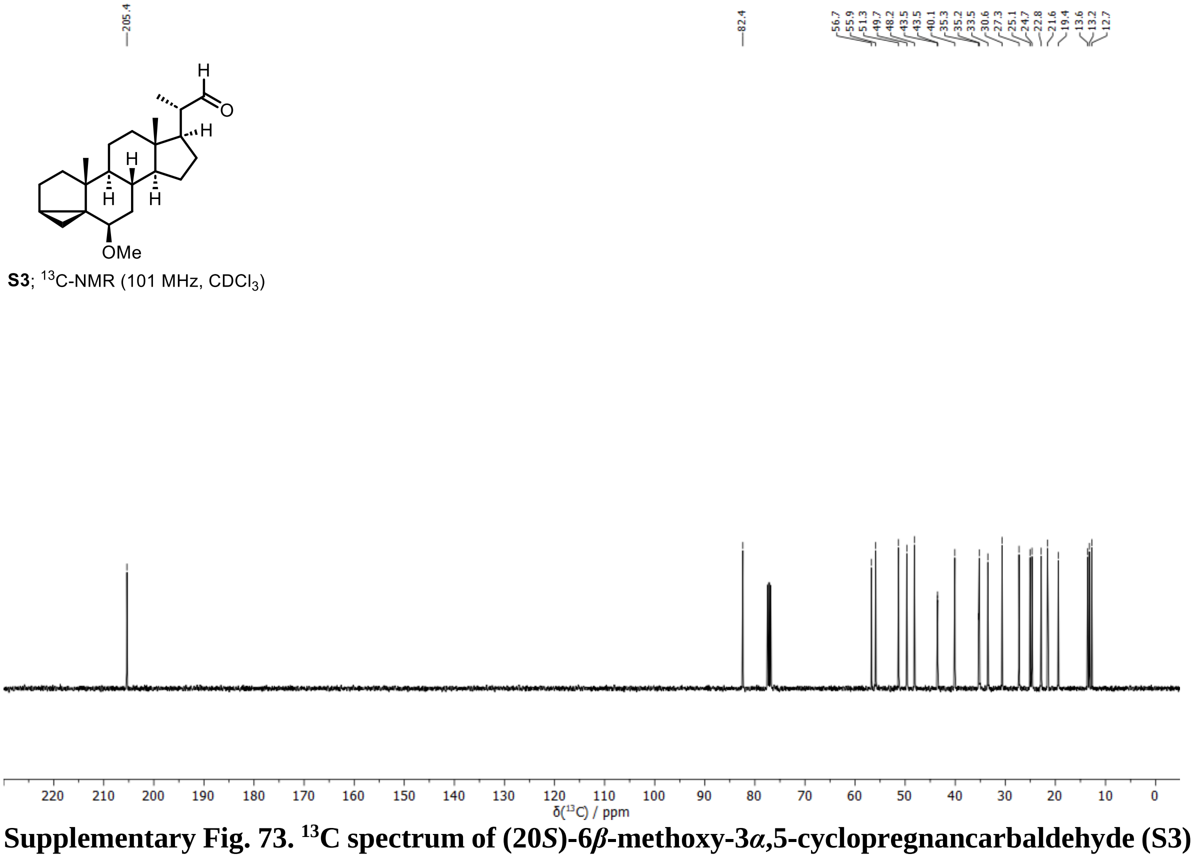

Supplementary Fig. 73. ^{13}C spectrum of (20*S*)-6*β*-methoxy-3*α*,5-cyclopregnancarbaldehyde (S3) (101 MHz, $CDCl_3$, 298 K).

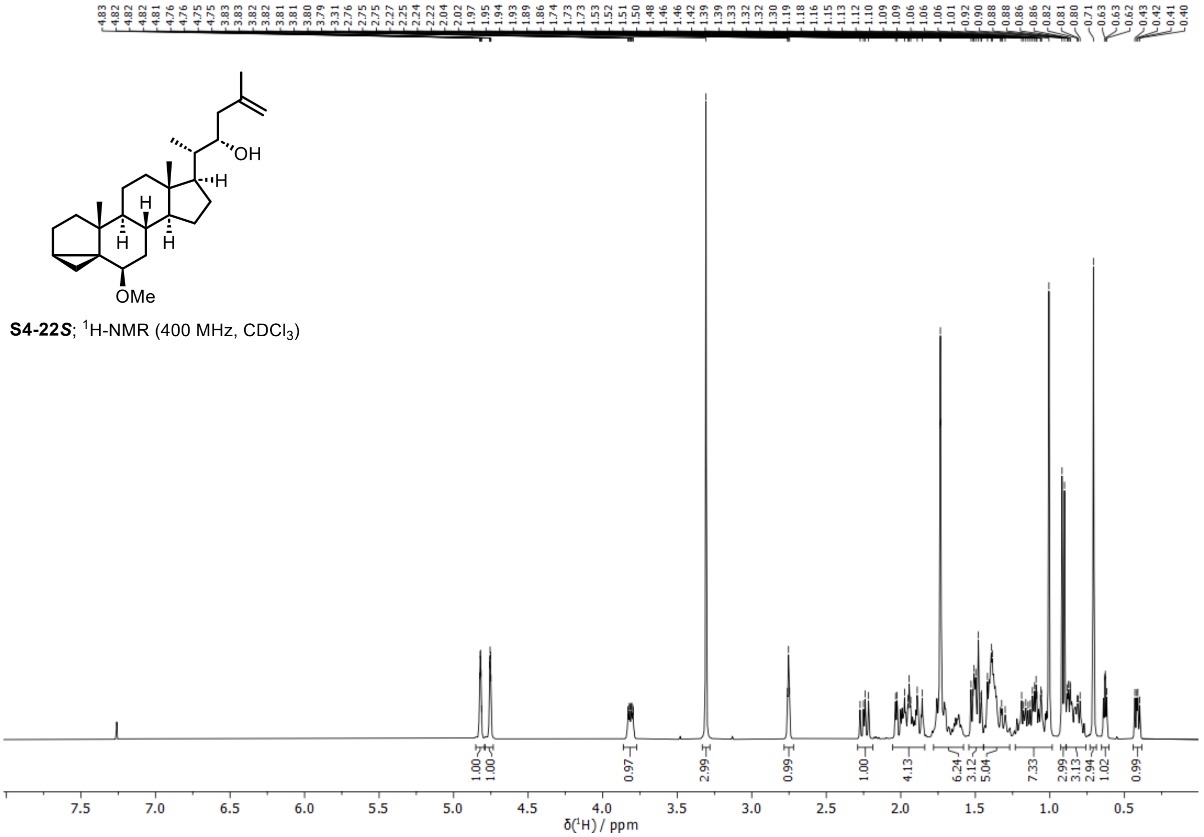

Supplementary Fig. 74. ^{1}H spectrum of (22*S*)-24-nor-6*β*-methoxy-3*α*,5-cyclocholest-25-en-22-ol (S4-(22*S*)) (400 MHz, $CDCl_3$, 298 K).

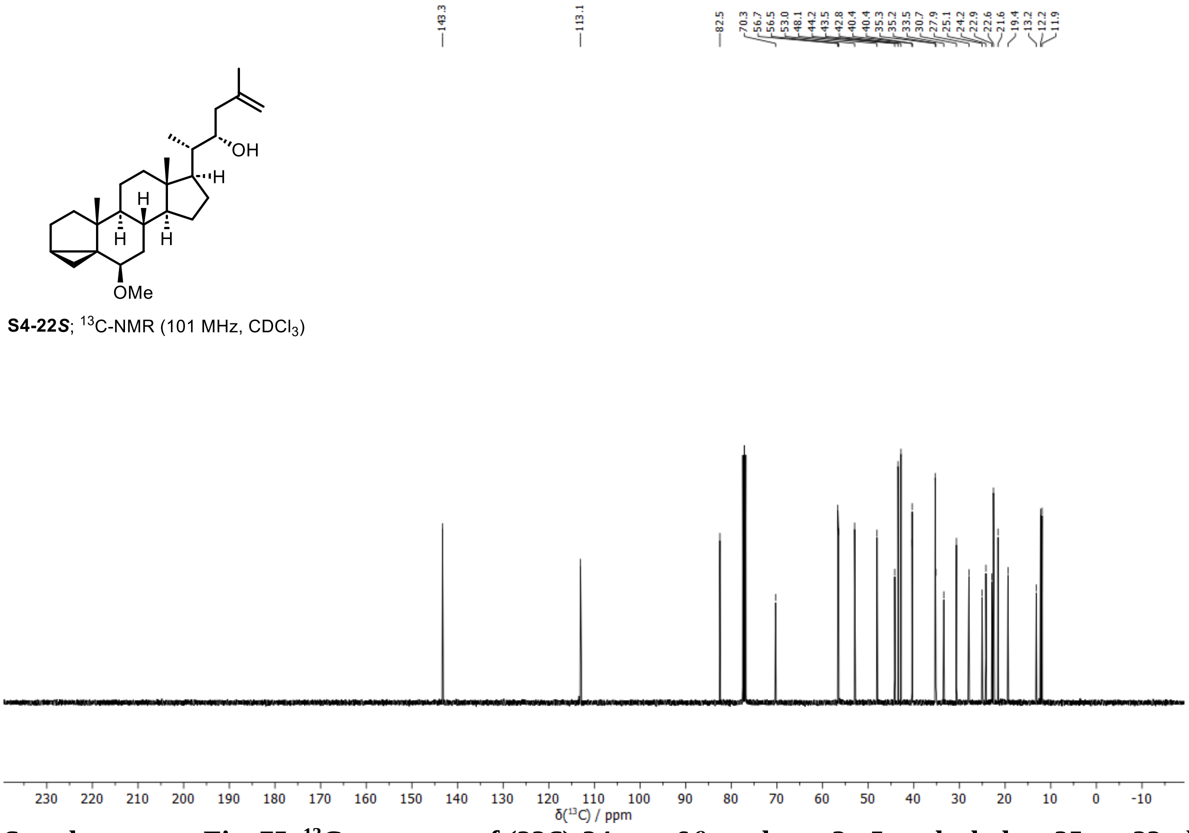

Supplementary Fig. 75. ^{13}C spectrum of (22*S*)-24-nor-6*β*-methoxy-3*α*,5-cyclocholest-25-en-22-ol (S4-(22*S*)) (101 MHz, $CDCl_3$, 298 K).

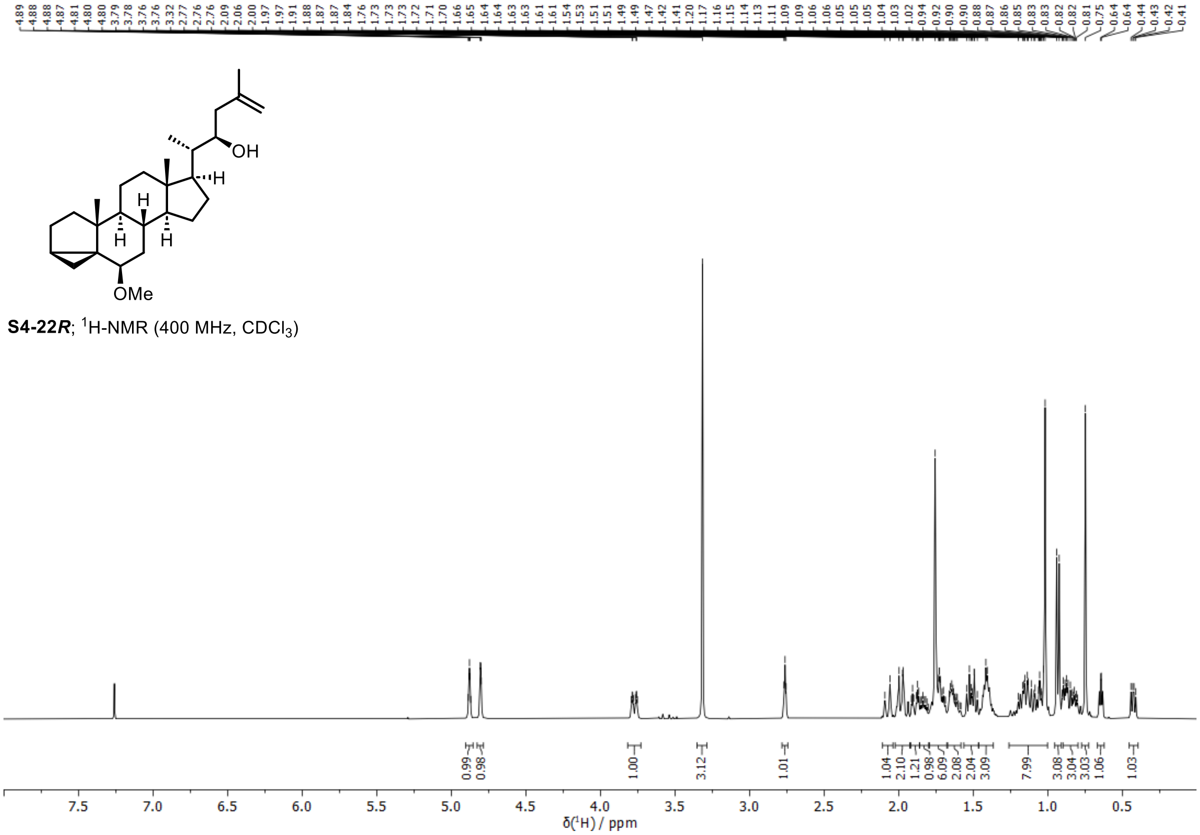

Supplementary Fig. 76. ^{1}H spectrum of (22*R*)-24-nor-6*β*-methoxy-3*α*,5-cyclocholest-25-en-22-ol (S4-(22*R*)) (400 MHz, $CDCl_3$, 298 K).

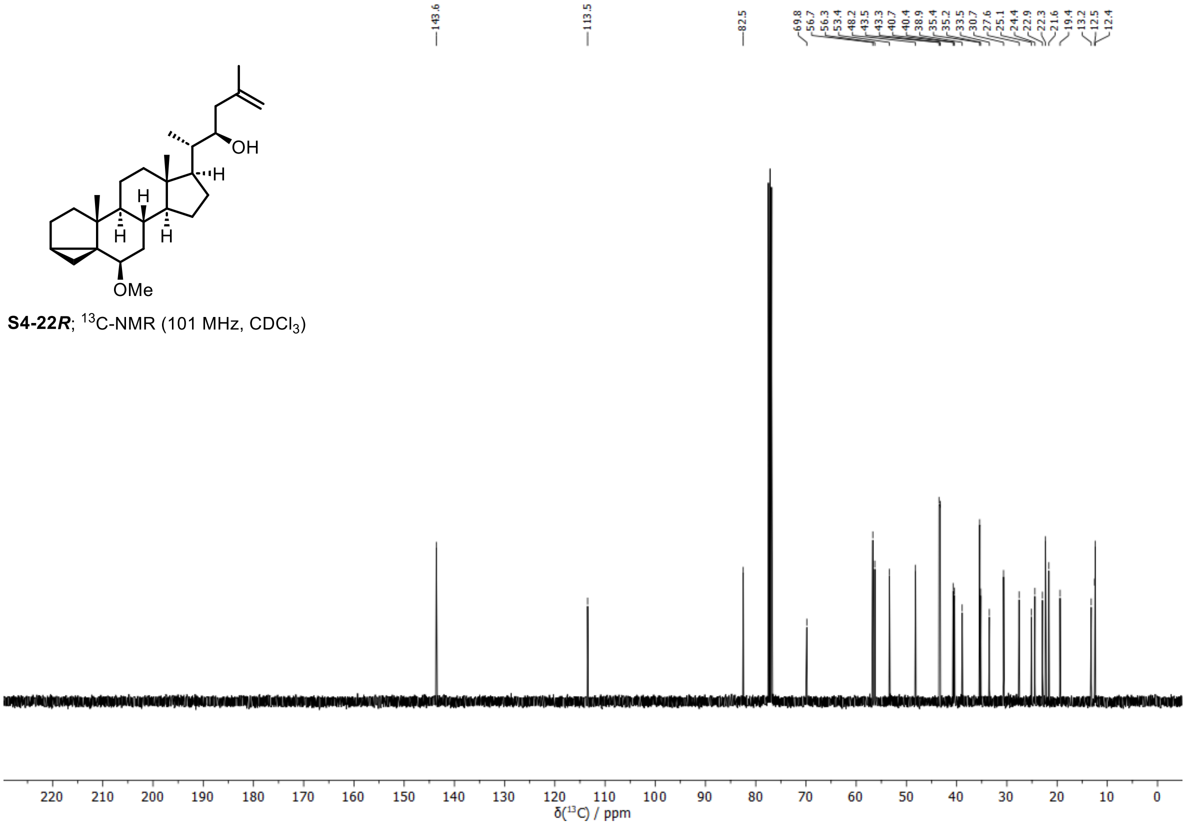

Supplementary Fig. 77. ^{13}C spectrum of (22*R*)-24-nor-6*β*-methoxy-3*α*,5-cyclocholest-25-en-22-ol (S4-(22*R*)) (101 MHz, $CDCl_3$, 298 K).

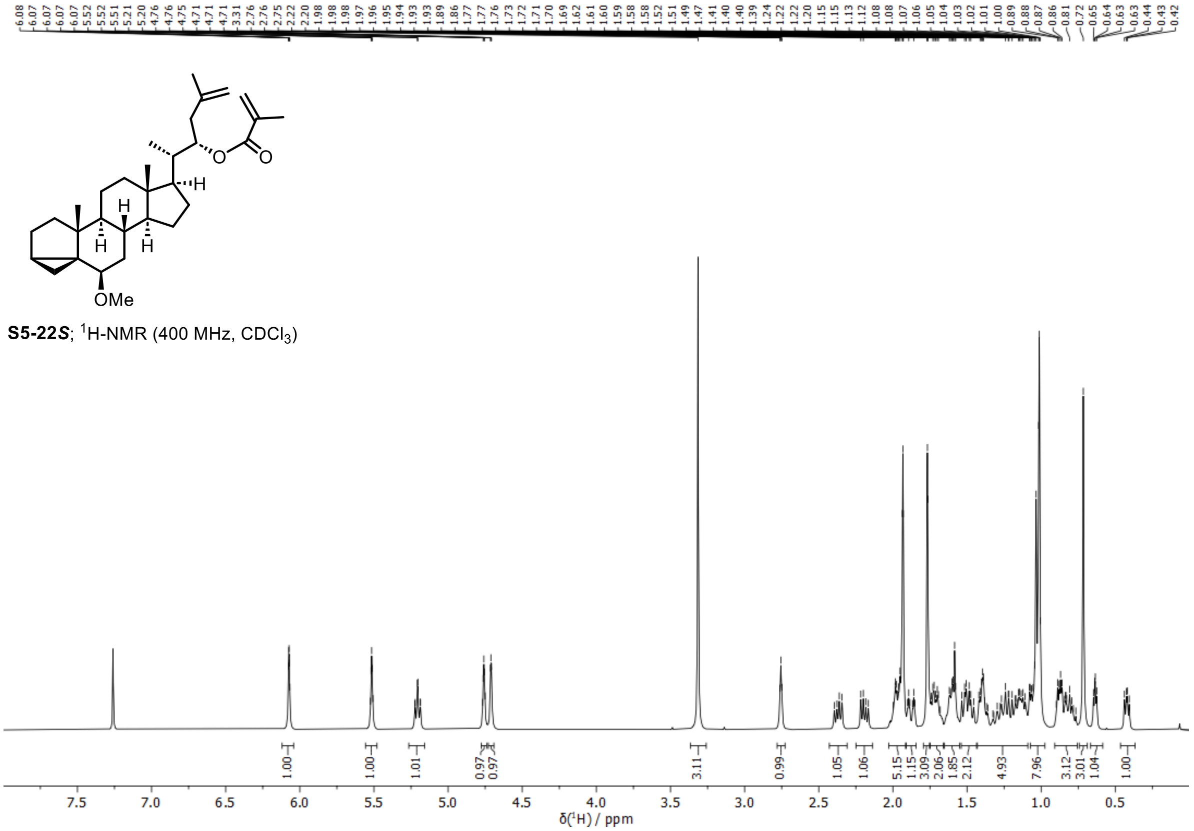

Supplementary Fig. 78. ^{1}**H spectrum of (22*S*)-24-nor-6*β*-methoxy-3*α*,5-cyclocholest-25-ene-22-methacrylate (S5-(22*S*)) (400 MHz, CDCl$_3$, 298 K).**

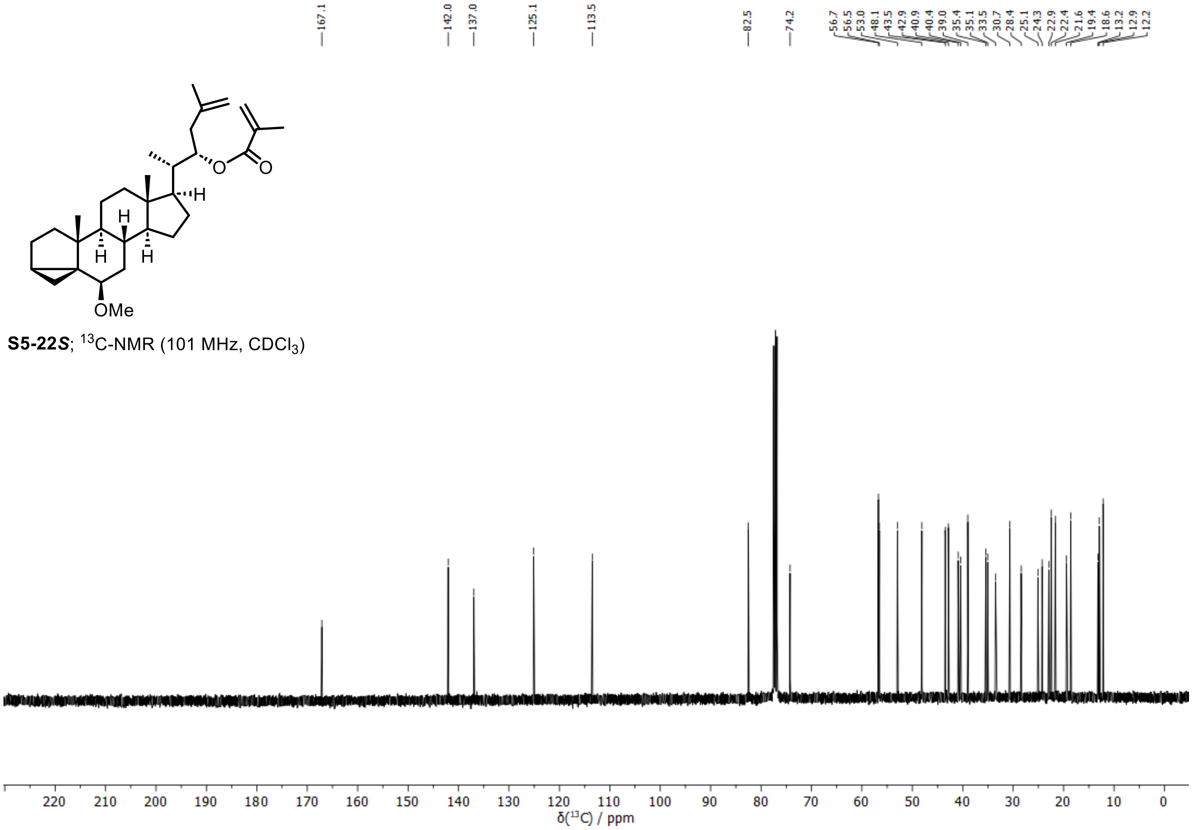

Supplementary Fig. 79. ^{13}**C spectrum of (22*S*)-24-nor-6*β*-methoxy-3*α*,5-cyclocholest-25-ene-22-methacrylate (S5-(22*S*)) (101 MHz, CDCl$_3$, 298 K).**

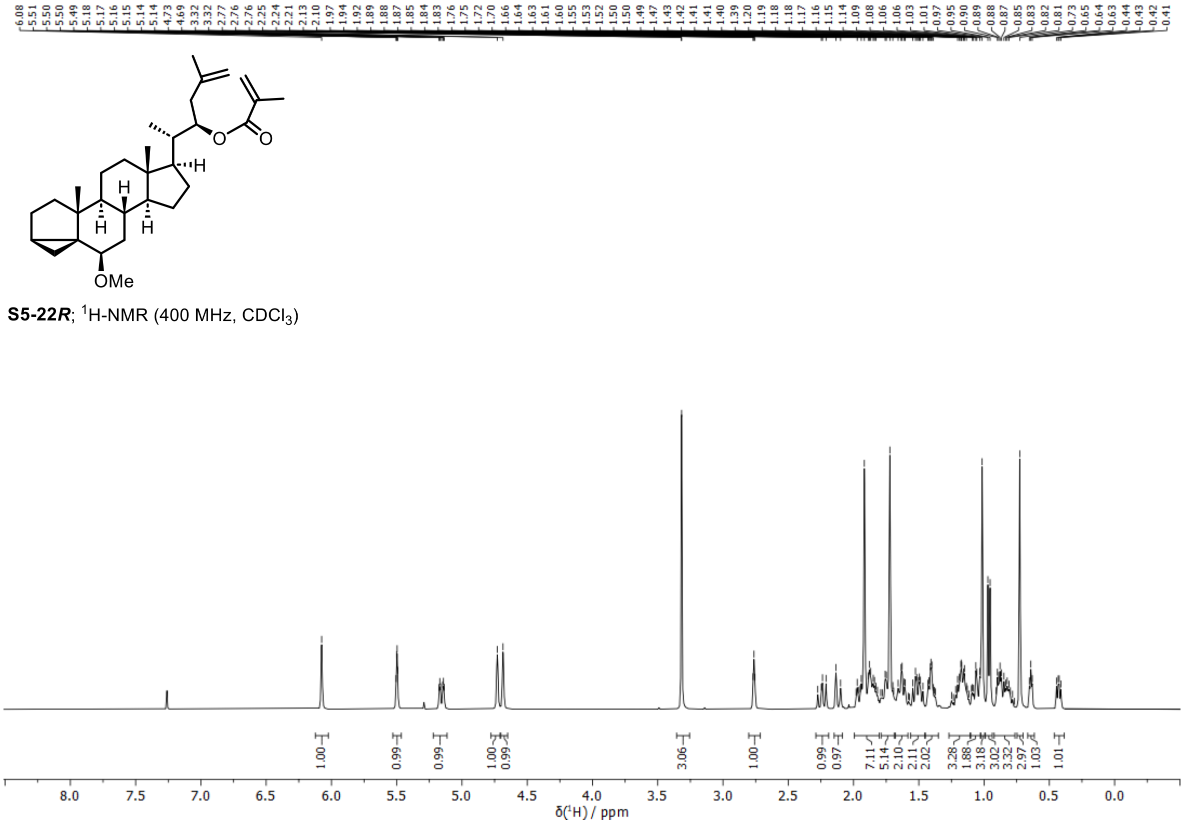

Supplementary Fig. 80. ^{1}H spectrum of (22*R*)-24-nor-6*β*-methoxy-3*α*,5-cyclocholest-25-ene-22-methacrylate (S5-(22*R*)) (400 MHz, $CDCl_3$, 298 K).

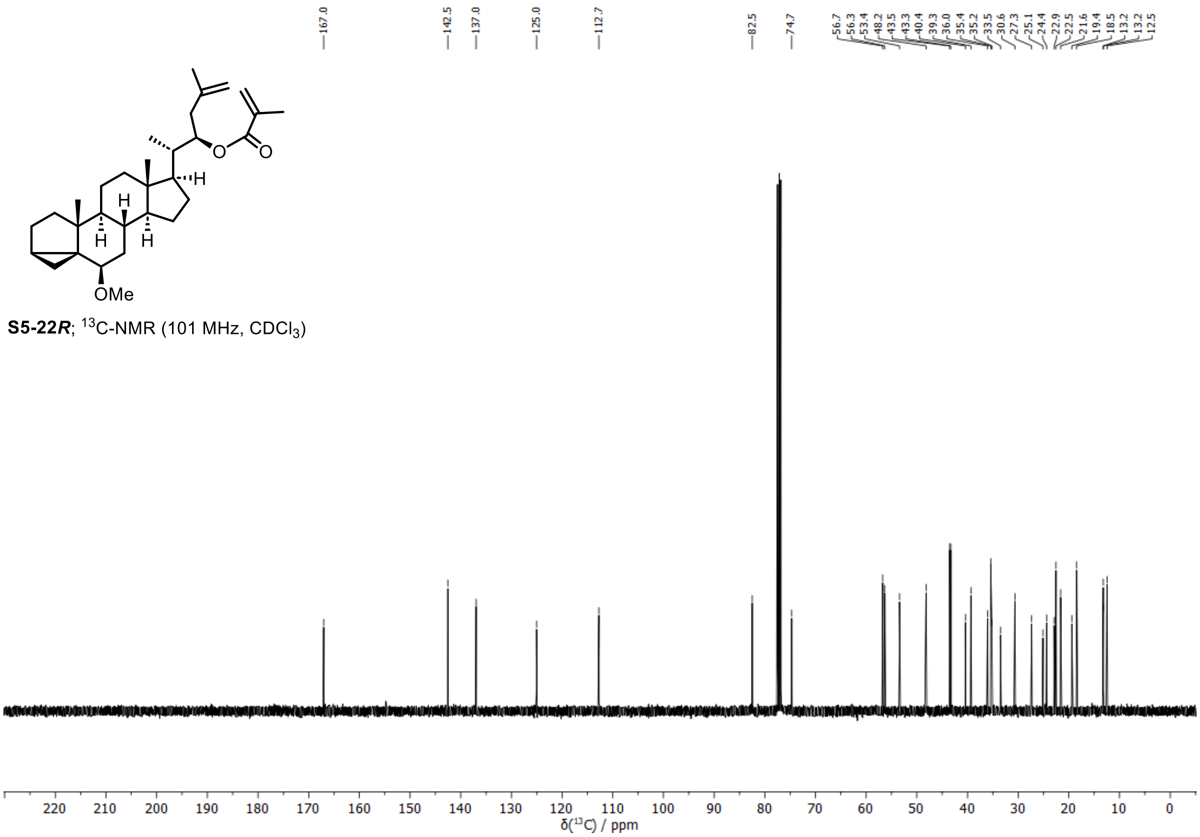

Supplementary Fig. 81. ^{13}C spectrum of (22*R*)-24-nor-6*β*-methoxy-3*α*,5-cyclocholest-25-ene-22-methacrylate (S5-(22*R*)) (101 MHz, $CDCl_3$, 298 K).

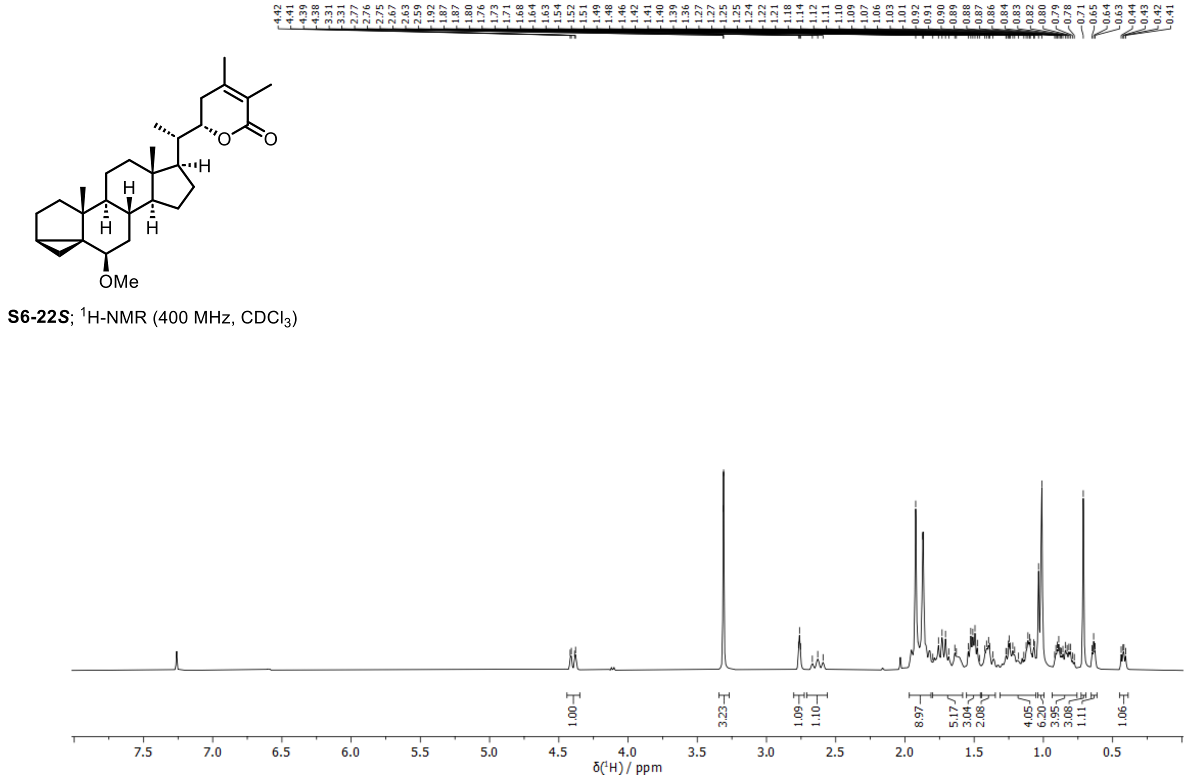

Supplementary Fig. 82. ^{1}H spectrum of (22*S*)-6*β*-methoxy-3*α*,5-cycloergosta-24,25-diene-26,22-lactone (S6-(22*S*)) (400 MHz, CDCl$_3$, 298 K).

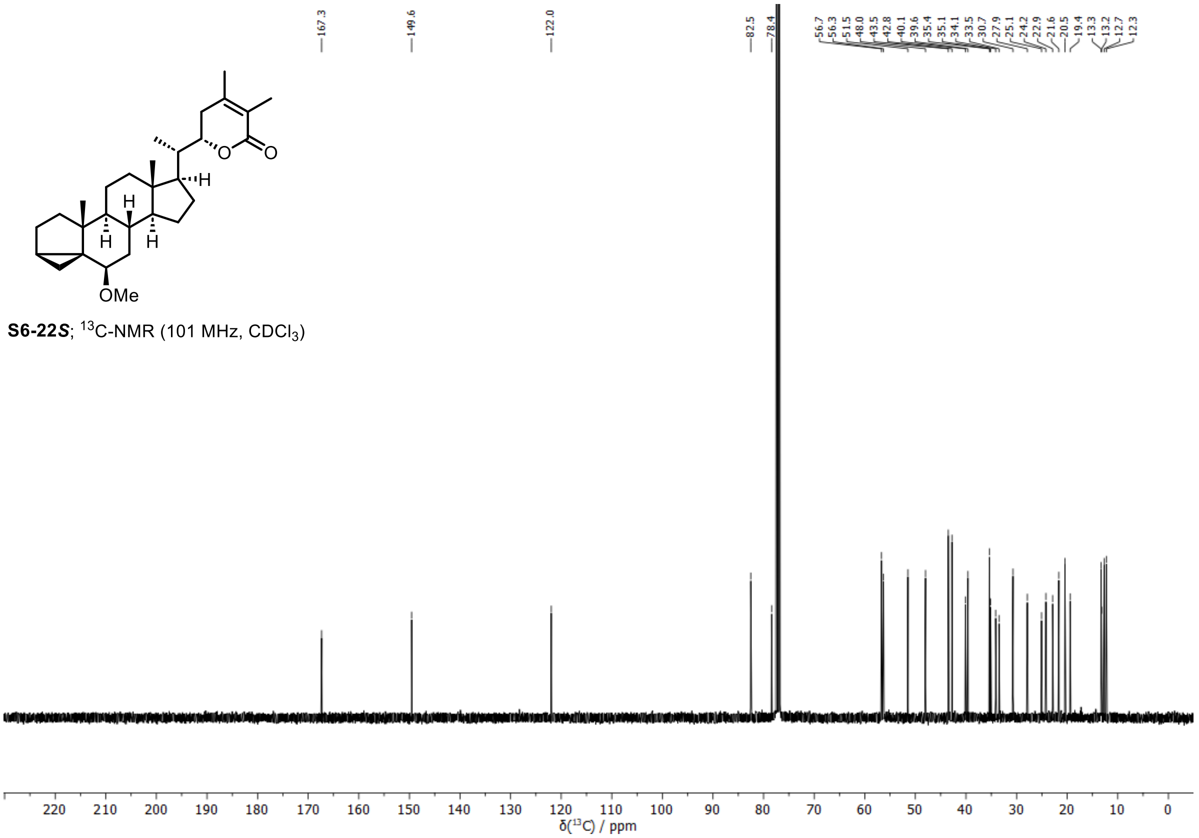

Supplementary Fig. 83. ^{13}C spectrum of (22*S*)-6*β*-methoxy-3*α*,5-cycloergosta-24,25-diene-26,22-lactone (S6-(22*S*)) (101 MHz, CDCl$_3$, 298 K).

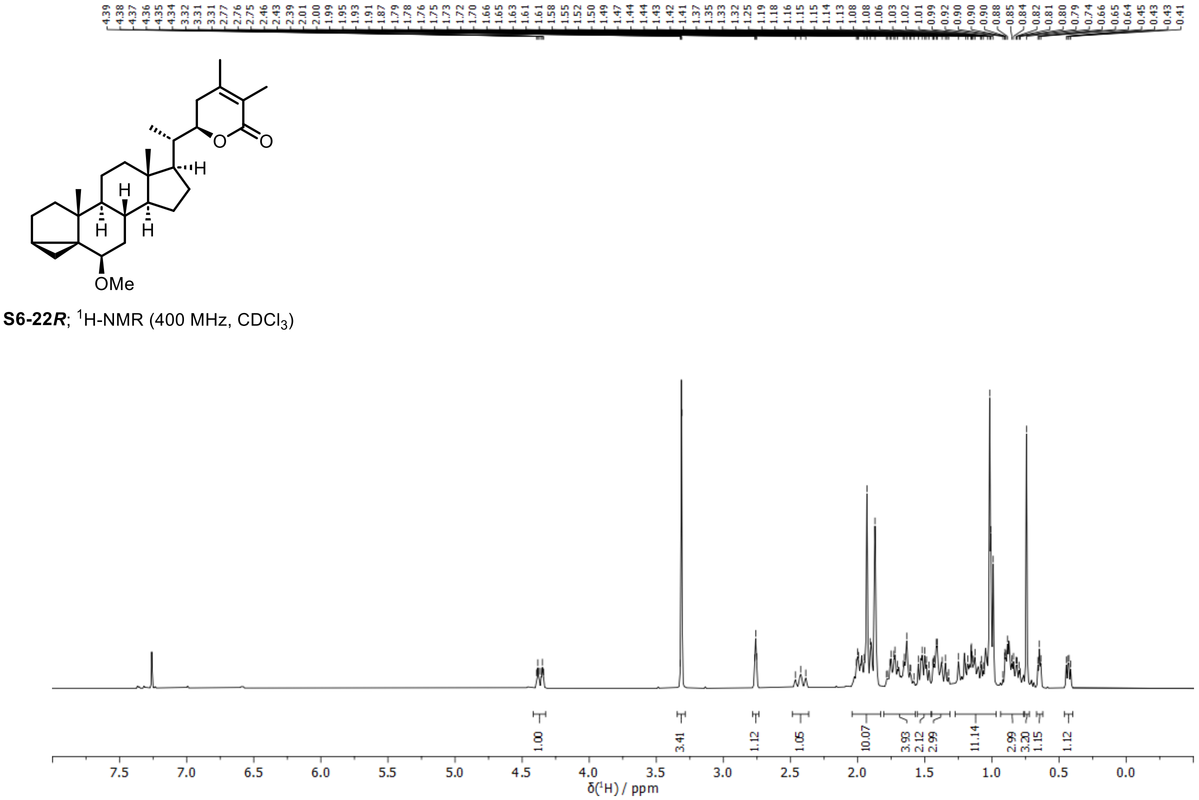

Supplementary Fig. 84. **^{1}H spectrum of (22*R*)-6*β*-methoxy-3*α*,5-cycloergosta-24,25-diene-26,22-lactone (S6-(22*R*)) (400 MHz, $CDCl_3$, 298 K).**

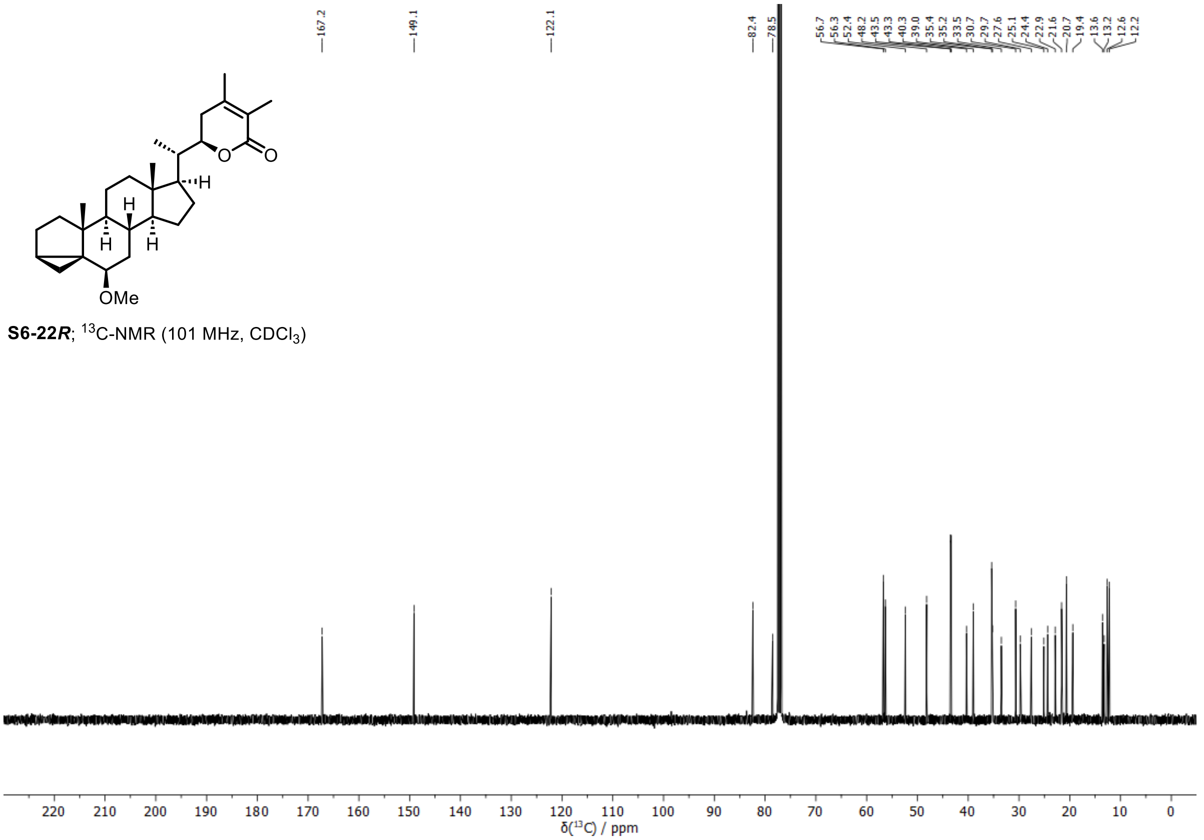

Supplementary Fig. 85. **^{13}C spectrum of (22*R*)-6*β*-methoxy-3*α*,5-cycloergosta-24,25-diene-26,22-lactone (S6-(22*R*)) (101 MHz, $CDCl_3$, 298 K).**

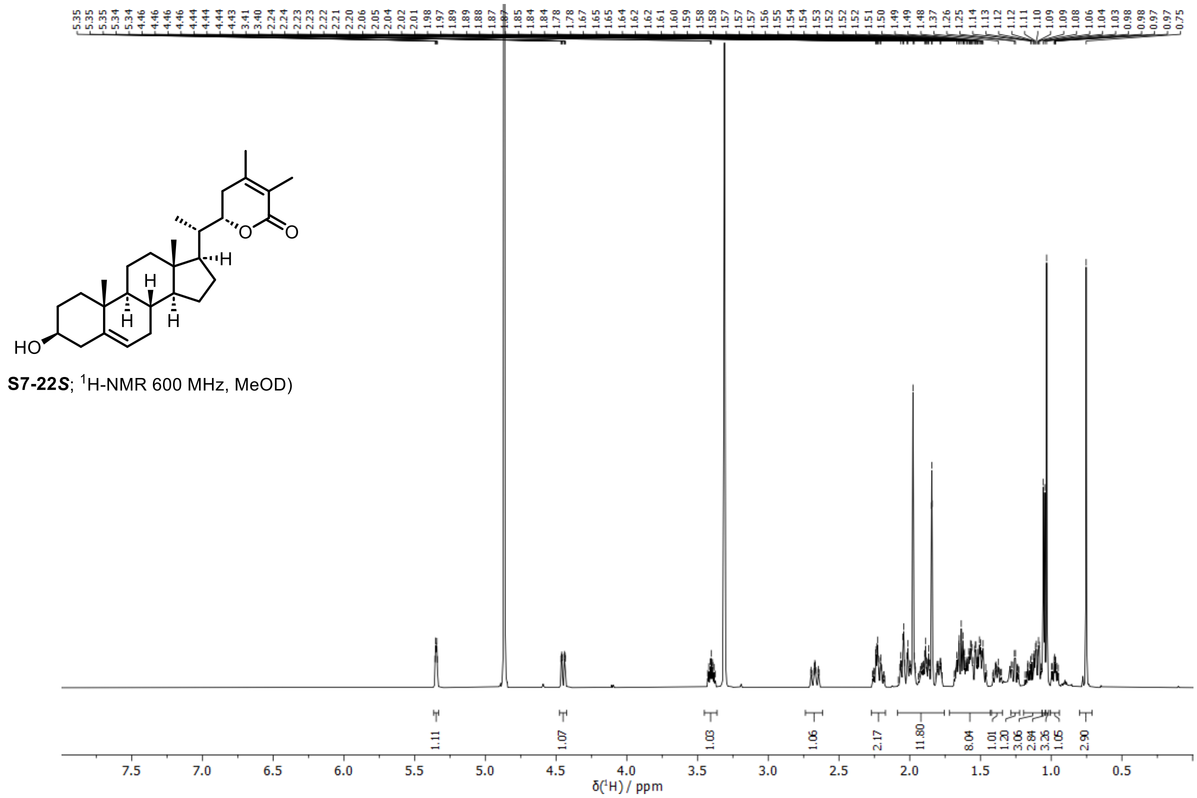

Supplementary Fig. 86. ^{1}H spectrum of (22*S*)-3*β*-hydroxyergosta-5,24-diene-26,22-lactone (S7-(22*S*)) (600 MHz, MeOD, 298 K).

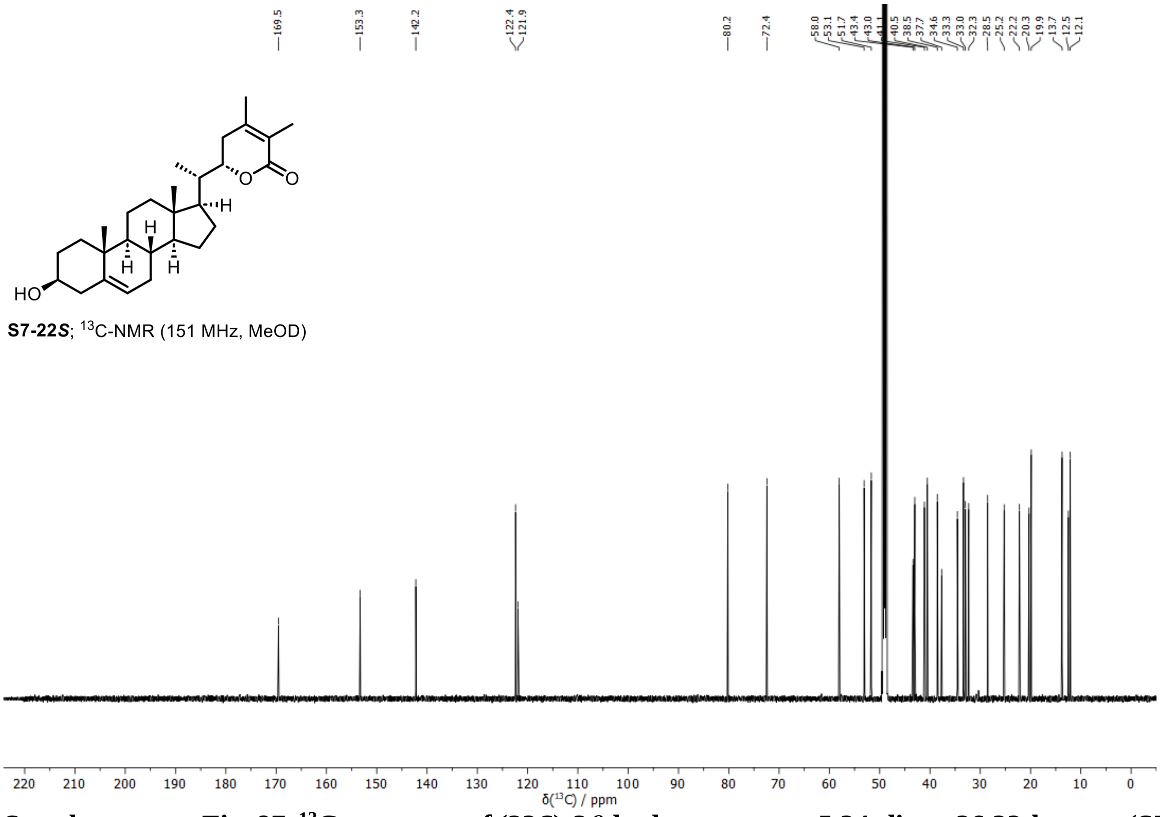

Supplementary Fig. 87. ^{13}C spectrum of (22*S*)-3*β*-hydroxyergosta-5,24-diene-26,22-lactone (S7-(22*S*)) (151 MHz, MeOD, 298 K).

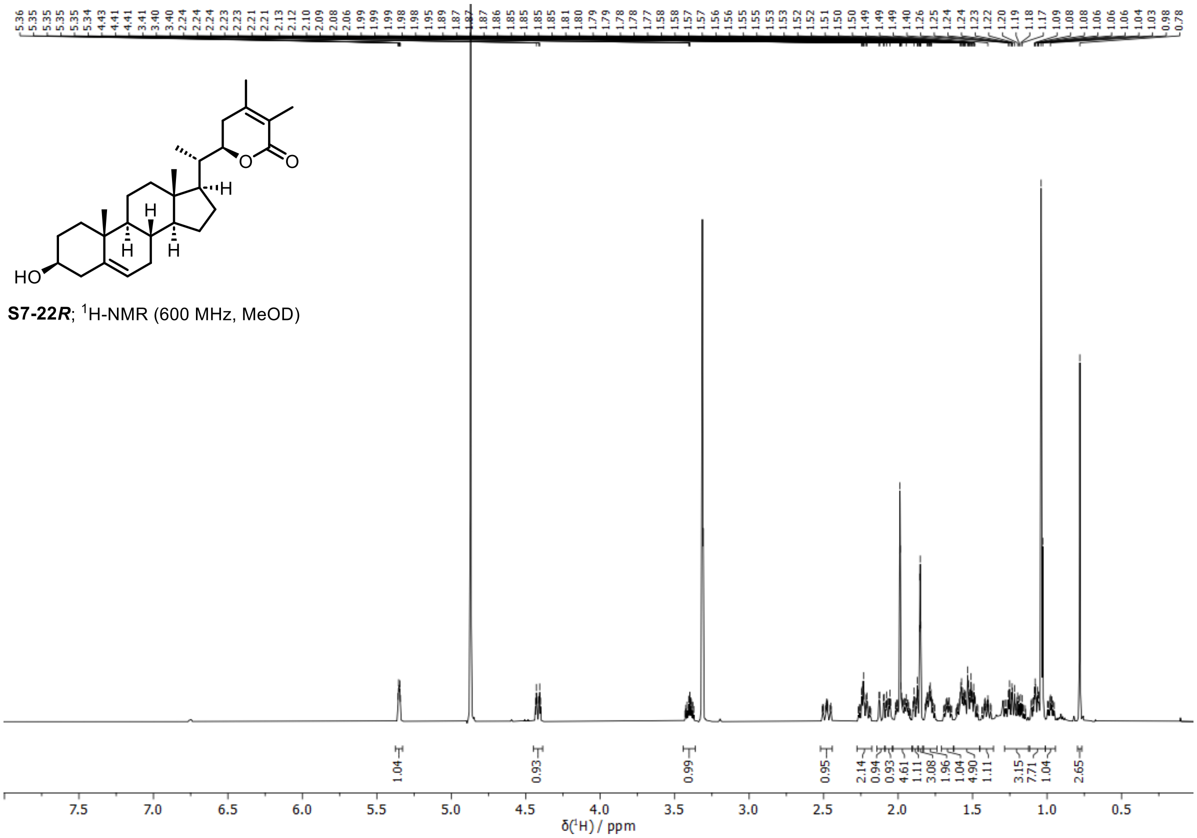

Supplementary Fig. 88. ^{1}H spectrum of (22*R*)-3*β*-hydroxyergosta-5,24-diene-26,22-lactone (S7-(22*R*)) (600 MHz, MeOD, 298 K).

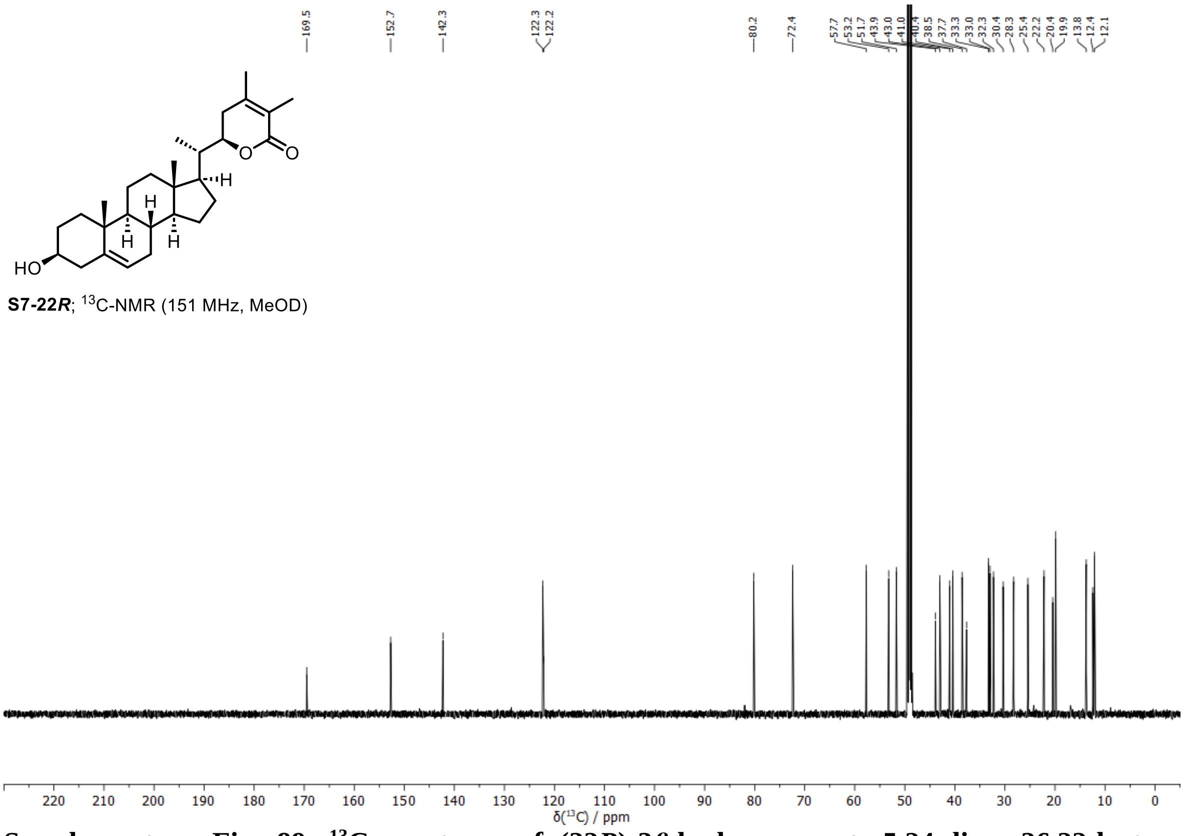

Supplementary Fig. 89. ^{13}C spectrum of (22*R*)-3*β*-hydroxyergosta-5,24-diene-26,22-lactone (S7-(22*R*)) (151 MHz, MeOD, 298 K).

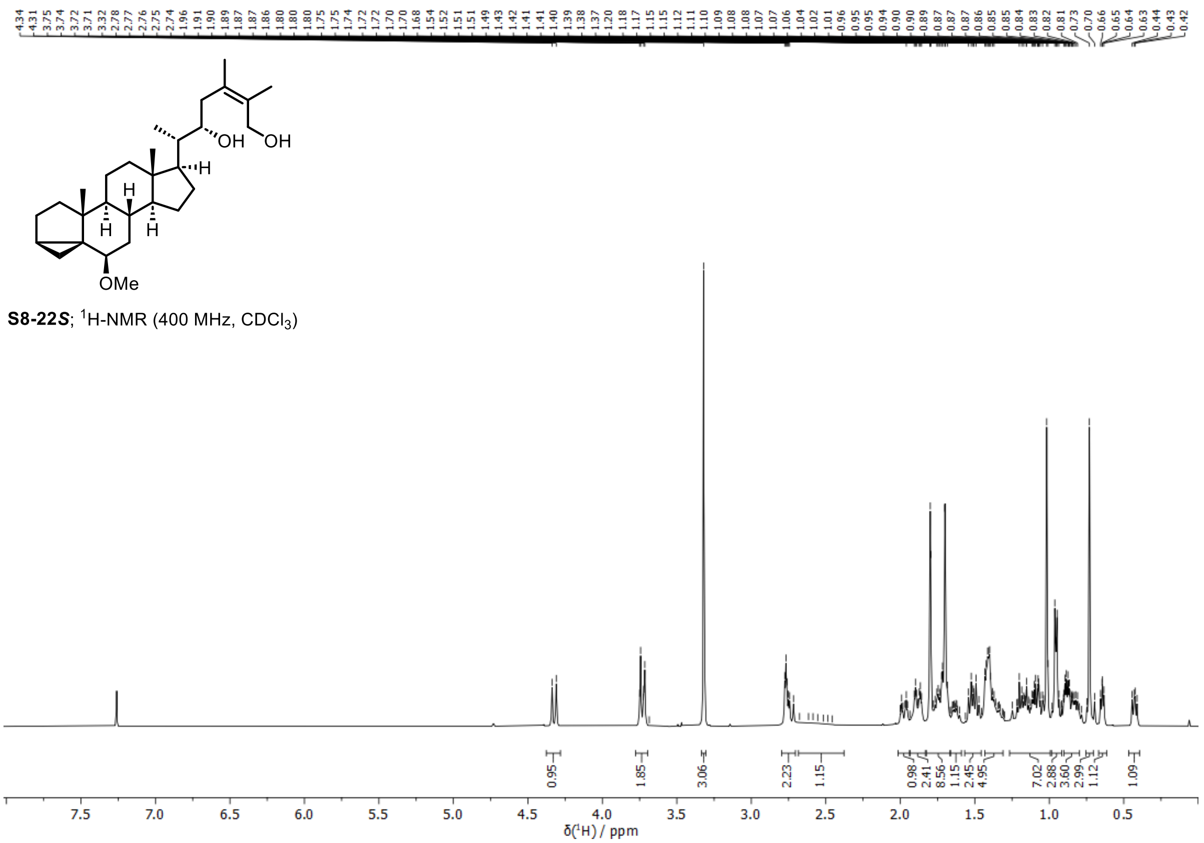

Supplementary Fig. 90. ^{1}H spectrum of (22*S*)-6*β*-methoxy-3*α*,5-cycloergost-24-ene-22,26-diol (S8-(22*S*)) (400 MHz, $CDCl_3$, 298 K).

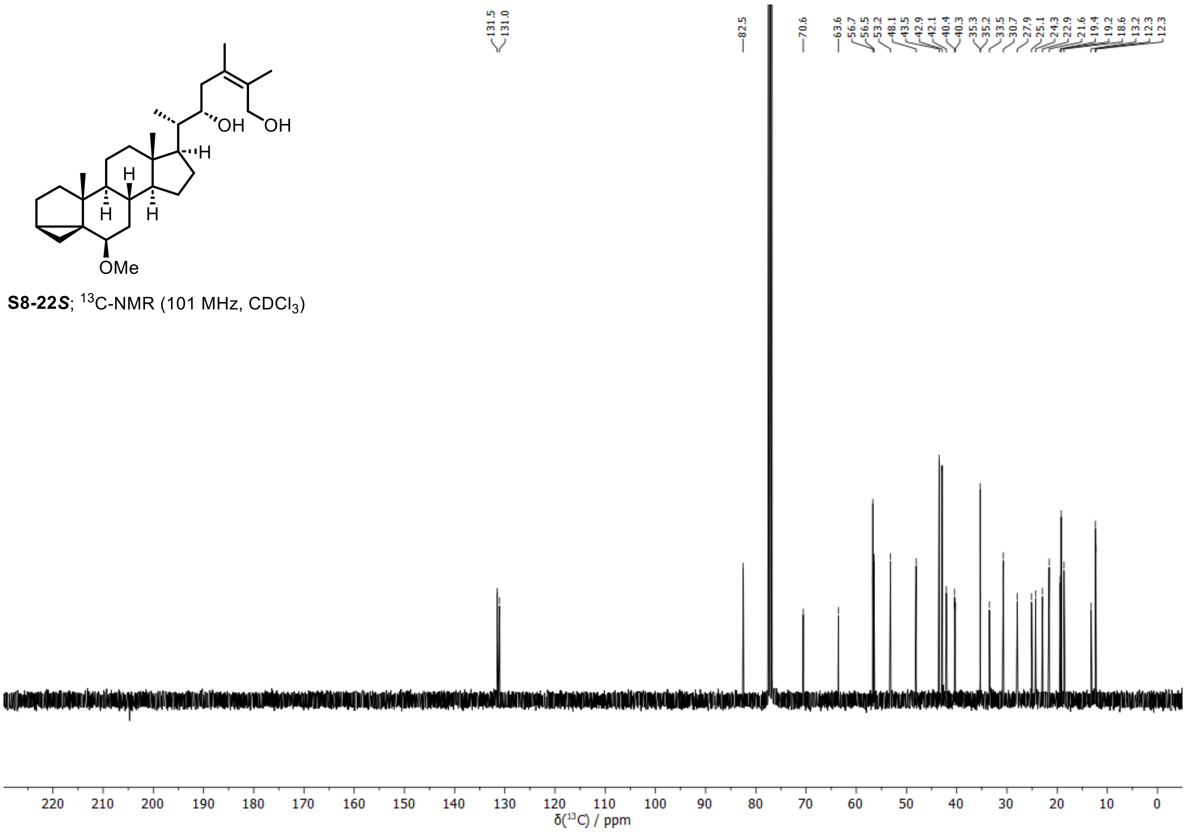

Supplementary Fig. 91. ^{13}C spectrum of (22*S*)-6*β*-methoxy-3*α*,5-cycloergost-24-ene-22,26-diol (S8-(22*S*)) (101 MHz, $CDCl_3$, 298 K).

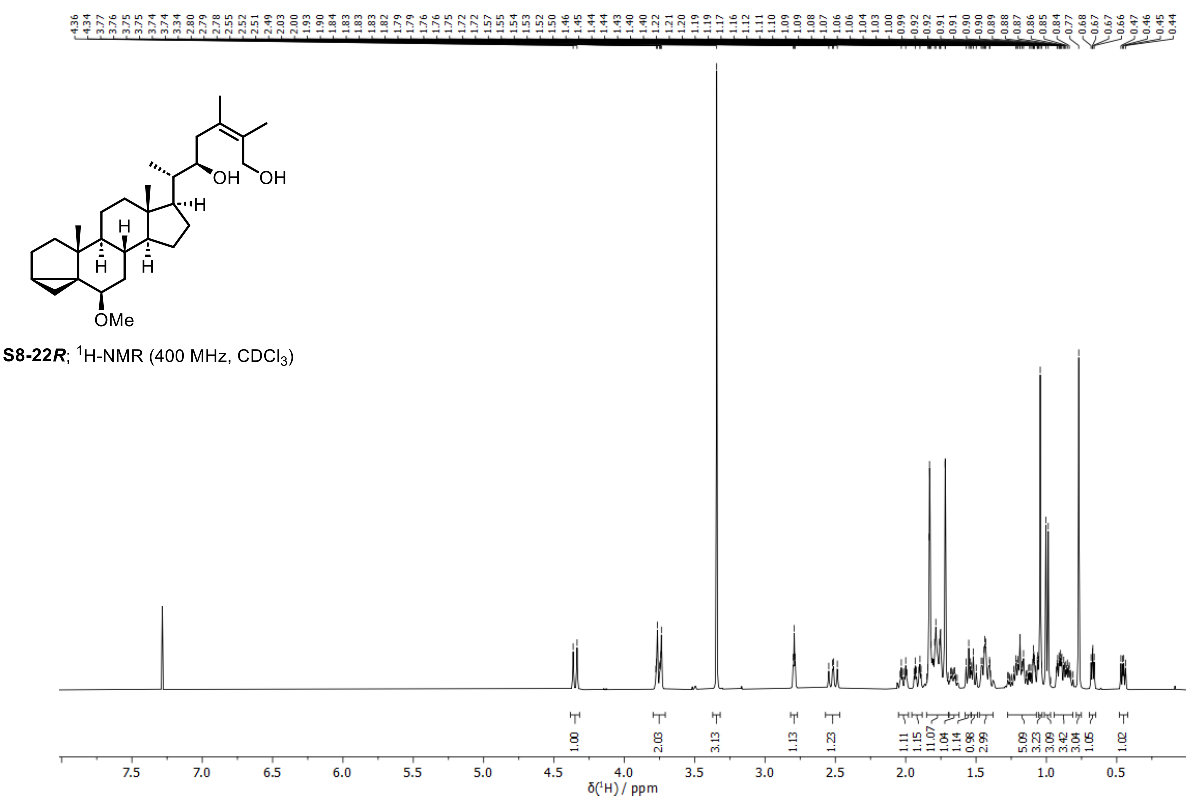

Supplementary Fig. 92. ^{1}H spectrum of (22*R*)-6*β*-methoxy-3*α*,5-cycloergost-24-ene-22,26-diol (S8-(22*R*)) (400 MHz, $CDCl_3$, 298 K).

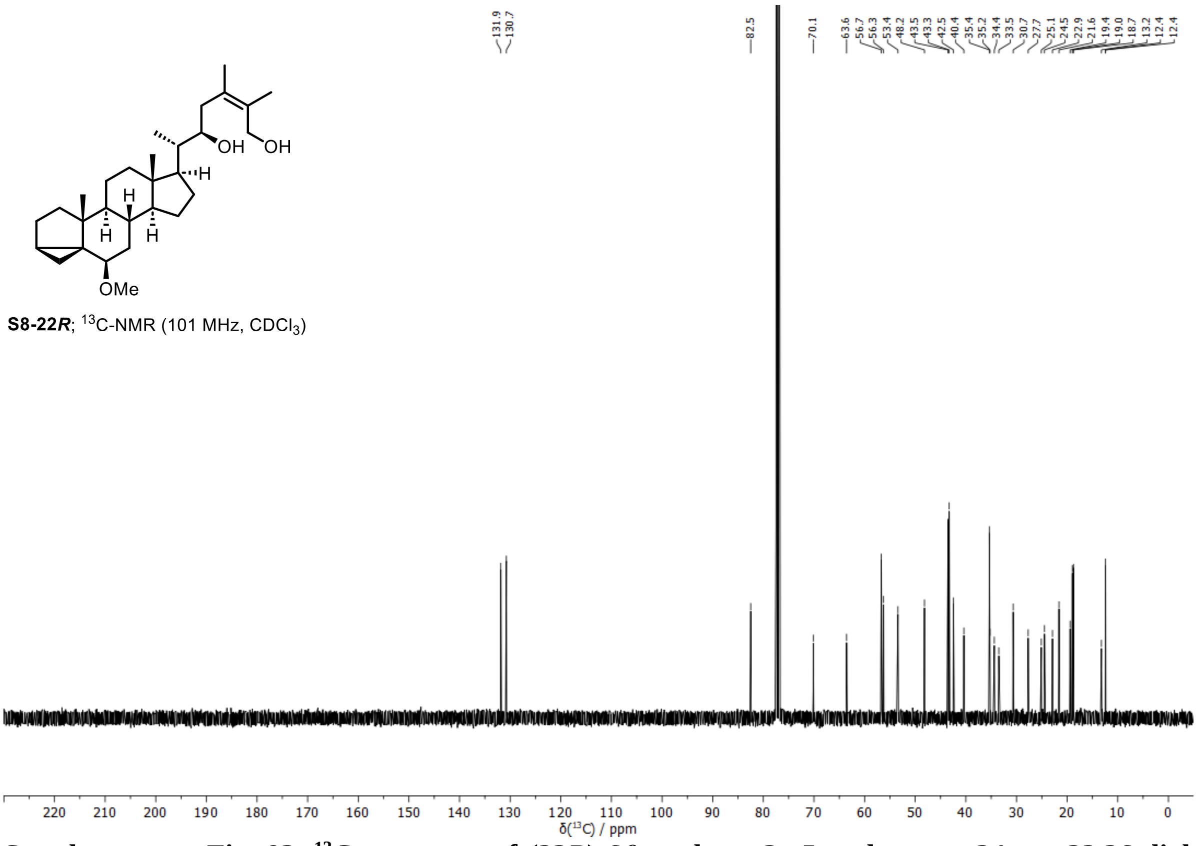

Supplementary Fig. 93. ^{13}C spectrum of (22*R*)-6*β*-methoxy-3*α*,5-cycloergost-24-ene-22,26-diol (S8-(22*R*)) (101 MHz, $CDCl_3$, 298 K).

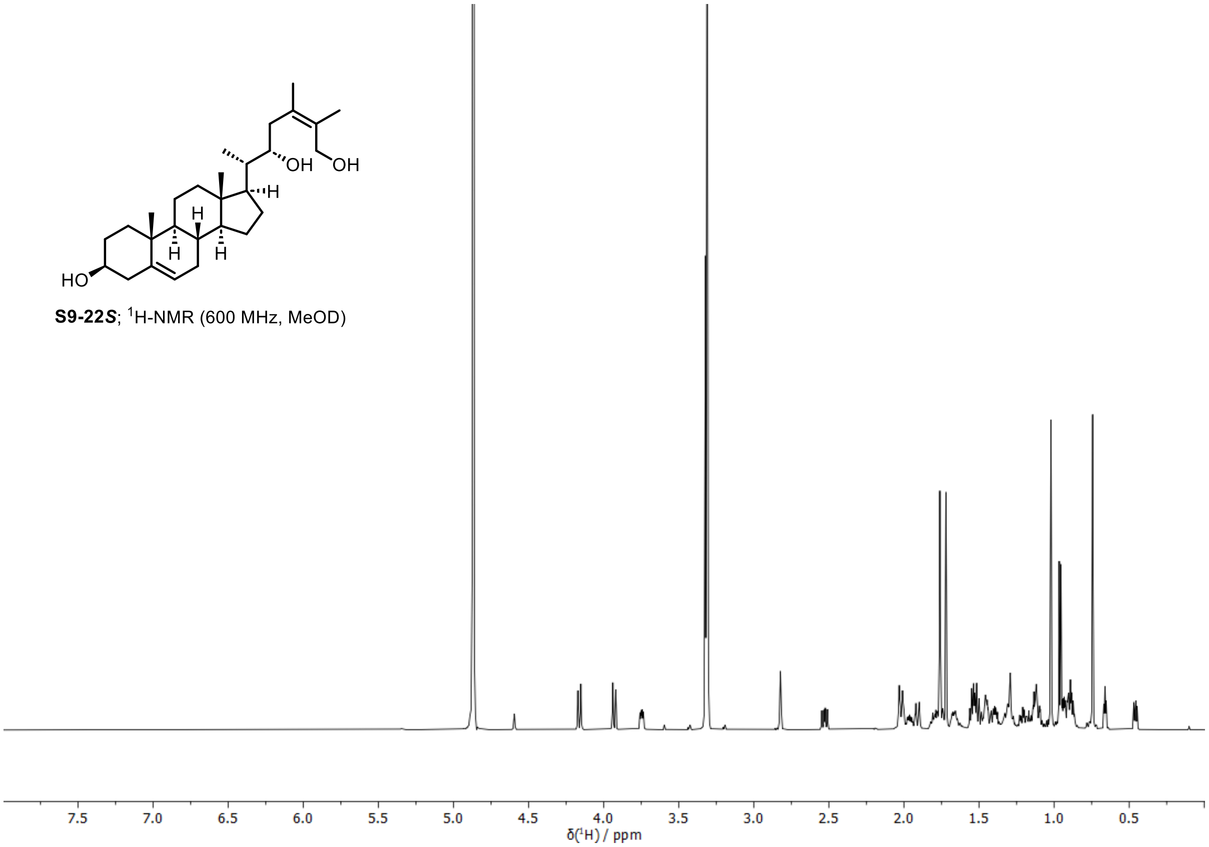

Supplementary Fig. 94. ¹H spectrum of (22*S*)-ergosta-5,24-diene-3*β*,22,26-triol (S9-(22*S*)) (600 MHz, MeOD, 298 K).

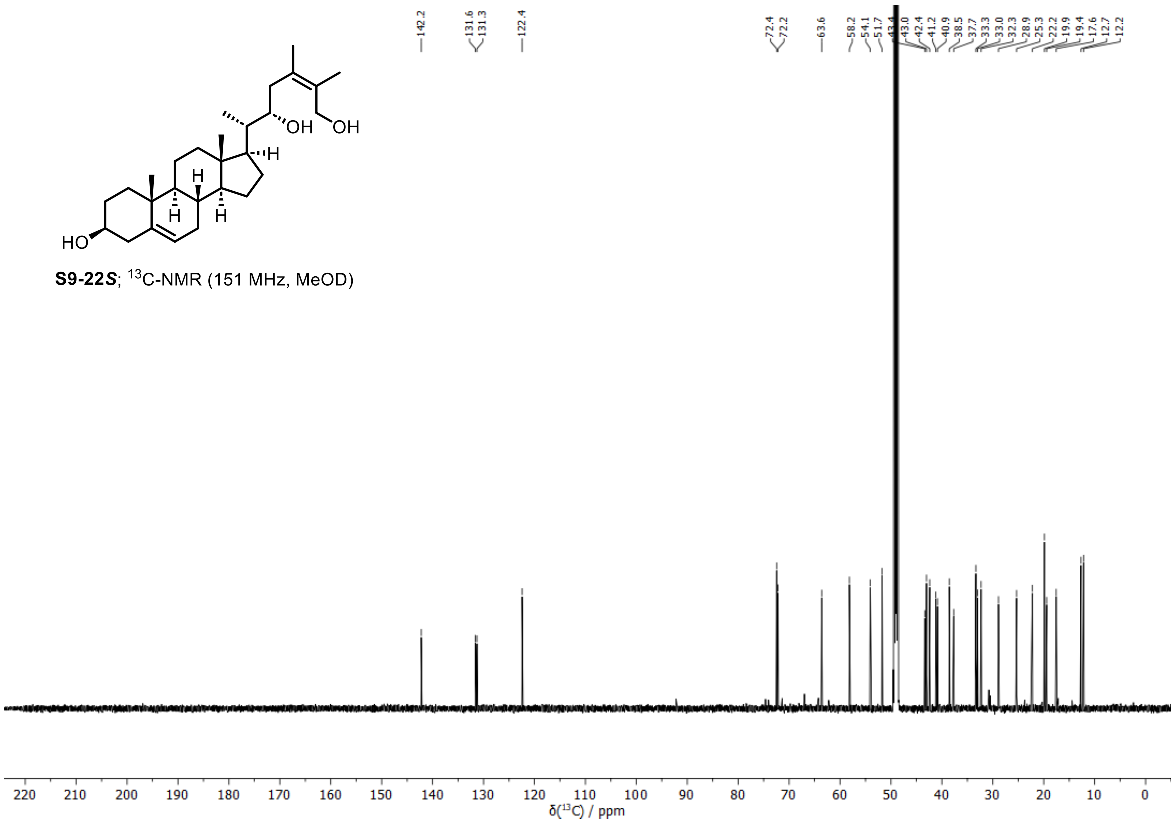

Supplementary Fig. 95. ¹³C spectrum of (22*S*)-ergosta-5,24-diene-3*β*,22,26-triol (S9-(22*S*)) (151 MHz, MeOD, 298 K).

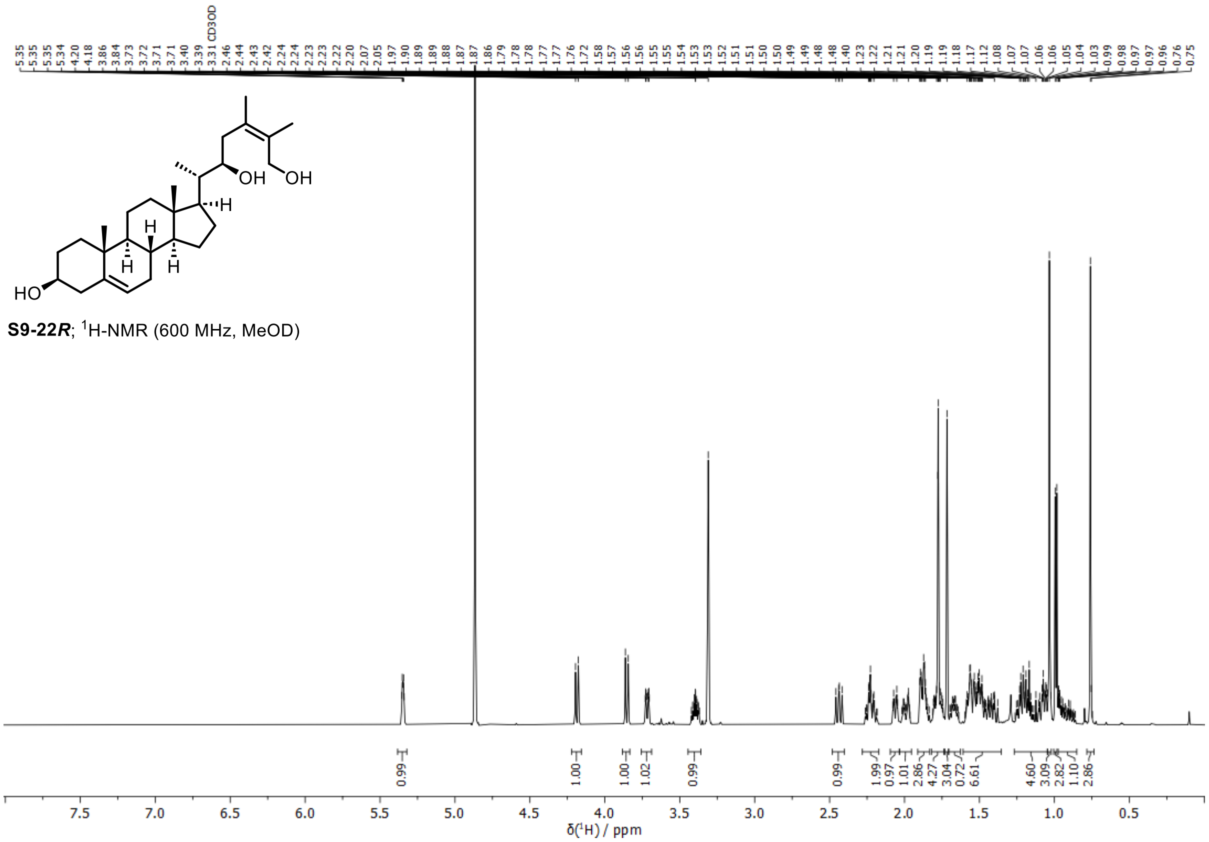

Supplementary Fig. 96. ^{1}H spectrum of (22*R*)-ergosta-5,24-diene-3*β*,22,26-triol (S9-(22*R*)) (600 MHz, MeOD, 298 K).

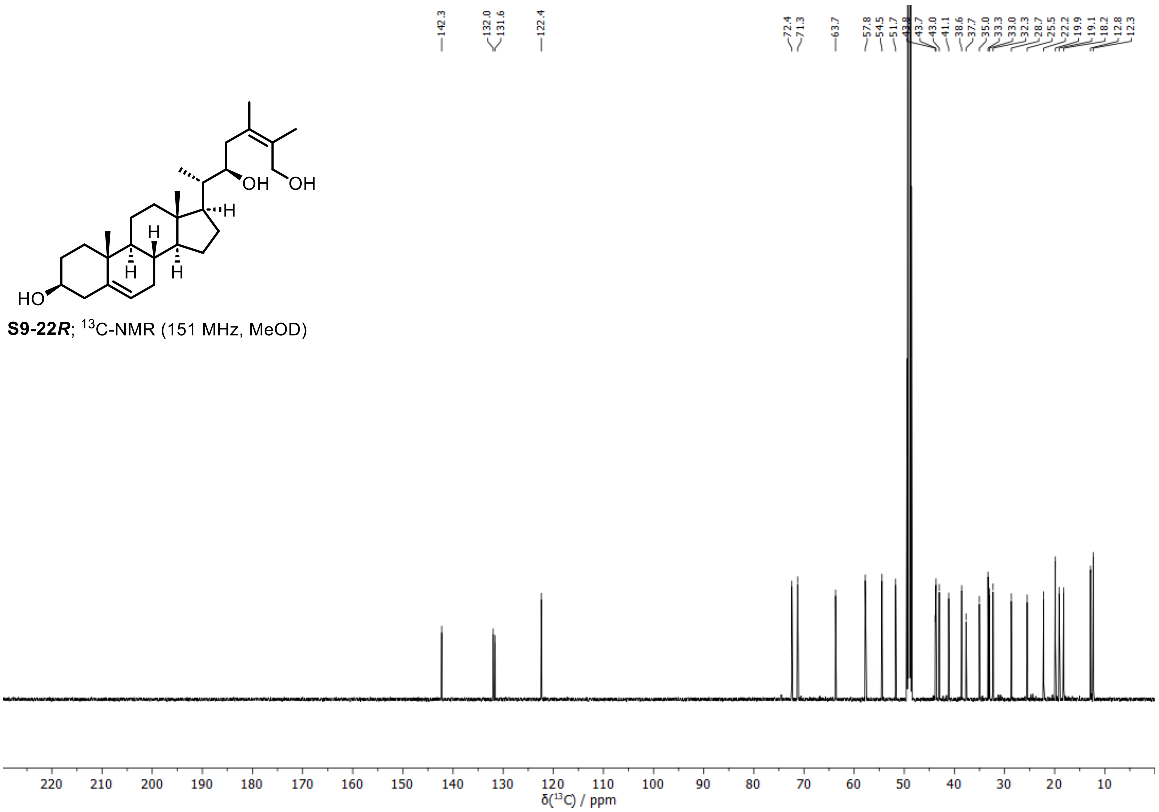

Supplementary Fig. 97. ^{13}C spectrum of (22*R*)-ergosta-5,24-diene-3*β*,22,26-triol (S9-(22*R*)) (151 MHz, MeOD, 298 K).

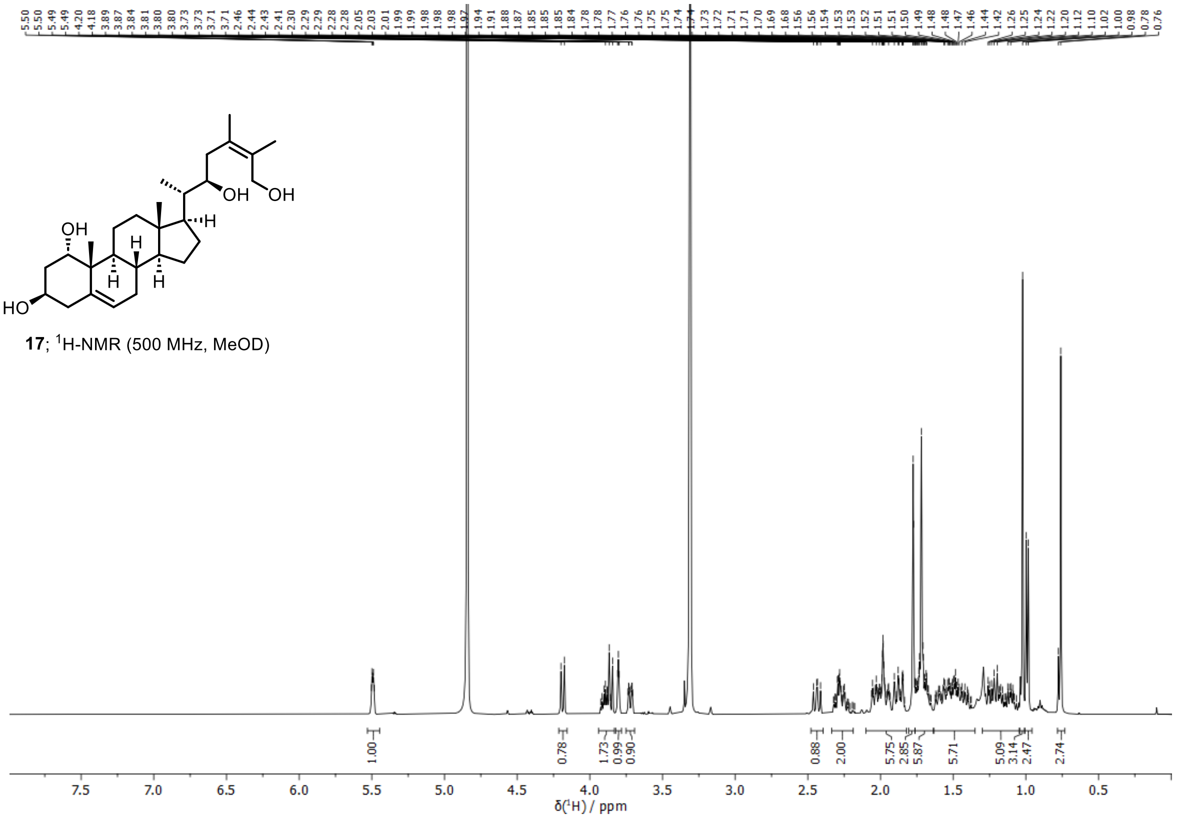

Supplementary Fig. 98. ^{1}H spectrum of (22*R*)-ergosta-5,24-diene-1α,3β,22,26-tetrol (17) (500 MHz, MeOD, 298 K).

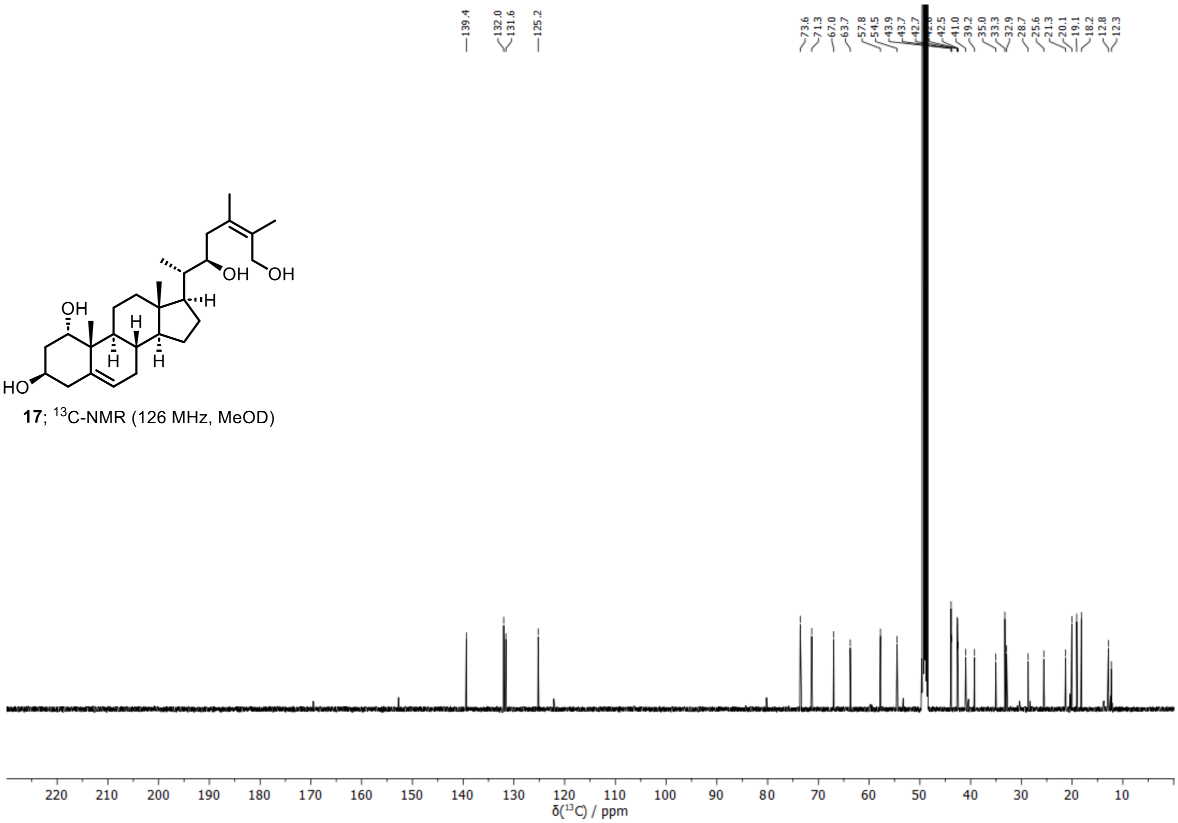

Supplementary Fig. 99. ^{13}C spectrum of (22*R*)-ergosta-5,24-diene-1α,3β,22,26-tetrol (17) (126 MHz, MeOD, 298 K).

Supplementary Table 1. Assembly statistics of *W. somnifera* genome.

Complete and single-copy BUSCOs (S), complete and duplicated BUSCOs (D), fragmented BUSCOs (F), and missing BUSCOs (M).

Parameter	NextDenovo2 assembly
Total number of contigs	93
Average contig length (bp)	28,223,445
Maximal contig length (bp)	152,231,989
N50 (bp)	71,322,777
N90 (bp)	21,319,378
GC Content (%)	38.34
Total length of contigs (bp)	2,878,791,398
BUSCO genes (Solanales_odb12)	97.7% [S:51.4%, D:46.3%], F:0.8%, M:1.5%

Supplementary Table 2. Comparison of *W. somnifera* genome sequence from this study against available NCBI GCA_039654485.1 genome.

The estimated genome size of *W. somnifera* is 2.94 Gb. The following table shows that our assembled genome sequence is more complete and more contiguous compared to the previously available assembly.

**Parameter**	**This study**	**GCA_039654485.1** [22]
Assembly size	2.88 Gbp	1.98 Gbp
Number of contigs/scaffolds	93	1,077,956
N50	71 Mbp	80 kb
BUSCO (solanales_odb12)	97.7%	91.8%

Supplementary Table 3. Comparison of NMR data of (22*R*)-ergosta-5,24(28)-diene-3β,22,25-triol (phyministerol A) (12) with literature data[24].

Discrepancies besides systematic differences in chemical shifts are highlighted in red and described in the footnotes.

**Pos.**	**Our ^{13}C data** C_5D_5N 151 MHz, 298 K	**Reference ^{13}C data**[24] C_5D_5N 125 MHz	**Shift Δ** (ppm)	**Our ^{1}H data** C_5D_5N 600 MHz, 298 K	**Reference ^{1}H data**[24] C_5D_5N 500 MHz	**Shift Δ** (ppm)
**1**	38.3	37.6	0.7	1.17 m<br>1.87 m	1.13 m<br>1.83 m	0.04<br>0.04
**2**	33.1	32.4	0.7	1.85 m [b]<br>2.11 m	1.11 m<br>2.07 m	0.74<br>0.04
**3**	71.7	71.0	0.7	3.88 m	3.83 m	0.05
**4**	44.0	43.3	0.7	 2.66 m	2.59 m<br>2.61 m	 0.05
**5**	142.4	141.8	0.6			
**6**	121.6	121.0	0.6	5.45 d (5.0)	5.41 d (5.0)	0.04
**7**	32.6	32.0	0.6	1.62 m [b]<br>1.98 m	 1.96 m	 0.02
**8**	32.6 [a]	32.7 [a]	0.1	1.45 m [b]	1.56 m	0.11
**9**	51.0	50.3	0.7	1.01 m	0.99 m	0.02
**10**	37.3	36.9	0.4			
**11**	21.8	21.2	0.6	1.44 m<br>1.54 m	1.39 m<br>1.49 m	0.05<br>0.05
**12**	40.4	39.8	0.6	1.19 m<br>2.04 m	1.17 m<br>2.01 m	0.02<br>0.03
**13**	43.3	42.6	0.7			
**14**	56.9	56.3	0.6	0.97 m	0.96 m	0.02
**15**	25.1	24.5	0.6	1.07 m<br>1.60 m	1.04 m<br>1.57 m	0.03<br>0.03
**16**	28.3	27.6	0.7	1.42 m<br>1.94 m	1.39 m<br>1.91 m	0.03<br>0.03
**17**	54.1	53.5	0.6	1.30 m	1.27 m	0.03
**18**	12.4	11.8	0.6	0.67 s	0.65 s	0.02
**19**	20.0	19.4	0.6	1.07 s	1.03 s	0.04
**20**	43.4	42.7	0.7	2.04 m	2.00 m	0.04
**21**	13.4	12.8	0.6	1.24 d (6.7)	1.21 d (7.0)	0.03
**22**	75.1	74.4	0.7	4.19 m	4.16 m	0.03
**23**	33.3 [a]	32.0 [a]	1.3	2.49 d (14.2) [b]<br>2.64 m [b]	1.86 m<br>1.96 m	0.63<br>0.68
**24**	157.5	156.9	0.6			
**25**	72.5	71.9	0.6			
**26**	30.7	30.1	0.6	1.67 s	1.64 s	0.03
**27**	31.4	30.8	0.6	1.61 s	1.58 s	0.03
**28**	110.0	109.5	0.5	5.22 br s<br>5.36 br s	5.19 br s<br>5.33 br s	0.03<br>0.03

[a] Possibly swapped assignments of C8 and C23 in ref. [24]. Our assignments are supported by HMBC correlations.

[b] Our proton assignments are supported by HSQC and COSY correlations.

Supplementary Table 4. The closest homologues of CYP genes putatively linked to withanolide biosynthesis based on gene silencing by others [25,26] are part of the withanolide gene clusters.

CYP gene (reference)	Match type	Bait sequence from literature	Best BLAST hit					
			Species	Position in genome	Gene ID	Gene name	Identity (%)	E-value
WsCYP749B1 (ref. [25])	Protein sequence	MMIAVIAFSVFLIGVVVLGRYLYKSWWYPISLQHLMNSQGIKGPRYEFGNGNSRATAEILMKFNNAPMDISHDIFPRLQPHFRSWIKLYGSTFLYWMNSTKPQLVVSDVELIKEIFTNKQDSFGKAKFDGILKRFVGDGSVFQKGHKWLKLRKVADNVFHAQSLKDMLPAMVGRVESMLKTWKSYEGKEIEVFEEFRLLSLEMISNSVFGNDYSTGKHIFSMLDKIAYISAMSYGKSRNPIIDKLFRSSEEIQADKILEELSDSFAGIIKKREDRVKAGEANNFGDDFLGSLLEGRFNADENARISVDEIIEECKSFYFAGHKTVTSLLSWSMLLLASNTDWQERAKNEVLEVLGQENPKAESISRLKTVGMIINEALRLYPPFILLQRDVTKNTSLGKLKVPAGTEVIIAILAVHHNSEIWGEDAHLFKPERFAEGVSKATRDQVMAFLSFGYGLRKCVGFNFATMEVKIALSMILQRYRLTVSPNYTHSPIATFTLHPSNGIQIMLHPL	*Withania somnifera*	N/A	Wsom003G00477.1	WsCYP749B-2b	99.61	0.0
				N/A	Wsom003G00478.1	WsCYP749B-2a	98.83	0.0
				N/A	Wsom003G00475.1	WsCYP749B-2c	98.24	0.0
				N/A	Wsom090G00485.1	WsCYP749B-a	89.24	0.0
				N/A	Wsom090G00473.1	WsCYP749B-b	85.80	0.0
PB.11591.2 (ref. [26])	Protein sequence	MIPSDLVFDFTWKLVAVVLMVLLVRGFWRTYVSKFSFMYGNREDVETDVEAGPVPRTPLLSLRYSLHHAPMLSANSDTKLAMKGISHGACDYLVKLVRIEELRNIWQHIIRRKKVEPKMEYNLVFLSTAFAVGILTLISVLKRANGWFYSMKFSSEKCRLPPGDMGWPVVGNMLFFVKCLSAYDLKSFVSYFVTRFGQGGMYRTFMFGKPSVIVTTPELCRKILMDDENFDLGFPSYILELLRKEPIGGTSYQEDRLSRRLMTPIKSHALVSYFFDFLSETVQTTFEKWATTGESLQLLFEMKKPTFKVLMQVLIGGDQVENKLLDTLFKENNFRFAGLRSLPLDYPGSTYNRAMKGRGEIVKIYERIINERKVMIAKTRGEPRTNLLDIMLDSQYDGEGKVLNDENIMKVLLWYTFSGYESIAKVATQTIMLLEKHPECFKKAKEEQEEIVKRRSSPDAGLTFGEIGQMKYVNNVINETLRLGSTETVLFRDARTDVNINGYTIPKGWKVLALLGNLYMDPKTYVKPKEFNPSRWDDFETKPNSFIPFGVGLRLCPGSNLVRLEVSVFLHYFLLNYRLEQLNKDSKAEACIAKFKKISA	*Physalis grisea*	N/A	Pgri02031.1	PgCYP88C-d	99.17	0.0
			Physalis pruinosa	N/A	Ppru02037.1	PpCYP88C-d	99.17	0.0
PB.29095.11 (ref. [26])	Primer (forward)	ATTGGAGCACTGGTTCTTGGTAGG	*Physalis grisea*	Chr1:114882829-114882806	Pgri02022.1	PgCYP749-a	100.00	3.26e-05
				Chr1:115027450-115027473	Pgri02030.1	PgCYP749-b	100.00	3.26e-05
			Physalis pruinosa	Chr1:116023998-116023975	Ppru02028.1	PpCYP749-a	100.00	3.28e-05
				Chr1:116168631-116168654	Ppru02036.1	PpCYP749-b	100.00	3.28e-05
PB.29095.11 (ref. [26])	Primer (reverse)	GTTCCCACTGCCAAATTCGTAAGG	*Physalis grisea*	Chr1:114882710-114882733	Pgri02022.1	PgCYP749-a	100.00	3.26e-05
				Chr1:115027569-115027546	Pgri02030.1	PgCYP749-b	100.00	3.26e-05
			Physalis pruinosa	Chr1:116023879-11602390	Ppru02028.1	PpCYP749-a	100.00	3.28e-05
				Chr1:116168750-116168727	Ppru02036.1	PpCYP749-b	100.00	3.28e-05

Supplementary Table 5. Genomic locations of closest homologues of protein sequences reported in connection with withanolide biosynthesis in *Withania somnifera* in previous studies.

Gene (reference)	Sequence	Best blast hit				
		Gene ID	Sequence	contig	Identity (%)	E-value
WsCYP71B35 ([27])	MEIYQLFNSVAVFLLFIFLLLRKWKAPKLKLPPGPWKLPFIGSLHHLALSGPIPHHGLTNLAKRYGPLMSLQLGEVIMIVISSPRMAKEVLKTQDLVFATRPKKLTYADIVHYGSTDVVFSPYGAYWRQIRKVCILELLSAKMVNNFVSSIRQDGLSNIISSIRSKSNLSVNLTEKIIWFTSSVTCRSAFGKIRNEHQDKLIYLQKEMLSLSIAVNLADFFPSWKFLHDLDGSKSRLLKVHGKVDEILEYVVNEHKQNRANGKRGNGEFGGEDLIDVMLRIRESGELQVPISDDNIKAIILDMFSAGSETTSTTINWAFAEMMKNPGALAKAQAEVRRSLKGKKDFQQIDLDELKYLKLVIKETLRLHPPIPLFVPRECMEQTKIDGYDIPLKARVLVNAWAIGRDPESWDDPESFLPERFENSAVDFTGNHHQFIPFSSGRRMCPGMLFGLANVGQLLAQLLYHFHWKLPNGQSHLSLDMTESPGVSATKKDDLILIATPYYS	Wsom075G00351.1	MEIYQLFNSVAVFLLFIFLLLRKWKAPKLKLPPGPWKLPFIGSLHHLALSGPIPHHGLTNLAKRYGSLMSLQLGEVIMIVISSPRMAKEVLKTQDLVFATRPKKLTYADIVHYGSTDVVFSPYGAYWRQIRKICILELLSAKMVNNFVSSIRQDELSNIISSIRSKSNLSVNLTEKIIWFTSSVTCRSAFGKIRNEHQDKLIYLQKEMLSLSIAVNLADFFPSWKFLHDLDGSKSRLLKVHGKVDEILEYVVNEHKQNRANGKRGNGEFGGEDLIDVMLRIRESGELQVPISDDNIKAIIIDMFSAGSETTSTTINWAFAEMMKNPGVLAKAQAEVRQSLKGKKDFQQIDLDELKYLKLVIKETLRLHPPIPLFVPRECMEQTKIDGYDIPLKARVLVNAWAIGRDPESWDDPESFLPERFENSPVDFTGNHHQFIPFSSGRRMCPGMLFGLANVGQLLAQLLYHFHWKLPNGQSHLSLDMTESPGVSATKKMILF	ctg075	98.577	0.0
WsGT4 ([28])	MEKLLNKSHVLVLPFPVQGHINPMVQFSKRLASKGSIKVTLITIDSISKSMPILEYGPIKIYSIPHNDSPPQSYDQFLEWFRLLVSINLKKIVETLSDSEYPMRLLVYDSITTWAIDLAHDLGLKGAAFFTQSCALSVIYYHMDPENRKVGYDGCGGVSLPSLPLLEKQDLPSFVCQSDLYPSLSKLVFSRNINFKKADWLLFNSFDVLEKEVIDWLRTQCRIKTIGPIVPSLYLDKRLKDDKEYGLSLFKPNSETCMKWLDSREIDSVVYVSFGSLASLGEQQMEELASGLMMSDCYFLWVVRATEENKLTEELLSKAKEKCLIVNWSPQLDVLAHQAVGCFFTHCGWNSTLEALSLGVPMVAMPQWSDQPTNAKFICDVWQTGVRVKAGENGIITREEVASSIKEVMKEEKGVMLKENAIKWKNLAKEAVDEGGSSDKNIEEFLKIVMY	Wsom026G00494.1	MEKLLNKSHVLVLPFPVQGHINPMVQFSKRLASKGSIKVTLITIDSISKSMPILEYGPIKIYSIPHNDSPPQSYDQFLEWFRLLVSINLKKIVETLSDSEYPMRLLVYDSITTWAIDLAHDLGLKGAAFFTQSCALSVIYYHMDPENRKVGYDGCGGVSLPSLPLLEKQDLPSFVCQSDLYPSLSKLVFSRNINFKKADWLLFNSFDVLEKEVIDWLRTQCRIKTIGPIVPSLYLDKRLKDDKEYGLSLFKPNSETCMKWLDSREIDSVVYVSFGSLASLGEQQMEELASGLMMSDCYFLWVVRATEENKLTEELLSKAKEKCLIVNWSPQLDVLAHQAVGCFFTHCGWNSTLEALSLGVPMVAMPQWSDQPTNAKFICDVWQTGVRVKAGENGIITREEVASSIKEVMKEEKGVMLKENAIKWKNFAKEAVDEGGSSDKNIEEFLKIVMH	ctg026	99.557	0.0
WsGT6 ([28])	MAQPHVLLVTFPAQGHINPSLQFAKRLIKLGIGVTFATSIFAHRRMAKATTSNALKGLNFAGFSDGYDDGSKPDMDDPKRYMSEIRSRGSQTIRDILLKSSDKGRPVTSLVYTLLLPWAAEVARELHIPSALLWIQPATVLDIYYYYFNGYEDDIKCSSNDPNWCIQLPRLPLLKSQDLPSFLLSSSAEHAKYSFALPTFKEQLDTLDGEENPKVLLNTFDALELDALKAIEKYNLIGIGPLIPSSFLDGKDPLDSSFGGDLFQKSNDYMEWLDSKPKASIVYISFGSILSLSRNQKEEIAKGLIEIKRPFLWVIRDQDNGKEVEKEEEELSCMMELEKQGKIVPWCSQLEVLTHPSLGCFVSRCGWNSTLESLSLGVPMVAFPHWTDQGTNAKLIEDAWKTGVRLRVNEDGVVESEEIIRCIELVMDGGEKGEEMRKNAQKWKELAREAVKEGGSSEMNMKAFVQQVGKGC	Wsom021G00073.1	MAQPHVLLVTFPAQGHINPSLQFAKRLIKLGIEVTFATSIFAHRRMAKATTSNALKGLNFAGFSDGYDDGFKPDMDDPKRYMSEIRSRGSQTIRDILLKSSDKGRPVTSLVYTLLLPWAAEVARELHIPSALLWIQPATVLNIYYYYFNGYEDDIKCSSNDPNWCIQLPRLPLLKSQDLPSFLLSSSAEHAKYSFALPTFKEQLDTLDGEENPKVLLNTFDALELDALKAIEKYNLIGIGPLIPSSFLDGKDPLDSSFGGDLFQKSNDYMEWLDSKPKASIVYISFGSILSLSRNQKEEIAKGLIEIKRPFLWVIRDQDNGKEVEKEEEELSCMMELEKQGKIVPWCSQLEVLTHPSLGCFVSHCGWNSTLESLSLGVPMVAFPHWTDQGTNAKLIEDVWKTGVRLRVNEDGVVESEEIIRCIELVMDGGEKGEEMRKNAQKWKELAREAVKEGGSSEMNMKAFVQQVGKGC	ctg021	98.941	0.0
WsCYP71B10 ([25])	MMFFPLFVALLIILLSFLLRKAKRKGKDNLPPGPLGLPLIGNLHQYDSLTPHIYFWKLSKKYGKIFSLKLGSANMVVVSSVNLAKEVLKIQDLVYCSRPSLLGLQKLSYNGQDIGLSPYNDYWRELRKICIIHLFSLKKVQCFSPIREDEVSRMIKKISQQATTSQVTNLSNIVISLTTSIICRVAFGITFDGETQERRKFDEVLKVAEEMLAGFFISDYFPLLGWVDKLTGKINRLEKNFKDLDEFYEGLIEQHLSPNRPKSMEGDIVDLLLQLKKQQSTPIDLTLDNIKGIIMNMLIGGRDTTAATVIWAMTALIANPNALKKVQAEIRESVRKTSIVNENDVQNLSYFNAVIKETFRLYPPGPLLIARETMQNSTLEGYEIKQKTIVHVNFWAIARDPEYWENPEKFIPERFLNSDIDFKGQNFELIPFGAGRRGCPAMALGVATVELILSNLLYAFNWELPCGMKKDDIDTDVEPGLTMHKKTPLCLVPRNYH	Wsom021G00065.1	MMFFPLFVALLIILIRFLLRKAKRKGKDNLPPGPLGLPFIGNLHQYDSLTPHIYFWKLFKKYGKIFSLKLGSANMVVVSSANLAKEVLKIQDLVYCSRPSLLCLQKLSYNGHDIGFAPYNDYWRELRKICVIHLFSLKKVQCFSPIREDEVSRMIKKISQQATTSQVTNLSNIVISLATSIICRVAFGITFDGETQERRKFDEVLKVTEEMLAGFFISDYFPLLGWVDKLTGKINRLEKNFKDLDEFYEGLIEQHLSPNRPKSMEGDIVDLLLQLKKEQSTPIDLTLDNIKGIIMNMLIGGRDTTAATVIWAMTALIANPNALKKVQAEIRESVRKTSIVNENDVQNLSYFKAVIKETFRLYPPGPLLIARETMRNSTLEGYEIKQKTIVQVNIWAIARDPEYWENPEKFIPERFLNSDIDFKGQNFEFIPFGAGRRGCPAMALGVATVELILSNLLYAFNWELPSGMKKDDIDTDVLPGLTMHKKTPLCLVPRNYP	ctg021	95.968	0.0
WsCYP76 ([25])	MFQLGTEPFKAMACLKQKYGPVLWLKLGTSTNIMVVQTARAAAELFKNHDISFADRFIPIVNQAHNYCQGSMALCQYGSYWRFQRRICTVEMFIHKKISETVPVRRKCVDNMLKWIGKAANSAEKGSGIEVTRFVFLTSFNMLGNLILSKDLADPESEEASEFFNAMKGIMVWSGVANVSDIFPFLRKFDLQNLRKKMERDMGKAMEIASIFLKEREEERKKGAEKIGKDFLDALLEFEGTGKDEPAKLSEHEIKVLLVEMFLAGTETTSSSVEWALAELLRHPQAMDKVKTEISKLIGPNRKFEENDIDNLPYMQAVIKESLRLHPPLPLLIPRETVHDTKFMGYDVPKGTRVLVNAWEIGRDPECWDDPMSFKPERFLGSKVDMKGQHYELIPFGAGRRMCVGLPLGHRMMHFALGSLLHEFEWELPDGVSPKSINMDVSMGITARKQESLKVIPKKLETLINLI	Wsom046G01543.1	MEWEWSYLFFSTIILLPAFILLFSQKKATKCSYKLPPGPPGLPIFGNMFQLGTEPFKAMACLKQKYGPVLWLKLGTSTNIMVVQTARAAAELFKNHDISFADRFIPIVNQAHNYCQGSMALCQYGSYWRFQRRICTVEMFIHKKISETVPVRRKCMDNMLKWIGKAANSAEKGSGIEVTRFVFLASFNMLGNLILSKDLADPESEEASEFFNAMKGITVWSGVANVSDIFPFLRKFDLQNLRKKMERDMGKAMEIASIFLKEREEERKKGAEKIGKDFLDALLEFEGTGKDEPAKLSEHEIKVLLVEMFLAGTETTSSSVEWALAELLRHPQAMDKVKTEISKLIGPNRKFEENDIDNLPYMQAVIKESLRLHPPLPLLIPRETVHDTKFMGYDVPKGTRVLVNAWAIGRDPECWDDPMSFKPERFLGSKVDMKGQHYELIPFGAGRRMCVGLPLGHRMMHFALGSLLHEFEWELPDGVSPKSINMDVSMGITARKQESLKVIPKKLETLINLI	ctg046	99.143	0.0
WsCYP710A11 ([29])	MASIWVLISPWIPYFFSFIALLLLLEQIFYLKKKRFLPGPNLVLPFFGSVFSLVTNPTKFWELQSALAKSTEHGFSANYIIGKFILYIHSTDLSHKVFANVRPDAFHLIGHPFGKKLFGEHNLIYMFGQEHKELRRRIAPNFTPKALASYTDIQQRIIIKYFKSWLDQAAKSPNTPIPLRFLCRDLNLDTSQTVFVGPYLNEESRKQFNVDYNYFNVGLMNLPFDLPGFAFRNARLAVGRLVETLSVCVEQSLNKMQSNEEPTCLIDFWMQENIREIEEAKINGLQKPFEYTNKELGGYLFDFLFAAQDASTSALLWAIMLLDSHREILERVREEVAKFWSPESEQPLTADMLREMKYLEAVAREIVRIRAPATMVPHIAGEEFRLTEDYVIPKGTIVFPSVFDSSFQGFPEPEKFDPDRFMEERQEERVYKKNFLAFGAGPHACVGQKYAINHLMLFVAIFTALIDFKRHKTDGCDDISYIPTIAPKDDCKVFLSQRCTPFPSLS	Wsom004G01279.1	MASIWVLISPWIPYFFSFIALLLLLEQIFYLKKKRFLPGPNLVLPFFGSVFSLVTNPTKFWELQSALAKSTEHGFSANYIIGKFILYIHSTDLSHKVFANVRPDAFHLIGHPFGKKLFGEHNLIYMFGQEHKELRRRIAPNFTPKALATYTDIQQRIIIKYFKSWLDQAAKSPNTPIPLRFLCRDLNLDTSQTVFVGPYLNEESRKQFNVDYNYFNVGLMNLPFDLPGFAFRNARLAVGRLVETLSVCVEQSLNKMQSNEEPTCLIDFWMQENIREIEEAKINGLQKPFEYTNKELGGYLFDFLFAAQDASTSALLWAIVLLDSHREILERVRAEVAKFWSPESEQPLTADMLREMKYLEAVAREIVRIRAPATMVPHIAGEEFRLTEDYVIPKGTIVFPSVFDSSFQGFPEPEKFDPDRFMEERQEERVYKKNFLAFGAGPHACVGQKYAINHLMLFVAIFTALIDFKRHKTDGCDDISYIPTIAPKDDCKVFLSQRCTPFPSLS	ctg004	99.407	0.0
WsCYP85A69 ([30])	MAFFLVFLASFFGLCIFSTSLLRWNQVKYNNKNLPPGTMGWPLFGETTEFLKLGPSFMKNQRARYGSFFKSHILGCPTIVSMDAELKRYNEAKGLVPGYPQRLLDIQGKCNIAAVNGSAHKYRRGALLSHISPTRLRDQLLPKIRMFTRSHLSNWERKVMHIQEKTKEMAFLSSCKQIAGIESSSSAQEFMPEFFKQVQRTLSLPIDLPNTNYHRGFQARKIFVRLRRTQTEERRGSKQIHQNMLGYRTKTEANRFKSTEAERMHGIITLLYSGTKTVSTTSRLAEKYLHDHPKVQEELKEEHMAIREKKKPEDPIDYNDYKAMRFTRADLLETSRFAREANGVRRKTTRDMERNGYIIPGGWRIYVVYTREGNYDPRVYPEPYTFNPWRWMDRSQEHQNSFELFGGGTRQCPGKELGDPEISTFLHYFVTKYRWEEVGGEPRNKFPRVPAPNGLRKEFQLTL	Wsom005G00737.1	MAFFLVFLASFFGLCIFSTSLLRWNQVKYNNKNLPPGTMGWPLFGETTEFLKLGPSFMKNQRARYGSFFKSHILGCPTIVSMDAELNRYILVNEAKGLVPGYPQSMLDILGKCNIAAVNGSAHKYMRGALLSLISPTMIRDQLLPKIDEFMRSHLSNWDNKVIDIQEKTNKMAFLSSLKQIAGIESSSLAQEFMPEFFKLVLGTLSLPINLPNTNYHRGFQARKIIVRLLRTLIEERRGSKQIHHDMLGYMMNEEANRFKLTDDEMIDLIITLLYSGYETVSTTSMMAVKYLHDHPKVLEELIKEHMAIREKKKPEDPIDYNDYKAMRFTRAVILETSRLAIVVNGVLRKTTRDMEINGYIIPKGWRIYVYTRELNYDPRVYPEPYTFNPWRWMDKSLEHQNSFLVFGGGTRQCPGKELGVAEISTFLHYFVTKYRWEEVGGDKLMKFPRVEAPNGLRIRVSTH	ctg005	84.279	0.0
WsCYP93Id ([31])	MMLFLLFLLLALPIILIFLLPKAKEGGKNTLPPGPIGLPLIGNLHQYDSLTPHLYYWKLSKKYGKIFSLKLGSTPMVVVSSAKLAKEVLKTQDLVFCSRPSILGQQKLSYNGREIIFAPYSDYWREMRKICVLHLFSLKKVQSFRPIREDEVSRMIKKISQQAATSKITNLSNLMISLTSTIICRVAFGVRFDEEAHQRKRFDYLLAEAQAGMATFFVSDLFPSLCWIDKLTGLTKRLGRIFKNLDEIYEEIIEQHQNPNRPTSMEGDIVDLLLQLKKEQSTPIDLALEDIKGILMDVLVAGSDTSAAAVVWAMTALIKNPEVMRTVQEEIRKSIGRKGIVTEDDIQNMPYFKLVVKEVFRLYPPVPLLVLRESMKKSKLEGYEIQAGTIIHVNSWAIARDPEIWENPEEFIPERLLNSDIDYKGQDFELIPFGAGRRVCPGIALGVASMELALSNLLYAFDWELPRGMKKEDIDTNVRPGITMHKKNELCLIPKNYL	Wsom021G00061.1	MMLFLLFLLLALPIILIFLLPKAKEGGKNTLPPGPIGLPLIGNLHQYDSLTPHLYYWKLSKKYGKIFSLKLGSTPMVVVSSAKLAKEVLKTQDLVFCSRPSILGQQKLSYNGREIIFAPYSDYWREMRKICVLHLFSLKKVQSFRPIREDEVSRMIKKISQQAATSKITNLSNLMISLTSTIICRVAFGVRFDEEAHQRKRFDYLLAEAQAVMATFFVSDLFPSLCWIDELTGLTKRLGRIFKNLDEIYEEIIEQHQNPNRPTSMEGDIVDLLLQLKKEQSTPIDLALEDIKGILMDVFVAGSDTSAAAVVWAMTALIKNPEVMRKVQEEIRKSIGRKGIVTEDDIQNMPYFKLVVKEVFRLYPPVPLLVLRESMKKSKLEGYEIQAGTIIHVNSWAIARDPEIWENPEEFIPERLLNSDIDYKGQHFELIPFGAGRRVCPGIALGVASMELALSNLLYAFDWELPRGMKKEDIDTNVRPGITMHKKNELCLIPKNYL	ctg021	98.996	0.0

Supplementary Table 6. Gene expression data of previous genes described in Supplementary Table 5.

Ws24ISO genes are used as reference sequences representing withanolide cluster genes discovered in this study. Number of samples (n), Transcripts per million (TPM).

	Leaf (n=12)					Root (n=31)				
	Average exp. (in TPM)	Standard deviation	Pearson's correlation coefficient			Average exp. (in TPM)	Standard deviation	Pearson's correlation coefficient		
			Ws24ISO-a	Ws24ISO-b	Ws24ISO-2a			Ws24ISO-a	Ws24ISO-b	Ws24ISO-2a
Ws24ISO-a	0.76	0.83	1.00	0.13	0.87	5.67	6.09	1.00	0.87	0.92
Ws24ISO-b	100.33	63.99	0.13	1.00	-0.23	67.91	137.86	0.87	1.00	0.85
Ws24ISO-2a	0.11	0.11	0.87	-0.23	1.00	1.41	1.57	0.92	0.85	1.00
WsCYP71B35	3.97	2.29	-0.52	0.51	-0.66	2.23	1.21	-0.13	0.01	-0.14
WsGT4	6.52	2.69	-0.12	0.48	-0.30	8.66	8.75	-0.01	-0.13	-0.13
WsGT6	13.77	17.63	0.38	0.80	0.05	116.56	123.32	-0.21	-0.25	-0.27
WsCYP71B10	21.82	19.39	-0.59	0.48	-0.74	12.82	22.08	0.47	0.54	0.46
WsCYP76	2.52	1.78	-0.53	0.46	-0.62	80.47	69.88	0.08	0.18	0.06
WsCYP71A11	6.24	14.37	0.42	0.67	0.22	4.01	1.98	0.03	0.19	-0.09
WsCYP85A69	0.00	0.01	0.39	-0.29	0.42	0.03	0.06	-0.14	0.02	-0.10
WsCYP93Id	22.94	17.55	-0.58	0.58	-0.74	1.00	1.29	0.49	0.55	0.49

Supplementary Table 7. RNA-seq SRA IDs used for structural annotation.

Withania somnifera	SRR8382569, SRR8382570, SRR8382571, SRR1012863, SRR1012864, , SRR1019197, SRR1019198, SRR1197573, SRR1197746, SRR8205409, SRR8205410, SRR14739595, SRR14739596, SRR14739597, SRR14739598, SRR520142, SRR520143, SRR10985100, SRR10985101, SRR10985102, SRR10985103, SRR10985104, SRR10985105, SRR12022208, SRR12022209, SRR12022210, SRR12022211, SRR12022212, SRR12022213, SRR12022214, SRR12022215, SRR12022216, SRR12022217, SRR12022218, SRR12022219, SRR12022220, SRR12022221, SRR12022222, SRR12022223, SRR12022224, SRR12022225, SRR12022226, SRR12022227, SRR12022228, SRR12022229, SRR12022230, SRR12022231, SRR12052462, SRR12052463, SRR12052464, SRR12052465, SRR12052466, SRR12052467
Physalis grisea	SRR20680386, SRR20680387, SRR20680388, SRR20680389, SRR20680390, SRR20680391, SRR20680392, SRR20680393, SRR20680394, SRR20680395, SRR20680396, SRR20680397, SRR20680398, SRR20680399, SRR20680400, SRR20680401, SRR20680402, SRR20680403, SRR20680404, SRR20680405, SRR20680406, SRR20680407, SRR20680408, SRR20680409, SRR20680410, SRR20680411, SRR20680412, SRR20680413, SRR20680414, SRR20680415, SRR20680416, SRR20680417, SRR20680418, SRR20680419, SRR20680420, SRR20680421, SRR20680422, SRR20680423, SRR20680424, SRR2068042
Physalis pruinosa	SRR7066585, SRR7066587, SRR7066588, SRR7066589, SRR7066590, SRR7066591, SRR7066592

Supplementary Table 8. Source of genomic data sets used for gene prediction, synteny analysis, and phylogenetic tree building.

Note: The gene annotation sequences of *Withania somnifera*, *Physalis grisea,* and *Physalis pruinosa* generated in this study are used in the synteny and phylogenetic analyses as well**.**

Species	Used for			Reference
	Gene prediction	Synteny analysis	Phylogenetic trees	
Atropa belladonna	✓		✓	[32]
Capsicum annuum			✓	[33]
Capsicum baccatum			✓	[34]
Capsicum chinense			✓	[34]
Datura stramonium	✓	✓	✓	[32]
Datura wrightii		✓	✓	[35]
Iochroma cyaneum	✓		✓	[36]
Ipomoea nil			✓	[37]
Lycium barbarum	✓		✓	[38]
Nicotiana attenuata			✓	[39]
Nicotiana otophora			✓	[40]
Nicotiana sylvestris			✓	[41]
Nicotiana tabacum		✓	✓	[40]
Nicotiana tomentosiformis			✓	[41]
Petunia inflata			✓	[42]
Physalis floridana	✓	✓	✓	[43]
Solanum appendiculatum			✓	[44]
Solanum chilense			✓	[45]
Solanum clarkiae			✓	GCA_011800125.2
Solanum dulcamara			✓	[46]
Solanum lycopersicum	✓	✓	✓	[47]
Solanum melongena			✓	[48]
Solanum okadae			✓	[49]
Solanum pennellii			✓	[50]
Solanum pimpinellifolium			✓	[51]
Solanum sitiens			✓	[52]
Solanum stenotomum			✓	[53]
Solanum tuberosum			✓	[54]
Solanum verrucosum			✓	GCF_900185275.1

Supplementary Table 9. List of yeast strains.

At: *A. thaliana*; *Pp*: *P. pruinosa*; *Pper*: *P. peruviana*; ^{opt}: codon-optimised for yeast.

**Strain**	**Genotype**	**Source**
ST7574	MATa; *HIS3; TRP1; LEU2; TRP1; URA3; MAL2-8C SUC2* + pCfB2312 (2µm Cas9 KanMX)	Euroscarf
KMY14	ST7574 XI-1:: *<-T_{ADH1}-Pper7RED-P_{PGK1}-P_{TEF1}-Pper24ISO-T_{CYC1}->*	This work
KMY23	KMY14 + *erg5-Δ1 erg4-Δ1*	This work
KMY48	ST7574 + *erg5-Δ1 erg4-Δ1*	This work
KMY50	KMY23 + X-4:: *<-T_{ADH1}-Pper7RED-P_{PGK1}-P_{GPD1}-upc2-1-T_{CYC1}->*	This work
KMY53	KMY50 + XII-5:: *<-T_{ADH1}-YEH2-P_{FBA1}-P_{TDH3}-ARE2-T_{CYC1}->*	This work
KMY54	KMY50 + XII-5:: *<-T_{ADH1}-YEH1-P_{TEF1}-P_{TDH3}-ARE2-T_{CYC1}->*	This work
KMY55	KMY23 + XI-3:: *<-T_{ADH1}-PpCYP87G1-P_{ADH2}-P_{TEF1}-AtATR1-T_{CYC1}->*	This work
KMY71	KMY23 + X-4:: *P_{GAL1}-AtATR1-T_{CYC1}->*	This work
KMY75	KMY71 + XI-3:: *<-T_{ADH1}-PpCYP87G1^{opt}-P_{TEF1}-P_{TDH3}-PpCYP88C7^{opt}-T_{CYC1}->*	This work
KMY77	KMY23 + *Pper24iso-Δ1*	This work
KMY78	KMY55 + *Pper24iso-Δ1*	This work
KMY82	KMY75 + XII-2:: *<-P_{GAL1}-PpCYP749B2^{opt}-T_{CYC1}->*	This work
KMY83	KMY71 + XII-2:: *<-T_{ADH1}-PpCYP87G1^{opt}-P_{TEF1}*	This work
KMY84	KMY71 + XII-2:: *<-P_{TDH3}-PpCYP88C7^{opt}-T_{CYC1}->*	This work
KMY85	KMY75 + XII-5:: *<-P_{GAL1}-PpSDR-a^{opt}-T_{CYC1}->*	This work
KMY86	KMY75 + XII-5:: *<-P_{GAL1}-PpSDR-b^{opt}-T_{CYC1}->*	This work
KMY87	KMY75 + XII-5:: *<-P_{GAL1}-WsSDR-a-T_{CYC1}->*	This work
KMY88	KMY75 + XII-5:: *<-P_{GAL1}-WsSDR-b-T_{CYC1}->*	This work
KMY89	KMY82 + XII-5:: *<-P_{GAL1}-PpSDR-a^{opt}-T_{CYC1}->*	This work
KMY90	KMY82 + XII-5:: *<-P_{GAL1}-PpSDR-b^{opt}-T_{CYC1}->*	This work
KMY91	KMY82 + XII-5:: *<-P_{GAL1}-WsSDR-a-T_{CYC1}->*	This work
KMY92	KMY82 + XII-5:: *<-P_{GAL1}-WsSDR-b-T_{CYC1}->*	This work
KMY93	BSY1 + XII-5:: *<-P_{ADH2}-PpCYP87G1^{opt}- T_{CYC1}->*	This work
KMY94	BSY1 + XII-5:: *<-P_{ADH2}-PpCYP88C7^{opt}- T_{CYC1}->*	This work
KMY95	BSY1 + XII-5:: *<-P_{ADH2}-PpCYP749B2^{opt}- T_{CYC1}->*	This work
BSY1	ST7574 X-2:: *<-P_{ADH2}-CrCPR-T_{ADH1}, P_{PCK1}-CrCYB5-T_{CYC1}->*	Ref. [55]

Supplementary Table 10. List of plasmids for metabolic engineering in *S. cerevisiae*.

Strain	Description	Reference
pCfB8622	Plasmid containing gRNA expression cassette targeting *ADE2* in *S. cerevisiae* (source plasmid for gRNA modifications)	[58]
pCfB3035	Plasmid for integration of genes and promoters into site X-4	[56]
pCfB3036	Plasmid for integration of genes and promoters into site XI-1	[56]
pCfB2904	Plasmid for integration of genes and promoters into site XI-3	[56]
pCfB2909	Plasmid for integration of genes and promoters into site XII-5	[56]
pCfB3039	Plasmid for integration of genes and promoters into site XII-2	[56]
pKME15	pCfB3036- T_{ADH1}-*Pper7RED*-P_{PGK1}-P_{TEF1}-*Pper24ISO*-T_{CYC1}	This work
pKME22	Plasmid containing gRNA expression cassette targeting *ERG4* in ST7574	This work
pKME24	Plasmid containing gRNA expression cassette targeting *ERG5* in ST7574	This work
pKME56	Plasmid containing gRNA expression cassette targeting *Pper24ISO* in KMY23	This work
pKME61	pCfB3035-T_{ADH1}-*Pper7RED*-P_{PGK1}-P_{GPD1}-*upc2-1*-T_{CYC1}	This work
pKME62	pCfB2909-T_{ADH1}-*YEH2*-P_{FBA1}-P_{TDH3}-*ARE2*-T_{CYC1}	This work
pKME63	pCfB2904-T_{ADH1}-*PpCYP87G1*-P_{ADH2}-P_{TEF1}-*AtATR1*-T_{CYC1}	This work
pKME64	pCfB2909-T_{ADH1}-*YEH1*-P_{TEF1}-P_{TDH3}-*ARE2*-T_{CYC1}	This work
pKME73	pCfB3035-P_{GAL1}-*AtATR1*-T_{CYC1}	This work
pKME74	pCfB2904-T_{ADH1}-*PpCYP87G1*^{opt}-P_{TEF1}-P_{TDH3}-*PpCYP88C7*^{opt} -T_{CYC1}	This work
pKME80	pCfB3039-P_{GAL1}-*PpCYP749B2*^{opt}-T_{CYC1}	This work
pKME81	pCfB3039-T_{ADH1}-*PpCYP87G1*^{opt} -P_{TEF1}	This work
pKME82	pCfB3039-P_{TDH3}-*PpCYP88C7*^{opt}-T_{CYC1}	This work
pKME83	pCfB2909-P_{GAL1}-*PpSDR-a*^{opt}-T_{CYC1}	This work
pKME84	pCfB2909-P_{GAL1}-*PpSDR-b*^{opt}-T_{CYC1}	This work
pKME85	pCfB2909-P_{GAL1}-*WsSDR-a*-T_{CYC1}	This work
pKME86	pCfB2909-P_{GAL1}-*WsSDR-b*-T_{CYC1}	This work
pKME87	pCfB2909-P_{ADH2}-*PpCYP87G1*^{opt}-T_{CYC1}	This work
pKME88	pCfB2909-P_{ADH2}-*PpCYP749B2*^{opt}-T_{CYC1}	This work
pKME89	pCfB2909-P_{ADH2}-*PpCYP88C7*^{opt}-T_{CYC1}	This work

Supplementary Table 11. List of guide RNA sequences for gene deletions in yeast.

Target gene name	gRNA sequence 5' – 3'
ERG4 (*YGL012W*)	GGCTTCAGCCATTTACCCTG
ERG5 (*YMR015C*)	GTGTAGCGTAGAATTATGAG
Pper24ISO	TTCGTCCCAAGAGGGAGACG

Supplementary Table 12. Purification of ergosta-5,25-diene-3β,24ξ-diol (7) heterologously produced in *N. benthamiana*.

Ergosta-5,25-diene-3β,24ξ-diol (**7**)	**Extraction solvent**		Hexane	**Plant dry weight**	78 g
	**Volume**		1.8 L	**Crude extract**	1,080 mg
	**Instrument**	**Column**	**Solvents**	**Gradient**	**Yield**
	Biotage	SNAP KP-Sil 25g	A: Petroleum ether<br>B: Ethyl acetate	0-25% B (10 CV)<br>25-30% B (3 CV)<br>0% B (8 CV)<br>30-100% B (3 CV)<br>100% B (5 CV)	9 mg
	LC-MS	Kinetex 5 µm C18 100 Å<br>250 x 10 mm	A: H_2O/NH_4OAc<br>B: Methanol/NH_4OAc<br>Flowrate: 5 mL/min	85% B (4 min)<br>85-90% B (10 min)<br>100% B (2 min)<br>85% B (2 min)	2 mg

Supplementary Table 13. Purification of ergosta-5,24(28)-diene-3β,25-diol (8) heterologously produced in *N. benthamiana*.

Ergosta-5,24(28)-diene-3β,25-diol **(8)**	**Extraction solvent**		Hexane	**Plant dry weight**	78 g
	**Volume**		1.8 L	**Crude extract**	1,080 mg
	**Instrument**	**Column**	**Solvents**	**Gradient**	**Yield**
	Biotage	SNAP KP-Sil 25g	A: Petroleum ether<br>B: Ethyl acetate	0-25% B (10 CV)<br>25-30% B (3 CV)<br>30% B (8 CV)<br>30-100% B (3 CV)<br>100% B (5 CV)	34 mg
	LC-MS	Kinetex 5 µm C18 100 Å<br>250 x 10 mm	A: H_2O/NH_4OAc<br>B: Methanol/NH_4OAc<br>Flowrate: 4.5 mL/min	85% B (3 min)<br>85-90% B (10 min)<br>100% B (3 min)<br>85% B (3 min)	2 mg

Supplementary Table 14. Purification of (22*R*)-ergosta-5,24-diene-3β,22-diol (10) heterologously produced in *N. benthamiana*.

(22*R*)-Ergosta-5,24-diene-3β,22-diol **(10)**	**Extraction solvent**		Hexane	**Plant dry weight**	78 g
	**Volume**		1.8 L	**Crude extract:**	1,080 mg
	**Instrument**	**Column**	**Solvents**	**Gradient**	**Yield**
	Biotage	SNAP KP-Sil 25g	A: Petroleum ether<br>B: Ethyl acetate	0-25% B (10 CV)<br>25-30% B (3 CV)<br>30% B (8 CV)<br>30-100% B (3 CV)<br>100% B (5 CV)	16 mg
	LC-MS	Luna 5 µm C8 100 Å<br>250 x 10 mm	A: H_2O/NH_4OAc<br>B: Methanol/NH_4OAc<br>Flowrate: 5 mL/min	85-90% B (8 min)<br>90%-100% B (3 min)<br>100% B (2 min)<br>85% B (2 min)	3 mg

Supplementary Table 15. Purification of (22*R*)-ergosta-5,24(28)-diene-3β,22,25-triol (phyministerol A) (12) heterologously produced in *N. benthamiana*.

(22*R*)-Ergosta-5,24(28)-diene-3β,22,25-triol (phyministerol A) (**12**)	**Extraction solvent**		Ethyl acetate	**Plant dry weight**	45 g
	**Volume**		1.2 L	**Crude extract**	1,847 mg
	**Instrument**	**Column**	**Solvents**	**Gradient**	**Yield**
	Biotage	Sfaer Silica HC D 10g<br>Sfaer Silica HC D 5g (2x)	A: Petroleum ether<br>B: Ethyl acetate	0-30% B (10 CV)<br>30% B (10 CV)<br>30-100% B (3 CV)<br>100% B (5 CV)	33 mg
	Biotage	Sfaer Silica HC D 5g	A: Petroleum ether<br>B: Ethyl acetate	70% B (12 CV)<br>70-100% B (2 CV)<br>100% B (3 CV)	14 mg
	LC-MS	Luna 5 µm<br>C8 100 Å<br>250 x 10 mm	A: H_2O/NH_4OAc<br>B: Methanol/NH_4OAc<br>Flowrate: 5 mL/min	85-100% B (10 min)<br>100% B (3 min)<br>100-85% B (0.1 min)<br>85% B (2 min)	2 mg

Supplementary Table 16. Purification of (22*R*)-24,25-epoxy-ergost-5-ene-3β,22-diol (13) heterologously produced in *N. benthamiana*.

(22*R*)-24,25-Epoxy-ergost-5-ene-3β,22-diol (**13**)	**Extraction solvent**		Hexane	**Plant dry weight**	78 g
	**Volume**		1.8 L	**Crude extract**	1,080 mg
	**Instrument**	**Column**	**Solvents**	**Gradient**	**Yield**
	Biotage	SNAP KP-Sil 25g	A: Petroleum ether<br>B: Ethyl acetate	0-25% B (10 CV)<br>25-30% B (3 CV)<br>30% B (8 CV)<br>30-100% B (3 CV)<br>100% B (5 CV)	17 mg
	LC-MS	Kinetex 5 µm C18 100 Å 250 x 10 mm	A: H_2O/NH_4OAc<br>B: Methanol/NH_4OAc<br>Flowrate: 5 mL/min	85% B (4 min)<br>85-90% B (10 min)<br>100% B (2 min)<br>85% B (2 min)	1 mg

Supplementary Table 17. Purification of (22*R*)-ergosta-5,24(28)-diene-1α,3β,22,25-tetrol (15) heterologously produced in *N. benthamiana*.

FA: Formic acid

<table>
<tr><td rowspan="6">(22R)-Ergosta-5,24(28)-diene-1α,3β,22,25-tetrol (15)</td><td colspan="2">Extraction solvent</td><td>Ethyl acetate</td><td>Plant dry weight:</td><td>78 g</td></tr>
<tr><td colspan="2">Volume</td><td>1 L</td><td>Crude extract</td><td>4,453 mg</td></tr>
<tr><td>Instrument</td><td>Column</td><td>Solvents</td><td>Gradient</td><td>Yield</td></tr>
<tr><td>Biotage</td><td>SNAP KP Sil 100g</td><td>A: Petroleum ether
B: Ethyl acetate</td><td>0-100% B (11 CV)
100% B (3 CV)</td><td>206 mg</td></tr>
<tr><td>Biotage</td><td>Sfaer C18 D 12g</td><td>A: Water
B: Acetonitrile</td><td>10-90% B (15 CV)
90-100% B (1 CV)
100% B (5 CV)</td><td>10 mg</td></tr>
<tr><td>LC-MS</td><td>Kinetex 5 µm C18
100 Å 250 x 10 mm</td><td>A: H_2O/FA
B: Acetonitrile/FA
Flowrate: 5 mL/min</td><td>40-78.6% B (10 min)
78.6-90% B (1 min)
90% B (2 min)
90-40% B (0.1 min)
40% B (2 min)</td><td>1 mg</td></tr>
</table>

Supplementary Table 18. X-ray crystallographic data of (22*S*)-6*β*-methoxy-3*α*,5-cycloergosta-24,25-diene-26,22-lactone (S6-(22*S*)).

**Parameter**	**Value**
Identification code	MB293twin_twin1_hklf4
ccdc deposition number	2369097
Empirical formula	$C_{29}H_{44}O_3$
Formula weight	440.64
Temperature/K	100.00(10)
Crystal system	monoclinic
Space group	$P2_1$
a/Å	7.57280(10)
b/Å	20.7677(5)
c/Å	15.8495(4)
α/°	90
β/°	93.261(2)
γ/°	90
Volume/Å^3	2488.61(9)
Z	4
ρ_{calc}g/cm^3	1.176
μ/mm^{-1}	0.570
F(000)	968.0
Crystal size/mm^3	0.466 × 0.362 × 0.08
Radiation	Cu Kα (λ = 1.54184)
2Θ range for data collection/°	5.584 to 159.238
Index ranges	$-9 \le h \le 9$, $-25 \le k \le 25$, $-20 \le l \le 20$
Reflections collected	14886
Independent reflections	14886 [R_{int} = 0.048, R_{sigma} = 0.0209]
Data/restraints/parameters	14886/1/590
Goodness-of-fit on F^2	1.109
Final R indexes [I>=2σ (I)]	R_1 = 0.0452, wR_2 = 0.1238
Final R indexes [all data]	R_1 = 0.0483, wR_2 = 0.1262
Largest diff. peak/hole / e Å^{-3}	0.20/-0.26
Flack parameter	-0.11(14)

Supplementary Table 19. List of fragments for VIGS in *W. somnifera*.

Overhangs for In-Fusion cloning are highlighted in bold and underlined.

Name (Insertion site)	Sequence	Purpose
PDS (EcoRI-BamHI)	**TAAGGTTACCGAATT**GCCGTTTTGATTTCCCCGAAGCTTTACCTGCTCCTTTAAATGGAATTTTGGCCATCCTGAAAAACAATGAAATGCTTACATGGCCAGAGAAAGTCAAATTTGCAATCGGACTTTTGCCAGCAATGCTTGGAGGGCAATCTTATGTTGAAGCTCAAGACGGAATAAGTGTTAAGGACTGGATGAAAAAGCAAGGCGTGCCGGATAGGGTGACAGATGAGGTGTTCATTGCCATGTCAAAGGCGCTTAACTTTATAAACCCTGATGAGCTTTCAATGCAGTGCATCTTAATCGCGCTGAACAGGTTTCTTCAGGAGAAACATGGTTCAAAAATGGCCTTTTTAGATGGTAATCCTCCTGAGAGACTTTGCATGCCGATTGTTGAACATATCGAGTCAAAAGGT**GATCCGGTACCGAGC**	Construction of pTRV2-*PDS*: consists of base pairs 617-1017 of *PDS* + overhangs for In-Fusion cloning into pTRV2-MCS linearised with EcoRI and BamHI
GFP (EcoRI-BamHI)	**TAAGGTTACCGAATT**TCAAGATACCCAGATCATATGAAACGGCATGACTTTTTCAAGAGTGCCATGCCCGAAGGTTATGTACAGGAAAGAACTATATTTTTCAAAGATGACGGGAACTACAAGACACGTGCTGAAGTCAAGTTTGAAGGTGATACCCTTGTTAATAGAATCGAGTTAAAAGGTATTGATTTTAAAGAAGATGGAAACATTCTTGGACACAAATTGGAATACAACTATAACTCACACAATGTATACATCATGGCAGACAAACAAAAGAATGGAATCAAAGTTAACTTCAAAATTAGACACAACAT**GATCCGGTACCGAGC**	Construction of pTRV2-*GFP*: consists of base pairs 214-512 of *Aequorea victoria GFP* + overhangs for In-Fusion cloning into pTRV2-MCS linearised with EcoRI and BamHI
GFP (KpnI-XhoI)	**TCAAAAGGTGATCCG**TCAAGATACCCAGATCATATGAAACGGCATGACTTTTTCAAGAGTGCCATGCCCGAAGGTTATGTACAGGAAAGAACTATATTTTTCAAAGATGACGGGAACTACAAGACACGTGCTGAAGTCAAGTTTGAAGGTGATACCCTTGTTAATAGAATCGAGTTAAAAGGTATTGATTTTAAAGAAGATGGAAACATTCTTGGACACAAATTGGAATACAACTATAACTCACACAATGTATACATCATGGCAGACAAACAAAAGAATGGAATCAAAGTTAACTTCAAAATTAGACACAACAT**TCGAGGCCCGGGCAT**	Construction of pTRV2-*PDS-GFP*: consists of base pairs 214-512 of *Aequorea victoria GFP* + overhangs for In-Fusion cloning into pTRV2-*PDS* linearised with KpnI and XhoI
24ISO (KpnI-XhoI)	**TCAAAAGGTGATCCG**ATGATGCCACCCAAAGTTGCTGTGCTTAAGGCCACCCAAAGCGAGTATATCAGGAATCCCACACAGAATCTACAAGCTGCCAGTGAAATCTATGATCCCCTATGATGGAGCAAAGAAATGCCATGACGTGGAGCTTTTTCTAATC**TCGAGGCCCGGGCAT**	Construction of pTRV2-*PDS-24ISO*: consists of *Ws24ISO-b* fragments analogous to VIGS construct by Knoch *et al.*[59] + overhangs for In-Fusion cloning into pTRV2-*PDS* linearised with KpnI and XhoI
CYP87G1 (KpnI-XhoI)	**TCAAAAGGTGATCCG**AAAGCCTTTGTGAAAGGCTTTCTCTCATTCCCTATCAATTTGCCCGGAACAGCTTTTCATTCAGGTTTACAGGGGCGTAAAAGTGCTATAAAGATGATCAAAGACATTTTCGAGAAAAGGAGGTCATCAAAGGAGAACGCAAATGAACAAGACTTCATTGATCACCTACTTCAAGAAATAGACAAGGAGGAGACATTTATAACAGAAGACACAGCAGTGGATCTGATCTTCTTTGTTATATTTGCGGCTCATGAAACCACTTCTTCAACCATGACACTACTCTTTAAATACTTCACAGAG**TCGAGGCCCGGGCAT**	Construction of pTRV2-*PDS-CYP87G1*: consists of base pairs 601-900 of *WsCYP87G-b* + overhangs for In-Fusion cloning into pTRV2-*PDS* linearised with KpnI and XhoI
CYP88C7 (KpnI-XhoI)	**TCAAAAGGTGATCCG**AAGTCATGCATTAGTATCTTACTTCTTCGATTTCTTAACTGAAACTGTGAGAAGTACGTTCGAGAAATGGGCCACTACCGGAGAAACACTTCAGTTACTCTTTGAGATGAAAAAACCTACATTTAAAGTGCTTATGCAAATACTTATAGGTGGCAATCAAGTTGAAAATGAATTGCTTGACGCTTTGTTCAAGGAGAACAATTTCCGATTTGCTGGTCTTCGTAGCATGCCCCTCGATTTCCCCGGATCTACTTACAACAGGGCAATGAAGGGTCGAGGAGAAATAGTTAAGATATACGA**TCGAGGCCCGGGCAT**	Construction of pTRV2-*PDS-CYP88C7*: consists of base pairs 444-743 of *WsCYP88C-d* + overhangs for In-Fusion cloning into pTRV2-*PDS* linearised with KpnI and XhoI
CYP749B2 (KpnI-XhoI)	**TCAAAAGGTGATCCG**TAAATTTTTCCGGTCATCTGAAGAGATACAGGCGGATAAAATCCTTGAAGAACTCAGTGACTCGTTCGCAGGAATCATAAAGAAAAGAGAAGATAGAGTTAAGGCAGGAGAAGTAGTTAATTTTGGGGATGATTTTCTTGCATCACTCTTAGAGGGTCGCTTTAATGCTGATGAGATTGCAAGAATCTCAGTGGCTGAAATCATTGAAGAATGCAAATCTTTCTATTTTGCTGGACATAAAACAGTGACTAGTTTGCTAAGCTGGTGCATGCTTCTCTTAGCAAGCAACATGGATTGGCA**TCGAGGCCCGGGCAT**	Construction of pTRV2-*PDS-CYP749B2*: consists of base pairs 693-992 of *WsCYP749B-b* + overhangs for In-Fusion cloning into pTRV2-*PDS* linearised with KpnI and XhoI
SDR (KpnI-XhoI)	**TCAAAAGGTGATCCG**CTGACGAGAAACAAGTCCAATCATTGGTAAAAACAACGTTACAAATTCATGGTCGACTCGATATTATGTTCAGTAATGCCGGAATTTTCAGTGCTGACGACGGAAAACAAGTTATGCTAGGTTTTAACCTGGATGGTTTAGACAAGGTAATGAGTATCAACGTTCGAGGATCAGCTGCGTGTGTGAAGTACGCGGCGGAGGCAATGGTGGAGGGAGGCGTTAAAGGGAAAATACTATGCACGGGGAGTTCGGTGACTTTGATGGGGGCGTCCAATCATATTGACTACGCTATGTCGAAGCA**TCGAGGCCCGGGCAT**	Construction of pTRV2-*PDS-SDR*: consists of base pairs 197-497 of *WsSDR-b* + overhangs for In-Fusion cloning into pTRV2-*PDS* linearised with KpnI and XhoI

Supplementary references

1. Ou, S. *et al.* Benchmarking transposable element annotation methods for creation of a streamlined, comprehensive pipeline. *Genome Biol.* **20**, 275 (2019).
2. Xiong, W., He, L., Lai, J., Dooner, H. K. & Du, C. HelitronScanner uncovers a large overlooked cache of Helitron transposons in many plant genomes. *Proc. Natl. Acad. Sci. U. S. A.* **111**, 10263–10268 (2014).
3. Xu, Z. & Wang, H. LTR_FINDER: an efficient tool for the prediction of full-length LTR retrotransposons. *Nucleic Acids Res.* **35**, W265–W268 (2007).
4. Ou, S. & Jiang, N. LTR_FINDER_parallel: parallelization of LTR_FINDER enabling rapid identification of long terminal repeat retrotransposons. *Mob. DNA* **10**, 48 (2019).
5. Ou, S. & Jiang, N. LTR_retriever: A highly accurate and sensitive program for identification of long terminal repeat retrotransposons. *Plant Physiol.* **176**, 1410–1422 (2018).
6. Ellinghaus, D., Kurtz, S. & Willhoeft, U. LTRharvest, an efficient and flexible software for de novo detection of LTR retrotransposons. *BMC Bioinformatics* **9**, 18 (2008).
7. Su, W., Gu, X. & Peterson, T. TIR-Learner, a new ensemble method for TIR transposable element annotation, provides evidence for abundant new transposable elements in the maize genome. *Mol. Plant* **12**, 447–460 (2019).
8. Shi, J. & Liang, C. Generic Repeat Finder: A high-sensitivity tool for genome-wide *de novo* repeat detection. *Plant Physiol.* **180**, 1803–1815 (2019).
9. Zhang, R.-G. *et al.* TEsorter: An accurate and fast method to classify LTR-retrotransposons in plant genomes. *Hortic. Res.* **9**, uhac017 (2022).
10. Benson, G. Tandem repeats finder: a program to analyze DNA sequences. *Nucleic Acids Res.* **27**, 573–580 (1999).
11. Velásquez, A. C., Chakravarthy, S. & Martin, G. B. Virus-induced gene silencing (VIGS) in *Nicotiana benthamiana* and tomato. *J. Vis. Exp. JoVE* e1292 (2009) doi:10.3791/1292.
12. Fernandez-Pozo, N., Rosli, H. G., Martin, G. B. & Mueller, L. A. The SGN VIGS tool: User-friendly software to design virus-induced gene silencing (VIGS) constructs for functional genomics. *Mol. Plant* **8**, 486–488 (2015).
13. Brosa, C., Nusimovich, S. & Peracaula, R. Synthesis of new brassinosteroids with potential activity as antiecdysteroids. *Steroids* **59**, 463–467 (1994).
14. Rosenbaum, N. *et al.* Formal semisynthesis of demethylgorgosterol utilizing a stereoselective intermolecular cyclopropanation reaction. *Eur. J. Org. Chem.* **2021**, 1568–1574 (2021).
15. Schwartz, C., Raible, J., Mott, K. & Dussault, P. H. Fragmentation of carbonyl oxides by *N*-oxides: An improved approach to alkene ozonolysis. *Org. Lett.* **8**, 3199–3201 (2006).

16. Matsuya, Y. *et al.* Synthesis of sominone and its derivatives based on an RCM strategy: Discovery of a novel anti-Alzheimer's disease medicine candidate "Denosomin". *Org. Lett.* **11**, 3970–3973 (2009).
17. Sheldrick, G. M. SHELXT – Integrated space-group and crystal-structure determination. *Acta Crystallogr. Sect. Found. Adv.* **71**, 3–8 (2015).
18. Dolomanov, O. V., Bourhis, L. J., Gildea, R. J., Howard, J. a. K. & Puschmann, H. OLEX2: A complete structure solution, refinement and analysis program. *J. Appl. Crystallogr.* **42**, 339–341 (2009).
19. Sheldrick, G. M. Crystal structure refinement with SHELXL. *Acta Crystallogr. Sect. C Struct. Chem.* **71**, 3–8 (2015).
20. Chung, S.-K. *et al.* Synthesis and bioactivities of steroid derivatives as antifungal agents. *Tetrahedron* **54**, 15899–15914 (1998).
21. Elliger, C. A. *et al.* Petuniasterones, novel ergostane-type steroids of *Petunia hybridia* Vilm. (Solanaceae) having insect-inhibitory activity. X-ray molecular structure of the 22,24,25-[(methoxycarbonyl)orthoacetate] of 7α,22,24,25-tetrahydroxy ergosta-1,4-dien-3-one and of 1α-acetoxy-24,25-epoxy-7α-hydroxy-22-(methylthiocarbonyl)acetoxyergost-4-en-3-one. *J. Chem. Soc. Perkin 1* 711–717 (1988) doi:10.1039/P19880000711.
22. Withania somnifera genome assembly ASM3965448v1. *NCBI* https://www.ncbi.nlm.nih.gov/datasets/genome/GCA_039654485.1/.
23. Takahashi, K. *et al.* Metabolic conversion of 24-methyl-Δ25-cholesterol to 24-methylcholesterol in higher plants. *Bioorg. Med. Chem.* **14**, 732–738 (2006).
24. Wu, J. *et al.* New steroids with anti-inflammatory activity from the whole plants of *Physalis minima*. *Nat. Prod. Res.* (2024) doi:10.1080/14786419.2024.2340048.
25. Shilpashree, H. B., Sudharshan, S. J., Shasany, A. K. & Nagegowda, D. A. Molecular characterization of three CYP450 genes reveals their role in withanolides formation and defense in *Withania somnifera*, the Indian Ginseng. *Sci. Rep.* **12**, 1602 (2022).
26. Hua, C. *et al.* Identification of P450 candidates associated with the biosynthesis of physalin-class compounds in *Physalis angulata*. *Int. J. Mol. Sci.* **24**, 14077 (2023).
27. Shilpashree, H. B., Narayanan, A. K., Kumar, S. R., Barvkar, V. & Nagegowda, D. A. The cytochrome P450 enzyme WsCYP71B35 from *Withania somnifera* has a role in withanolides biosynthesis and defence against bacteria. *Physiol. Plant.* **176**, e14180 (2024).
28. Anjali, P., Narayanan, A. K., Parihar, D., Patil, A. & Nagegowda, D. A. Functional characterization of two glycosyltransferases from *Withania somnifera* illuminates their role in withanosides biosynthesis and defence against bacteria. 2024.02.12.579880 Preprint at https://doi.org/10.1101/2024.02.12.579880 (2024).

29. Sharma, A. *et al.* Characterization and overexpression of sterol Δ22-desaturase, a key enzyme modulates the biosyntheses of stigmasterol and withanolides in *Withania somnifera* (L.) Dunal. *Plant Sci.* **301**, 110642 (2020).
30. Sharma, A., Rather, G. A., Misra, P., Dhar, M. K. & Lattoo, S. K. Gene silencing and over-expression studies in concurrence with promoter specific elicitations reveal the central role of WsCYP85A69 in biosynthesis of triterpenoids in *Withania somnifera* (L.) Dunal. *Front. Plant Sci.* **10**, 842 (2019).
31. Srivastava, S. *et al.* Light and auxin responsive cytochrome P450s from *Withania somnifera* Dunal: Cloning, expression and molecular modelling of two pairs of homologue genes with differential regulation. *Protoplasma* **252**, 1421–1437 (2015).
32. Zhang, F. *et al.* Revealing evolution of tropane alkaloid biosynthesis by analyzing two genomes in the Solanaceae family. *Nat. Commun.* **14**, 1446 (2023).
33. Hulse-Kemp, A. M. *et al.* Reference quality assembly of the 3.5-Gb genome of *Capsicum annuum* from a single linked-read library. *Hortic. Res.* **5**, 4 (2018).
34. Kim, S. *et al.* New reference genome sequences of hot pepper reveal the massive evolution of plant disease-resistance genes by retroduplication. *Genome Biol.* **18**, 210 (2017).
35. Goldberg, J. K., Olcerst, A., McKibben, M., Hare, D. J. & Bronstein, J. A *de novo* long-read genome assembly is contiguous and complete enough to study paleo-polyploidy and herbivore-induced transcriptional responses of the sacred datura plant (*Datura wrightii*). 2023.05.08.539846 Preprint at https://doi.org/10.1101/2023.05.08.539846 (2023).
36. Powell, A. F. *et al.* Genome sequence for the blue-flowered Andean shrub *Iochroma cyaneum* reveals extensive discordance across the berry clade of Solanaceae. *Plant Genome* **15**, e20223 (2022).
37. Hoshino, A. *et al.* Genome sequence and analysis of the Japanese morning glory *Ipomoea nil*. *Nat. Commun.* **7**, 13295 (2016).
38. Cao, Y.-L. *et al.* Wolfberry genomes and the evolution of Lycium (Solanaceae). *Commun. Biol.* **4**, 671 (2021).
39. Xu, S. *et al.* Wild tobacco genomes reveal the evolution of nicotine biosynthesis. *Proc. Natl. Acad. Sci. U. S. A.* **114**, 6133–6138 (2017).
40. Sierro, N. *et al.* The tobacco genome sequence and its comparison with those of tomato and potato. *Nat. Commun.* **5**, 3833 (2014).
41. Sierro, N. *et al.* Reference genomes and transcriptomes of *Nicotiana sylvestris* and *Nicotiana tomentosiformis*. *Genome Biol.* **14**, R60 (2013).
42. Bombarely, A. *et al.* Insight into the evolution of the Solanaceae from the parental genomes of *Petunia hybrida*. *Nat. Plants* **2**, 16074 (2016).
43. Lu, J. *et al.* The Physalis floridana genome provides insights into the biochemical and morphological evolution of Physalis fruits. *Hortic. Res.* **8**, 244 (2021).

44. Wu, M. *et al.* Inferring the genetic basis of sex determination from the genome of a dioecious nightshade. *Mol. Biol. Evol.* **38**, 2946–2957 (2021).
45. Stam, R. *et al.* The *de novo* reference genome and transcriptome assemblies of the wild tomato species *Solanum chilense* highlights birth and death of NLR genes between tomato species. *G3 Genes Genomes Genetics* **9**, 3933–3941 (2019).
46. Christenhusz, M. J. M. *et al.* The genome sequence of bittersweet, *Solanum dulcamara* L. (Solanaceae). *Wellcome Open Res.* **8**, 409 (2023).
47. Hosmani, P. S. *et al.* An improved de novo assembly and annotation of the tomato reference genome using single-molecule sequencing, Hi-C proximity ligation and optical maps. 767764 Preprint at https://doi.org/10.1101/767764 (2019).
48. Barchi, L. *et al.* Improved genome assembly and pan-genome provide key insights into eggplant domestication and breeding. *Plant J.* **107**, 579–596 (2021).
49. Achakkagari, S. R., Tai, H. H., Davidson, C., De Jong, H. & Strömvik, M. V. The complete mitogenome assemblies of 10 diploid potato clones reveal recombination and overlapping variants. *DNA Res.* **28**, dsab009 (2021).
50. Bolger, A. *et al.* The genome of the stress-tolerant wild tomato species *Solanum pennellii*. *Nat. Genet.* **46**, 1034–1038 (2014).
51. Wang, X. *et al.* Genome of *Solanum pimpinellifolium* provides insights into structural variants during tomato breeding. *Nat. Commun.* **11**, 5817 (2020).
52. Molitor, C. *et al.* De novo genome assembly of *Solanum sitiens* reveals structural variation associated with drought and salinity tolerance. *Bioinformatics* **37**, 1941–1945 (2021).
53. Yan, L. *et al.* Genome assembly of primitive cultivated potato *Solanum stenotomum* provides insights into potato evolution. *G3 GenesGenomesGenetics* **11**, jkab262 (2021).
54. Pham, G. M. *et al.* Construction of a chromosome-scale long-read reference genome assembly for potato. *GigaScience* **9**, giaa100 (2020).
55. Dias, S. L. *et al.* Biosynthesis of the allelopathic alkaloid gramine in barley by a cryptic oxidative rearrangement. *Science* **383**, 1448–1454 (2024).
56. Jessop-Fabre, M. M. *et al.* EasyClone-MarkerFree: A vector toolkit for marker-less integration of genes into *Saccharomyces cerevisiae* via CRISPR-Cas9. *Biotechnol. J.* **11**, 1110–1117 (2016).
57. Urban, P., Mignotte, C., Kazmaier, M., Delorme, F. & Pompon, D. Cloning, Yeast expression, and characterization of the coupling of two distantly related *Arabidopsis thaliana* NADPH-cytochrome P450 reductases with P450 CYP73A5. *J. Biol. Chem.* **272**, 19176–19186 (1997).
58. Milne, N., Tramontin, L. R. R. & Borodina, I. A teaching protocol demonstrating the use of EasyClone and CRISPR/Cas9 for metabolic engineering of Saccharomyces cerevisiae and *Yarrowia lipolytica*. *FEMS Yeast Res.* **20**, foz062 (2020).

59. Knoch, E. *et al.* Third DWF1 paralog in Solanaceae, sterol Δ24-isomerase, branches withanolide biosynthesis from the general phytosterol pathway. *Proc. Natl. Acad. Sci. U. S. A.* **115**, E8096–E8103 (2018).
