# Supplementary material for: Phylogenomics and metabolic engineering reveal a conserved gene cluster in Solanaceae plants for withanolide biosynthesis
Source: Nat Commun. 2025 Jul 10;16:6367. doi: 10.1038/s41467-025-61686-1 (PMC12246201; doi:10.1038/s41467-025-61686-1)
Supplement: Supplementary file 3 — Description of Additional Supplementary Files [file 41467_2025_61686_MOESM3_ESM.pdf]

Description of Additional Supplementary Files

File Name: Supplementary Data 1
Description: List of genes in withanolide gene clusters.

File Name: Supplementary Data 2
Description: Overview of electron impact fragmentations and proposed fragments of compounds investigated in this work (TMS ethers).

File Name: Supplementary Data 3
Description: NMR data of isolated compounds compared with [26,27- 2H6]24- methyldesmosterol (2) (Takahashi et al.).

File Name: Supplementary Data 4
Description: List of primers.

File Name: Supplementary Data 5
Description: Nucleotide sequences for metabolic engineering in S. cerevisiae used in this study.
