# Supplementary material for: Phylogenomics and metabolic engineering reveal a conserved gene cluster in Solanaceae plants for withanolide biosynthesis
Source: Nat Commun. 2025 Jul 10;16:6367. doi: 10.1038/s41467-025-61686-1 (PMC12246201; doi:10.1038/s41467-025-61686-1)
Supplement: Supplementary file 9 — Reporting Summary [file 41467_2025_61686_MOESM9_ESM.pdf]

Corresponding author(s): Pucker, Franke

Last updated by author(s): Jun 19, 2025

Reporting Summary

Nature Portfolio wishes to improve the reproducibility of the work that we publish. This form provides structure for consistency and transparency in reporting. For further information on Nature Portfolio policies, see our Editorial Policies and the Editorial Policy Checklist.

Statistics

For all statistical analyses, confirm that the following items are present in the figure legend, table legend, main text, or Methods section.

n/a	Confirmed	
☐	☒	The exact sample size (*n*) for each experimental group/condition, given as a discrete number and unit of measurement
☐	☒	A statement on whether measurements were taken from distinct samples or whether the same sample was measured repeatedly
☐	☒	The statistical test(s) used AND whether they are one- or two-sided<br>*Only common tests should be described solely by name; describe more complex techniques in the Methods section.*
☒	☐	A description of all covariates tested
☒	☐	A description of any assumptions or corrections, such as tests of normality and adjustment for multiple comparisons
☐	☒	A full description of the statistical parameters including central tendency (e.g. means) or other basic estimates (e.g. regression coefficient) AND variation (e.g. standard deviation) or associated estimates of uncertainty (e.g. confidence intervals)
☐	☒	For null hypothesis testing, the test statistic (e.g. *F*, *t*, *r*) with confidence intervals, effect sizes, degrees of freedom and *P* value noted<br>*Give P values as exact values whenever suitable.*
☒	☐	For Bayesian analysis, information on the choice of priors and Markov chain Monte Carlo settings
☒	☐	For hierarchical and complex designs, identification of the appropriate level for tests and full reporting of outcomes
☒	☐	Estimates of effect sizes (e.g. Cohen's *d*, Pearson's *r*), indicating how they were calculated

Our web collection on statistics for biologists contains articles on many of the points above.

Software and code

Policy information about availability of computer code

Data collection	https://github.com/NancyChoudhary28/Withanolide_biosynthesis or https://doi.org/10.5281/zenodo.15632852
Data analysis	Custom code was deposited on GitHub: https://github.com/NancyChoudhary28/Withanolide_biosynthesis or https://doi.org/10.5281/zenodo.15632852<br>GC-MS data was analysed with Agilent MSD ChemStation F.01.03.2357. LC-MS data was analysed with Agilent OpenLab CDS ChemStation C.01.10. NMR data was analysed with TopSpin (version 4.1.3) or MestReNova (version 14.3.1).<br>Bioinformatics tools used are described in detail in the Methods section (Guppy v 6.4.6+ae70e8f, NextDenovo2 v2.5.2, RagTag v2.1.0, HISAT 2.2.1, GeMoMa v1.9, MMseqs2 (version 15-6f452), BUSCO v5.8.2, Extensive de-novo TE Annotator (EDTA) v2.2.1, Tandem Repeats Finder (TRF) v4.10.0, JCVI/MCscan v1.4.14, MAFFT v7.520, iTOL v6.8, NCBI SRA toolkit v3.1.0, Kallisto v0.50.1).

For manuscripts utilizing custom algorithms or software that are central to the research but not yet described in published literature, software must be made available to editors and reviewers. We strongly encourage code deposition in a community repository (e.g. GitHub). See the Nature Portfolio guidelines for submitting code & software for further information.

Data

Policy information about availability of data

All manuscripts must include a data availability statement. This statement should provide the following information, where applicable:

- Accession codes, unique identifiers, or web links for publicly available datasets
- A description of any restrictions on data availability
- For clinical datasets or third party data, please ensure that the statement adheres to our policy

Sequences of genes from the withanolide gene clusters shown here are provided as Supplementary Data 1 in xlsx format. Raw sequencing reads are available from the European Nucleotide Archive: Withania somnifera genomic data (PRJEB64854) and corresponding RNA-seq data (ERR13615536). The assembled genome sequence and corresponding annotation of W. somnifera is available via GenBank (GCA_965601375) or LeoPARD (https://leopard.tu-braunschweig.de/receive/dbbs_mods_00078608). Re-annotations of the Physalis genome sequences are available via LeoPARD (https://leopard.tu-braunschweig.de/receive/dbbs_mods_00077980) or GitHub (https://github.com/NancyChoudhary28/Withanolide_biosynthesis/tree/main/data). Scripts and commands of the Circos plot generation are described in a GitHub repository (https://github.com/NancyChoudhary28/Withanolide_biosynthesis or https://doi.org/10.5281/zenodo.15632852).

Research involving human participants, their data, or biological material

Policy information about studies with human participants or human data. See also policy information about sex, gender (identity/presentation), and sexual orientation and race, ethnicity and racism.

Reporting on sex and gender	Does not apply.
Reporting on race, ethnicity, or other socially relevant groupings	Does not apply.
Population characteristics	Does not apply.
Recruitment	Does not apply.
Ethics oversight	Does not apply.

Note that full information on the approval of the study protocol must also be provided in the manuscript.

Field-specific reporting

Please select the one below that is the best fit for your research. If you are not sure, read the appropriate sections before making your selection.

[x] Life sciences　[ ] Behavioural & social sciences　[ ] Ecological, evolutionary & environmental sciences

For a reference copy of the document with all sections, see nature.com/documents/nr-reporting-summary-flat.pdf

Life sciences study design

All studies must disclose on these points even when the disclosure is negative.

Sample size	No sample size calculation was carried out. For virus-induced gene silencing, 3-6 replicates that showed a photobleaching phenotype were used. All other experiments were carried out in triplicates. Triplicates were sufficient to obtain clear trends.
Data exclusions	No data was excluded.
Replication	All results were observed in at least three independent biological replicates. Virus-induced gene silencing results were confirmed in a second independent experiment. All attempts at replication were successful.
Randomization	Positions of yeast cultures in shakers and of N. benthamiana plants in the greenhouse or phytochamber were randomised.
Blinding	No blinding was used. Automated peak integration of chromatographic data was employed to avoid human bias.

Reporting for specific materials, systems and methods

We require information from authors about some types of materials, experimental systems and methods used in many studies. Here, indicate whether each material, system or method listed is relevant to your study. If you are not sure if a list item applies to your research, read the appropriate section before selecting a response.

Materials & experimental systems

n/a	Involved in the study
x	Antibodies
x	Eukaryotic cell lines
x	Palaeontology and archaeology
x	Animals and other organisms
x	Clinical data
x	Dual use research of concern
	x Plants

Methods

n/a	Involved in the study
x	ChIP-seq
x	Flow cytometry
x	MRI-based neuroimaging

Plants

Seed stocks	Withania somnifera catalogue number XX-0-STGAL—22/1985<br>Nicotiana benthamiana LAB strain: common and widespread model organism
Novel plant genotypes	Nicotiana benthamiana: Transient expression only
Authentication	Does not apply.
